# The ubiquitin-dependent ATPase p97 removes cytotoxic trapped PARP1 from chromatin

**DOI:** 10.1038/s41556-021-00807-6

**Published:** 2022-01-10

**Authors:** Dragomir B. Krastev, Shudong Li, Yilun Sun, Andrew J. Wicks, Gwendoline Hoslett, Daniel Weekes, Luned M. Badder, Eleanor G. Knight, Rebecca Marlow, Mercedes Calvo Pardo, Lu Yu, Tanaji T. Talele, Jiri Bartek, Jyoti S. Choudhary, Yves Pommier, Stephen J. Pettitt, Andrew N. J. Tutt, Kristijan Ramadan, Christopher J. Lord

**Affiliations:** 1 The CRUK Gene Function Laboratory, London, UK; 2 Breast Cancer Now Toby Robins Research Centre, The Institute of Cancer Research, London, UK; 3 MRC Oxford Institute for Radiation Oncology, Department of Oncology, University of Oxford, Oxford, UK; 4 Developmental Therapeutics Branch, Laboratory of Molecular Pharmacology, Center for Cancer Research, National Cancer Institute, NIH, Bethesda, MD USA; 5 The Breast Cancer Now Research Unit, King’s College London, London, UK; 6 Functional Proteomics Laboratory, The Institute of Cancer Research, London, UK; 7 Department of Pharmaceutical Sciences, College of Pharmacy and Health Sciences, St. John’s University, Queens, NY USA; 8 Danish Cancer Society Research Center, Copenhagen, Denmark; 9 Division of Genome Biology, Department of Medical Biochemistry and Biophysics, Science for Life Laboratory, Karolinska Institute, Stockholm, Sweden

**Keywords:** DNA damage response, Targeted therapies, Ubiquitylation, Sumoylation

## Abstract

Poly (ADP-ribose) polymerase (PARP) inhibitors elicit antitumour activity in homologous recombination-defective cancers by trapping PARP1 in a chromatin-bound state. How cells process trapped PARP1 remains unclear. Using wild-type and a trapping-deficient PARP1 mutant combined with rapid immunoprecipitation mass spectrometry of endogenous proteins and Apex2 proximity labelling, we delineated mass spectrometry-based interactomes of trapped and non-trapped PARP1. These analyses identified an interaction between trapped PARP1 and the ubiquitin-regulated p97 ATPase/segregase. We found that following trapping, PARP1 is SUMOylated by PIAS4 and subsequently ubiquitylated by the SUMO-targeted E3 ubiquitin ligase RNF4, events that promote recruitment of p97 and removal of trapped PARP1 from chromatin. Small-molecule p97-complex inhibitors, including a metabolite of the clinically used drug disulfiram (CuET), prolonged PARP1 trapping and enhanced PARP inhibitor-induced cytotoxicity in homologous recombination-defective tumour cells and patient-derived tumour organoids. Together, these results suggest that p97 ATPase plays a key role in the processing of trapped PARP1 and the response of tumour cells to PARP inhibitors.

## Abstract (web summary)

Krastev et al. report that trapped PARP1 undergoes SUMOylation, followed by ubiquitylation, resulting in the recruitment of the p97 ATPase to remove trapped PARP1 from chromatin and prevent PARP inhibitor-induced cytotoxicity.

## Main

Poly (ADP-ribose)-polymerase (PARP) inhibitors (PARPi) selectively kill tumour cells with impaired homologous recombination and are approved for use in homologous recombination-defective breast, ovarian, pancreatic and prostate cancers^1^. PARP1 (also known as ARTD1), the key target of PARPi, is a ubiquitously expressed nuclear enzyme that uses NAD^+^ to synthesise poly(ADP-ribose) (PAR) chains on substrate proteins (heteromodification) and itself (automodification). This catalytic activity (PARylation), which is enhanced by PARP1 binding to damaged DNA, initiates DNA repair by driving the recruitment/concentration of DNA-repair effectors and modulating chromatin structure. Once DNA repair is initiated, PARP1 is released from DNA via auto-PARylation. Most clinical PARPi bind the NAD^+^-binding site (catalytic domain) and inhibit catalytic activity, but also induce chromatin retention of PARP1 (PARP trapping); this latter characteristic is a key driver of PARPi-mediated cytotoxicity^2^. Consistent with this, deletion of *PARP1* causes PARPi resistance, as do in-frame *PARP1* insertion/deletion mutations that impair PARP1 trapping^3^. Moreover, the chemical modification of a PARPi with poor trapping properties into a derivative with enhanced trapping properties, but similar catalytic potency, enhances cytotoxicity^4^. Although it is known that specific PARP1 mutations alter PARP1 trapping^3^, as does modulation of the amount of residual PAR on PARP1 via PAR-glycosylase^5^, how trapped PARP1 is released from damaged DNA is poorly understood.

By generating a series of protein–protein interaction profiles of either trapped or non-trapped PARP1, we show that trapped PARP1 binds p97 ATPase (also known as valosin-containing protein, VCP). p97 ATPase is a hexameric unfoldase/segregase that unfolds and disassembles ubiquitylated substrates through its central pore^6,7^ including Aurora B kinase, CMG helicases, the licensing factor CDT1^8^ and the TOP1-cleavage complex^9–11^. We show that the PARP1–p97 interaction is mediated by sequential PIAS4-mediated SUMOylation and RNF4-mediated ubiquitylation of trapped PARP1. Ufd1-mediated p97 recruitment to trapped and modified PARP1 ultimately leads to the removal of PARP1 from chromatin. In addition, we show that p97 inhibition by a metabolite of the clinically used drug disulfiram, leads to prolonged PARP1 trapping and profound PARPi sensitivity, suggesting an approach to enhance PARPi-induced cytotoxicity. Collectively, our findings suggest that the PARP1–p97 axis is essential for the removal of trapped PARP1 and the cellular response to PARPi.

## Results

### Identification of trapped PARP1-associated proteins

**Fig. 1:**
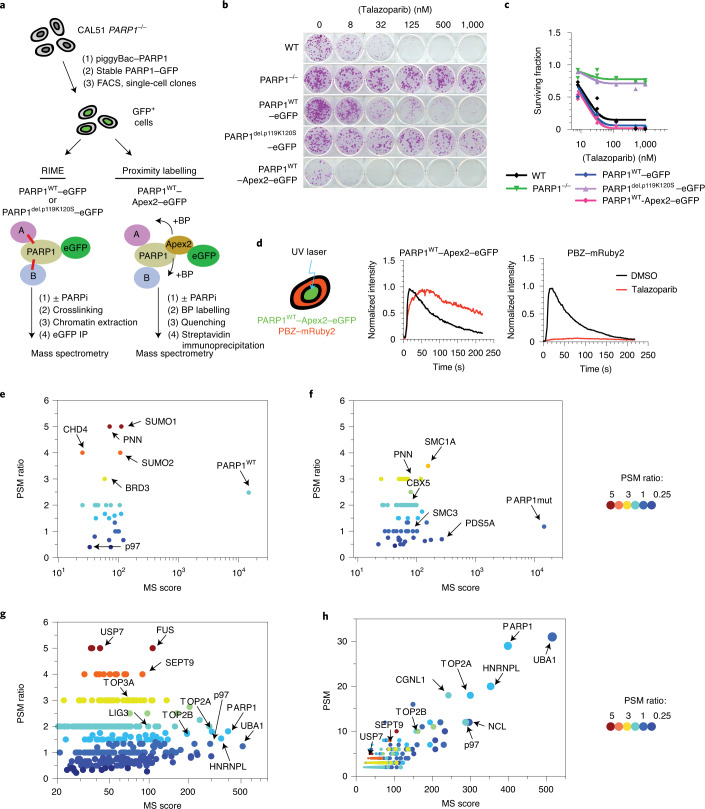
Identification of trapped PARP1-interacting proteins. **a**, Schematic describing the identification of trapped PARP1 protein–protein interactomes via RIME or proximity labelling linked to mass spectrometry. The cells were exposed to either PARPi + MMS (to enable trapping) or MMS (no trapping) for 1 h, after which PARP1-interacting/proximal proteins were identified by mass spectrometry analysis. BP, biotin-phenol. **b**, Clonogenic assay illustrating the restoration of PARPi sensitivity in the complemented *PARP1*^–/–^ CAL51 cells as described in **a**. PARP1 protein expression in the different clones is shown in Extended Data Fig. 1a. **c**, Quantification of the colony formation assay shown in **b**; data are the mean of two biological replicates. **d**, PARP1^WT^–Apex2–eGFP protein localizes to sites of DNA damage, generates PAR and can be trapped by PARPi. Cells expressing PARP1^WT^–Apex2–eGFP were transfected with a PAR sensor, a PBZ PAR-binding domain fused to mRuby2 (left). PARP1^WT^–Apex2–eGFP and PBZ–mRuby2 accumulate at the sites of UV micro-irradiation. Exposure to 100 nM talazoparib causes sustained accumulation of PARP1^WT^–Apex2–eGFP (middle) but abolishes PAR production (right). Data represent two independent experiments with similar results. DMSO, dimethylsulfoxide. **e**,**f**, PARP1 interactions that are enriched under PARP1-trapping conditions (as defined by the PSM ratio ((talazoparib + MMS) ÷ MMS) and MS scores). Scatter plots are shown for PARP1^WT^–eGFP (**e**) and PARP1^del.p.119K120S^–eGFP (**f**) RIME. **g**, PARP1 interactions that are enriched under PARP1-trapping conditions for PARP1^WT^–Apex2–eGFP proximity labelling. RIME and proximity labelling were performed in three independent experiments. **h**, A graph plotting the PSM against MS score for PARP1^WT^–Apex2–eGFP proximity labelling interactions shows that p97 is among the most abundant proteins identified in the PARP1^WT^–Apex2–eGFP proximity labelling. Source data

**Extended Data Fig. 1:**
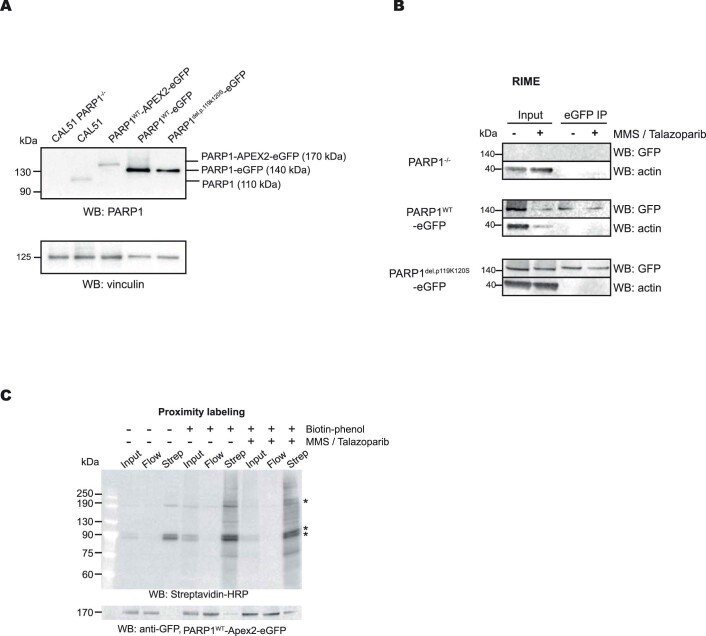
Proteomic profiling of PARP1 transgene-expressing CAL51 cells. **a**. Western blot showing the expression of PARP1 transgenes, detected by an PARP1 antibody. Data shown represent 2 biological replicas. **b**. A Western blot analysis of the purified PARP1-associated proteins as described in the RIME experiment in Fig. 1a. Data shown represent 3 biological replicas. **c**. Western blot analysis of the purified biotinylated proteins isolated in the PARP1WT-Apex2-eGFP proximity labelling experiment. Immunoblotting using Streptavadin-HRP is shown in the top panel, whilst anti-GFP immunoblotting is shown in the bottom panel. Endogenously biotinylated proteins are indicated as*. Data shown represent 3 biological replicas. Source data

To understand the nature of the trapped PARP1 complex, we used two orthogonal systems to generate mass spectrometry-based PARP1 protein–protein interactomes from cells with either trapped or non-trapped PARP1: (1) rapid immunoprecipitation mass spectrometry of endogenous proteins (RIME)^12^ and (2) in vivo Apex2 peroxidase-mediated labelling of proximal proteins^13^. We used a PARPi-resistant PARP1-defective cell line^3^, CAL51 *PARP1*^–/–^, into which we introduced either wild-type *PARP1* (*PARP1*^WT^) or a trapping-deficient *PARP1*^del.p.119K120S^ transgene^3^ (Fig. 1a) fused to enhanced green fluorescent protein (eGFP) for RIME or Apex2–eGFP for proximity labelling. We established single-cell clones that expressed the desired PARP1 fusion proteins (Extended Data Fig. 1a). Validating these transgenes, we found that expression of either PARP1^WT^–eGFP or PARP1^WT^–Apex2–GFP proteins re-established PARPi sensitivity in *PARP1*^–/–^ cells (Fig. 1b,c), whereas expression of the PARP1^del.p.119K120S^–eGFP did not. We also used a PAR-binding PBZ–mRuby2 probe and an ultraviolet light (UV) micro-irradiation assay to demonstrate that PARP1^WT^–Apex2–eGFP localized to DNA-damage sites where it generated PAR. In the presence of PARPi, PARP1 was retained at the site of damage (Fig. 1d).

As PARP1 translocates to chromatin following DNA damage, we first used RIME-based immunoprecipitation^12,14^ to identify proteins associated with trapped PARP1 (Fig. 1a). Cells expressing PARP1^WT^–eGFP or PARP1^del.p.119K120S^–eGFP were exposed to PARP1-trapping conditions (methyl methanesulfonate (MMS) + talazoparib), after which the protein interactions were stabilized by formaldehyde crosslinking. Talazoparib traps DNA-bound PARP1, whereas MMS was used to create DNA lesions. After trapping, the chromatin-bound proteins were isolated and the PARP1-associated complexes were immunoprecipitated using GFP-Trap beads. The proteins were then identified by mass spectrometry. We also included an analysis of the parental *PARP1*^–/–^ cells lacking eGFP to identify proteins that bind non-specifically to GFP-Trap beads (Extended Data Fig. 1b). The non-specific bead-binding proteins were removed from the list of identified proteins (see Methods). As a result, we identified 50 PARP1-associated proteins in cells expressing wild-type PARP1 (either in the presence or absence of PARPi; Supplementary Table 1) and 144 PARP1-associated proteins in cells expressing PARP1^del.p.119K120S^–eGFP (Supplementary Table 2). In both datasets, PARP1 was by far the most abundant protein identified by its mass spectrometry (MS) score (Fig. 1e,f). To prioritize proteins for further analysis, we used the MS score and the enrichment ratio of peptide spectrum matches (PSM) in the cells exposed to MMS + talazoparib compared with cells exposed to MMS alone. As expected, MMS + talazoparib increased the PARP1 PSM enrichment ratio in cells expressing PARP1^WT^–eGFP but not in those expressing PARP1^del.p.119K120S^–eGFP (2.5 versus 1.1, respectively). The trapping-defective PARP1^del.p.119K120S^–eGFP mutant seemed to interact with the cohesion complex subunit PDS5A, regardless of the presence of PARPi (Fig. 1f), suggesting that some interaction between mutant PARP1 and chromatin did exist independently from trapping. Both mutant and wild-type PARP1 also seemed to interact with chromatin-associated proteins (for example, CHD4), but when compared with PARP1^del.p.119K120S^–eGFP, PARP1^WT^–eGFP showed a relative enrichment of the small ubiquitin modifier proteins SUMO1 (PSM ratio of five in PARP1^WT^ versus one in PARP1^del.p.119K120S^) and SUMO2 (PSM ratio of four in PARP1^WT^ versus unidentified in PARP1^del.p.119K120S^; Fig. 1e).

**Extended Data Fig. 2:**
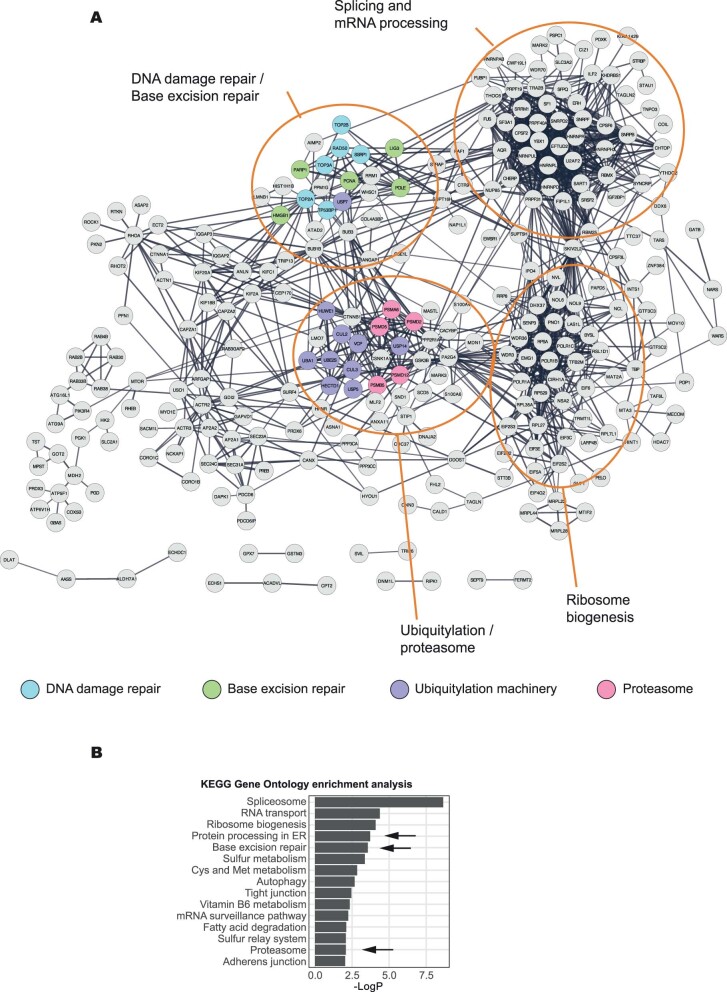
Bioinformatic analysis of trapped PARP1 proteomic data. **a**. STRING network diagram of proteins identified by PARP1 proximity labelling under PARPi trapping conditions (as described in Methods). The graph shows connected nodes identified with a high stringency threshold of 0.7 (non-connected proteins are excluded from this visualisation). The colour coding corresponds to the following functional annotation: DNA damage repair-associated proteins (blue), base excision repair (green), ubiquitylation machinery (purple) and proteasome (magenta). Clusters, enriched for certain biological processes are indicated (for example ‘Ubiquitylation/proteosome’). **b**. Summary of gene set ontology analysis of the networks presented in (F). KEGG terms enriched at p-value <0.01 are shown. Source data

As an orthogonal MS approach, we employed Apex2-mediated proximity labelling. Western blotting confirmed PARP1^WT^–Apex2–eGFP biotinylation in the presence of biotin-phenol (Extended Data Fig. 1c), which was further increased when PARP1 labelling was conducted under trapping conditions (Extended Data Fig. 1c). Biotinylated proteins were purified under stringent conditions and analysed by mass spectrometry. A caveat to our work was our inability to generate a trapping-defective PARP1 fused to Apex2–eGFP; this prevented us from using this control in the proximity labelling. We used cells expressing PARP1^WT^–eGFP instead to filter out non-specific interactions with beads. As a result of this filtering, we identified a higher number of proteins—that is, 360—associated with PARP1 in our proximity labelling analysis than for RIME (either in the presence or absence of PARPi; Supplementary Table 3). A STRING network analysis, using a high-stringency cutoff (0.7) representing the trapped PARP1 interactome network (Extended Data Fig. 2a), was enriched in proteins associated with PARP1-mediated base-excision repair (PCNA, HMGB1, LIG3, PARP1 and POLE; *P* < 0.01; Extended Data Fig. 2a,b), giving us high confidence in the analysis. Gene-set ontology analysis also identified an enrichment in proteins involved in the spliceosome and ribosome biogenesis (Supplementary Table 4). We also identified a number of well-characterized PARylation targets (for example, PCNA, NCL, FUS and ILF3; refs. ^15,16^), strengthening the idea that we identified bona fide PARP1-proximal proteins. Notably, ‘protein processing in endoplasmic reticulum’ (*P* < 10^−3^) and ‘proteasome’ (*P* < 0.01) seemed to be enriched in the gene-set ontology analysis, observations we focus on later in this manuscript. The MS and PSM scores showed a positive correlation and identified that PARP1, p97, UBA1 and TOP2A were among the most abundant proteins (Fig. 1g,h). Proteins that showed high enrichment ratios in PARP1-trapping conditions—for example, USP7—were generally identified with a low MS score pointing to a low abundance. As a trapping-deficient mutant was not available to perform a comparison similar to the RIME analysis, for the Apex2 analysis, we prioritized the high MS score over the high PSM ratio in our further considerations as it would represent higher stoichiometric interactions at DNA-damage sites. Among the most abundant labelled proteins were ubiquitin-like modifier-activating enzyme 1 (UBA1), which has been previously implicated in ubiquitylation events at the sites of DNA damage^17^, and the transitional endoplasmic reticulum ATPase p97, which acts as a central component of a ubiquitin-controlled process. The ATP-dependent unfoldase activity of p97 extracts proteins from chromatin before their proteasomal degradation or recycling^9–11,18–20^. Furthermore, p97, working with cofactors that often contain ubiquitin-binding domains, recognises client proteins via ubiquitylation events, mostly those involving ubiquitylation of the K48 and K6 residues^21,22^. In addition, p97 was identified in the PARP1^WT^, but not in the PARP1^del.p.119K120S^, RIME analysis, strengthening the idea that it may interact with trapped PARP1.

### Trapped PARP1 is sequentially SUMOylated and ubiquitylated

**Fig. 2:**
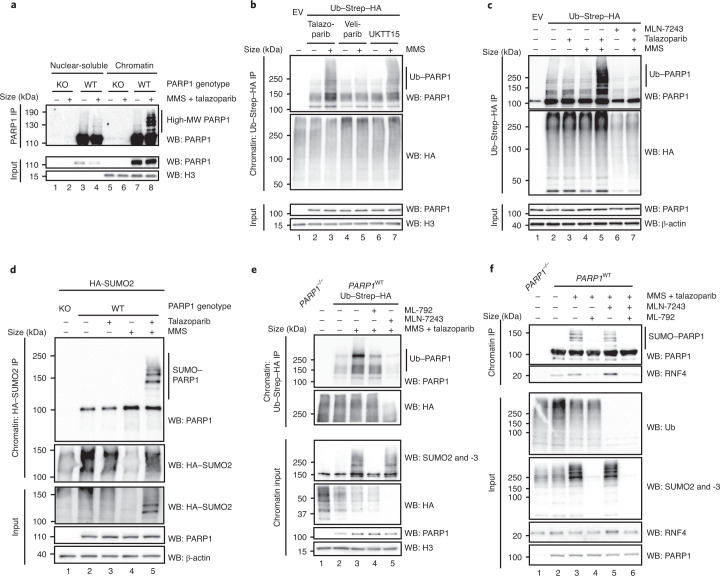
Trapped PARP1 is SUMOylated and ubiquitylated. **a**, PARP1-trapping conditions elicit high-molecular-weight forms of PARP1 in the chromatin fraction. *PARP1*^−/−^ (KO) and *PARP1*^WT^ HEK293 cells were exposed to PARP1 trapping (MMS + talazoparib) and fractionated into nuclear-soluble and chromatin-bound fractions. High-molecular-weight (MW) forms of PARP1 are more prevalent in the chromatin fraction after trapping (lane 8). **b**, PARP1 trapping leads to PARP1 ubiquitylation. HEK293 cells were transfected with an Ub–Strep–HA-expressing construct and exposed to 0.01% MMS, 100 nM talazoparib, 10 μM veliparib or 10 μM UKTT15. Chromatin fractions were prepared in denaturing conditions, ubiquitylated proteins were immunoprecipitated and the presence of PARP1 was detected. **c**, As in **b**, HEK293 cells were transfected with an Ub–Strep–HA-expressing construct and exposed to combinations of MMS, talazoparib or 5 μM MLN-7243. The presence of high-molecular-weight ubiquitin forms of PARP1 were reduced by MLN-7243 exposure (lane 7 versus lane 5). The input controls for these experiments are shown in Extended Data Fig. 3a. **b**,**c**, EV, empty vector. **d**, Trapped PARP1 is SUMOylated. HEK293 PARP1^WT^ and PARP1^−/−^ cells were transfected with a HA–SUMO2-expressing construct and subsequently treated with 0.01% MMS or 100 nM talazoparib. The HA–SUMO2-modified proteins were purified from the chromatin fraction and PARP1 was detected via immunoblotting. High-exposure blots of PARP1 SUMOylation are shown in Extended Data Fig. 3b. **e**, SUMOylation and ubiquitylation inhibitors prevent trapped PARP1 modification. Similarly to **c**, the ubiquitylated pool of proteins was immunoprecipitated from the chromatin fraction of cells exposed to MLN-7243 (5 μM) or ML-792 (1 μM) and the presence of high-molecular-weight PARP1 isoforms was identified by immunoblotting. **f**, PARP1 is modified and interacts with RNF4 in a SUMO-dependent manner. *PARP1*^WT^ and *PARP1*^–/–^ HEK293 cells were exposed to trapping conditions in the presence of either 5 μM MLN-7243 or 1 μM ML-792 and PARP1 was immunoprecipitated under native conditions using PARP1-Trap beads. Western blotting for PARP1 revealed that modified PARP1 isoforms were abrogated by exposure to ML-792 but not MLN-7243. Abrogation of SUMOylation prevented the association between PARP1 and RNF4, whereas inhibition of ubiquitylation stabilized the interaction. Data represent two biological replicates. IP, immunoprecipitation; and WB, western blot. Source data

Our RIME analysis suggested that trapped PARP1 was associated with SUMO1 and SUMO2, whereas our proximity labelling analysis identified ubiquitylation and p97 to be associated with trapped PARP1. This raised the hypothesis that trapped PARP1 is modified by SUMOylation and ubiquitylation. This hypothesis was consistent with the observation that in cells cultured in MMS + PARPi, chromatin-associated PARP1 was present as multiple high-molecular-weight forms that could conceivably represent SUMOylated and/or ubiquitylated PARP1, forms that are absent in the nuclear-soluble fraction (Fig. 2a).

**Extended Data Fig. 3:**
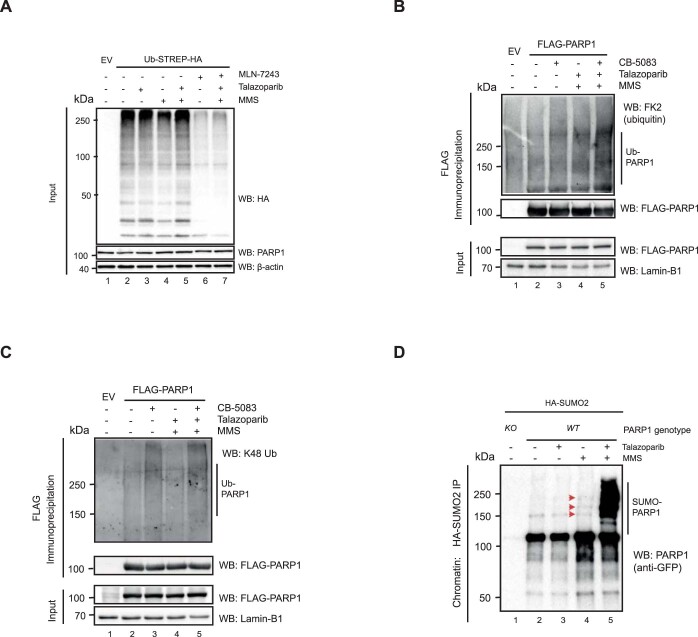
Trapped PARP1 is SUMOylated and ubiquitinated. **a**. Input controls for Fig. 2c, showing the efficacy of MLN-7243 to inhibit ubiquitylation. Data shown represent 3 biological replicas. **b**. Reciprocal denaturing IP over PARP1-FLAG showed accumulation of trapped PARP1 ubiquitination in HEK293s. HEK293 cells were transfected with PARP1-FLAG-expressing construct for 24 hours then treated with 100 nM talazoparib/0.01% MMS and/or 10 µM CB-5083. Cells were lysed, chromatin was digested and then incubated with anti-FLAG beads. 4% of sample was harvested for input pre-incubation. Data shown represent 2 biological replicas. **c**. As in (C), but the immunoprecipitated proteins were analysed with an anti-K48 Ub chains recognising antibody. This experiment has been performed once. **d**. High exposure blot of PARP1 SUMOylation from Fig. 2d, red arrows show SUMOylated PARP1 in MMS treated samples. Data shown represent 2 biological replicas. Source data

We first assessed whether ubiquitylated PARP1 was present in cells exposed to PARPi with different trapping properties. We used the following PARP inhibitors: (1) the potent PARP1 trapper talazoparib; (2) veliparib, a clinical PARPi that effectively inhibits PARP1 catalytic activity but has minimal trapping properties; and (3) a recently described structural derivative of veliparib, UKTT15, that is able to elicit PARP1 trapping^4^. Cells were exposed to MMS + PARPi, after which the ubiquitylated pool of proteins was isolated from the chromatin fraction via ubiquitin–streptavidin–haemagglutinin (Ub–Strep–HA) isolation. In this fraction, high-molecular-weight isoforms of PARP1 were more prevalent in the talazoparib- or UKTT15-exposed cells compared with veliparib-exposed cells (Fig. 2b), suggesting that PARP1 ubiquitylation was enhanced by PARP1 trapping. We repeated the Ub–Strep–HA pulldown experiment in the presence of the E1 ubiquitin activating enzyme inhibitor MLN-7243 (also known as TAK243). Western blotting with an antibody to PARP1 revealed that trapping conditions led to the formation of high-molecular-weight PARP1 isoforms; these were almost completely abolished when the cells were exposed to MLN-7243, consistent with these high-molecular-weight isoforms representing ubiquitylated PARP1 (Fig. 2c and Extended Data Fig. 3a). The poly-ubiquitylation of PARP1 was also observed in reciprocal denaturing immunoprecipitation experiments, where PARP1 was immunoprecipitated from HEK293 cells transfected with a FLAG-tagged *PARP1* complementary DNA-expression construct (Extended Data Fig. 3b). We also identified poly-ubiquitin chains on PARP1 that were linked by K48 linkage (Extended Data Fig. 3c).

The presence of SUMO1 and SUMO2 in our trapped PARP1 interactome suggested that PARP1 may also be modified by SUMOylation in addition to ubiquitylated PARP1. We expressed HA epitope-tagged SUMO2 and isolated the SUMOylated pool of proteins under denaturing conditions from the chromatin fraction. Trapped PARP1 was clearly modified by SUMOylation (Fig. 2d). We also found that when cells were exposed to MMS alone (to induce DNA damage and activate PARP1) in the absence of PARPi, there was a depletion in the total pool of SUMO2 and a minimal level of PARP1 SUMOylation, as previously observed^23^ (Fig. 2d and Extended Data Fig. 3d). However, this did not occur to the same extent as seen under PARP1-trapping conditions. This suggested that PARP1 is SUMOylated when it becomes trapped. Interestingly, when cells cultured in PARP1-trapping conditions were incubated in the presence of a SUMOylation inhibitor (ML-792, which inhibits SUMO-activating enzyme), the levels of high-molecular-weight forms of ubiquitylated PARP1 decreased (Fig. 2e), suggesting PARP1 ubiquitylation following trapping could require previous PARP1 SUMOylation. Conversely, a ubiquitylation inhibitor had no effect on PARP1 SUMOylation (Fig. 2f), suggesting that the SUMOylation of trapped PARP1 is required for its ubiquitylation but that the ubiquitylation of trapped PARP1 is not a prerequisite for PARP1 SUMOylation.

### Trapped PARP1 is sequentially modified by PIAS4 and RNF4

The pattern of SUMOylation and ubiquitylation of trapped PARP1 suggested the concert action of a SUMO E3 ligase and a SUMO-targeted ubiquitin ligase (STUbL). We assessed whether PIAS4 (a SUMO E3 ligase) and RNF4 (a STUbL) were responsible. PIAS4 has been previously implicated as a SUMO E3 ligase for PARP1 in its non-trapped state^24^ and RNF4 has previously been implicated in modulating the transcriptional activity of PARP1 (ref. ^25^) as well as being involved in the repair of topoisomerase cleavage complexes, which also represent a ‘trapped’ nucleoprotein complex^26^. Chromatin co-immunoprecipitation of trapped PARP1 showed an increased interaction with RNF4 (Fig. 2f), consistent with our hypothesis. This PARP1–RNF4 interaction was reduced following inhibition of SUMOylation and stabilized in cells exposed to a ubiquitylation inhibitor, indicative of a ligase–substrate interaction (Fig. 2f).

**Fig. 3:**
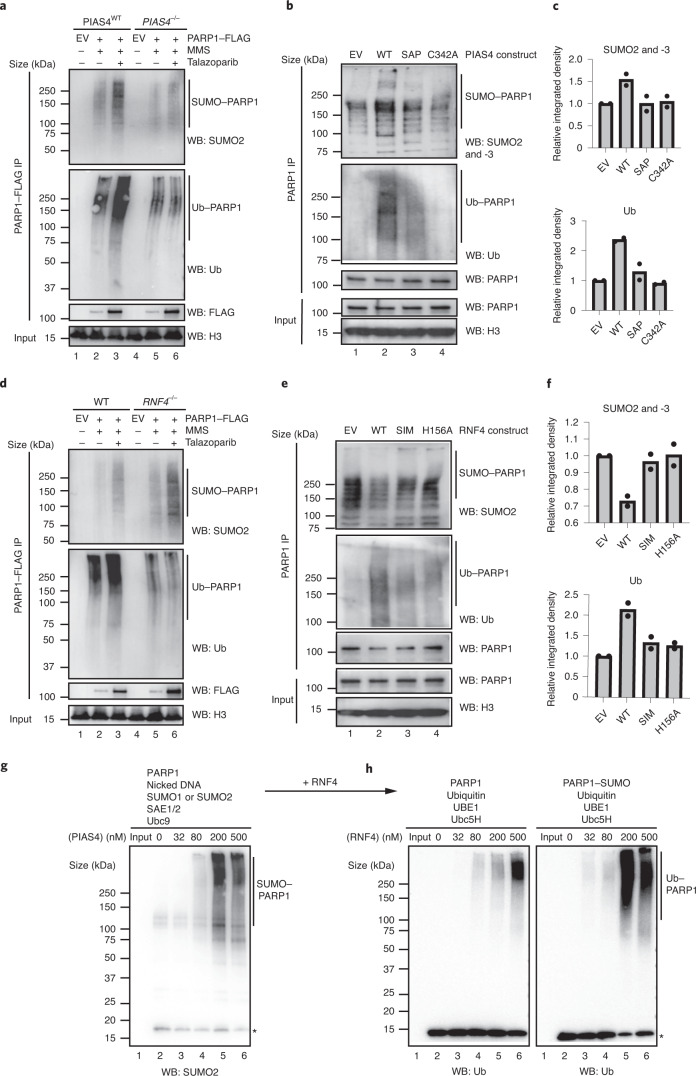
Trapped PARP1 is modified in a PIAS4- and RNF4-dependent manner. **a**, PARP1 is SUMOylated in a PIAS4-dependent manner in vivo. Wild-type and *PIAS4*^–/–^ HCT116 cells were transfected with FLAG–PARP1-expressing plasmid, exposed to trapping and the chromatin-bound PARP1 was investigated for SUMOylation and ubiquitylation. The levels of SUMO1 (Extended Data Fig. 4a), SUMO2 and ubiquitin were reduced in the *PIAS4*^–/–^ cells (see Extended Data Fig. 4b,c for the total ubiquitin input and quantification of the blots, respectively). **b**, *PIAS4*^–/–^ HCT116 cells were transfected with different PIAS4-expressing constructs for 48 h, followed by 30 min talazoparib (10 µM) treatment in the presence of 0.01% MMS and PARP1 immunoprecipitation. **c**, Abundance of SUMO2 and -3 (top)- and ubiquitin (bottom)-modified PARP1 in **b**. **b**,**c**, Data represent two biological replicates. SAP, PIAS4 with deleted SAP domain; and C342A, catalytic dead PIAS4. **d**, Similarly to **a**, trapped PARP1 was purified from the chromatin fraction of wild-type and *RNF4*^–/–^ MCF7 cells. The PARP1 ubiquitylation levels were reduced in the *RNF4*^–/–^ cells, whereas SUMO1- (Extended Data Fig. 4e) and SUMO2-ylation were increased (see Extended Data Fig. 4f,g for the total ubiquitin input and quantification of the blots). **e**, *RNF4*^–/–^ MCF7 cells were transfected with different RNF4-expressing plasmids for 48 h and processed as in **b**. **f**, Abundance of SUMO2 and -3 (top)- and ubiquitin (bottom)-modified PARP1 in **e**. **e**,**f**, Data represent two biological replicates. SIM, RNF4 with deleted SUMO-interacting motif; and H156A, catalytic dead RNF4. **g**, PIAS4 mediates PARP1 SUMOylation in vitro. Recombinant PARP1 was incubated with nicked DNA, SUMO1 or SUMO2, SAE1 and -2, Ubc9 and an increasing concentration of PIAS4. PIAS4 led to a concentration-dependent increase of SUMOylation (Extended Data Fig. 5c). *Free SUMO2. **h**, RNF4 mediates PARP1 ubiquitylation in a SUMO-dependent manner in vitro. PARP1 SUMOylation reactions were supplemented with ubiquitin, UBE1, Ubc5H and an increasing concentration of RNF4. SUMOylated PARP1 was a better substrate for ubiquitylation. *Free ubiquitin. **a**,**d**,**g**,**h**, Data shown represent two independent experiments with similar results. EV, empty vector; IP, immunoprecipitation; WB, western blot; and WT, wild type. Source data

**Extended Data Fig. 4:**
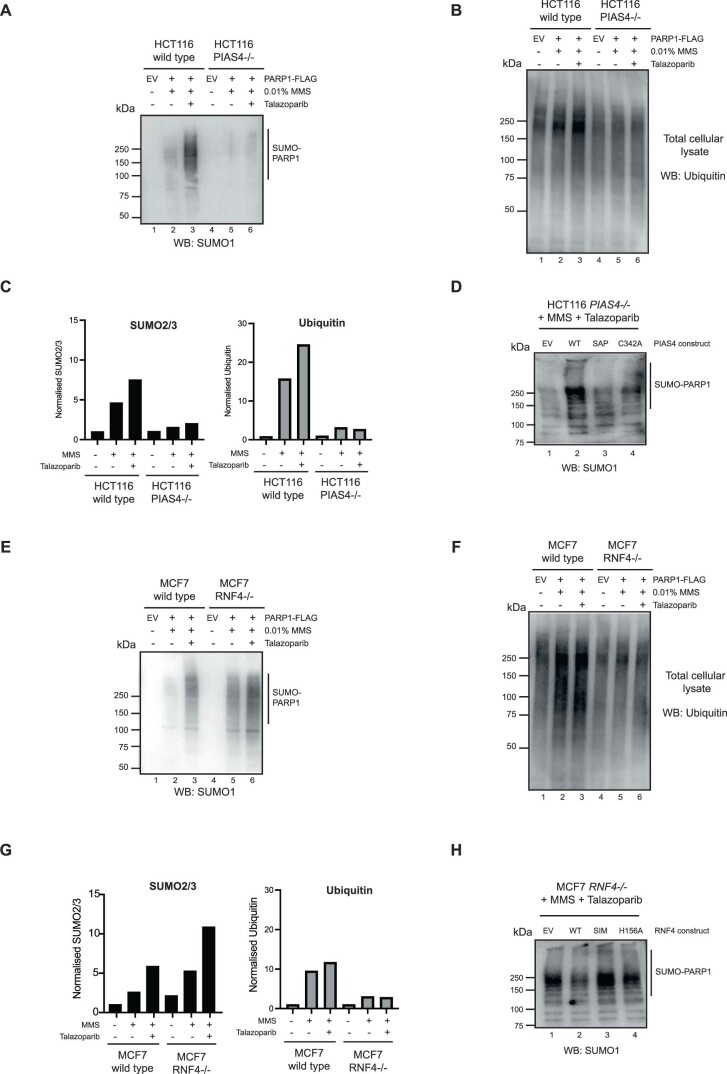
Trapped PARP1 is modified in a PIAS4-dependent manner. **a**. PARP1 SUMOylation by SUMO1 was detected as described in Fig. 3a. Data shown represent 2 biological replicas. **b**. Western blotting for total ubiquitin input for Fig. 3a. Data shown represent 2 biological replicas. **c**. A quantification of the SUMO2/3ylated and ubiquitylated PARP1 isoforms in the gels in Fig. 3a. **d**. PARP1 SUMOylation by SUMO1 detected as described in Fig. 3b. Data shown represent 2 biological replicas. **e**. PARP1 SUMOylation by SUMO1 was detected as described in Fig. 3d. n = 1 biological replicas. **f**. Western blotting for total ubiquitin input for Fig. 3d. Data shown represent 2 biological replicas. **g**. A quantification of the SUMO2/3ylated and ubiquitylated PARP1 isoforms in the gels in Fig. 3d. **h**. PARP1 SUMOylation by SUMO1 detected as described in Fig. 3e. Data shown represent 2 biological replicas. Source data

**Extended Data Fig. 5:**
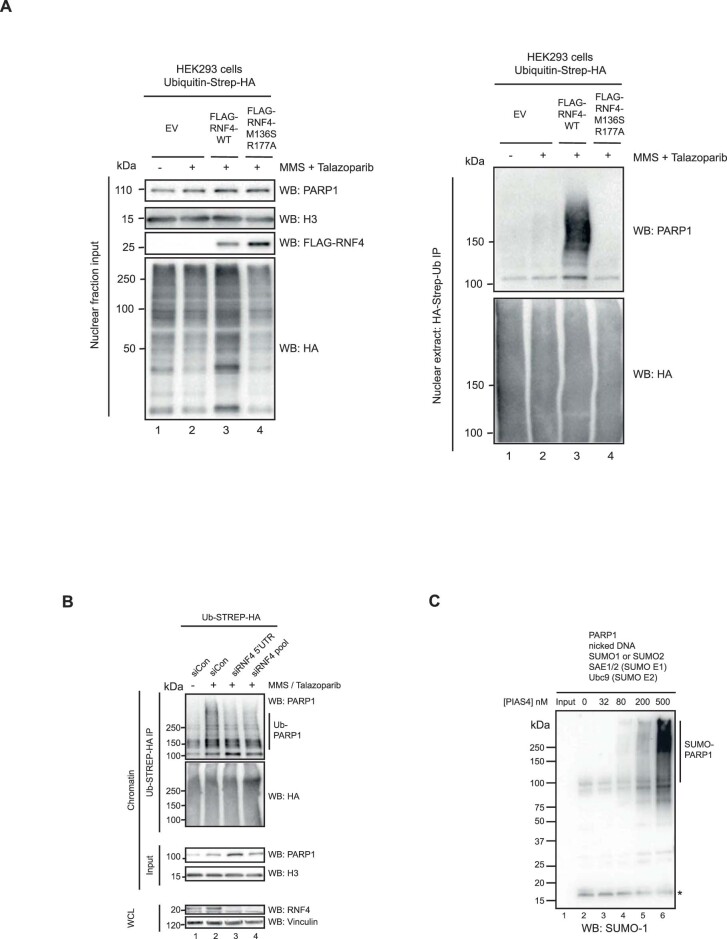
Trapped PARP1 is modified in a RNF4-dependent manner. **a**. Overexpression of RNF4-WT increased PARP1 ubiquitination under trapping conditions. HEK293 cells were transfected with Ubiquitin-Strep-HA in combination with either FLAG-RNF4-WT or M136S/R177A mutant (E2 binding mutant, dominant negative) expressing constructs. After treatment with MMS + Talazoparib, the cells were fractionated and ubiquitylated proteins were purified from the chromatin-bound fraction via Streptactin beads. Data shown represent 2 biological replicas. **b**. RNF4 depletion prevents PARP1 ubiquitination under PARP trapping conditions. Denaturing IP of UB-HA-STREP-expressing HEK293 cells, similar to Fig. 2b. Cells were depleted of RNF4 with either a 5’UTR sequence or Dharmarcon SMARTpool and were treated with 100 nM Talazoparib and 0.01% MMS. Pulldown was conducted with Streptactin beads. Data shown represent 2 biological replicas. **c**. *In vitro* SUMOylation assay as described in Fig. 3g. The reactions were incubated in the presence of SUMO1, which was subsequently detected by anti-SUMO1 antibody. The asterisk indicates the free SUMO1. Data shown represent 2 biological replicas. Source data

To delineate the relationship between SUMOylation and ubiquitylation of trapped PARP1 and a possible role for the SUMO E3 ligase PIAS4 in this process, we used *PIAS4*^–/–^ HCT116 and *RNF4*^–/–^ MCF7 cell lines^26^. Both cell lines were transfected with a FLAG–PARP1-expressing cDNA construct. After culturing the cells in MMS + PARPi, we immunoprecipitated PARP1 from the chromatin fraction. Western blotting with an antibody to SUMO2 and -3 revealed that PIAS4 is necessary for efficient SUMOylation and ubiquitylation of trapped PARP1 (Fig. 3a and Extended Data Fig. 4a–c). Re-expression of wild-type PIAS4 in *PIAS4*^–/–^ cells reversed these effects, but this was not achieved when we expressed a DNA binding-deficient form of PIAS4 (SAP domain deleted) or the catalytically inactive p.C342A PIAS4 mutant^27^ (Fig. 3b,c and Extended Data Fig. 4d). Interestingly, although PARP1 ubiquitylation was decreased in *RNF4*^–/–^ cells (confirming that RNF4 activity is responsible for this modification), PARP1 SUMOylation was increased (Fig. 3d and Extended Data Fig. 4e–g). Re-expression of wild-type RNF4 in the *RNF4*^–/–^ cells also reversed these effects, but this was not the case in cells expressing the SUMO-interacting motif-deleted^28^ or catalytically inactive p.H156A mutant forms of RNF4 (Fig. 3e,f and Extended Data Fig. 4h). We also observed strong RNF4-dependent PARP1 ubiquitylation by overexpressing wild-type RNF4 in cells cultured in MMS + PARPi (Extended Data Fig. 5a), an effect that was not observed when we expressed a dominant-negative E2 binding-mutant form of RNF4 (p.M136S/R177A). Using the *RNF4*^–/–^ cells and dominant-negative mutants of RNF4, we found that RNF4 was responsible for 80–95% of the ubiquitylation of trapped PARP1. We also found that silencing of the *RNF4* gene reduced ubiquitylation of trapped PARP1 (Extended Data Fig. 5b). Together, these data established RNF4 as a STUbL E3 ligase for trapped PARP1. Although PIAS4 and RNF4 accounted for the majority of SUMOylation and ubiquitylation of trapped PARP1, both *PIAS4*^–/–^ and *RNF4*^–/–^ cells exhibited some residual PARP1 SUMOylation and ubiquitylation, suggesting that other ligases might also contribute to the modification of trapped PARP1.

Finally, we tested the interdependency of PARP1 SUMOylation and ubiquitylation events using in vitro SUMOylation and ubiquitylation reactions. Incubation of recombinant PARP1 in the presence of a synthetic nicked DNA substrate, SUMO1 or SUMO2, SAE1 (SUMO E1), Ubc9 (SUMO E2) and an increasing concentration of PIAS4 drove a concentration-dependent SUMOylation of PARP1 (Fig. 3g and Extended Data Fig. 5c). The addition of ubiquitin, UBE1 (Ub E1), Ubc5H (Ub E2) and an increasing concentration of RNF4 to this reaction led to efficient PARP1 ubiquitylation (Fig. 3h). In contrast, RNF4 displayed much lower PARP1 ubiquitylating activity in the absence of SUMOylation (Fig. 3h). Collectively, these data suggested a stepwise process, where following trapping, PARP1 is initially SUMOylated by PIAS4, followed by STUbL RNF4-driven ubiquitylation.

### p97 interacts with modified trapped PARP1

Although the above experiments suggested a link between the trapping of PARP1 by PARPi and subsequent PARP1 SUMOylation and ubiquitylation, the functional significance of these post-translational modifications remained to be determined. Our mass spectrometry analysis suggested that under PARP1-trapping conditions there was an enhanced interaction between PARP1 and p97, an ATPase involved in the removal of ubiquitylated substrate proteins from chromatin. We therefore hypothesized that the SUMOylation and ubiquitylation of trapped PARP1 serve as a necessary prelude to the recruitment of p97 ATPase and the removal of trapped PARP1 from chromatin.

**Fig. 4:**
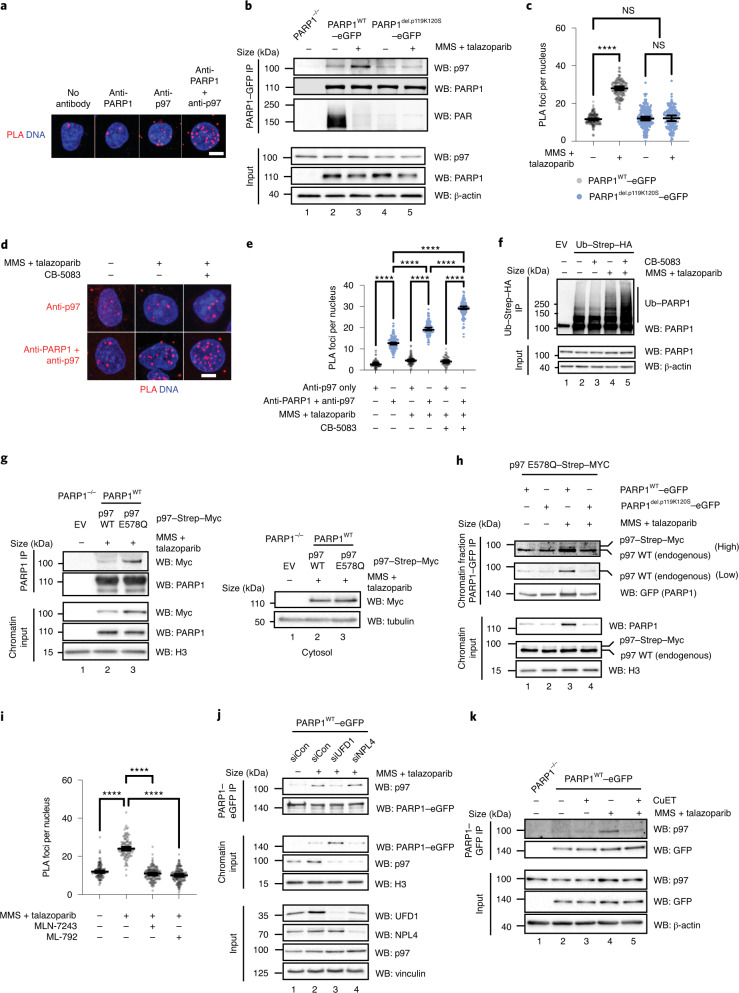
PARP1 interacts with p97 in a trapping-dependent manner. **a**, Images of a PLA for endogenous PARP1 and p97 in CAL51 cells. **b**, The PARP1–p97 interaction is increased following DNA damage. CAL51 cells expressing PARP1^WT^–eGFP or PARP1^del.pK119S120^–eGFP were exposed to trapping conditions and PARP1–GFP was immunoprecipitated under native conditions. Data represent two biological replicates. **c**, PARP1–p97 PLA (anti-PARP1 and anti-p97) in CAL51 cells expressing either PARP1^WT^–eGFP or PARP1^del.pK119S120^–eGFP. The geometric mean and 95% confidence interval (CI) are shown; *n* = 2,016 cells from three independent experiments. **d**, PARP1–p97 PLA in CAL51 cells under trapping. PLA with p97 antibody alone (top) or p97 + PARP1 antibody (bottom). **a**,**d**, Scale bars, 5 μm. Data represent three biological replicates. **e**, Number of PLA foci in **d**. The geometric mean and 95% CI are shown; *n* = 2,035 cells from three independent experiments. **f**, Inhibition of p97 increases the presence of ubiquitylated PARP1. HEK293 cells expressing Ub–Strep–HA were cultured in PARP1-trapping conditions in the presence or absence of 10 μM CB-5083 and the ubiquitylated proteins were immunoprecipitated under denaturing conditions (see Extended Data Fig. 6c for the input controls). Data represent three biological replicates. **g**, HEK293 cells expressing either wild-type p97–Myc or p97 p.E578Q–Myc were transfected with a FLAG–PARP1 construct, exposed to trapping conditions and PARP1 immunoprecipitated from the chromatin fraction. Data represent two biological replicates. **h**, CAL51 cells expressing PARP1^WT^–eGFP or PARP1^del.p.119K120S^–eGFP were transfected with p97 E578Q–Strep–Myc for 18 h, exposed to trapping conditions and then fractionated. The chromatin PARP1–eGFP immunoprecipitate was probed by antibody that detected both endogenous and ectopically expressed p97. Data represent two biological replicates. **i**, PARP1–p97 co-localization is reduced by ubiquitylation (5 μM MLN-7243) or SUMOylation (1 μM ML-792) inhibitors. Number of PARP1–p97 PLA (anti-PARP1 and anti-p97) foci. The geometric mean and 95% CI are shown; *n* = 1,316 cells from three independent experiments. **c**,**e**,**i**, ****P < 0.0001; NS, not significant; ordinary one-way analysis of variance (ANOVA). **j**, The p97 adaptor UFD1 mediates the interaction between p97 and trapped PARP1. Chromatin-bound co-immunoprecipitation. Data represent three biological replicates; siCon, control short interfering RNA (siRNA); siUFD1, siRNA to *UFD1*; and siNPL4, siRNA to *NPL4*. **k**, As per **j**, the PARP1–p97 interaction is disrupted by the p97 sequestration agent, CuET. Data represent two biological replicates. EV, empty vector; IP, immunoprecipitation; WB, western blot; and WT, wild type. Source data

**Extended Data Fig. 6:**
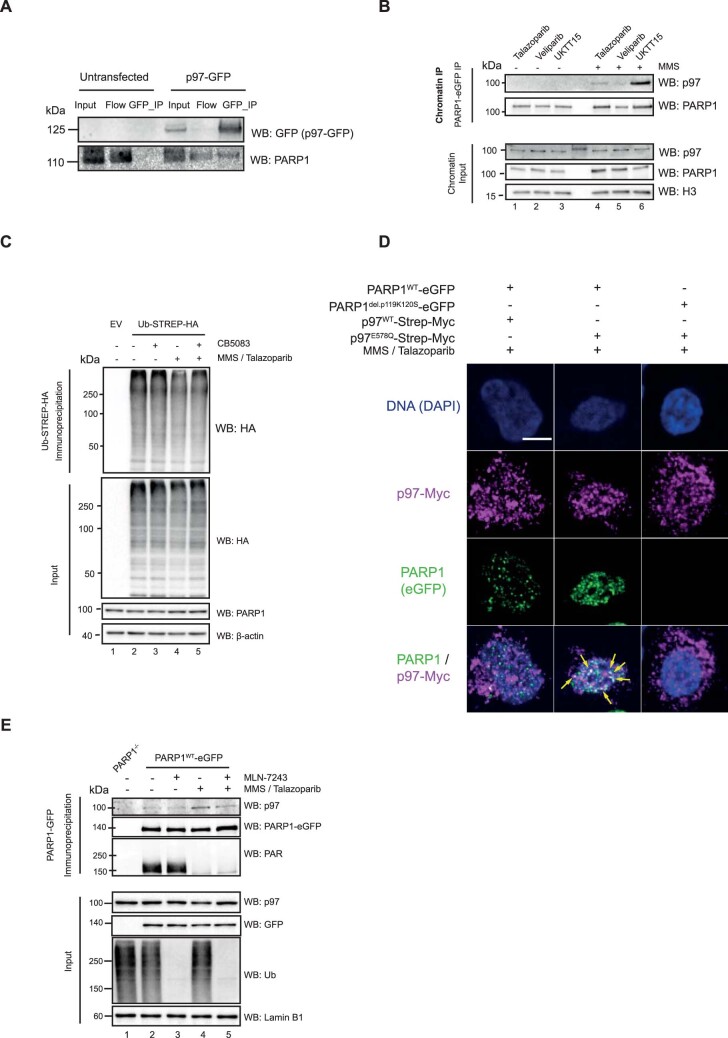
Trapped PARP1 interacts with p97. **a**. Western blot analysis of Co-IP confirms PARP1–p97 interaction. CAL51 cells were transiently transfected with p97-WT-GFP-expressing construct. Subsequently, GFP was immunoprecipitated in native conditions and the presence of PARP1 investigated by Western blotting. Data shown represent 2 biological replicas. **b**. PARP1 interacts with p97 in a trapping-dependant manner. Cells were treated with 0.01% MMS in the presence of 100 nM talazoparib, 10 µM veliparib or 10 µM UKTT15. PARP1 associated proteins were immunoprecipitated and the presence of p97 was investigated by immunoblotting. Data shown represent 2 biological replicas. **c**. Western blots for denaturing IP experiment shown in Fig. 4f. Data shown represent 3 biological replicas. **d**. p97 E578Q mutant colocalises with PARP1 under trapping conditions. CAL51 PARP1WT-eGFP and PARP1del.p.119K120S-eGFP cells were transfected with p97-WT-Strep-MYC or p97 E578Q-Strep-MYC constructs and then subsequently exposed to MMS + talazoparib to induce PARP1 trapping. Cell were then pre-extracted and fixed, and stained for trapped PARP1 and MYC (as described in^34^). The p97 E578Q-mutant colocalised with the trapped PARP1 signal in CAL51 PARP1WT-eGFP cells (yellow arrows) whereas PARP1del.p.119K120S-eGFP were unable to form trapped PARP1 foci. Scale bar = 5 µm. Data shown represent 2 biological replicas. **e**. Ubiquitin is required for the PARP1/p97 interaction in trapping conditions. Western blots of PARP1 co-immunoprecipitates from CAL51 PARP1^WT^-eGFP-expressing cells. Trapping increases the PARP1/p97 interaction (lane 4), an effect reversed by MLN-7243 (5 μM). Data shown represent 3 biological replicas. Source data

We confirmed the interaction between p97 and PARP1 using both proximity ligation assays (PLAs; Fig. 4a) and co-immunoprecipitation experiments (Extended Data Fig. 6a). We then verified that the PARP1–p97 interaction was enhanced in a trapping-dependant manner in PARP1 wild-type cells but not in cells expressing a DNA binding-deficient PARP1 mutant (Fig. 4b,c). UKTT15, but not veliparib, also led to an increase in the PARP1–p97 interaction, consistent with PARP1 trapping being important for this interaction, as opposed to catalytic inhibition of PARP1 (Extended Data Fig. 6b).

CB-5083 is a small molecule that inhibits p97 ATPase activity and induces a p97 substrate-trapping effect^29^; we found that CB-5083 caused an increase in PARP1–p97 interactions (Fig. 4d,e), suggesting that PARP1 could be a p97 substrate. Blocking p97 catalytic activity leads to the accumulation of ubiquitylated isoforms of its substrates^30,31^, which was also the case for trapped PARP1 (Fig. 4f). This was also observed using reciprocal immunoprecipitation of PARP1 under denaturing conditions (Fig. 4f and Extended Data Fig. 3b,c). We reproduced the substrate-trapping effect of p97 inhibition by expressing a dominant-negative ATPase-deficient p97 mutant, p.E578Q^18,32,33^ (Fig. 4g), consistent with trapped PARP1 being a p97 substrate. Furthermore, we demonstrated that the PARP1–p97 interaction was enhanced by expressing the p97 p.E578Q mutant in *PARP1*^–/–^ cells reconstituted with wild-type PARP1 but not the trapping-defective PARP1^del.pK119S120^ (Fig. 4h). This conclusion was further supported by co-localization immunofluorescence experiments, where p97 p.E578Q and trapped PARP1 foci^34^ were found to substantially overlap (Extended Data Fig. 6d).

Ubiquitylation is a mediator of p97 interactions^6,7^. When cells were exposed to ubiquitylation (MLN-7243) or SUMOylation (ML-792) inhibitors (which decreased trapped PARP1 ubiquitylation; Fig. 2e), the PARP1–p97 interaction was reduced (Fig. 4i and Extended Data Fig. 6e). p97 recognises and processes its ubiquitylated substrates using the NPL4–UFD1 complex, which mostly serves as a ubiquitin-binding receptor due to ubiquitin-binding domains in both NPL4 and UFD1 (refs. ^35,36^). When UFD1 was depleted, the interaction between trapped PARP1 and p97 was reduced (Fig. 4j). This was not the case when NPL4 was depleted, although, as expected, depletion of either subunit reduced the overall p97 recruitment to chromatin (Fig. 4j). Furthermore, only depletion of UFD1 led to a profound accumulation of trapped PARP1 (Fig. 4j). This suggested that the processing of trapped PARP1 is UFD1- but not NPL4-dependent. Although canonically, UFD1 is thought to function as an obligate heterodimer with NPL4, these observations seem consistent with previous work suggesting that NPL4 and UFD1 can recognise substrates independently of each other^7,8,10^. We also evaluated the effect of CuET, a metabolite of the approved alcohol-abuse drug disulfiram, which segregates p97 from chromatin into inactive agglomerates by disrupting NPL4 zinc finger motifs^37,38^ and thus serves as a tool that inactivates the entire p97 pool. Because of its ability to inactivate the p97 pool by forming agglomerates, CuET has a distinct mechanism of action compared with CB-5083 and also *NPL4*- or *UFD1*-gene silencing. We found that the PARP1–p97 interaction was almost completely abrogated by CuET exposure (Fig. 4k). Together, these observations suggest that the p97 system and its ubiquitin-binding cofactor UFD1 (p97–UFD1) recognise and physically interact with trapped PARP1.

### Trapped PARP1 is modulated by p97 activity

**Fig. 5:**
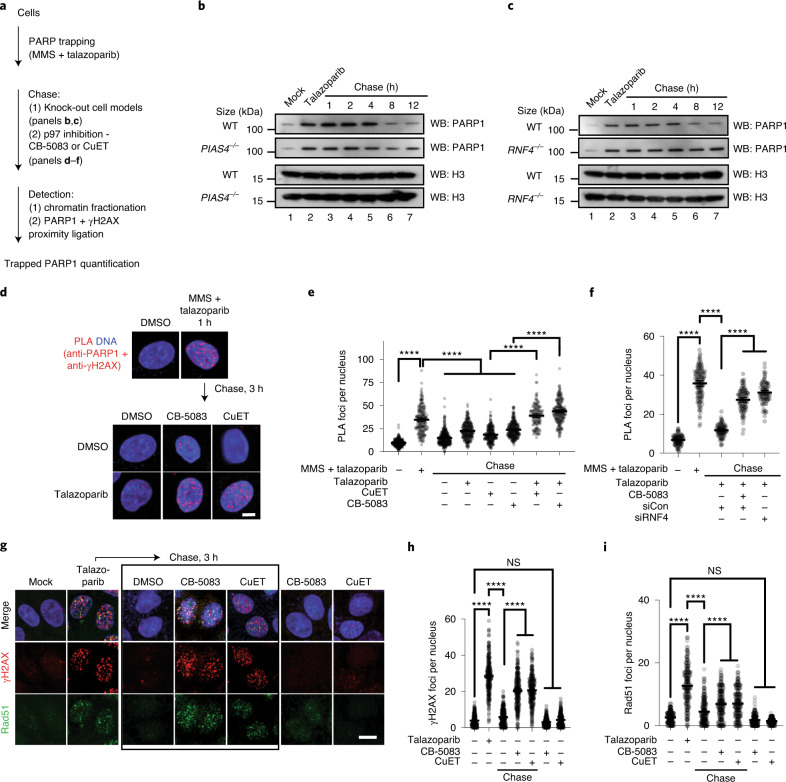
PARP trapping is modulated by the PIAS4–RNF4–P97 axis. **a**, Schematic of the trap–chase experiment. **b**, Trapped PARP1 is processed in a PIAS4-dependent manner. Trap–chase experiment in wild-type and *PIAS4*^–/–^ HCT116 cells. After PARP1 trapping, cells were chased in talazoparib-containing media. Samples were collected at the indicated time points for chromatin fractionation and western blotting. **c**, Trapped PARP1 is processed in a RNF4-dependent manner. Trap–chase experiment in wild-type and *RNF4*^–/–^ MCF7 cells as in **b**. **b**,**c**, Data represent two biological replicates. WB, western blot; and WT, wild type. **d**, Representative confocal microscopy images from a PARP1–γH2AX PLA trap–chase experiment. **e**, PARP1–γH2AX PLA foci persist in cells chased in PARPi plus p97 inhibitors. Number of PARP1–γH2AX PLA (anti-PARP1 + anti-γH2AX) foci in the trap–chase experiment in **d**; *n* = 5,736 cells from three independent experiments. **f**, PARP1–γH2AX PLA foci persist in cells with RNF4 silencing. Number of PLA foci in *n* = 1,235 cells from three independent experiments; siCon, control siRNA; and siRNF4, siRNA to *RNF4*. **g**, PARPi-induced RAD51 and γH2AX foci persist in the presence of p97 inhibitors. Representative confocal microscopy images from a trap–chase experiment (trap, talazoparib overnight; chase, p97 inhibitor-containing media) are shown for each condition. The cells were stained for the presence of γH2AX and RAD51 foci. Black box represents cells that were chased after talazoparib treatment, in contrast to the last two columns, which were not pre-treated with talazoparib. **d**,**g**, Scale bars, 5 μm. DMSO, dimethylsulfoxide. **h**,**i**, Number of γH2AX (**h**) and RAD51 foci (**i**) from the experiment in **g**; *n* = 1,750 cells from three independent experiments. **e**,**f**,**h**,**i**, The geometric mean and 95% CI are shown. ****P < 0.0001; NS, not significant; ordinary one-way ANOVA. Source data

**Extended Data Fig. 7:**
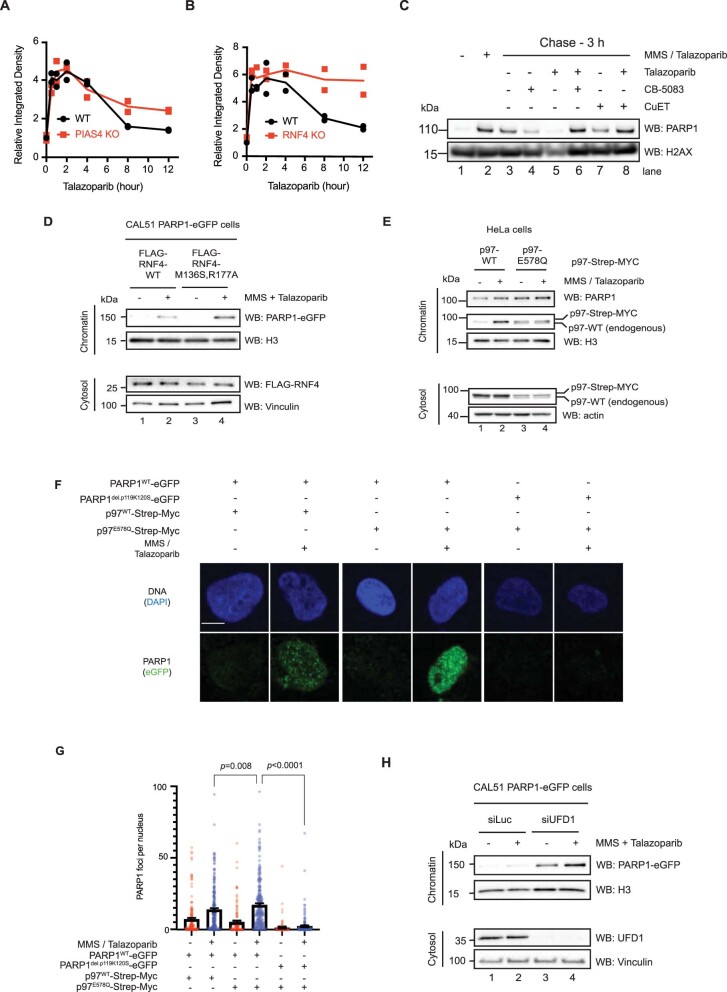
PARP1 trapping is modulated by PIAS4, RNF4 and p97. **a**. Quantification of the chromatin bound PARP1 in Fig. 5b; 2 biological replicas are displayed with individual points. **b**. Quantification of the chromatin bound PARP1 in Fig. 5c; 2 biological replicas are displayed with individual points. **c**. As described in Fig. 5a, trapping was induced in cells and subsequently chased as stated. Cells were then fractionated and the amount of chromatin-bound PARP1 was investigated by Western blotting. Data shown represent 3 biological replicas. **d**. CAL51 PARP1^WT^-eGFP cells were transfected with FLAG-RNF4-WT or FLAG-RNF-M136S/R177A (E2 binding mutant, dominant negative) constructs. Treatment occurred 24 h after expression. Data shown represent 2 biological replicas. **e**. HeLa cells were transfected with either p97-Strep-MYC cDNA or a p97 E578Q mutant-Strep-MYC. Sixteen hours later, cells were exposed to MMS + talazoparib. Data shown represent 2 biological replicas. **f**. CAL51 cells expressing PARP1^WT^-eGFP or PARP1^del.p119K120S^-eGFP were transfected with p97^WT^-Strep-MYC or p97^E578Q^-Strep-MYC-expressing construct. After trapping and pre-extraction, cells were fixed and imaged. Scale bar = 5 µm. **g**. Graph of quantification of PARP1–eGFP foci of the experiment presented in (F). n = 80-200 cells examined over 3 biologically independent experiments, mean ± SEM, *p*-values derived with a Kruskal-Wallis test. **h**. Western blots of trapped PARP1 from CAL51 PARP1–eGFP expressing cells transfected with a control siRNA (siLuc) or UFD1-targeting siRNA (siUFD1). 72 hours post transfection, cells were treated with MMS + talazoparib. Data shown represent 3 biological replicas.

We used a ‘trap–chase’ experimental approach to assess whether p97 removes trapped PARP1 from chromatin (Fig. 5a). Cells were exposed to MMS + PARPi to induce trapping (the ‘trap’) and then cultured in fresh media containing combinations of PARPi and p97-complex inhibitors (the ‘chase’). The amount of trapped PARP1 was evaluated either by chromatin fractionation or measuring the proximity of PARP1 to phosphorylated H2AX (γH2AX PLA^39^) at various time points during the chase. First, we followed the kinetics of trapped PARP1 in *PIAS4*^–/–^ and *RNF4*^–/–^ cells (Fig. 3a,d). Both *PIAS4*^–/–^ and *RNF4*^–/–^ cells showed slower resolution of chromatin-bound PARP1, especially at the later time points (Fig. 5b,c and Extended Data Fig. 7a,b), consistent with the idea that these SUMO/ubiquitin ligases promote the resolution of trapped PARP1 complex.

We then investigated the role of p97 activity with this assay by including talazoparib plus CB-5083 or CuET in the chase phase of the experiment and monitoring trapped PARP1 either via chromatin fractionation (Extended Data Fig. 7c) or PLA (Fig. 5d). After exposing cells to MMS + talazoparib, a significant amount of PARP1 was detected in the proximity of γH2AX (Fig. 5d), indicating that the ‘trapping’ part of the experiment was successful; after removing the trapping agents, the amount of trapped PARP1 decreased (for example, the PARP1–γH2AX PLA signal disappeared). The PARP1–γH2AX PLA signal also diminished when cells were chased in the presence of one agent—that is, a PARPi or a p97 inhibitor. Conversely, when cells were chased in the presence of both inhibitors, PARPi (talazoparib) and p97 inhibitor (either CB-5083 or CuET), the amount of trapped PARP1 persisted (Fig. 5d,e). Consistent with the idea that RNF4 is an upstream factor involved in the processing of trapped PARP1, we also found that gene silencing of *RNF4* led to the persistence of PARP1–γH2AX PLA foci (Fig. 5f). In addition, we assessed the effect on PARP1 trapping by the expression of a dominant-negative RNF4 p.M136S/R177A mutant (Extended Data Fig. 7d), a p97 p.E578Q mutant (Extended Data Fig. 7e–g) or UFD1 depletion (Extended Data Fig. 7h). All three interventions led to a higher level of trapped PARP1 in the chromatin fraction, confirming the importance of these proteins in the processing of trapped PARP1.

**Extended Data Fig. 8:**
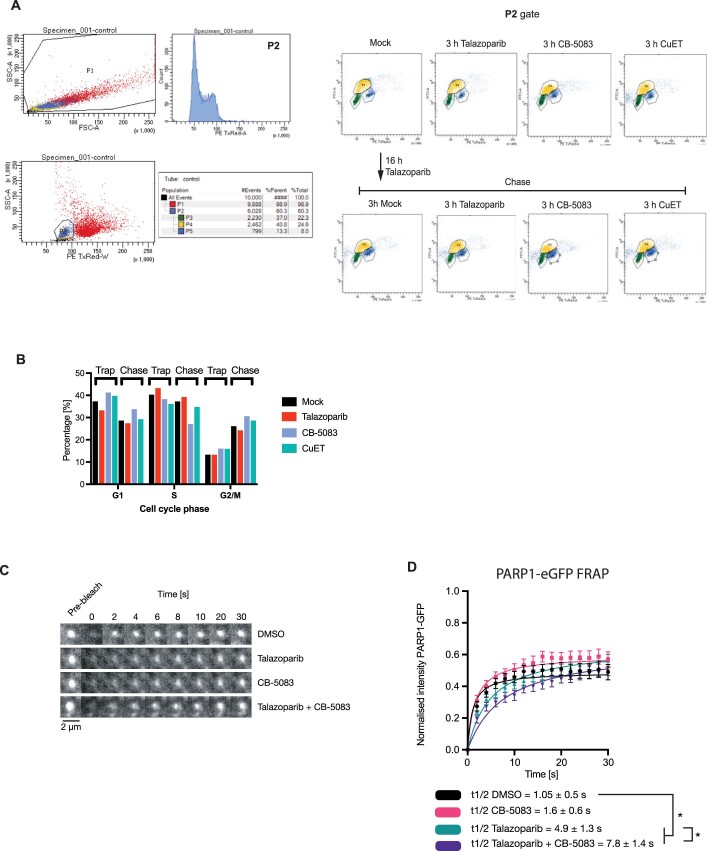
Cell cycle and ‘PARP1 exchange’ under p97 inhibition. **a**. Cell cycle profiling for the experiment shown in Fig. 5i. CAL51 cells were exposed to drugs as shown. One hour prior to fixation, 10 μM EdU was added to the media. EdU was stained by a click reaction with Alexa488-azide and DNA was stained by propidium iodide. **b**. A quantification of the G1, S and G2 populations from (A). **c, d**. CAL51 PARP1^WT^-eGFP cells were subjected to UV-micro-irradiation, accumulation of PARP1^WT^-eGFP at UV-laser induced DNA damage sites was monitored in the presence of DMSO (vehicle), talazoparib, CB-5083 or in combination. At the maximum time of PARP1^WT^-eGFP recruitment (typically 1 min after micro-irradiation) the focus was bleached with a 488 nm laser and recovery of PARP1^WT^-eGFP was monitored over time as described in^39^. **e**. Image montages of the micro-irradiation site for (L). Scale bar = 2 µm. **l**. A quantification of the FRAP described in (K). The fluorescent signal was scaled according to the maximum PARP1^WT^-eGFP immediately prior the photobleach to (equalling 1) and the signal immediately after photobleach (0), as in^39^. The FRAP data was fitted with one site-specific binding model of non-linear regression and the extra sum of squares F test was used to calculate the t_1/2_. The significance was determined with a two-sided t-test from two independent experiments, where 10 to 12 cells were quantified for each condition. * - p-value < 0.05.

In homologous-recombination proficient cells, trapped PARP1 activates RAD51-mediated DNA repair, monitored by assessing nuclear RAD51 foci. We found that a 16 h exposure of cells to PARPi elicited both γH2AX and RAD51 foci, but γH2AX and RAD51 foci diminished after 3 h when PARPi was removed from the culture media by washing, suggesting resolution of the DNA damage caused by trapped PARP1 (Fig. 5g–i). When we used p97 inhibitors (CB-5083 or CuET) in this chase period, the γH2AX and RAD51 foci persisted, indicating that the underlying trapped PARP1-related damage could not be resolved as efficiently. Incubating cells in the presence of p97 inhibitor alone did not induce γH2AX and RAD51 foci, suggesting that the persistence of γH2AX and RAD51 foci in experiments involving PARPi, followed by p97 inhibitor were indeed caused by PARPi. The effects on foci resolution were also not trivially explained by alterations in the cell cycle, as exposure of cells to p97 inhibitor for 3 h did not lead to significant changes in cell-cycle distribution (Extended Data Fig. 8a,b). We also noted that when we used fluorescence recovery after photobleaching (FRAP) to monitor the exchange of PARP1^WT^–eGFP at a UV laser stripe in the presence of a PARPi^40^, the addition of a p97 inhibitor (CB-5083) led to a modestly slower FRAP (PARP1^WT^–eGFP half-time of recovery (*t*_1/2_) of 4.9 ± 1.3 s in the presence of talazoparib versus 7.8 ± 1.4 s in the presence of talazoparib + CB-5083; two-sided *t*-test *P* < 0.05; Extended Data Fig. 8c,d).

### p97 inhibition potentiates PARPi cytotoxicity

**Fig. 6:**
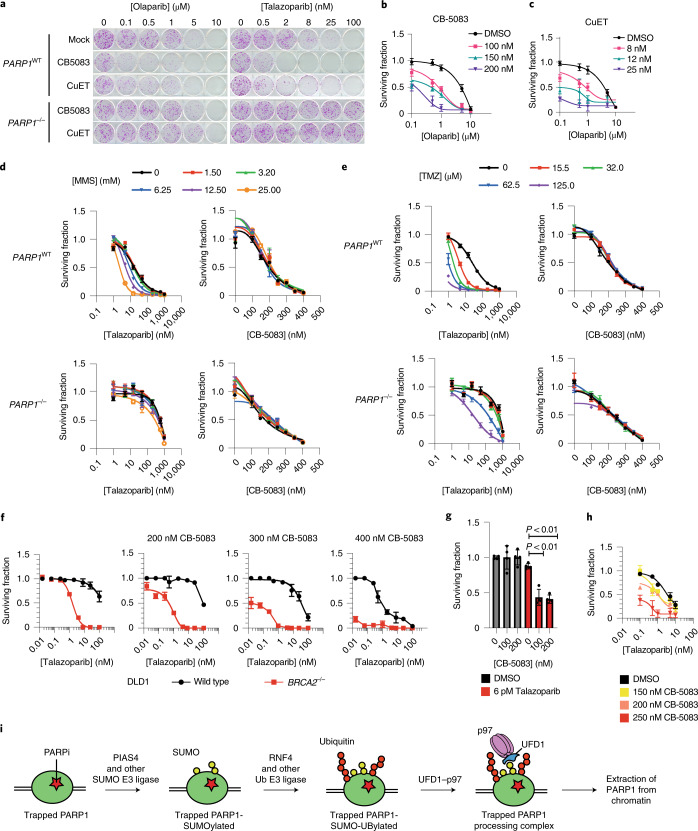
Inhibition of p97 potentiates the effect of PARPi. **a**, Inhibition of p97 potentiates the cytotoxicity of PARPi. CAL51 cells were exposed to PARPi (talazoparib (left) or olaparib (right)) in the presence of a p97 inhibitor (CB-5083 or CuET) for a period of 14 d. Images are shown for samples exposed to 100 nM CB-5083 and 8 nM CuET. **b**,**c**, Drug-response curves for CB-5083 (**b**) and CuET (**c**). See also Extended Data Fig. 9a,b. **d**,**e**, DNA alkylating agents that are used to induce PARP1 trapping do not enhance the cell-inhibitory effects of CB-5083. *PARP1*^WT^ and *PARP1*^–/–^ CAL51 cells were exposed to the alkylating agents MMS (**d**) or temozolomide (TMZ; **e**) in combination with either talazoparib (positive control) or CB-5083 for 7 d, after which the cell viability was measured. **f**, CB-5083 modulates the synthetic lethal effect of PARPi in *BRCA2*^–/–^ cells. Survival curves from clonogenic survival assays in *BRCA2*^WT^ and *BRCA2*^–/–^ DLD1 cells treated with different doses of CB-5083 and talazoparib. Colony formation images and quantification are shown in Extended Data Fig. 9e. **g**, Inhibition of p97 sensitizes mouse cancer organoid cells to PARPi. WB1P breast cancer organoids with *Brca1* and *p53* loss-of-function mutations were cultured in the presence of the indicated drugs for 7 d. Bright-field images of organoids are shown in Extended Data Fig. 9f. **h**, Inhibition of p97 sensitizes a human *BRCA1*-mutant patient-derived breast cancer organoid to PARPi. KCL014BCPO organoids were cultured in the indicated drugs for 7 d. Bright-field images of the organoids are shown in Extended Data Fig. 9g. **b**–**h**, Data are the mean ± s.d. of three biological replicates. **i**, Model of the processing of trapped PARP1. PARP1 trapped by the presence of PARPi on DNA is processed in a stepwise manner. It is initially SUMOylated in a PIAS4-dependant manner and subsequently ubiquitylated in an RNF4-dependent manner. p97 is recruited to the ubiquitin chains and binds via UFD1 and the ATPase activity of p97 extracts the modified PARP1 from the chromatin. DMSO, dimethylsulfoxide. Source data

**Extended Data Fig. 9:**
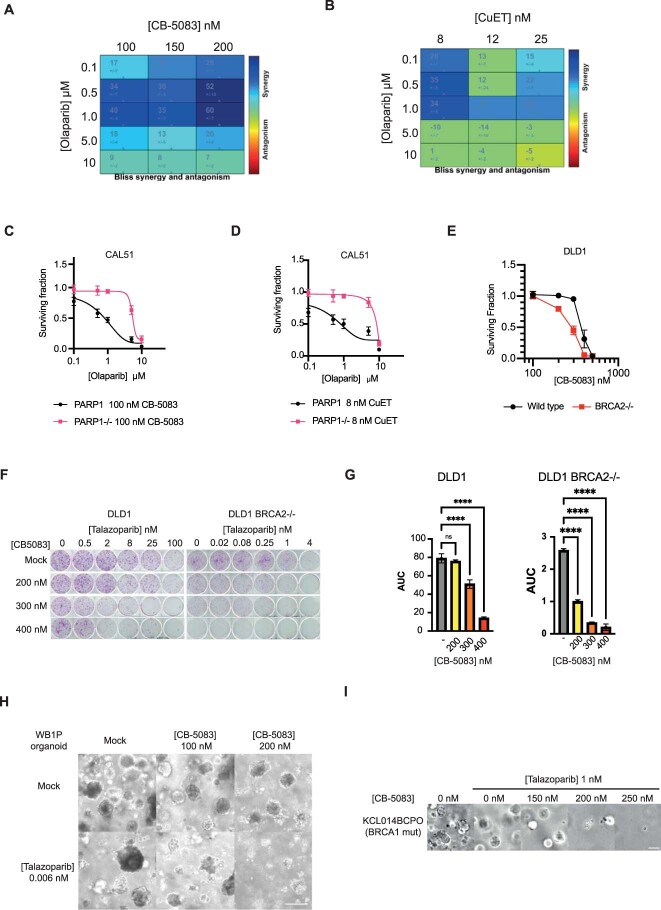
PARP inhibitors synergise with p97 inhibition. **a**. and **b**. Bliss synergy calculation, performed with the Combenefit software (CRUK Cambridge Institute), of the drug-response curves shown in Fig. 6b, c. **c**. and **d**. Drug-response curves for the colony formation assays presented in Fig. 6a. CAL51 WT or *PARP1*^−*/*−^ cells were treated with increasing concentrations of the PARP inhibitor Olaparib in the presence of either 100 nM CB-5083 (A) or 8 nM CuET (B). Surviving fractions were calculated based on the number of colonies after 14 days of exposure to the drugs. Shown are the mean ± SD of n = 3 biological replicas. **e**. A quantification of the CB-5083 single agent effect on the surviving fraction of DLD1 and DLD1 BRCA2^−/−^ cells, respectively. Shown are the mean ± SD of n = 3 biological replicas. **f**. Colony formation assays showing the synergistic effect between talazoparib and CB-5083 in DLD1 and DLD1 BRCA2^−/−^ cellular models. **g**. Area under the curve (AUC) analysis of the surviving fractions of DLD1 and DLD1 BRCA2^−/−^ cells in the presence of increasing concentrations CB-5083 combination as presented in Fig. 6f. Shown are the mean ± SD of n = 3 biological replicates; ordinary one-way ANOVA **** - *p* < 0.0001. **h**. Bright-field images, showing the effect of talazoparib-CB-5083 combination on the GEMM WB1P organoid as described in Fig. 6g. Scale bar = 200 µm. Data shown represent 2 biological replicas. **i**. Bright-field images showing the effect of talazoparib-CB-5083 combination on the KCL014BCPO organoid as described in Fig. 6h. Scale bar = 200 µm. Data shown represent 3 biological replicas.

Based on the prolonged PARP1-trapping effects described earlier, we hypothesized that p97 inhibition modulates the cytotoxic effects of PARPi. We assessed the effect of two p97 inhibitors (CB-5083 and CuET) on the cytotoxic effect of two trapping PARPi (talazoparib and olaparib) and observed a dose-dependent potentiation of the clonogenic effect of each PARPi by the presence of the p97 inhibitor (Fig. 6a–c). Bliss independence analysis confirmed that these drugs elicited supra-additive effects when used in combination (Extended Data Fig. 9a,b). This combinatorial effect was PARP1-trapping dependent as it was reversed in *PARP1*^–/–^ cells (Fig. 6a and Extended Data Fig. 9c,d), suggesting that it was also not due to other roles that p97 might play in DNA repair. Furthermore, p97 inhibitor (at concentrations used in the previous PARPi combinatorial experiments) did not enhance sensitivity to the alkylating agents MMS or temozolomide in either *PARP1*^WT^ or *PARP1*^–/–^ cells (Fig. 6d,e). This implied that other roles p97 might play in alkylation DNA-damage repair are unlikely to explain its ability to evict trapped PARP1 from chromatin and, following from that, the ability of CB-5083/CuET to sensitize to PARPi.

Because PARPi are approved for the treatment of cancers that have homologous-recombination defects and trapped PARP1 is the key cytotoxic event in homologous recombination-defective cells, we assessed the effect of combined exposure to CB-5083 + talazoparib in DLD1 cells with/without genetic ablation of *BRCA2*. When used alone, CB-5083 had a modest *BRCA2* synthetic lethal effect (Extended Data Fig. 9e), but it had a far greater effect on *BRCA2*^–/–^ DLD1 cells than isogenic wild-type *BRCA2* (*BRCA2*^WT^) cells when used in combination with talazoparib (Fig. 6f and Extended Data Fig. 9f,g). In tumour organoids derived from mice with combined *Brca1* and *Tp53* loss-of-function mutations (WB1P)^41^ we found that CB-5083 further sensitized tumour organoids to talazoparib (Fig. 6g and Extended Data Fig. 9h). We also assessed CB-5083 in combination with PARPi in a patient-derived organoid culture derived from a patient with triple-negative breast cancer harbouring a germline pathogenic BRCA1 p.R1203* mutation (*BRCA1* c.3607C>T), which was homozygous in the organoid. CB-5083 led to a marked shift in talazoparib sensitivity (Fig. 6h and Extended Data Fig. 9i), suggesting that p97 inhibition has the potential to potentiate the effects of PARPi in human tumour cells.

## Discussion

The effectiveness of PARPi in cancer treatment relies on their ability to trap PARP1 in the chromatin. Here we have delineated a biochemical cascade that processes trapped PARP1. Trapped PARP1 is sequentially SUMOylated by PIAS4 and then ubiquitylated by RNF4; these events recruit p97, whose ATPase activity removes PARP1 from chromatin (Fig. 6i). Importantly, interference with any of these processing steps leads to persistence of the trapped complex and enhanced PARPi sensitivity. Other factors might also influence this process, especially as other ubiquitin-processing enzymes are recruited to DNA damage and also PARP1 in a PAR-dependent manner (for example, the deubiquitylating enzyme ATXN3 (ref. ^42^) and the E3 ubiquitin ligase TRIP12 (ref. ^43^)).

During our studies we considered whether the effects of p97 modulation on PARP1 trapping/PARPi sensitivity might not be solely due to an effect of p97 on trapped PARP1 but could also be due to p97 modulating other DNA-repair processes. However, we think this unlikely for the following reasons: (1) the p97 inhibitors, when employed as single agents, did not elicit biomarkers of DNA damage such as γH2AX or RAD51 foci (Fig. 5g–l), or alterations in cell-cycle dynamics (Extended Data Fig. 8a,b); and (2) p97 inhibitors enhanced PARPi sensitivity in a PARP1-dependent manner (Fig. 6a,b), the p97 inhibitor CB-5083 did not alter sensitivity to MMS or temozolomide (Fig. 6c,d).

Our observations may lead to further questions. First, although PIAS4 and RNF4 seem to act in a linear manner, it is still possible that the balance of SUMOylation and ubiquitylation is influenced by other E3 ligases. The effect of losing PIAS4 on trapped PARP1 resolution was indeed modest (Fig. 5b), suggesting other proteins might also be involved. Second, our data also suggest that UFD1 is required for the recruitment of p97 to trapped PARP1 (Fig. 4j). How UFD1 recruits p97 to trapped PARP1 remains to be established. We note that canonically, UFD1 is thought to function as an obligate heterodimer with NPL4; however, we observed NPL4 silencing did not alter PARP1 trapping or the PARP1–p97 interaction, whereas UFD1 depletion did (Fig. 4j). Although we are unable to entirely rule out a role for NPL4 in the processing of trapped PARP1, it is possible that our described function of p97, similar to the removal of CDT1 and other substrates from chromatin^7,8,10^, is dependent on UFD1 only. Third, in most systems the p97-dependent removal of ubiquitylated proteins is coupled to proteasomal degradation, but this was not the case for PARP1. In addition, p97 is known to participate in substate recycling, as is the case for Aurora B^9^, yeast transcriptional repressor alpha^20^, Ub-LexA-VP16 (ref. ^19^) and MRE11 (ref. ^44^). This raises the possibility that PARP1 might also be a p97 substate that is recycled, not degraded.

Finally, the PARPi-generated DNA lesions seem to be processed in an analogous fashion to trapped TOP1-cleavage complexes^11^. Both PARPi and TOP1 inhibitors cause replication-fork stress and sensitivity in cells with homologous recombination defects and the sensitivity to both classes of agents is modulated by SLFN11 (ref. ^45^); both are SUMOylated, ubiquitylated and modified by p97 (reviewed in^46^ and data shown here). Thus, it seems plausible that the sensing and processing machinery that activate the SUMOylation and ubiquitylation of trapped PARP1 as well as trapped TOP1 and TOP2 cleavage complexes are also shared and not necessarily private to the precise nature of the nucleoprotein complexes, and might be related with their ability to interfere with normal DNA metabolism.

In conclusion, our work elucidates an elegant and highly orchestrated molecular machinery of PIAS4, RNF4 and UFD1–p97 that recognises and removes trapped PARP1 from chromatin.

## Methods

### Cells and cell culture

CAL51 (DSMZ, ACC 302), DLD1 (ATCC, CCL-221), DLD BRCA2^−/−^ (Horizon, HD 105-007), HeLa (ATCC, CCL-2, commonly misidentified cell line as set out by ICLAC; we did not authenticate but used directly from the ATCC) cells were maintained in DMEM medium supplemented with 10% fetal bovine serum (FBS) and 1×penicillin–streptomycin (Sigma-Aldrich). *PARP1*^−/−^ CAL51 cells were previously described^15^. They were transfected with a corresponding PARP1-expressing piggyBac construct in combination with hyPBase-expressing plasmid^47^. Single-cell clones were sorted by FACS 72 h after transfection and allowed to expand. These clones were characterized for the expression of the tagged protein by microscopy and western blotting. *PARP1*^–/–^ HEK293 cells were a gift from I. Ahel (University of Oxford)^48^. The HCT116 *PIAS4*^–/–^ and MCF7 *RNF4*^–/–^ cells were previously described^26^. The WB1P organoid line was previously described^49^. They were grown in a mix of 50% Matrigel (Corning) and 50% Advanced DMEM/F12 (Life Technologies) medium containing 10 mM HEPES (Sigma-Aldrich) pH 7.5, GlutaMAX (Invitrogen) and supplemented with 125 mM *N*-acetylcysteine (Sigma-Aldrich), B27 supplement and 50 ng ml^−1^ EGF (Life Technologies). The organoids were seeded at 10,000 cells per well of a 24-well plate and drugs were added at the indicated concentrations 24 h later. Cell viability was assessed using a 3D CellTiter-Glo assay (Promega). KCL014BCPO was derived (L.M.B. et al., manuscript in preparation), similarly to as described^50^. Briefly, human breast tumour samples were obtained from adult female patients after informed consent as part of a non-interventional clinical trial (BTBC study REC no. 13/LO/1248, IRAS ID 131133; principal investigator: A.N.J.T., study title: ‘Analysis of functional immune cell stroma and malignant cell interactions in breast cancer in order to discover and develop diagnostics and therapies in breast cancer subtypes’). This study had local research ethics committee approval and was conducted adhering to the principles of the Declaration of Helsinki. Specimens were collected from surgery and transported immediately. A clinician histopathologist or pathology-trained technician identified and collected tumour material into basal culture medium. The tumour samples were coarsely minced with scalpels and then dissociated using a Gentle MACS dissociator (Miltenyi). The resulting cell suspension was mechanically disrupted, filtered and centrifuged. The resulting cell pellets were then plated into three-dimensional (3D) cultures at approximately 1–2 ×10^3^ cells μl^−1^ in Ocello PDX medium and hydrogel. All cultures were maintained in humidified incubators at 37 °C and 5% CO_2_. All human cell-line identities were confirmed by short-tandem-repeat typing and verified to be free of mycoplasma infection using Lonza MycoAlert.

### Plasmids, antibodies and reagents

To generate PB-PARP1–eGFP, *PARP1* cDNA was cloned in a previously described piggyBac vector^51^. To generate the PARP1–Apex2–eGFP construct, the *Apex2* gene was amplified from Addgene vector 49386 and inserted between the *PARP1* and *eGFP* coding sequences via InFusion (Clonetek, 648910). PBZ–mRuby2 is described in^15^. Ub–Strep–HA was a gift from V. D’Angiolella; HA–SUMO2 was a gift from E. Yeh (ref. ^52^), FLAG–PARP1 was a gift from I. Ahel, p97-GFP was a gift from H. Mayer.

The wild-type PIAS4-expressing construct was obtained from Addgene (15208) and RNF4 from Origene (RC207273). The corresponding mutants with deleted SAP and SUMO-interacting motifs were generated as described^26^. The RNF4-M136S,R177A was a gift from R. Hay. Antibodies to the following were used: GFP (Sigma-Aldrich, 11814460001, clones 7.1 and 13.1; dilution: western blotting (WB), 1:5,000; immunofluorescence, 1:500; and PLA, 1:1,500), PARP (CST, 9532, 46D11; dilution 1:2,000 for immunoblotting and PLA), p97 (Abcam, ab11433 [5]; dilution: WB, 1:1,000; and PLA, 1:2,000), PAR (Trevigen, 4335-AMC-050; dilution: WB, 1:1,000), HA (Roche, 11867423001; dilution: WB, 1:5,000), FLAG (M2, Sigma-Aldrich, F1804; dilution: WB, 1:5,000 for immunoprecipitation), FLAG (Sigma-Aldrich F7425; dilution: WB, 1:5,000 for immunoblotting), Streptavidin–HRP (ThermoFisher, S911; dilution: WB, 1:1,000), PARP1 (Sigma-Aldrich, WH0000142M1; dilution: WB, 1:1,000; and PLA, 1:2,000), β-actin (Invitrogen, AM4302; dilution: WB, 1:5,000), lamin-B1 (ThermoFisher, PA5-19468; dilution: WB, 1:5,000), vinculin (Abcam, ab18058; dilution: WB, 1:5,000), phospho-H2AX (CST, 9718S; dilution: 1:2,000 for PLA), phospho-H2AX (Millipore, 05-636; dilution: 1:1,500 for foci immunostaining), RAD51 (Abcam, ab133534; dilution: 1:1,500 for foci immunostaining), histone H3 (CST, 9715; dilution: WB, 1:5,000), SUMO1 (CST, 4940; dilution: WB, 1:1,000), SUMO2/3 (CST, 4971; dilution: WB, 1:1,000), ubiquitin (Santa Cruz Biotechnology, sc-8017; dilution: WB, 1:1,000), RNF4 (Novusbio, NBP2-13243; dilution: WB, 1:1,000); UFD1L (Abcam, ab181080; dilution: WB, 1:1,000) and anti–rabbit IgG HRP (Rockland, 18-8816-31; dilution: WB, 1:5,000). Talazoparib was supplied by Pfizer as part of the BCN Catalyst programme. The following other small molecules were used: olaparib (Selleckchem, S1060), veliparib (Selleckchem, S1004), UKTT15 from in-house synthesis as described in^4^, MMS (Sigma-Aldrich, 129925-5G), CB-5083 (Selleckchem, S8101), CuET from in-house synthesis as described in^37^, MLN-7243 (Selleckchem, S8341) and ML-792 (Medchemexpress, HY-108702). The siRNAs were obtained from Dharmacon: RNF4 (L-006557-00-0005 and 3′ untranslated region siRNA sequence 5′-GGGCAUGAAAGGUUGAGAAUU-3′), UFD1L (L-017918-00-0005) and NPL4 (L-020796-01-0005).

### Western blotting

Standard protocols for SDS–PAGE and immunoblotting were used^53^. Nitrocellulose (GE Healthcare) or PVDF (BioRad) membranes were used to transfer proteins from polyacrylamide gels, depending on the antibody.

### Cellular fractionation immunoprecipitation

Cells were washed twice with PBS and then resuspended in buffer A (10 mM HEPES, 10 mM KCI, 340 mM sucrose, 10% glycerol, 2 mM EDTA, protease and phosphatase inhibitors, *N*-ethylmaleimide (NEM)). Triton X-100 was added to a final concentration of 0.1% and left on ice for 2–5 min, depending on the cell line. The supernatant was harvested as the cytosolic fraction and the pellet (nuclei) was then washed twice with buffer A. Buffer B (3 mM EDTA, 0.2 mM EGTA, 5 mM HEPES pH 7.9, protease and phosphatase inhibitors, NEM) was then added to burst the nuclei, after which lysates were kept on ice for 10 min. The supernatant was then removed as the nuclear-soluble fraction. The remaining chromatin pellet was then washed with buffer B in 0.5% Triton X-100, followed by benzonase buffer without MgCl_2_ (50 mM Tris–HCl pH 7.9, 100 mM NaCl). Benzonase digestion buffer (50 mM Tris–HCl pH 7.9, 10 mM MgCl_2_, 100 mM NaCl, protease and phosphatase inhibitors, NEM) supplemented with 125 U benzonase enzyme (Merk Millipore) was added to the pellet and rotated on a wheel at 4 °C for 1 h. The samples were then centrifuged at 20,000*g* for 15 min; chromatin input for the immunoprecipitation reaction was taken from the supernatant. The remaining supernatant was then incubated with the respective beads for 3 h at 4 °C with rotation. For native immunoprecipitations, ethidium bromide (1:200) was added to remove unwanted DNA–protein interactions. The beads were then washed three times with IP wash buffer (50 mM Tris–HCl pH 7.4, 150 mM NaCl, 0.5 mM EDTA, 0.05% Triton X-100) before elution with Laemmli buffer.

### Whole-cell immunoprecipitation

Cells were lysed in IP lysis buffer (50 mM Tris–HCl pH 7.4, 150 mM NaCl, 1 mM EDTA, 0.5% Triton X-100, protease and phosphatase inhibitors, NEM) and spun on a wheel at 4 °C for 10 min. The supernatant was removed, the pellet was washed once with benzonase buffer, and the supernatants were pooled together. Benzonase digestion buffer supplemented with 125 U benzonase enzyme (Merk Millipore) was added to the pellet and placed on a rotating wheel at 4 °C for 1 h. The samples were centrifuged at 20,000*g* and all supernatants were pooled. Input for the immunoprecipitation was then removed and the samples were incubated with the respective beads for 3 h at 4 °C with rotation. The beads were then washed three times with IP wash buffer before elution with Laemmlli buffer.

### Denaturing immunoprecipitation

Cells were lysed according to either the cell fractionation immunoprecipitation or whole-cell immunoprecipitation protocol as described earlier. Before incubation with beads, SDS was added to the samples to a concentration of 1% and boiled at 95 °C for 5 min. The samples were then diluted in 1% Triton X-100 to achieve a dilution of 1:10 (SDS at 0.1%) along with beads and rotated on a wheel at 4 °C for 3 h. The beads were then washed three times with IP wash buffer before elution with Laemmlli buffer.

### Cell viability and clonogenic survival assays

The viability of cells was measured using a CellTiter-Glo assay (Promega) after exposure to various concentrations of drugs for 6 d. The long-term effects of drug exposure were assessed by colony-formation assay after exposure of the cells to a drug-containing medium (refreshed weekly) for 12–14 d; the cells were stained at the end of the assay with sulforhodamine B. When plotting survival curves, the surviving fraction was calculated relative to the dimethylsulfoxide (solvent)-exposed cells.

The viability of the KCL014BCPO organoid line was measured using a 3D Cell Titre-Glo assay (Promega). Organoids were seeded in 24-well plates, with one 15 µl Matrigel droplet containing 3,000 cells per well. After 24 h, the organoids were treated with a drug-containing media (drug refreshed after 4 d) for 7 d before assessing their viability by measuring 3D Cell Titre-Glo luminescence.

### Apex2-mediated proximity labelling

For each condition tested, 5–10 × 10^6^ cells expressing PARP1–Apex2–eGFP were exposed to either 0.01% MMS or a combination of 0.01% MMS + 100 nM talazoparib for 1 h. In the last 30 min of the incubation, biotin-tyramide (Sigma-Aldrich, SML2135) was added to the media at a final concentration of 500 µM. To label proteins, H_2_O_2_ (Sigma-Aldrich, H1009) was added for 60 s at a final concentration of 1 mM. The reaction was quenched by washing the cells three times with freshly prepared quench solution (PBS containing 10 mM sodium ascorbate, 10 mM sodium azide, 5 mM Trolox (Sigma-Aldrich, 238813)). Subsequently, the cells were scraped in quench solution and washed twice in 0.1% IGEPAL CA-630 quench solution. The remaining nuclei were lysed in nuclear RIPA buffer (50 mM Tris–HCl pH 7.5, 1 M NaCl, 1% IGEPAL CA-630, 0.1% sodium deoxycholate, 1 mM EDTA) for 10 min on ice. The lysates were diluted with RIPA buffer without NaCl, to obtain a final concentration of 200 mM, sonicated for 1 min and incubated with 250 U benzonase for 20 min at room temperature. The lysates were clarified by centrifugation at 13,000*g* for 15 min at 4 °C. The protein concentration was determined and 1 mg total protein was incubated with 30 μl streptavidin-magnetic beads (ThermoFisher, 88816) for 1 h at room temperature. The beads were washed stringently by sequential washes—twice with RIPA lysis buffer, once with 1 M KCl, once with 0.1 M Na_2_CO_3_, once with 2 M urea and twice with RIPA lysis buffer—and processed further for mass spectrometry analysis.

### RIME of tagged protein

For each condition tested, 5–10 × 10^6^ cells were exposed to either 0.01% MMS or a combination of 0.01% MMS + 100 nM talazoparib for 1 h. At the end of the incubation period, formaldehyde (ThermoFisher, 28908) was added to the media to final concentration of 1% and incubated for 10 min at room temperature. The reaction was quenched by the addition of glycine (125 mM final concentration). The cells were collected and washed once in ice-cold PBS. The cells were resuspended in ice-cold PBS containing 0.1% Triton X-100 and protease inhibitor cocktail (Merck, 4693116001). The nuclei were centrifuged at 3,000*g* for 5 min at 4 °C, resuspended in PBS containing 1% IGEPAL CA-630 (Sigma-Aldrich) and protease inhibitors, and incubated on ice for 15 min. The remaining chromatin was centrifuged at 13,000*g* for 5 min at 4 °C and resuspended in PBS containing 0.1% IGEPAL CA-630 and protease inhibitors. The chromatin pellet was centrifuged at 13,000*g* for 5 min at 4 °C, resuspended in lysis buffer (20 mM HEPES pH 7.5, 150 mM NaCl, 0.5% sodium deoxycholate, 0.1% SDS, 10 mM MgCl_2_) supplemented with 250 U benzonase (Sigma-Aldrich, E1014) and incubated for 30 min at room temperature with rotation to release the chromatin-bound proteins. The supernatant was isolated after centrifugation (13,000*g* for 10 min at 4 °C) and incubated with 25 μl GFP-Trap (Chromotek, gtm-20) magnetic beads for 1 h at 4 °C with rotation. The beads were washed four times with the lysis buffer and processed further for mass spectrometry analysis.

### Mass spectrometry and data analysis

After initial washes according to the purification method, the beads were further washed twice with 50 mM ammonium bicarbonate. The proteins on the beads were digested with 0.1 μg μl^−1^ sequencing-grade trypsin (Roche) at 37 °C overnight. The peptide solution was neutralized with 5% formic acid; acetonitrile was added (60% final concentration) and the solution was filtered through a Millipore Mutiscreen HTS plate (pre-washed with 60% acetonitrile). The peptide solution was lyophilized on a SpeedVac and the peptides were dissolved in 20 mM TCEP–0.5% formic acid solution. The liquid chromatography with tandem mass spectrometry analysis was conducted on the Orbitrap Fusion Tribrid mass spectrometer coupled with a U3000 RSLCnano UHPLC system (ThermoFisher). The peptides were first loaded on a PepMap C18 trap (100 μm inner diameter × 20 mm, 100 Å, 5 μm) at 10 μl min^−1^ with 0.1% formic acid in H_2_O; they were then separated on a PepMap C18 column (75 µm inner diameter × 500 mm, 100 Å, 2 μm) at 300 nl min^−1^ and a linear gradient of 4–32% acetonitrile in 0.1% formic acid in 90 min with the cycle at 120 min. Briefly, the Orbitrap full MS survey scan was *m*/*z* 375–1,500 with a resolution of 120,000 at *m*/*z* 200, with the automatic gain control set at 40,000 and maximum injection time at 50 ms. Multiply charged ions (*z* = 2–5) with an intensity above 8,000 (for Lumos) or 10,000 (for Fusion) counts were fragmented in a higher collision dissociation cell at 30% collision energy and the isolation window at 1.6 Th. The fragment ions were detected in ion-trap mode with the automatic gain control at 10,000 and a maximum injection time of 35 ms. The dynamic exclusion time was set at 40 s with ±10 ppm.

The mass spectrometry proteomics data have been deposited to the ProteomeXchange Consortium via the PRIDE^54^ partner repository with the dataset identifier PXD024337. Raw mass spectrometry data files were analysed using Proteome Discoverer 1.4 (Thermo). Database searches were carried out using Mascot (version 2.4) against the UniProt human reference database (January 2018; 21,123 sequences) with the following parameters: trypsin was set as digestion mode with a maximum of two missed cleavages allowed. Precursor mass tolerance was set to 10 ppm and the fragment mass tolerance was set to 0.5 Da. Acetylation at the amino terminus, oxidation of methionine, carbamidomethylation of cysteine, and deamidation of asparagine and glutamine were set as variable modifications. Peptide identifications were set at a false-discovery rate of 1% using Mascot Percolator. Protein identification required at least one peptide with a minimum score of 20. For the Apex2-based proximity labelling mass spectrometry, the following steps were taken. Proteins identified with a single peptide were removed from further analysis. The PARP1–eGFP mass spectrometry profile under trapping conditions (MMS + talazoparib) was used as a negative control. Proteins identified with >2 unique peptides in this sample were removed from further analyses. Peptide spectrum matches (PSM) was used as a proxy of protein abundance in the samples. A ratio was built between the PARP1–Apex2–eGFP MMS + talazoparib PSM and PARP1–Apex2–eGFP MMS PSM as an indicator for enrichment in the trapping conditions. Where PSM values were absent from the PARP1–Apex2–eGFP MMS PSM (that is, no detection in the sample) a value of one was added to calculate a meaningful ratio (data provided in Supplementary Table 1). The list of genes was then searched on the STRING database to build a network of the hits. A high-confidence threshold was set for mapping the network, using a minimum required interaction score of 0.7 for connecting nodes. Single unconnected nodes were excluded from the network plots. The gene list was searched in the Enrichr database to assess which Kyoto Encyclopedia of Genes and Genomes 2019 pathway annotations are enriched in the dataset. The list of annotations was filtered using −log(*P*) values of 1.3 (*P* = 0.05) or 2 (*P* = 0.01; data are provided in Supplementary Table 2). For RIME analysis, the proteins identified with single peptides were removed from further analyses. Proteins identified in *PARP1*^–/–^ CAL51 cells were considered as background and removed from further analyses when they were identified with more than two unique peptides. Subsequently, the mass spectrometry data obtained from PARP1^WT^–eGFP or PARP1^del.p.119K120S^–eGFP cells were considered separately. For each cell line, a ratio was built between the MMS + talazoparib PSM and the MMS PSM as an indicator for enrichment in the trapping conditions. Where PSM values were absent from the MMS PSM (that is, no detection in the sample), a value of one was added to calculate a meaningful ratio (data in Supplementary Tables 3 and 4 for PARP1^WT^–eGFP and PARP1^del.p.119K120S^–eGFP, respectively).

### PLA

The PLA assays were carried out using a Duolink in situ red starter kit mouse/rabbit kit (Sigma-Aldrich) according to the manufacturer’s protocol. The primary antibodies used were: mouse anti-PARP1 (Sigma-Aldrich, WH0000142M1), rabbit anti-PARP (Cell Signaling), mouse anti-p97 (Abcam, ab11433) and rabbit anti-phospho-H2AX (Cell Signaling). The antibodies were used at a 1:1,500 dilution. Images were acquired on a Marianas advanced spinning disk confocal microscope (3i) and analysed using a custom CellProfiler pipeline. Typically, several hundred nuclei were counted per condition from at least two independent biological repeats.

### Micro-irradiation

Cells were cultured in glass-bottomed culture dishes (MaTek, P35G-0.170-14-C) in 10% FBS DMEM media and maintained at 37 °C and 5% CO_2_ in an incubation chamber mounted on the microscope. Imaging was carried out on an Andor Revolution system, ×60 water objective with micropoint at 365 nm. For FRAP analysis, the cells were acquired one at a time; each cell was irradiated at a single spot with 1 µm diameter in the nucleus. After the signal of recruitment reached its maximum (typically 30–60 s after micro-irradiation), the recruitment spot was bleached with a 488 nm laser and imaging continued with one frame per interval of 2 s. For each experimental condition, 10–12 cells were acquired and the experiment was repeated independently on a different imaging day. From the raw intensities of the micro-irradiation site, the spot intensity immediately before bleaching was set to one and the intensity immediately after bleaching to zero. The recovery data were fitted with one site-specific binding model of nonlinear regression (GraphPad Prism software) and the extra-sum-of-squares *F*-test was used to calculate the *t*_1/2_.

### Cell-cycle analysis

Cells were incubated in the presence of inhibitors for the corresponding amount of time. Ethylene-deoxyuridine (10 µM; ThermoFisher) was added to the media 1 h before fixation. Subsequently, the cells were trypsinized and fixed in ice-cold absolute ethanol. The cells were rehydrated via a PBS wash and permeabilized with 0.5% Triton X-100 in PBS for 15 min at room temperature with rotation. After a PBS wash, a click chemistry reaction cocktail was added to the cells (100 mM Tris–HCl pH 7.6, 4 mM CuSO_4_, 2.5 µM azide–Fluor 488 (Sigma), 100 mM sodium ascorbate (Sigma)) and incubated for 30 min at room temperature, protected from light. After a PBS wash, propidium iodide/RNase staining solution (Thermo) was added to the cells for 30 min. The cell-cycle profiles were acquired on a BD LSRII flow cytometer and analysed using the BD FACSDiva software.

### Chromatin fractionation

The chromatin fractionation assay for PARP trapping was based on a previously published protocol^2^. For the trap–chase experiments, cells were cultured in six-well plates, exposed to 100 nM talazoparib and 0.01% MMS for 1 h and subsequently incubated in media containing the appropriate drugs (typically 100 nM talazoparib, 10 μM CB-5083 or 1 M CuET) for a chase period of 3 h. The cells were fractionated using a Subcellular protein fractionation kit for cultured cells (ThermoFisher, 78840) according to the manufacturer’s recommendations.

### Statistics and reproducibility

No statistical method was used to pre-determine the sample size. No data were excluded from the analyses. The experiments were not randomized. The investigators were not blinded to allocation during experiments and outcome assessment. Denaturing and co-immunoprecipitations were performed twice, showing reproducibility, unless specified otherwise in the legends. For pre-extraction-based immunofluorescence microscopy, quantification and statistics were derived from *n* = 3 independent experiments. Immunofluorescence and PLA experiments were conducted in at least *n* = 3 independent biological repeats, and for each repeat, a few hundred cells were scored per condition. The data were pooled and analysed by ordinary one-way ANOVA (GraphPad Prism 9). Cellular growth inhibition assays were performed for at least *n* = 3 independent biological repeats and the statistical significance was derived using a two-way ANOVA (GraphPad Prism 9).

### Reporting Summary

Further information on research design is available in the Nature Research Reporting Summary linked to this article.

## Online content

Any methods, additional references, Nature Research reporting summaries, source data, extended data, supplementary information, acknowledgements, peer review information; details of author contributions and competing interests; and statements of data and code availability are available at 10.1038/s41556-021-00807-6.

## Supplementary information

Reporting Summary

Peer Review Information

Supplementary Table 1 Supplementary Table 1. List of proteins identified in the PARP1WT–eGFP RIME experiments. Supplementary Table 2. List of proteins identified in the PARP1del.p.119K120S–eGFP RIME experiments. Supplementary Table 3. List of proteins identified in the PARP1–Apex2–eGFP proximity labelling experiments. Supplementary Table 4. Gene ontology terms for the proteins identified in the PARP1–Apex2–eGFP proximity labelling experiments. The table contains the *P* values from Fisher’s exact tests.

## Data Availability

Mass spectrometry proteomics data have been deposited in the ProteomeXchange Consortium via the PRIDE partner repository (dataset identifier PXD024337). All other data supporting the findings of this study are available from the corresponding authors on reasonable request. Source data are provided with this paper.

## Source data

Source Data Fig. 1 Statistical source data.
Source Data Fig. 2 Statistical source data.
Source Data Fig. 2 Unprocessed western blots and/or gels.
Source Data Fig. 3 Unprocessed western blots and/or gels.
Source Data Fig. 4 Statistical source data.
Source Data Fig. 4 Unprocessed western blots and/or gels.
Source Data Fig. 5 Statistical source data.
Source Data Fig. 5 Unprocessed western blots and/or gels.
Source Data Fig. 6 Statistical source data.
Source Data Extended Data Fig. 1 Unprocessed western blots and/or gels.
Source Data Extended Data Fig. 2 Unprocessed western blots and/or gels.
Source Data Extended Data Fig. 3 Statistical source data.
Source Data Extended Data Fig. 3 Unprocessed western blots and/or gels.
Source Data Extended Data Fig. 4 Unprocessed western blots and/or gels.
Source Data Extended Data Fig. 5 Statistical source data.
Source Data Extended Data Fig. 5 Unprocessed western blots and/or gels.
Source Data Extended Data Fig. 6 Statistical source data.
