# Supplementary material for: The ubiquitin-dependent ATPase p97 removes cytotoxic trapped PARP1 from chromatin
Source: Nat Cell Biol. 2022 Jan 10;24(1):62–73. doi: 10.1038/s41556-021-00807-6 (PMC8760077; doi:10.1038/s41556-021-00807-6)
Supplement: Supplementary file 1 — Reporting Summary [file 41556_2021_807_MOESM1_ESM.pdf]

nature research

Corresponding author(s): Stephen J. Pettitt, Andrew N.J. Tutt, Kristijan Ramadan and Christopher J. Lord

Last updated by author(s): Oct 11, 2021

Reporting Summary

Nature Research wishes to improve the reproducibility of the work that we publish. This form provides structure for consistency and transparency in reporting. For further information on Nature Research policies, see our Editorial Policies and the Editorial Policy Checklist.

Statistics

For all statistical analyses, confirm that the following items are present in the figure legend, table legend, main text, or Methods section.

n/a	Confirmed	
☐	☒	The exact sample size (*n*) for each experimental group/condition, given as a discrete number and unit of measurement
☐	☒	A statement on whether measurements were taken from distinct samples or whether the same sample was measured repeatedly
☐	☒	The statistical test(s) used AND whether they are one- or two-sided<br>*Only common tests should be described solely by name; describe more complex techniques in the Methods section.*
☒	☐	A description of all covariates tested
☒	☐	A description of any assumptions or corrections, such as tests of normality and adjustment for multiple comparisons
☐	☒	A full description of the statistical parameters including central tendency (e.g. means) or other basic estimates (e.g. regression coefficient) AND variation (e.g. standard deviation) or associated estimates of uncertainty (e.g. confidence intervals)
☐	☒	For null hypothesis testing, the test statistic (e.g. *F*, *t*, *r*) with confidence intervals, effect sizes, degrees of freedom and *P* value noted<br>*Give P values as exact values whenever suitable.*
☒	☐	For Bayesian analysis, information on the choice of priors and Markov chain Monte Carlo settings
☒	☐	For hierarchical and complex designs, identification of the appropriate level for tests and full reporting of outcomes
☒	☐	Estimates of effect sizes (e.g. Cohen's *d*, Pearson's *r*), indicating how they were calculated

Our web collection on statistics for biologists contains articles on many of the points above.

Software and code

Policy information about availability of computer code

Data collection	Western blots were imaged and processed using ImageLab (BioRad) v5.2.1 and Imagej 1.53c.<br>PLA assays were imaged using Marianas Advanced Spinning disk confocal microscope 3i.<br>Image acquisition for trapped PARP1 colocalisation immunofluorescence was achieved using Andor Dragonfly Fusion software.<br>Image acquisition for trapped PARP1 accumulation after p97EQ expression was achieved using Nikon NIS-elements.<br>FACs profiles were acquired and analyzed with BD FACSdiva software.
Data analysis	Clonogenic curves, PLA graphs, immunofluorescence graphs were produced using GraphPad Prism v7.<br>Representative images for PLA and immunofluorescence were analysed using ImageJ 1.53c.<br>Quantification of trapped PARP1 and PLA were analysed by CellProfiler 4.1.3.<br>FACs profiles were analyzed with BD FACSdiva v9.0.

For manuscripts utilizing custom algorithms or software that are central to the research but not yet described in published literature, software must be made available to editors and reviewers. We strongly encourage code deposition in a community repository (e.g. GitHub). See the Nature Research guidelines for submitting code & software for further information.

Data

Policy information about availability of data

All manuscripts must include a data availability statement. This statement should provide the following information, where applicable:

- Accession codes, unique identifiers, or web links for publicly available datasets
- A list of figures that have associated raw data
- A description of any restrictions on data availability

The mass spectrometry proteomics data (Figure 1) have been deposited to the ProteomeXchange Consortium via the PRIDE partner repository with the dataset

identifier PXD024337. Source data are provided with this study. All other data supporting the findings of this study are available from the corresponding author on reasonable request.

Field-specific reporting

Please select the one below that is the best fit for your research. If you are not sure, read the appropriate sections before making your selection.

☒ Life sciences ☐ Behavioural & social sciences ☐ Ecological, evolutionary & environmental sciences

For a reference copy of the document with all sections, see nature.com/documents/nr-reporting-summary-flat.pdf

Life sciences study design

All studies must disclose on these points even when the disclosure is negative.

Sample size	For PLA, each experiment was replicated 3 times, with 200-250 total cells quantified for each condition, sample size was determined by accepted standard in the field for accurate assessment of interaction by PLA.<br>For PARP1 trapping immunofluorescence, no sample size calculations were made. Instead sample size was dependent on the frequency of successful transfection and expression, three replicate experiments were carried out and in total for each condition 80-220 cells were quantified.
Data exclusions	No relevant data was excluded from this study.
Replication	The experiments presented in this manuscript were typically performed in 2-3 biological replicates, with each attempt at replication successful. Number of times of experiments were replicated are indicated in the Methods section.
Randomization	In each experiment, different cell samples started from similar conditions and treatments were randomly allocated.
Blinding	Data did not require blinding as no qualitative measurements were taken. Furthermore, quantification of foci from PLA, immunofluorescence and survival in clonogenic assays in different conditions were all achieved using automatic software based protocols in Cellprofiler, so blinding was not performed to remove bias.

Reporting for specific materials, systems and methods

We require information from authors about some types of materials, experimental systems and methods used in many studies. Here, indicate whether each material, system or method listed is relevant to your study. If you are not sure if a list item applies to your research, read the appropriate section before selecting a response.

Materials & experimental systems

n/a	Involved in the study	
☐	☒	Antibodies
☐	☒	Eukaryotic cell lines
☒	☐	Palaeontology and archaeology
☒	☐	Animals and other organisms
☒	☐	Human research participants
☒	☐	Clinical data
☒	☐	Dual use research of concern

Methods

n/a	Involved in the study	
☒	☐	ChIP-seq
☐	☒	Flow cytometry
☒	☐	MRI-based neuroimaging

Antibodies

Antibodies used

GFP (Sigma-Aldrich, 11814460001, clones 7.1 and 13.1, dilution WB 1:5000 IF 1:500 PLA 1:1500);
PARP (CST, 9532, 46D11, dilution 1:2000) for immunoblotting and PLA;
p97 (Abcam, ab11433 [5], dilution WB 1:1000 PLA 1:2000) for immunoblotting and PLA;
PAR (Trevigen, 4335-AMC-050, dilution WB 1:1000);
HA (Roche, 11867423001, dilution WB 1:5000);
FLAG (M2, Sigma-Aldrich, F1804, dilution WB 1:5000) for immunoprecipitation;
FLAG (Sigma-Aldrich F7425, dilution WB 1:5000) for immunoblotting;
Streptavidin-HRP (ThermoFisher, S911, WB dilution 1:1000);
PARP1 (Sigma-Aldrich, WH0000142M1, dilution WB 1:1000 PLA 1:2000) for PLA;
β-actin (Invitrogen, AM4302, dilution WB 1:5000);
lamin-B1 (Thermo, PA5-19468, dilution WB 1:5000);
vinculin (Abcam, ab18058. dilution WB 1:5000);
phospho-H2AX (CST, 9718S, dilution 1:2000) for PLA;
phospho-H2AX (Millipore, 05-636, dilution 1:1500) for foci immunostaining;
RAD51 (Abcam, ab133534, dilution 1:1500) for foci immunostaining;
Histone H3 (CST, 9715, dilution WB 1:5000);

SUMO1 (CST, 4940, dilution WB 1:1000);
SUMO2/3 (CST, 4971, dilution WB 1:1000);
ubiquitin (Santa Cruz Biotechnology, sc-8017, dilution WB 1:1000);
RNF4 (Novusbio, NBP2-13243, dilution WB 1:1000);
UFD1L (Abcam, ab181080, dilution WB 1:1000);
Anti-Rabbit IgG HRP (Rockland, 18-8816-31, dilution WB 1:5000).

Validation

No homemade antibodies were used in this study, all antibodies were commercially validated as below.
GFP (Sigma-Aldrich, 11814460001)
Anti-GFP is tested for functionality and purity relative to a reference standard to confirm the quality of each new reagent preparation.
Purity: Both Anti-GFP mouse monoclonal antibodies (Clones 7.1 and 13.1) are >95% pure as determined by SDS-PAGE and ion-exchange HPLC analyses.
https://www.sigmaaldrich.com/GB/en/product/roche/11814460001?context=product

PARP (CST, 9532)
Validation found here: https://www.cellsignal.co.uk/products/primary-antibodies/parp-46d11-rabbit-mab/9532

p97 (Abcam, ab11433) for immunoblotting and PLA;
Validation found here: https://www.abcam.com/vcp-antibody-5-ab11433.html?#description_references

PAR (Trevigen, 4335-AMC-050);
Validation found here: https://trevigen.com/products-services/cell-stress-and-dna-damage/dna-damage/cell-stress-and-dna-damage-dna-damage-parp-parg-and-tankyrase/cell-stress-and-dna-damage-dna-damage-parp-parg-and-tankyrase-antibodies/anti-par-monoclonal-antibodyaffinity-purified/

HA (Roche, 11867423001);
Function tested in western blot.
https://www.sigmaaldrich.com/GB/en/product/roche/roahaha

FLAG (M2, Sigma-Aldrich, F1804) for immunoprecipitation;
Validation found here: https://www.sigmaaldrich.com/GB/en/product/sigma/f1804

FLAG (Sigma-Aldrich F7425) for immunoblotting;
Validation found here: https://www.sigmaaldrich.com/GB/en/product/sigma/f7425

Streptavidin-HRP (ThermoFisher, S911);
Validation found here: https://www.thermofisher.com/order/catalog/product/S911#/S911

PARP1 (Sigma-Aldrich, WH0000142M1) for PLA;
Validation found here: https://www.sigmaaldrich.com/GB/en/product/sigma/wh0000142m1

β-actin (Invitrogen, AM4302);
Validation found here: https://www.thermofisher.com/antibody/product/beta-Actin-Antibody-clone-AC-15-Monoclonal/AM4302

lamin-B1 (Thermo, PA5-19468, dilution);
Validation found here: https://www.thermofisher.com/antibody/product/Lamin-B1-Antibody-Polyclonal/PA5-19468

vinculin (Abcam, ab18058 [EPR19579]);
Validation found here: https://www.abcam.com/vinculin-antibody-epr19579-ab207440.html

phospho-H2AX (CST, 9718S);
Validation found here: https://www.cellsignal.co.uk/products/primary-antibodies/phospho-histone-h2a-x-ser139-20e3-rabbit-mab/9718

phospho-H2AX (Millipore, 05-636, dilution 1:500) for foci immunostaining;
Validation found here: https://www.merckmillipore.com/GB/en/product/Anti-phospho-Histone-H2A.X-Ser139-Antibody-clone-JBW301,MM_NF-05-636

RAD51 (Abcam, ab133534);
Validation found here: https://www.abcam.com/rad51-antibody-epr40303-ab133534.html

Histone H3 (CST, 9715);
Validation found here: https://www.cellsignal.co.uk/products/primary-antibodies/histone-h3-antibody/9715

SUMO1 (CST, 4940);
Validation found here: https://www.cellsignal.co.uk/products/primary-antibodies/sumo-1-c9h1-rabbit-mab/4940

SUMO2/3 (CST, 4971, dilution WB 1:1000);
Validation found here: https://www.cellsignal.co.uk/products/primary-antibodies/sumo-2-3-18h8-rabbit-mab/4971

ubiquitin (Santa Cruz Biotechnology, sc-8017, dilution WB 1:1000);
Validation found here: https://www.scbt.com/p/ubiquitin-antibody-p4d1

RNF4 (Novusbio, NBP2-13243, dilution WB 1:1000);
Validation found here: https://www.novusbio.com/products/rnf4-antibody_nbp2-13243

UFD1L (Abcam, ab181080, dilution WB 1:1000);
Validation found here https://www.abcam.com/ufd1l-antibody-epr12847-n-terminal-ab181080.html

Anti-Rabbit IgG HRP (Rockland, 18-8816-31, dilution WB 1:5000).
Validation found here: https://rockland-inc.com/Product.aspx?id=42151

Eukaryotic cell lines

Policy information about cell lines

Cell line source(s)	CAL51 WT were obtained from DSMZ (ACC 302).<br>CAL51 PARP1-/-, CAL51 PARP1-/- PARP1WT-eGFP,CAL51 PARP1/-/ PARP1KS-eGFP were validated in this study and previously in: Krastev, D.B. et al. Coupling bimolecular PARylation biosensors with genetic screens to identify PARylation targets. Nat Commun 9, 2016 (2018).<br>CAL51 PARP1-/- PARP1-APEX2-eGFP were generated and validated in this study<br>HEK293 were obtained from ATCC CRL-1573.<br>HeLa were obtained from ATCC CCL-2.<br>HEK293 PARP1-/- were generated and validated in: Ian Gibbs Seymour et al. , 2016, Mol Cell, https://www.ncbi.nlm.nih.gov/pmc/articles/PMC4858568/<br>HCT116 WT were obtained from ATCC (CCL-247).<br>MCF7 WT were obtained from ATCC (HTB-22).<br>HCT116 PIAS4-/-, MCF7 RNF4-/- were generated and validated in: Sun, Y. et al. A conserved SUMO pathway repair topoisomerase DNA-protein cross-links by engaging ubiquitin-mediated proteasomal degradation. Sci Adv 6 (2020).<br>DLD1 WT from ATCC (CCL-221)<br>DLD1 BRCA2-/- from Horizon (HD 105-007)
Authentication	CAL51 cell lines were not authenticated in our hands for this manuscript but were obtained and used directly from DSMZ (ACC 302) https://www.dsmz.de/collection/catalogue/details/culture/ACC-302.<br>The same is true for:<br>HEK293 https://www.atcc.org/products/crl-1573<br>HeLa https://www.atcc.org/products/ccl-2<br>MCF7 https://www.atcc.org/products/htb-22<br>HCT116 https://www.atcc.org/products/ccl-247<br>DLD1 WT https://www.atcc.org/products/ccl-221<br>DLD1 BRCA2-/- https://horizondiscovery.com/en/engineered-cell-lines/products/cancer-cell-lines?nodeid=entrezgene-675
Mycoplasma contamination	All cell lines used in this study were tested negative to mycoplasma by the MycoAlertTM Mycoplasma Detection Kit (Lonza LT07-218).
Commonly misidentified lines<br>(See ICLAC register)	No misidentified cell lines were used in this study.

Flow Cytometry

Plots

Confirm that:

☒ The axis labels state the marker and fluorochrome used (e.g. CD4-FITC).

☒ The axis scales are clearly visible. Include numbers along axes only for bottom left plot of group (a 'group' is an analysis of identical markers).

☒ All plots are contour plots with outliers or pseudocolor plots.

☒ A numerical value for number of cells or percentage (with statistics) is provided.

Methodology

Sample preparation	CAL51 tissue culture grown cell line was treated with drugs shown and subsequently pulsed with EdU for 20 min. The cells were trypsinized and click-chemistry was used to stain the EdU-positive cells with azide-FITC fluorophore. The cellular DNA was counterstained with propidium iodide.
Instrument	BD LSRII
Software	BR FACSdiva v9
Cell population abundance	From the total population of cells single cell were selected (P2) based on their side scatter (SSC-A) and PE TxRed-W scatter plot. They represented 60% of the total cellular suspension.

Gating strategy

Single cells were selected based on the SSC-A and PE TxRed-W scatter plot. The P2 population was subsequently plotted on a PE TxRed-A vs. FITC-A scattrer plot in order to select the G1 (P3), S (P4) and G2 (P5) populations.

☒ Tick this box to confirm that a figure exemplifying the gating strategy is provided in the Supplementary Information.
