# Supplementary material for: The ubiquitin-dependent ATPase p97 removes cytotoxic trapped PARP1 from chromatin
Source: Nat Cell Biol. 2022 Jan 10;24(1):62–73. doi: 10.1038/s41556-021-00807-6 (PMC8760077; doi:10.1038/s41556-021-00807-6)
Supplement: Source Data Fig. 2 — Unprocessed western blots and/or gels. [file 41556_2021_807_MOESM6_ESM.pdf]

Fig 2a

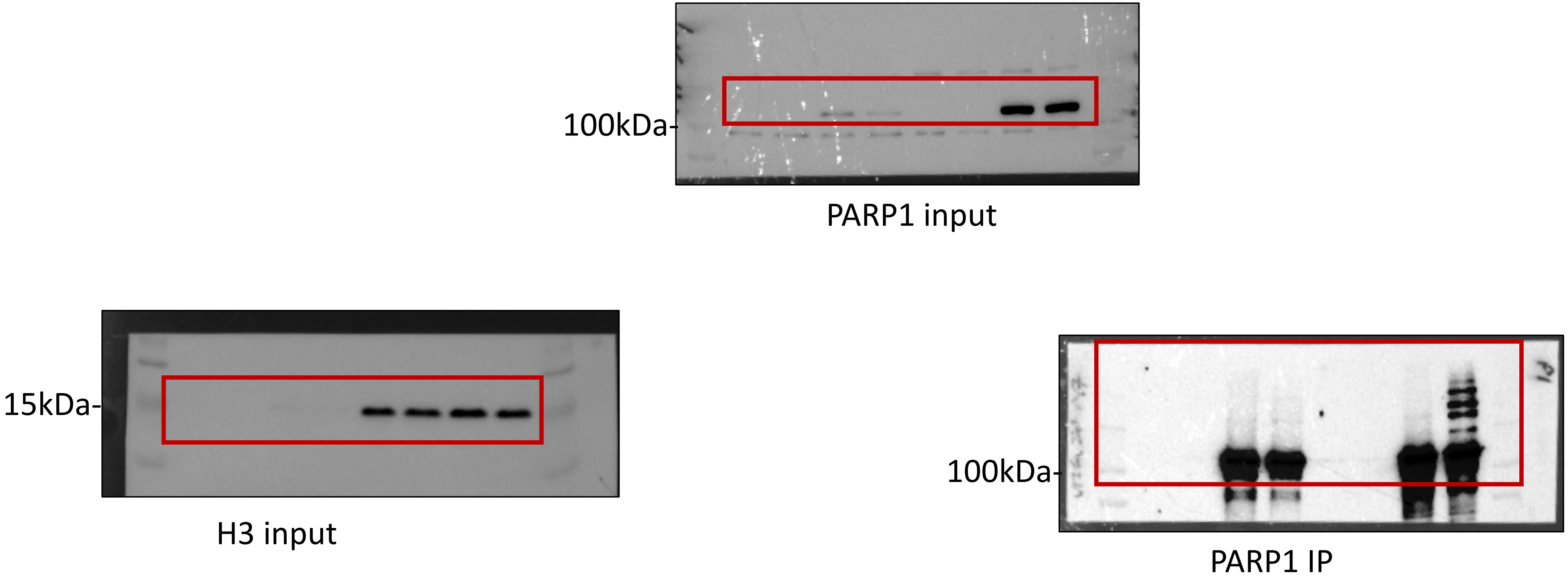

Fig 2b

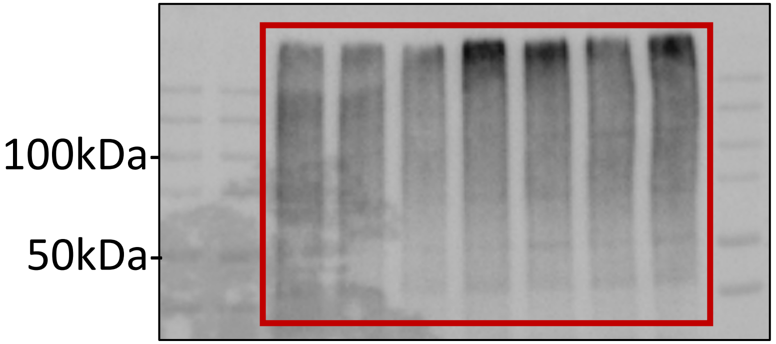

HA IP

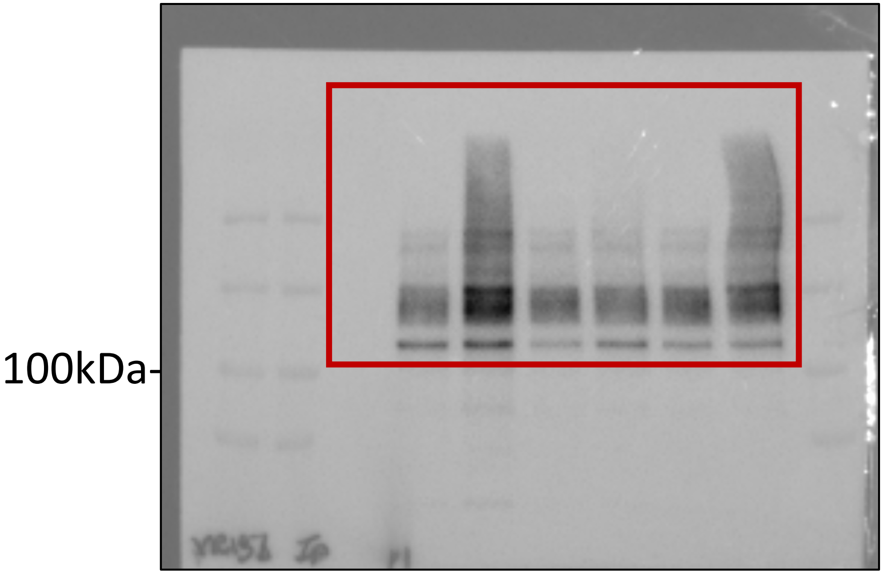

PARP1 IP

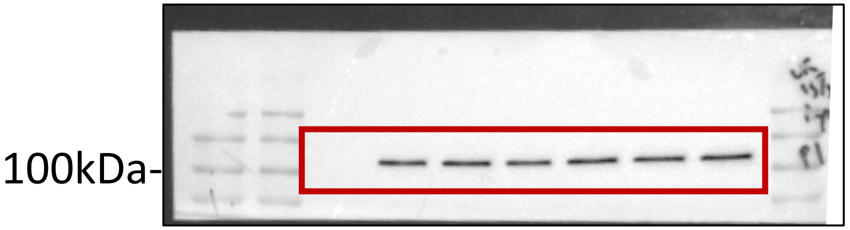

PARP1 input

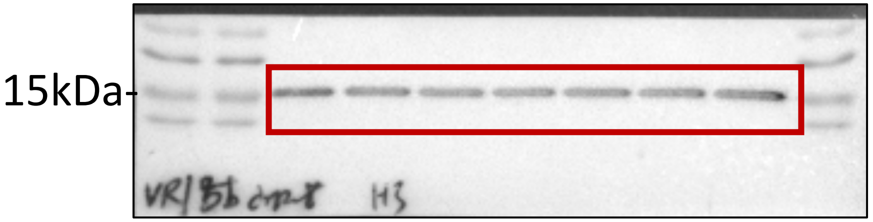

H3 input

Fig 2C

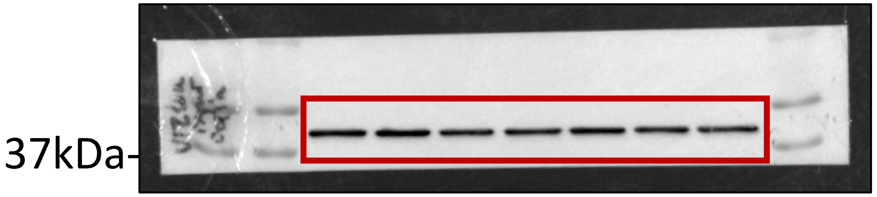

Actin input

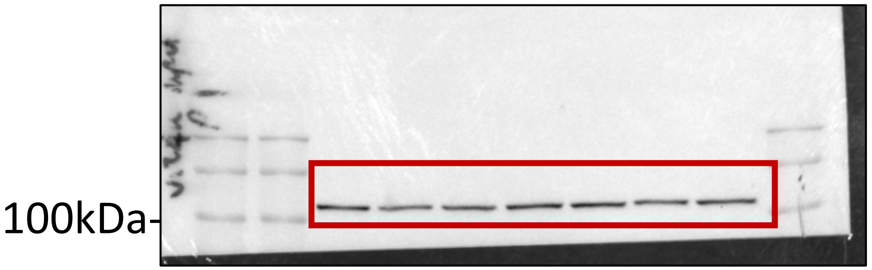

PARP1 input

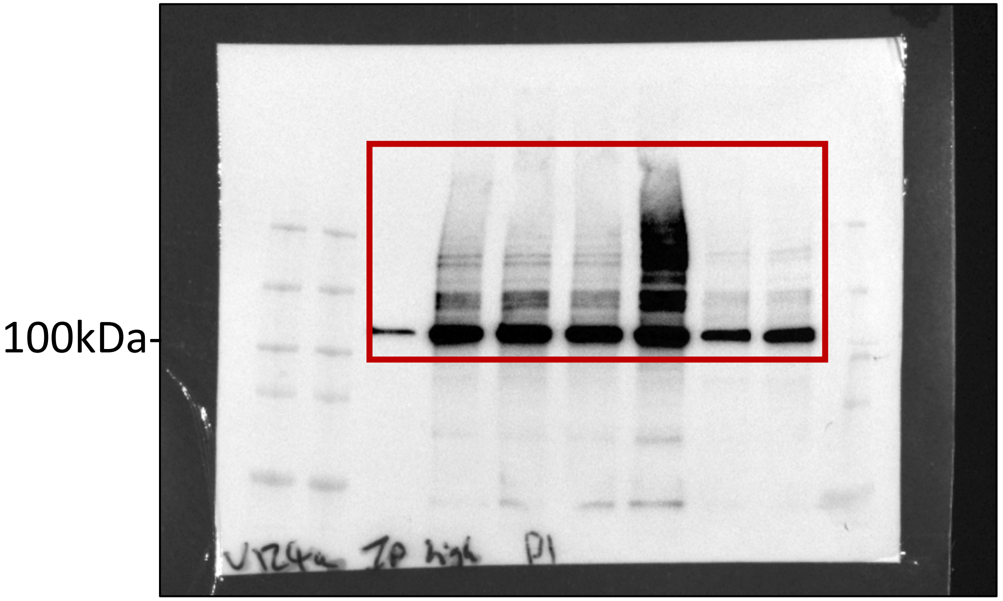

PARP1 IP

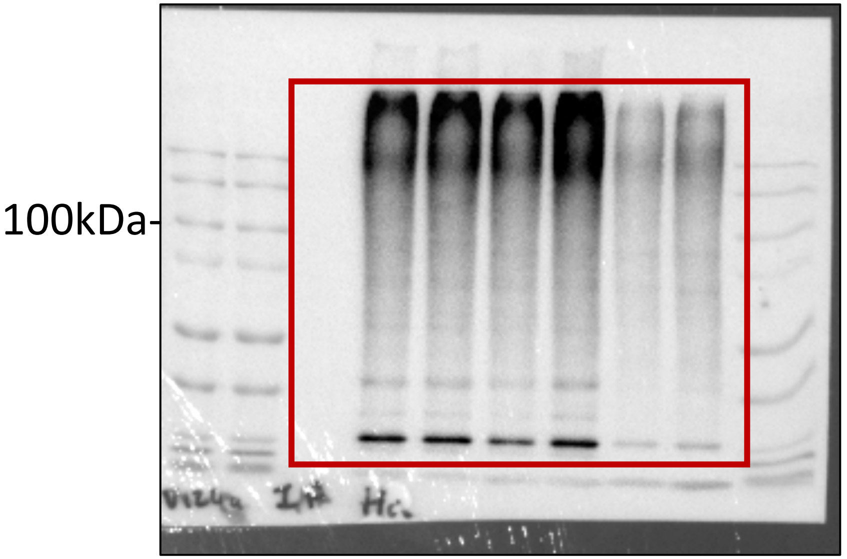

HA IP

Fig 2D

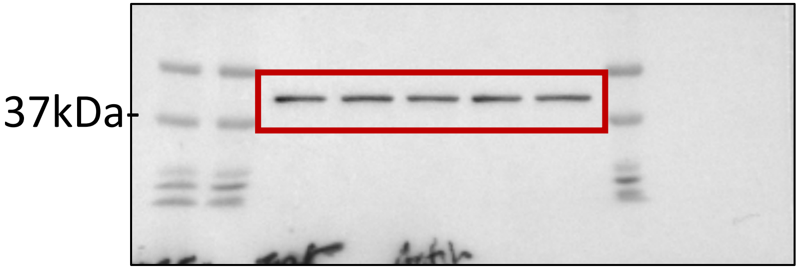

Actin input

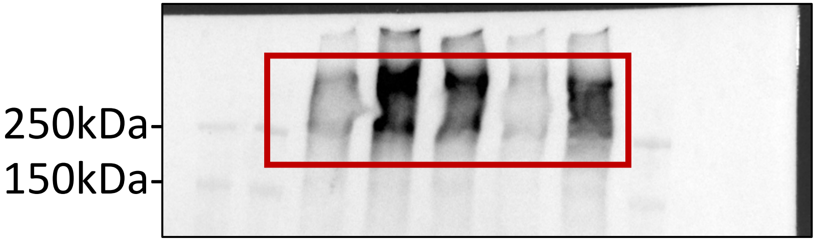

HA-SUMO2 IP

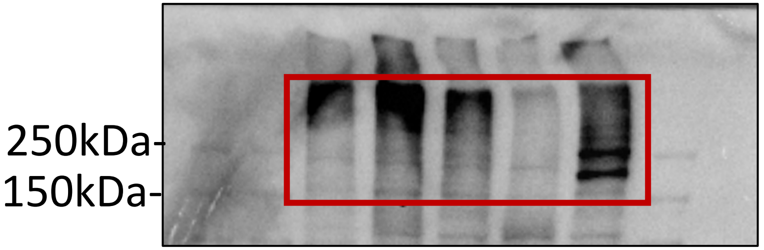

HA-SUMO2 Input

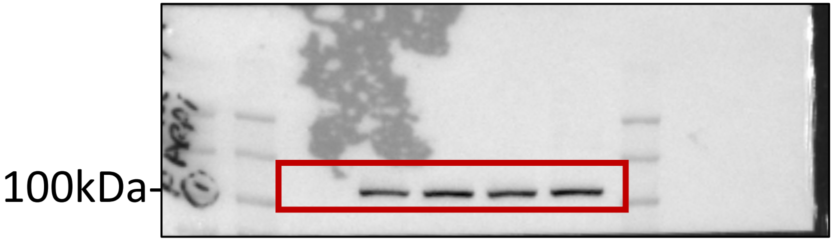

PARP1 Input

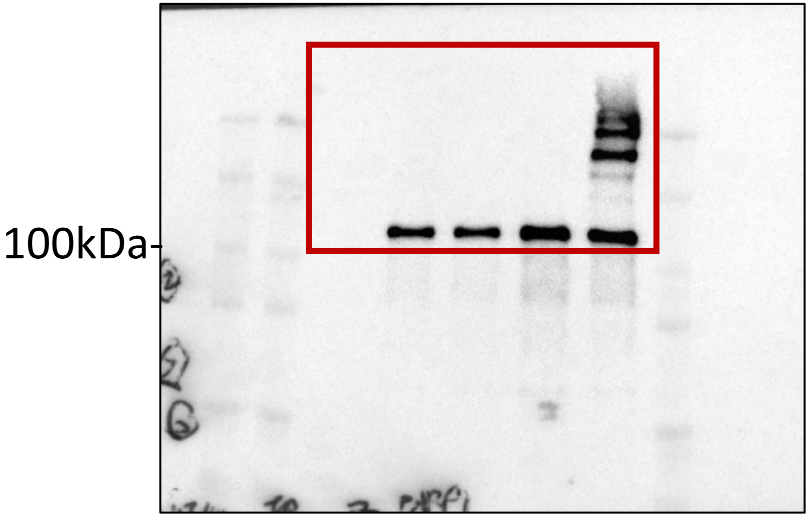

PARP1 IP

Fig 2E

15kDa-

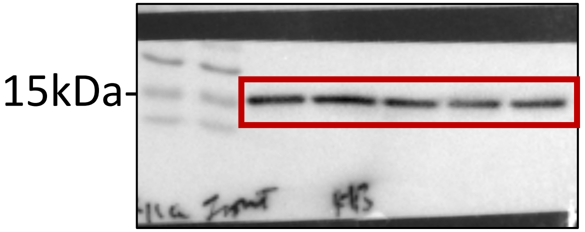

H3 Input

100kDa-

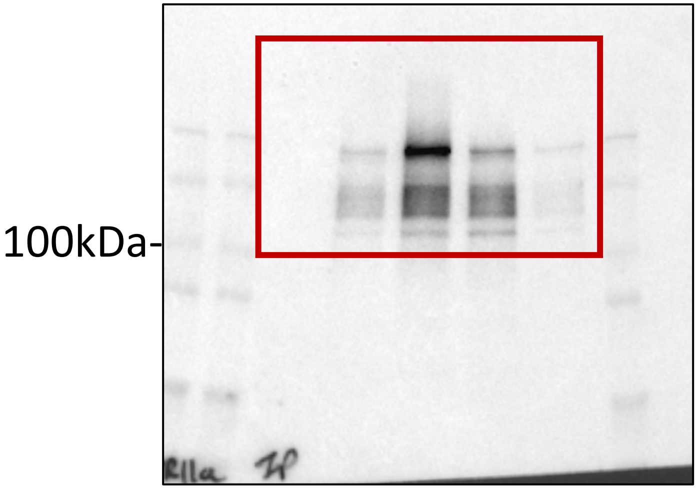

PARP1 IP

250kDa-

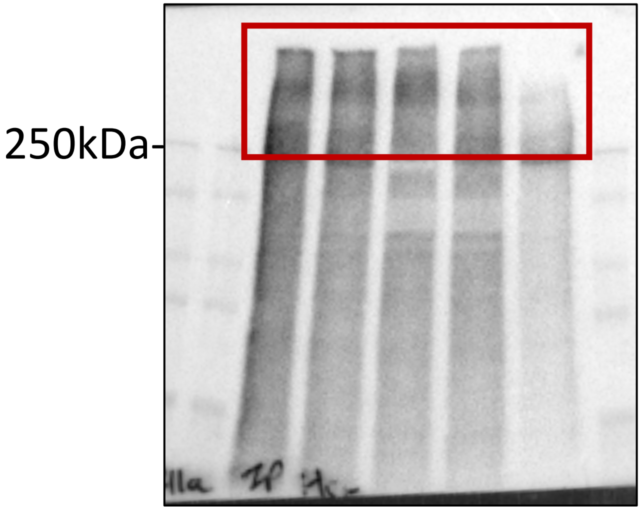

HA IP

50kDa-

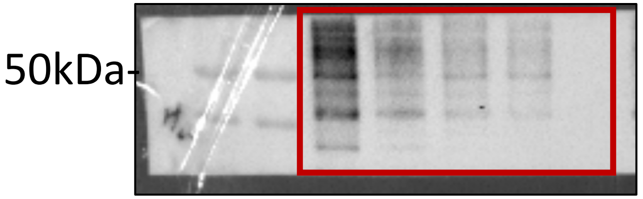

HA input

100kDa-

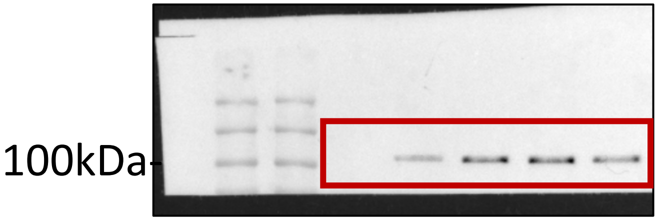

PARP1 input

150kDa-

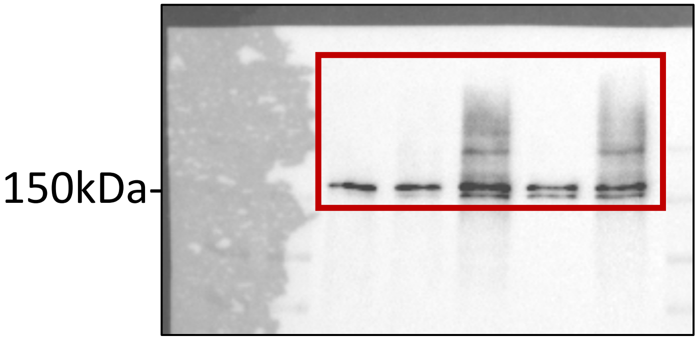

SUMO2/3 Input

Fig 2F

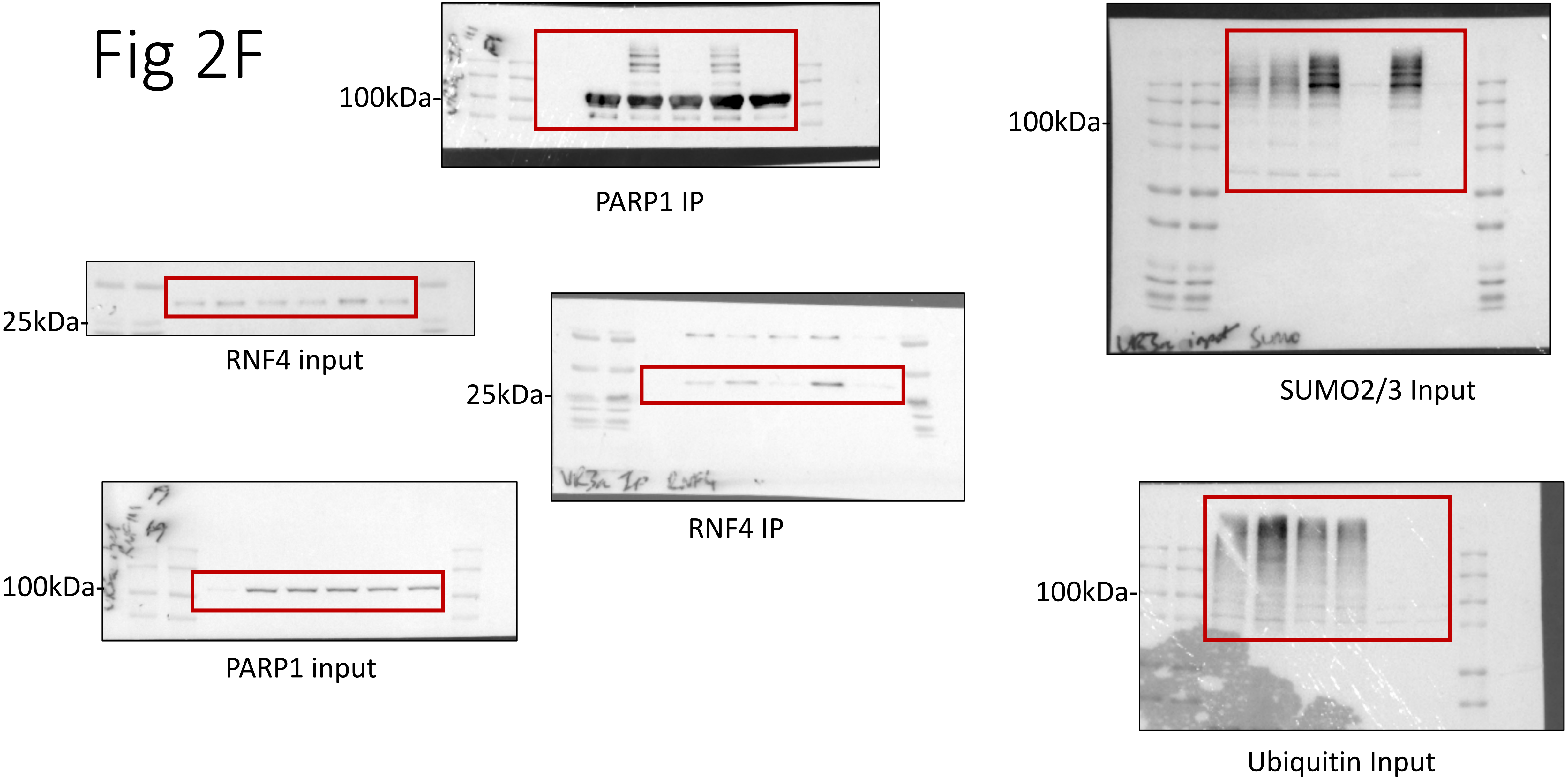
100kDa-
PARP1 IP
25kDa-
RNF4 input
25kDa-
RNF4 IP
100kDa-
PARP1 input
100kDa-
SUMO2/3 Input
100kDa-
Ubiquitin Input
