# Supplementary figures and images for: The ubiquitin-dependent ATPase p97 removes cytotoxic trapped PARP1 from chromatin
Source: Nat Cell Biol. 2022 Jan 10;24(1):62–73. doi: 10.1038/s41556-021-00807-6 (PMC8760077; doi:10.1038/s41556-021-00807-6)

Fig 3A

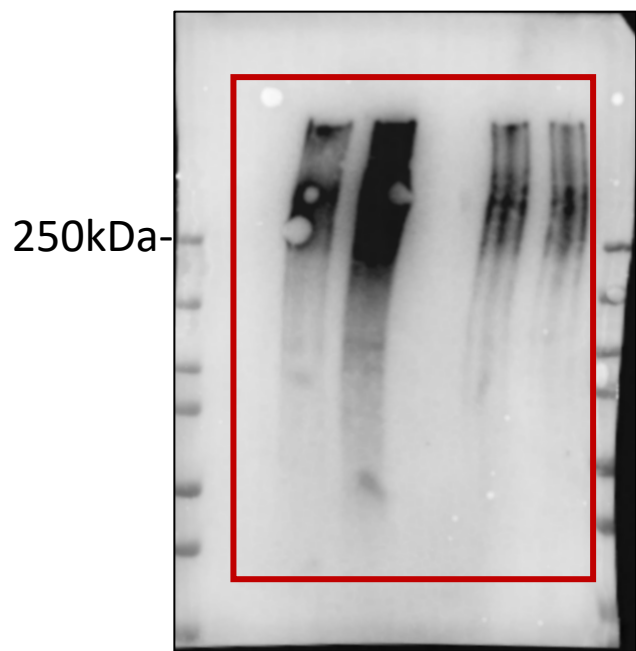

Ubiquitin IP

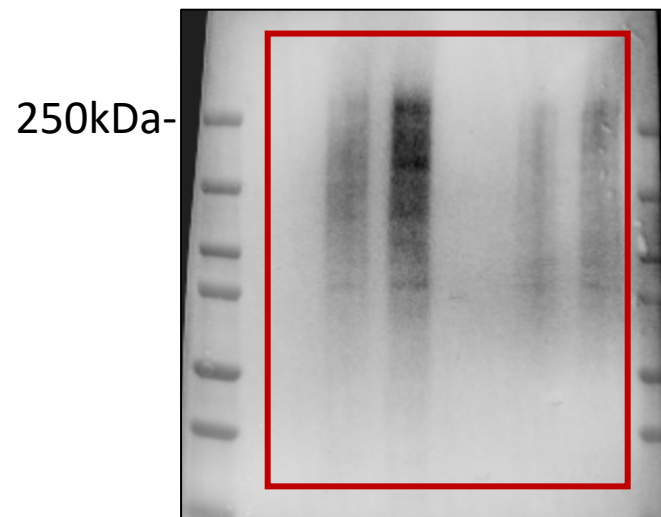

SUMO2 IP

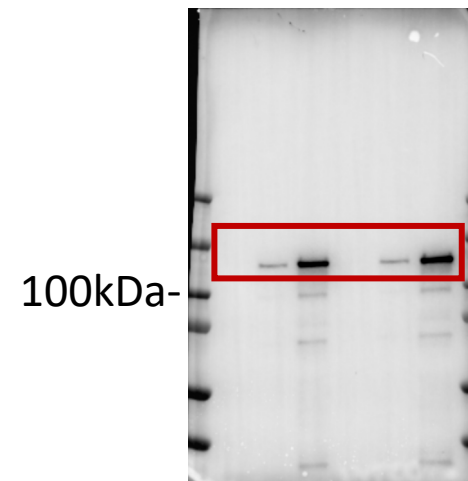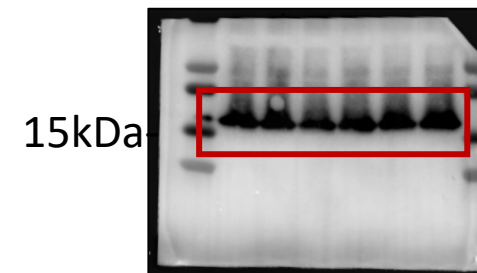

H3 input

Fig 3B

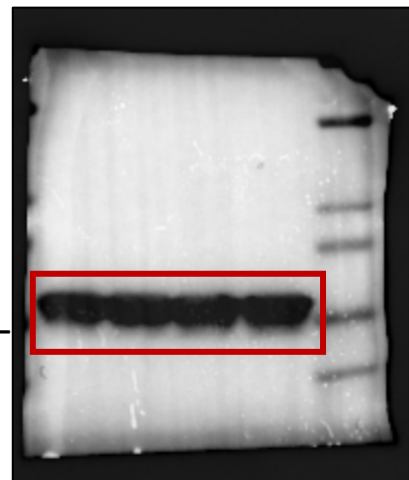

H3 input

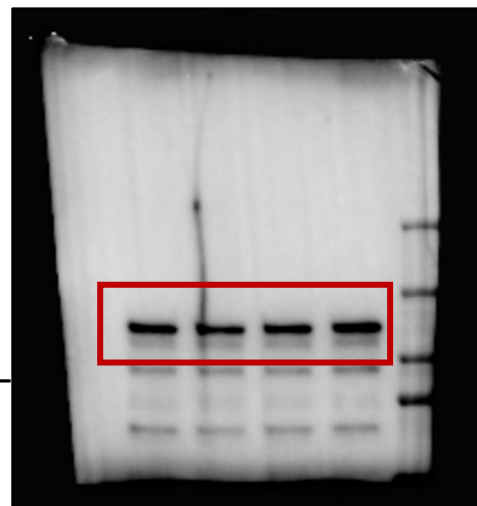

PARP1 input

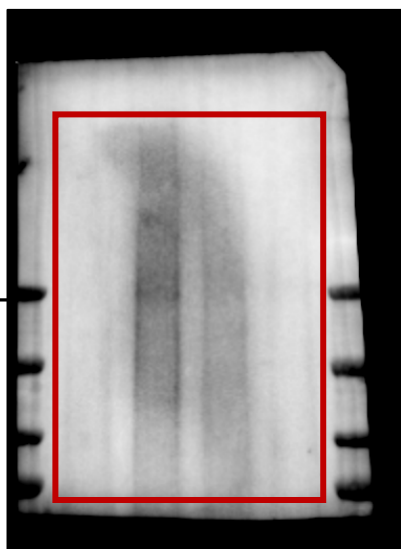

Ub IP

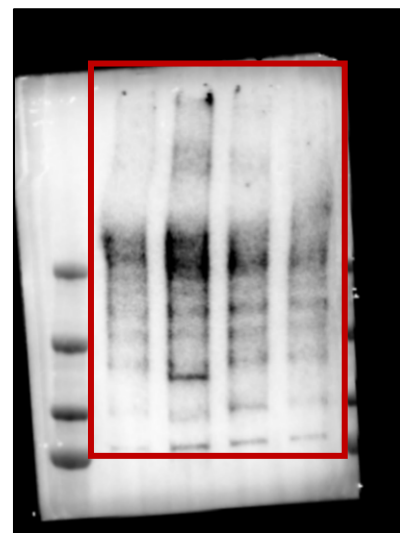

SUMO2/3 IP

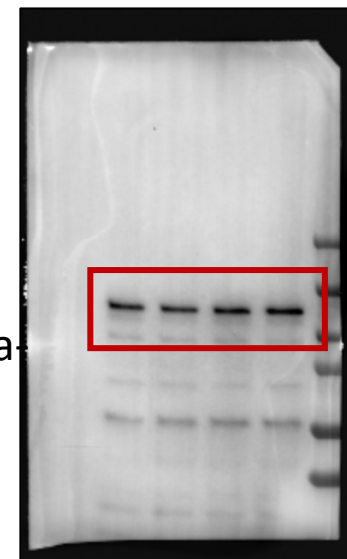

PARP1 IP

Fig 3D

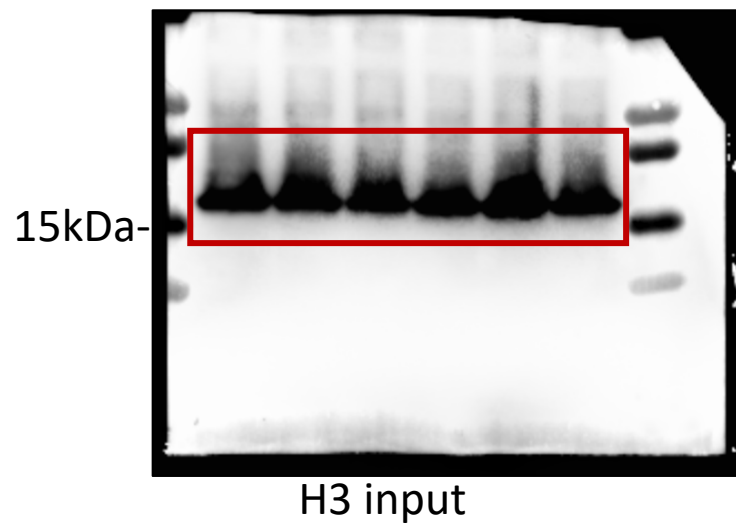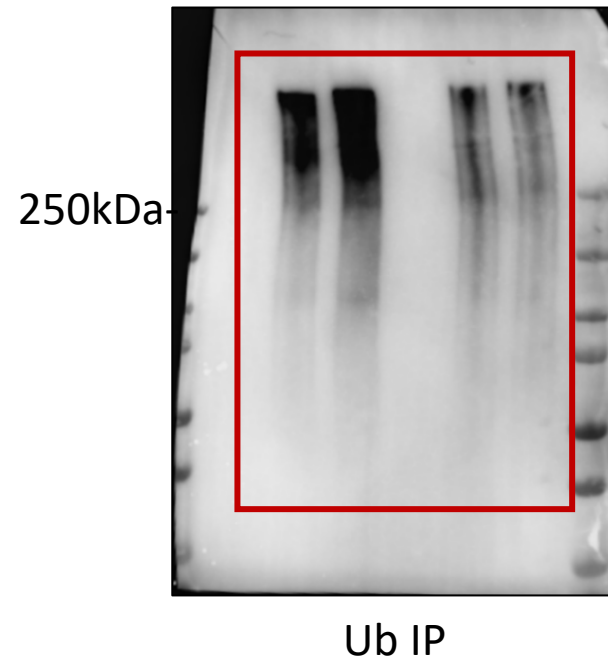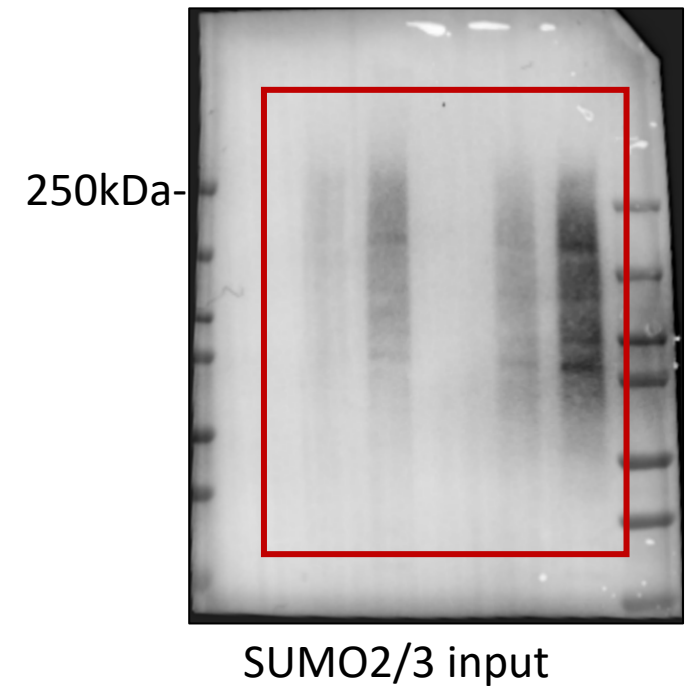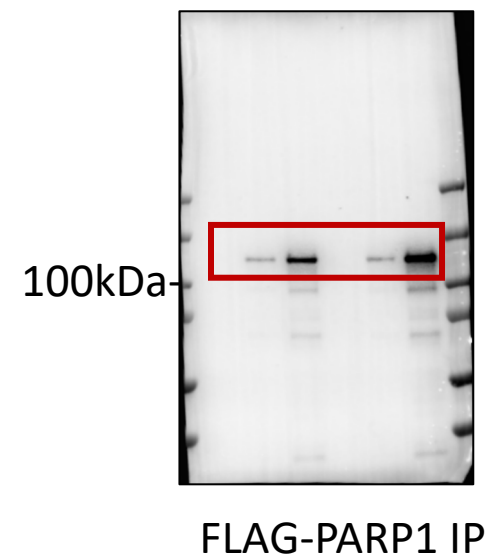

Fig 3E

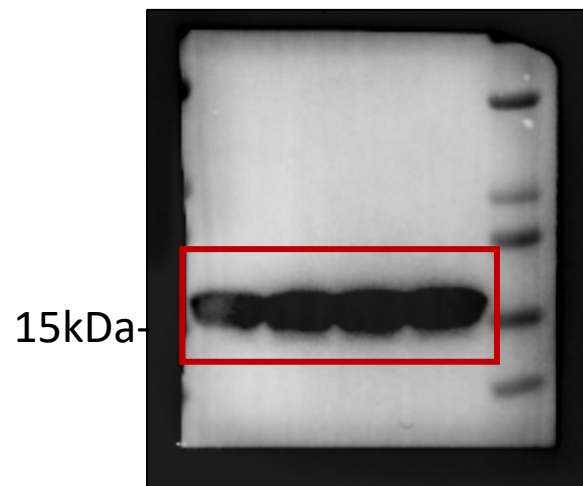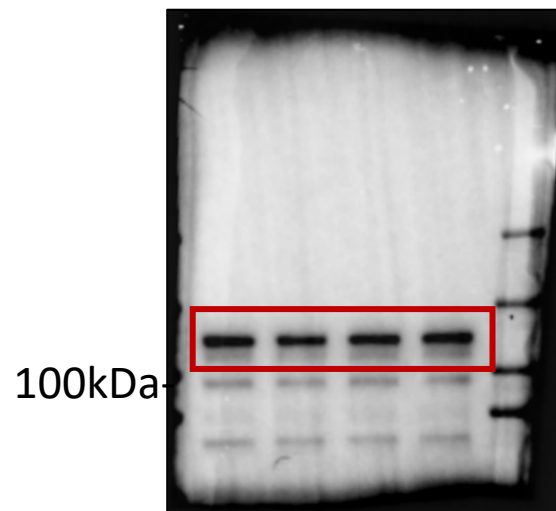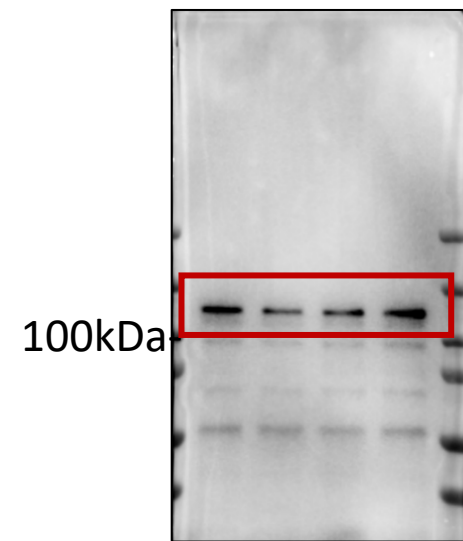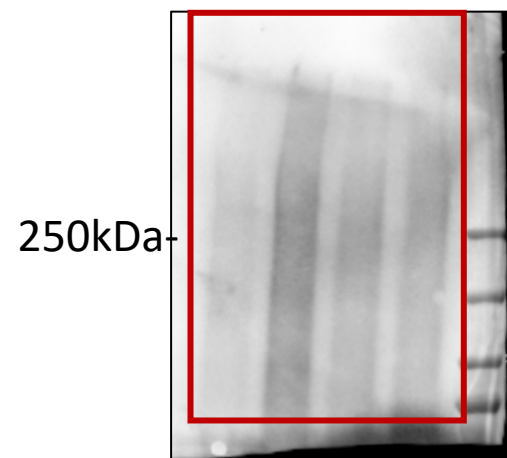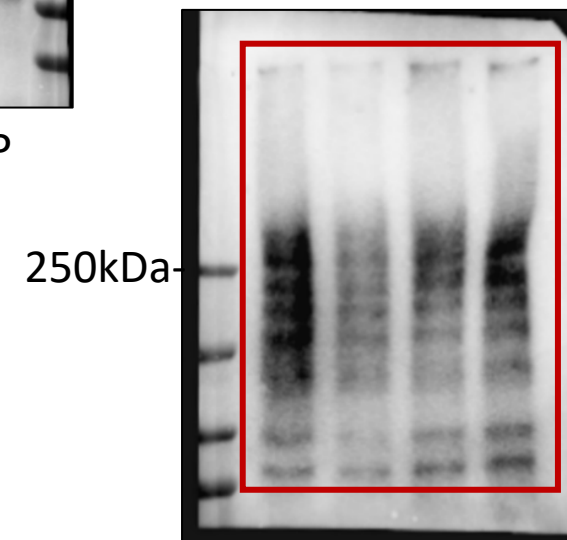

Fig 3G

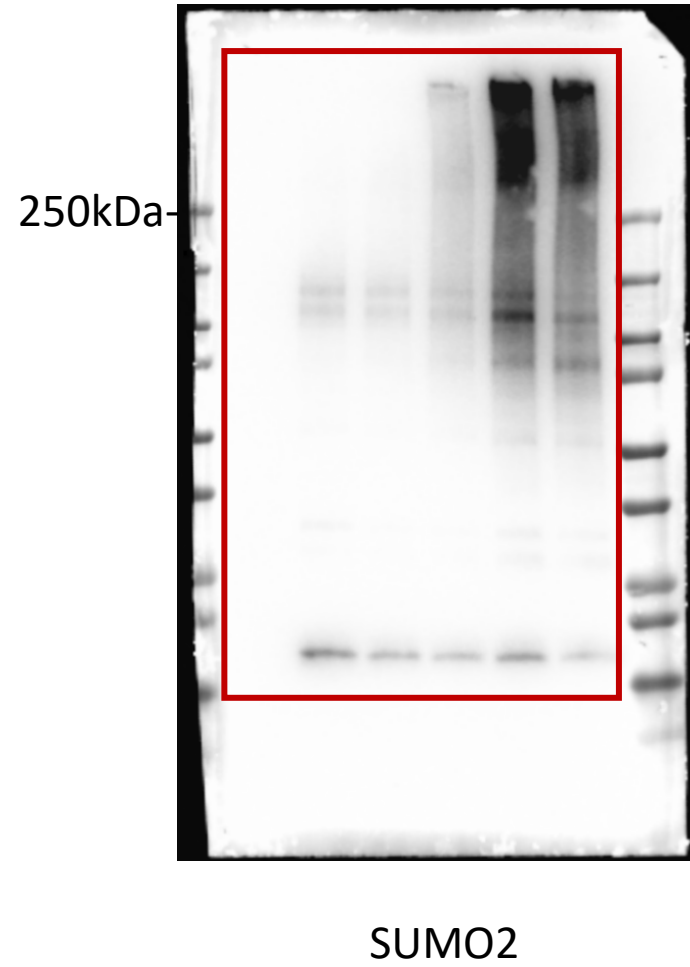

Fig 3H

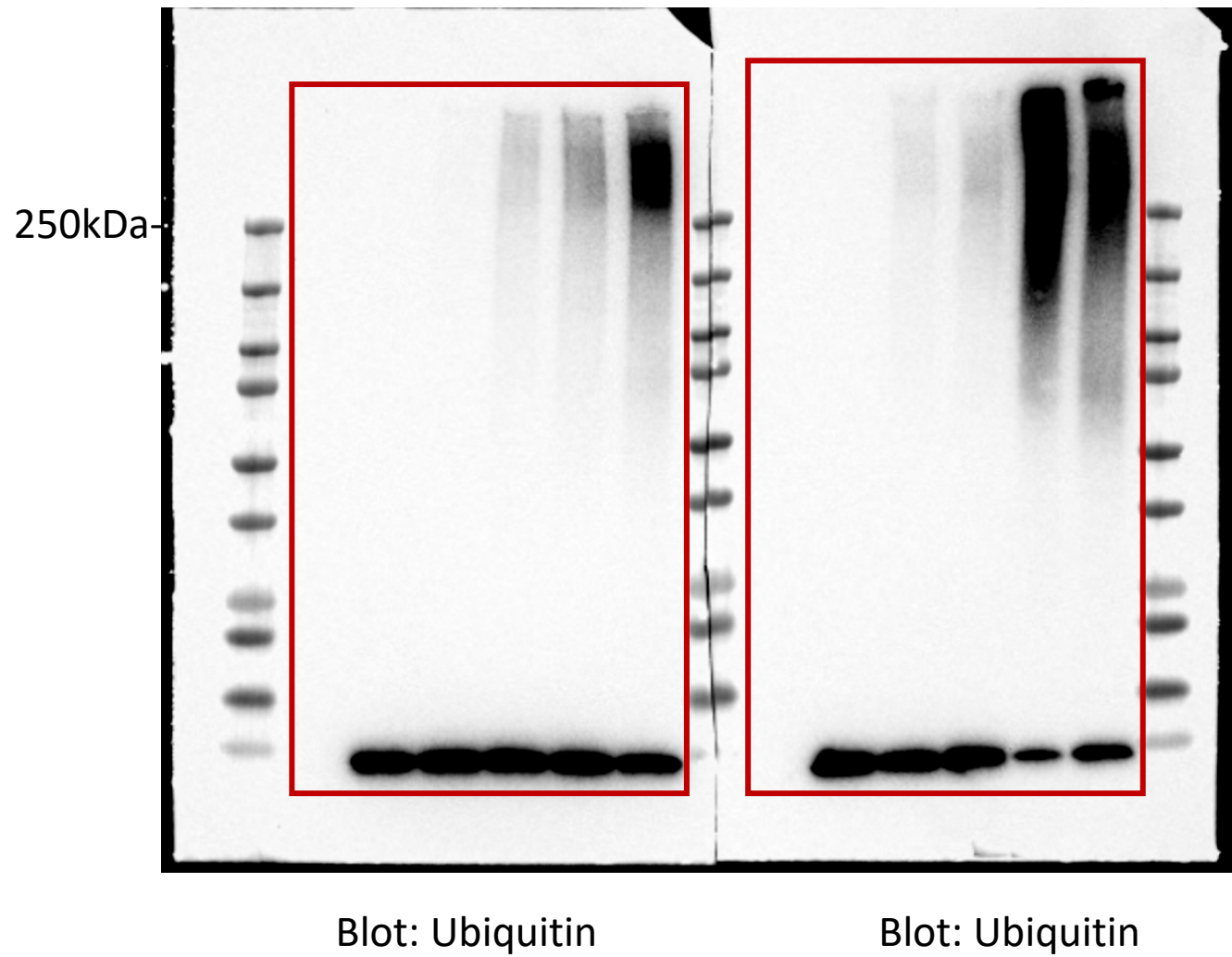

Supplement: Source Data Fig. 3 — Unprocessed western blots and/or gels. [file 41556_2021_807_MOESM7_ESM.pdf]

Fig 5B

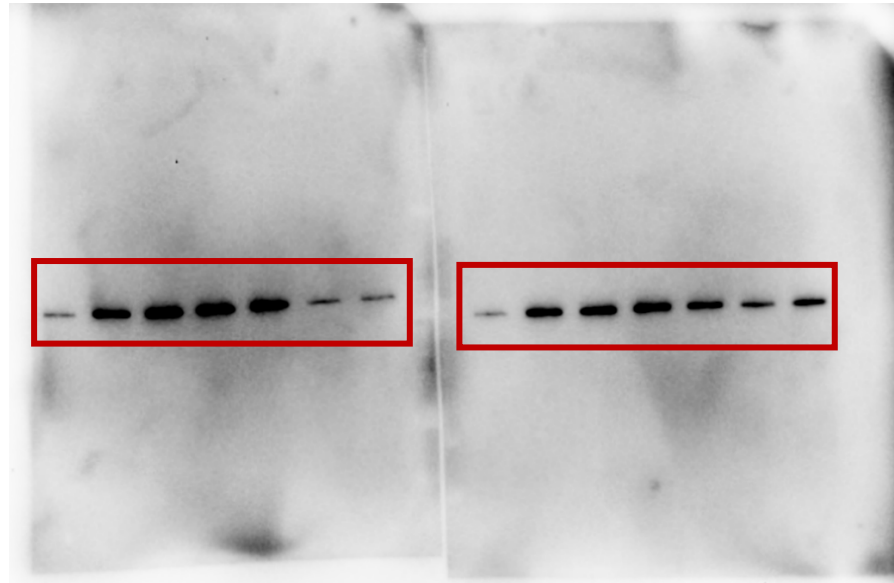

PARP1 (PIAS4 WT)

PARP1 (PIAS4-/-)

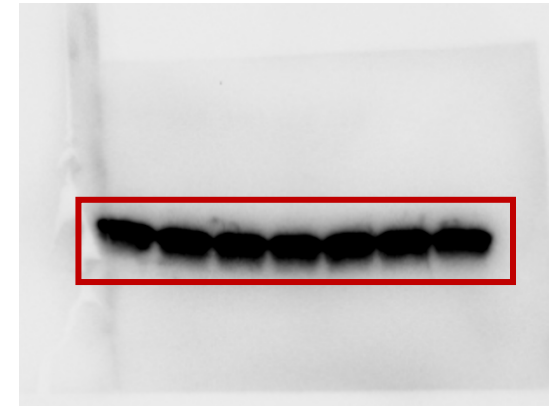

H3 (PIAS4 WT)

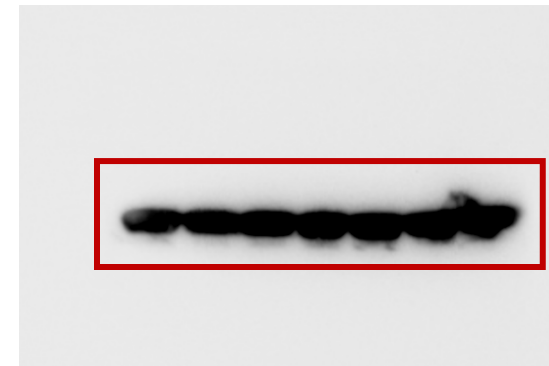

H3 (PIAS4 -/-)

Fig 5C

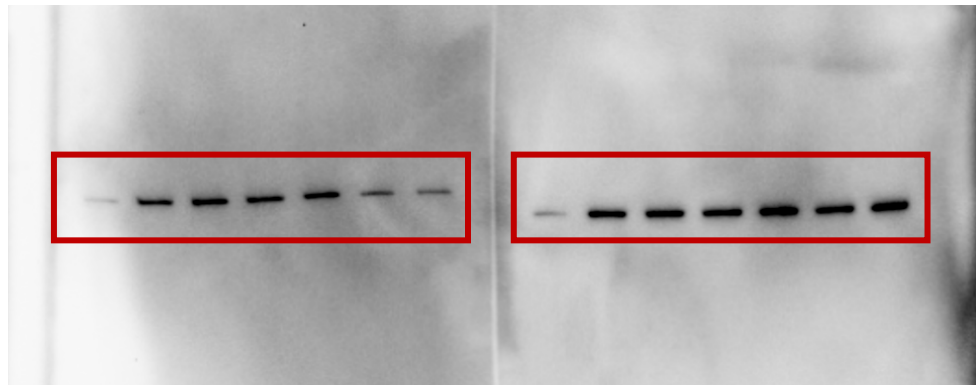

H3 (RNF4 WT)

H3 (RNF4 -/-)

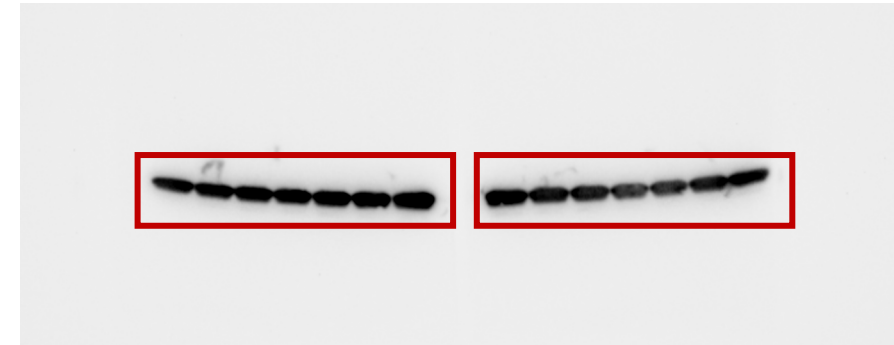

H3 (RNF4 WT)

H3 (RNF4 -/-)

Supplement: Source Data Fig. 5 — Unprocessed western blots and/or gels. [file 41556_2021_807_MOESM11_ESM.pdf]

# Supp Fig 2A

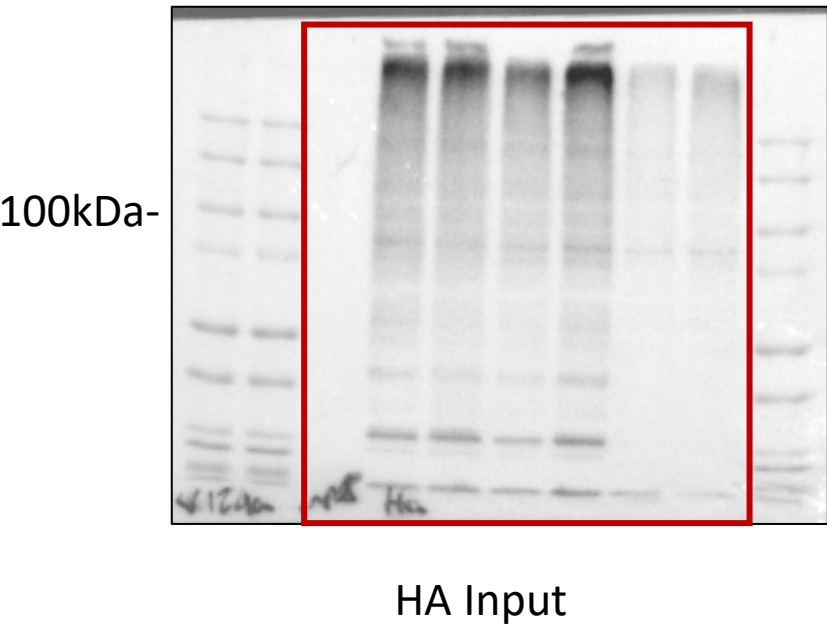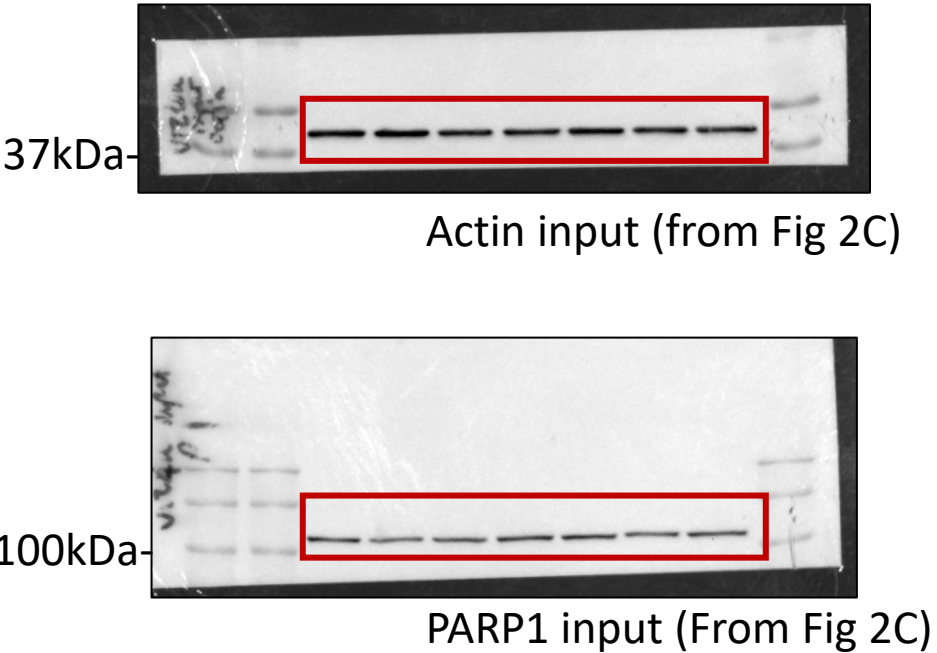

# Supp Fig 2B

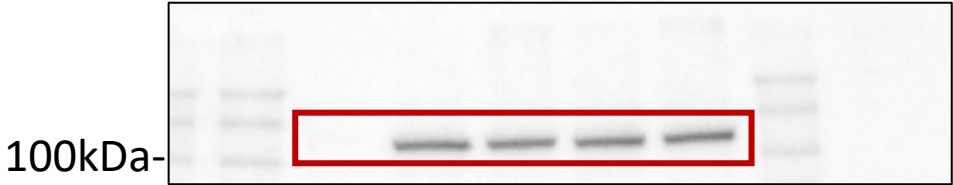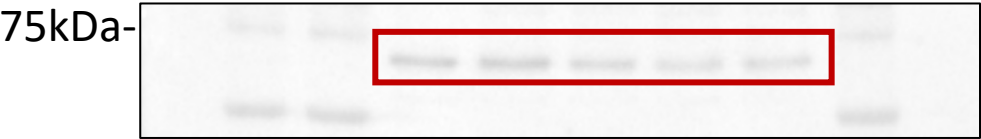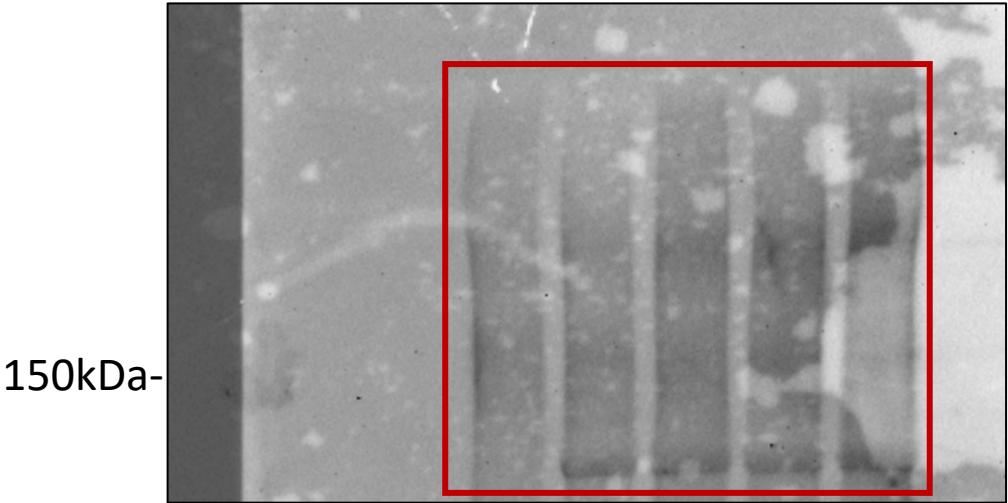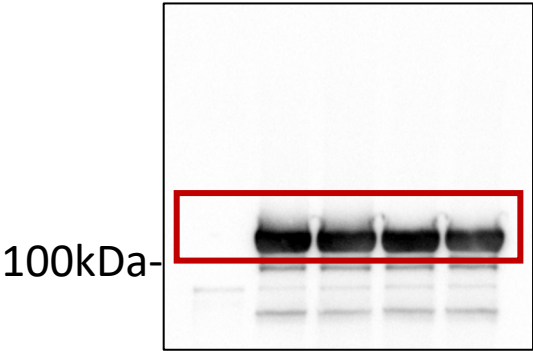

# Supp Fig 2C

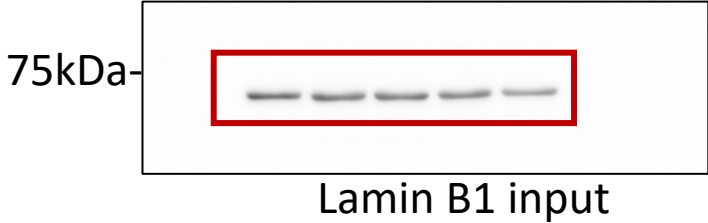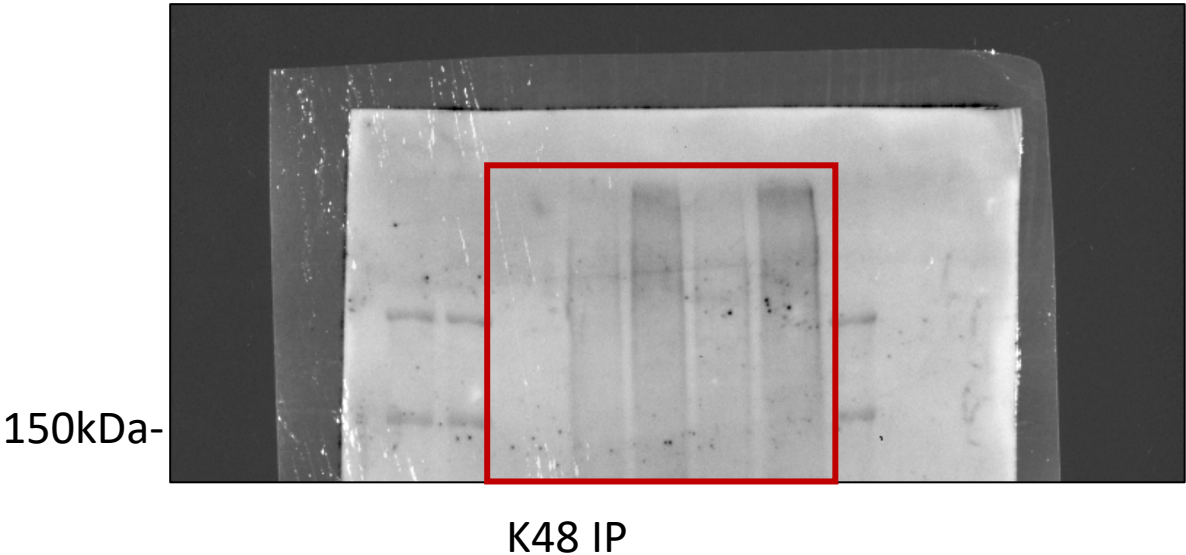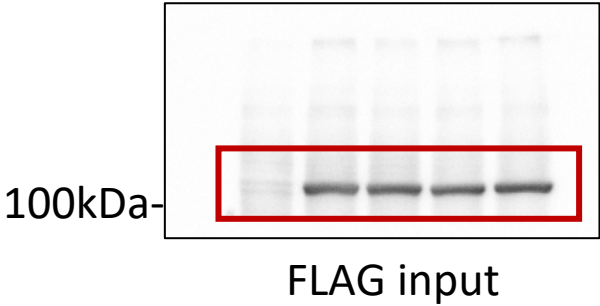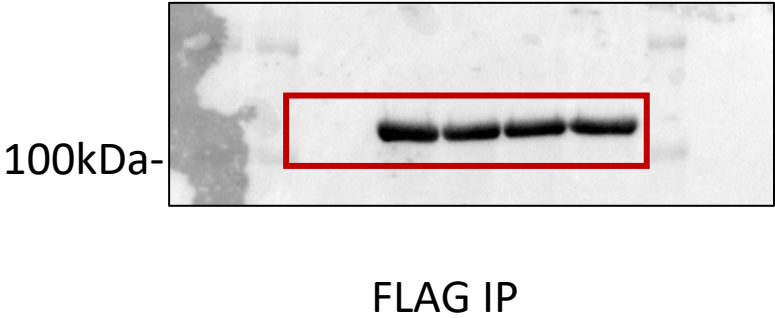

# Supp Fig 2D

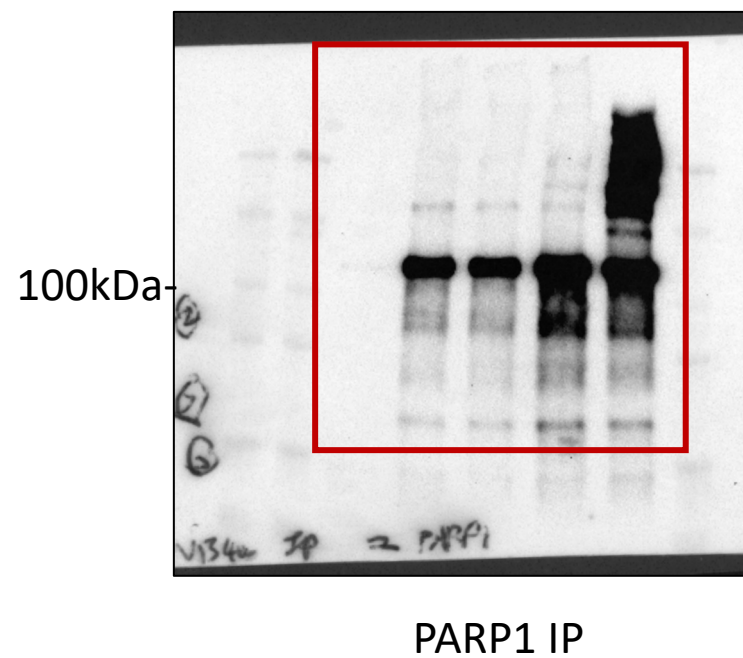

Supplement: Source Data Extended Data Fig. 2 — Unprocessed western blots and/or gels. [file 41556_2021_807_MOESM14_ESM.pdf]

# Supp Fig 3A

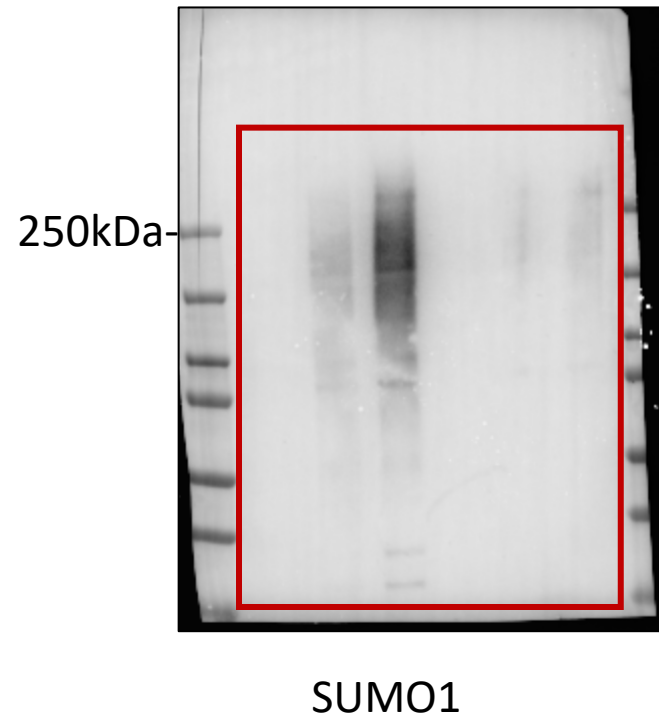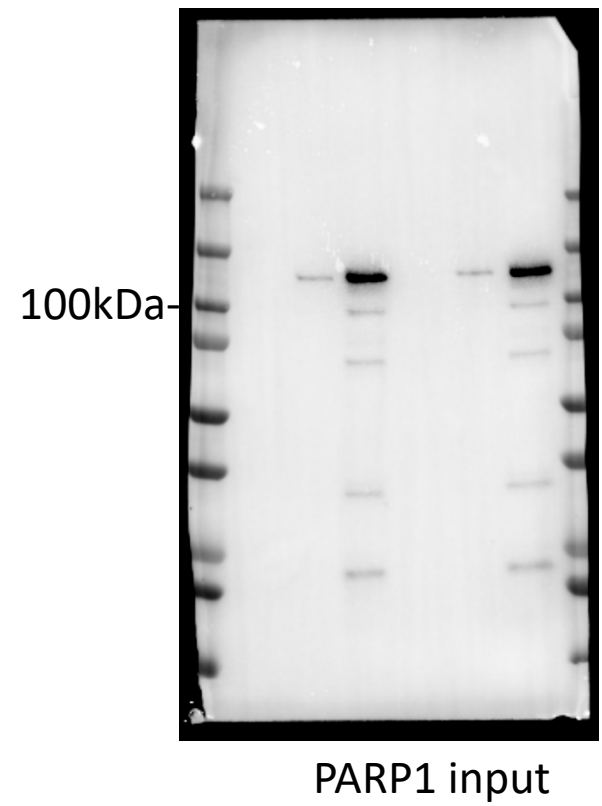

# Supp Fig 3B

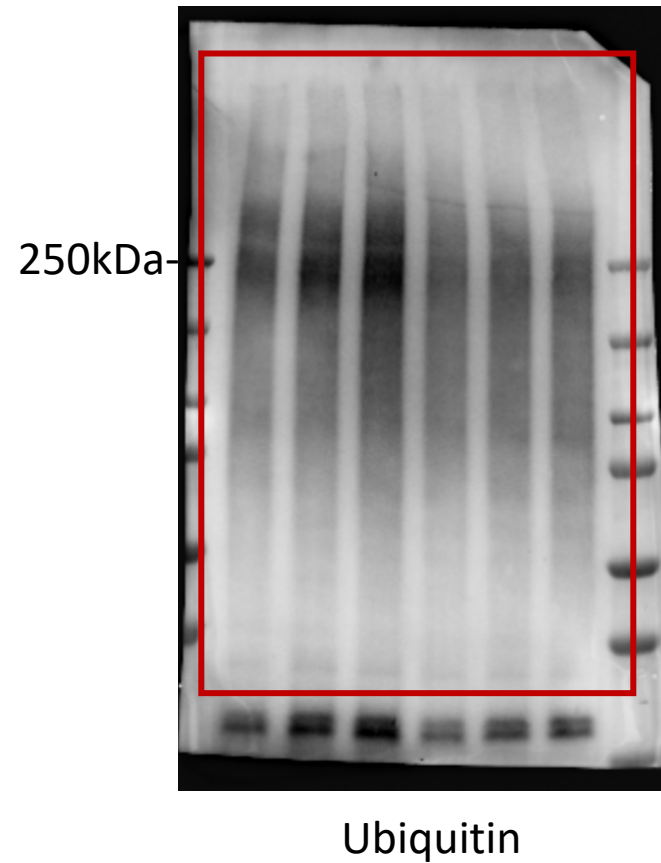

# Supp Fig 3D

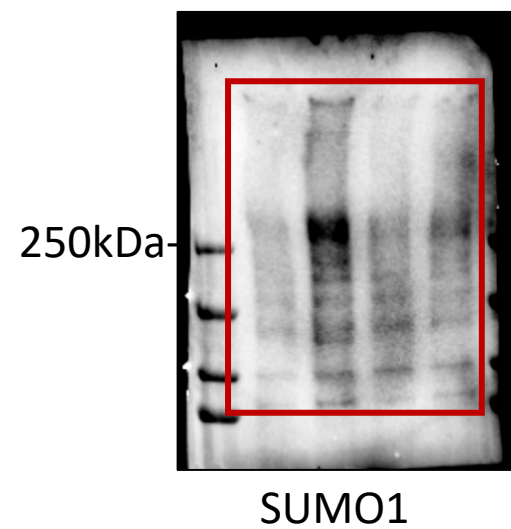

# Supp Fig 3E

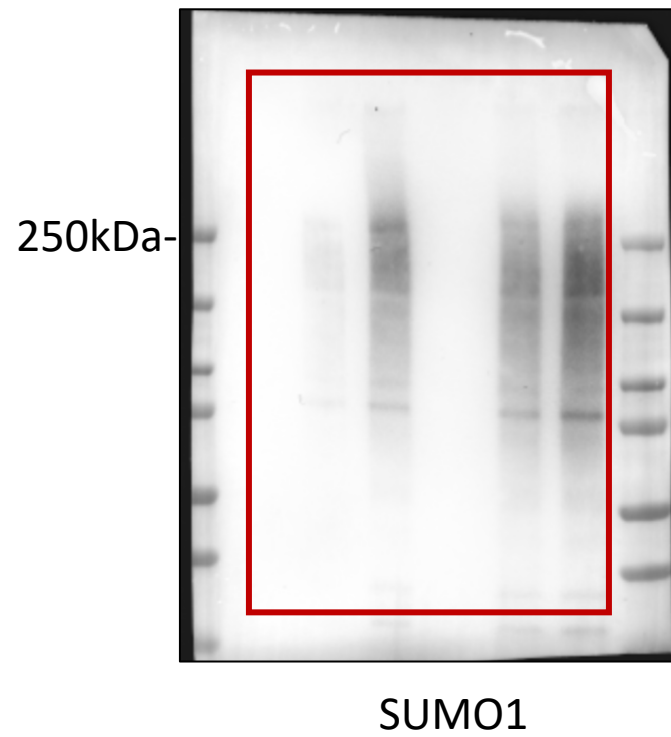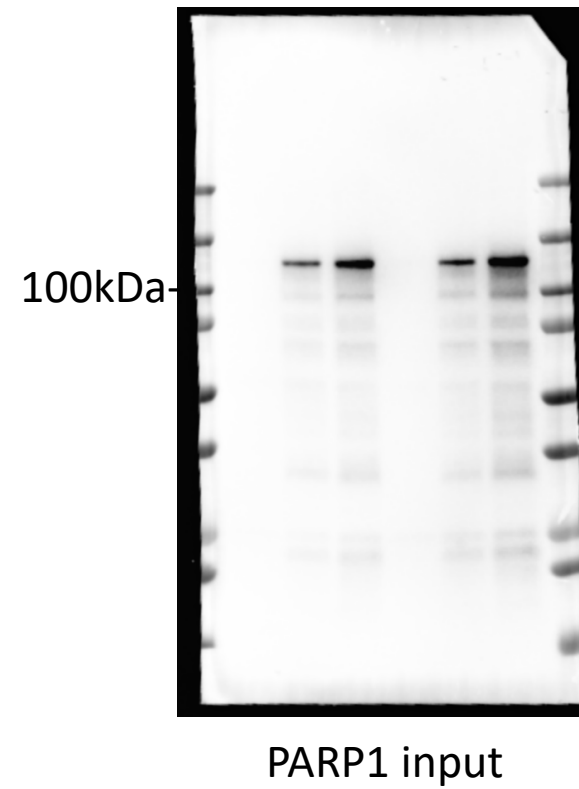

# Supp Fig 3F

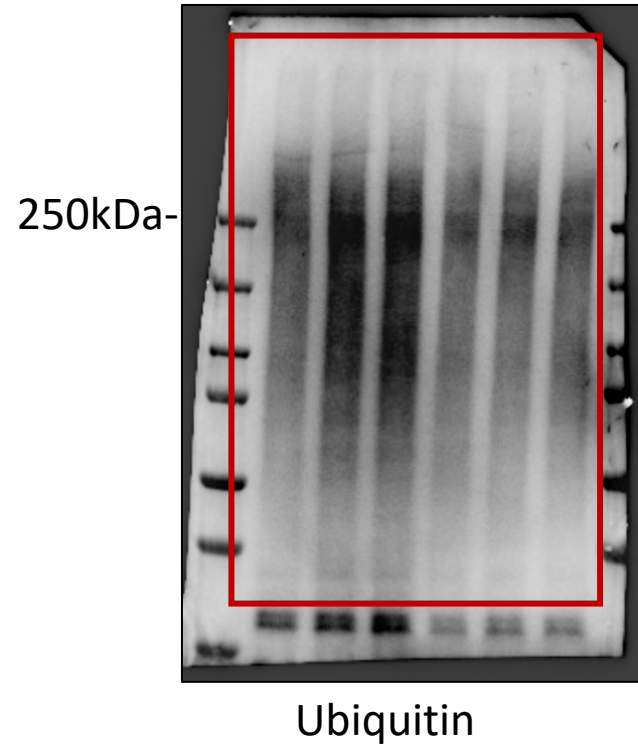

# Supp Fig 3H

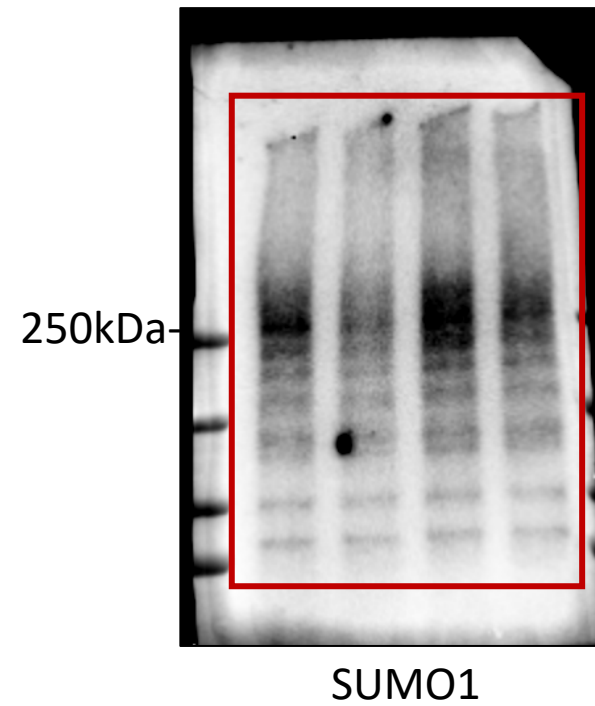

# Supp Fig 3I

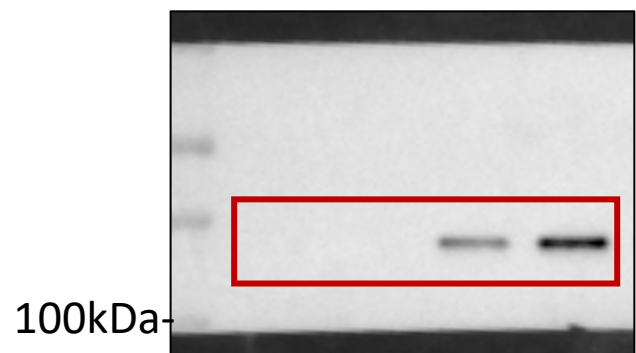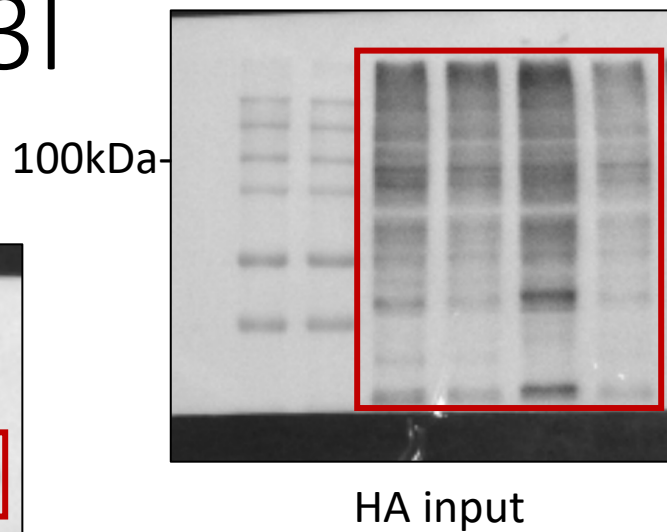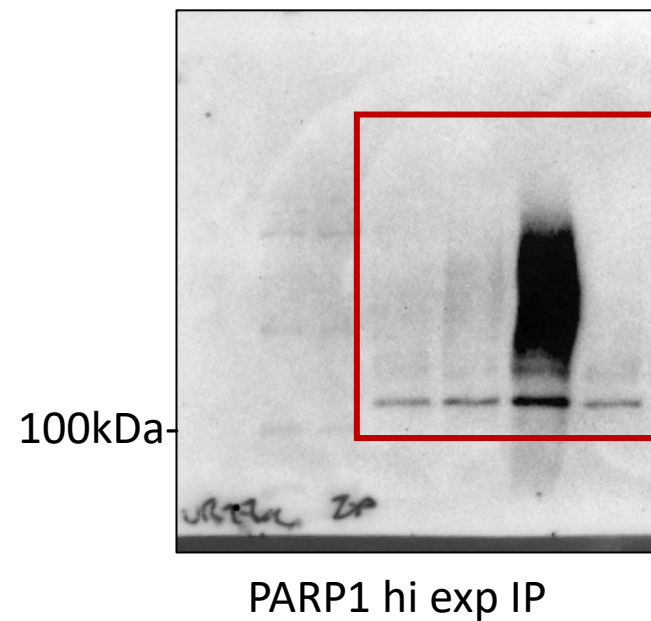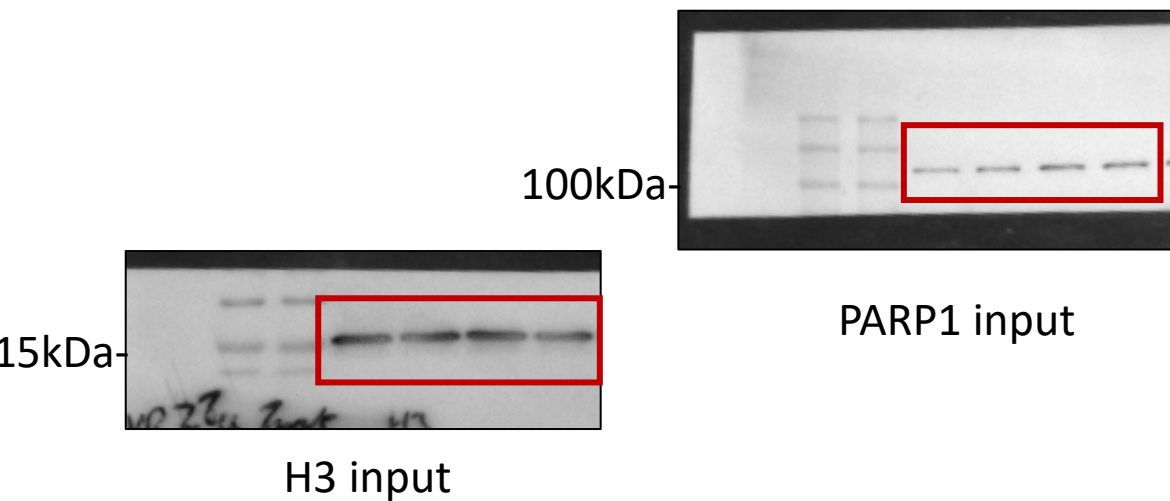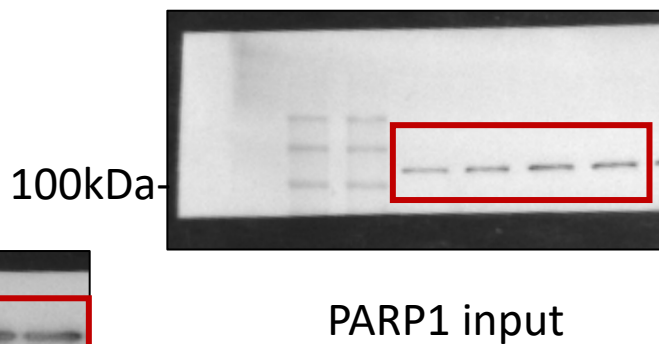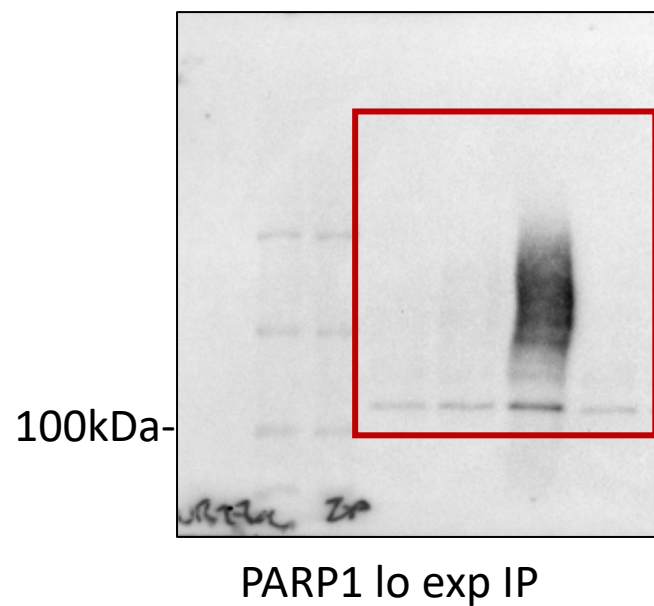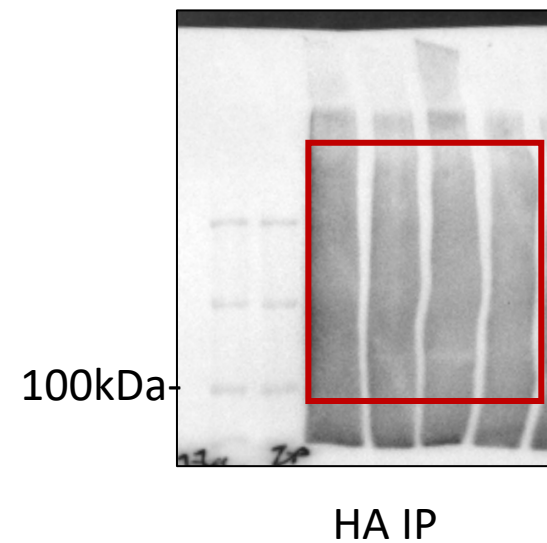

# Supp Fig 3J

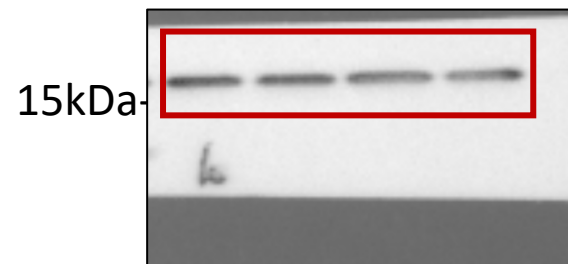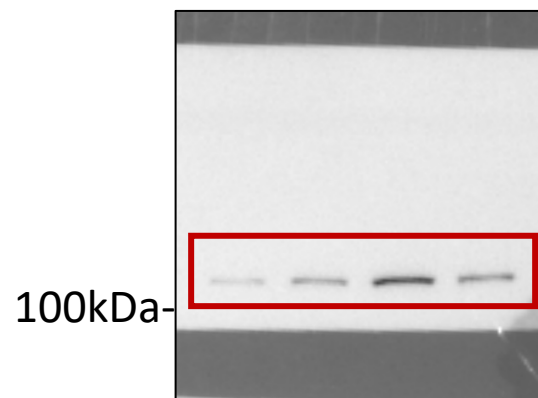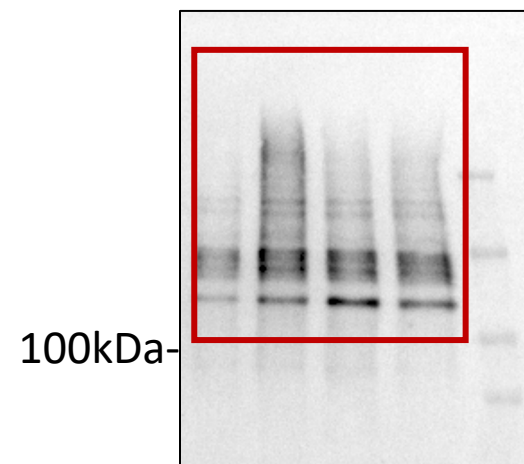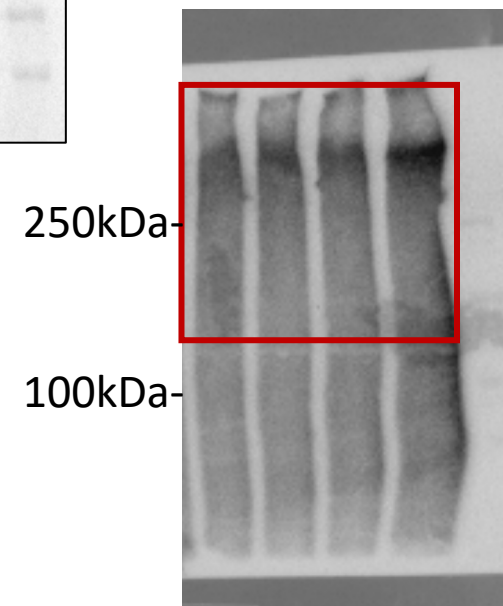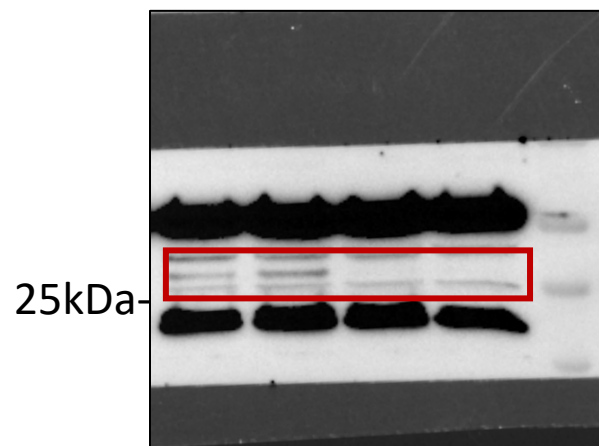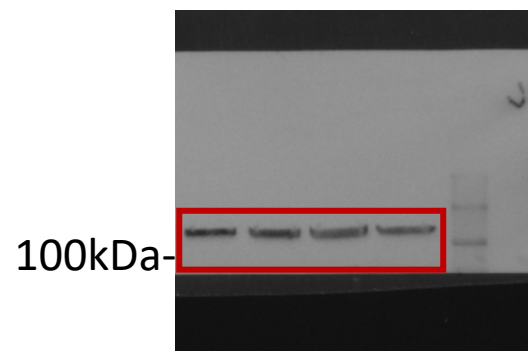

# Supp Fig 3K

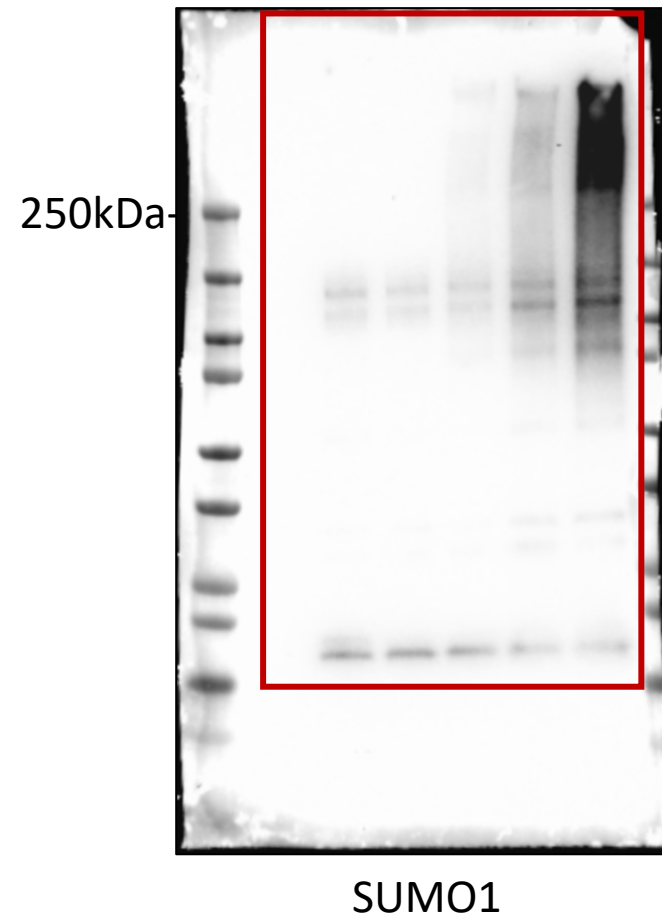

Supplement: Source Data Extended Data Fig. 3 — Unprocessed western blots and/or gels. [file 41556_2021_807_MOESM16_ESM.pdf]

# Supp Fig 4A

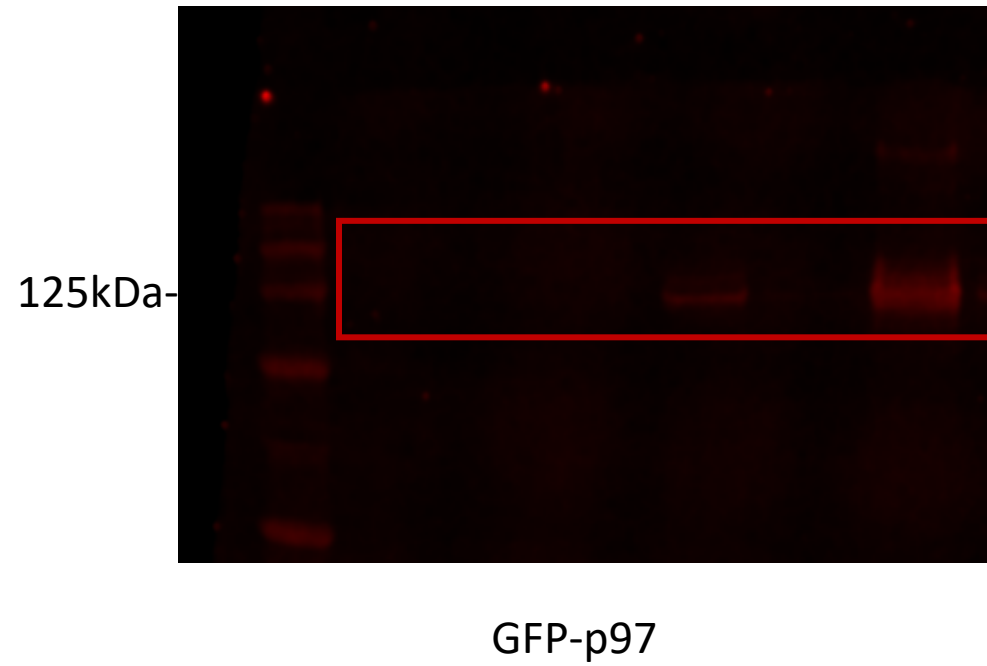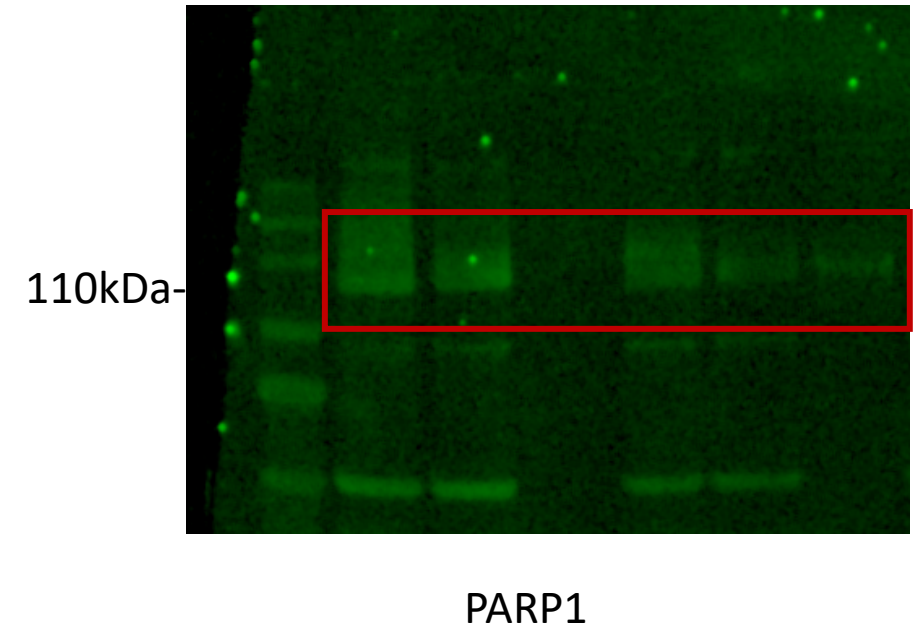

# Supp Fig 4B

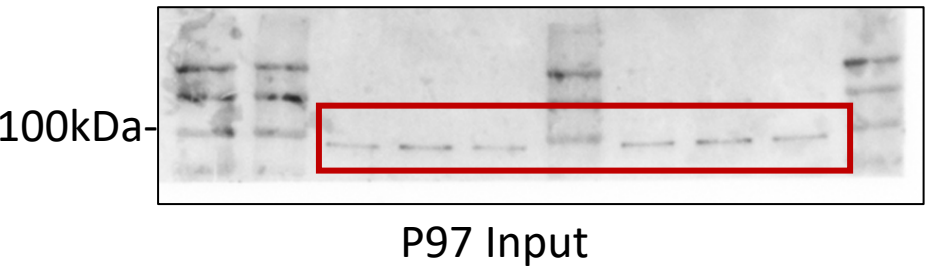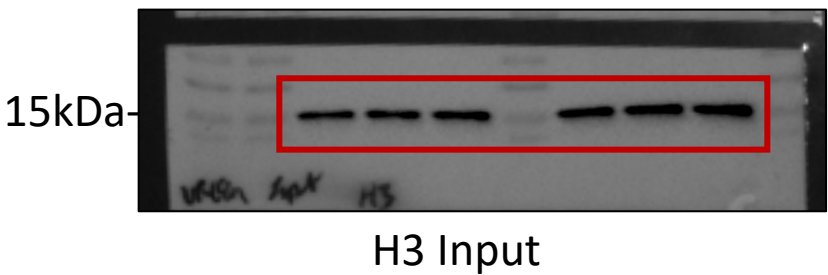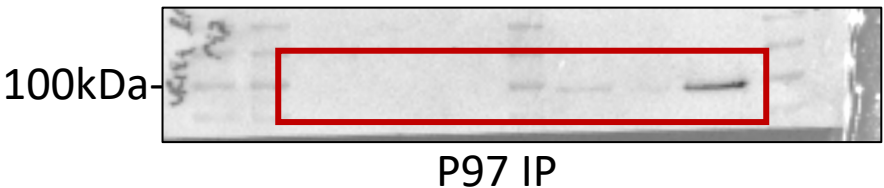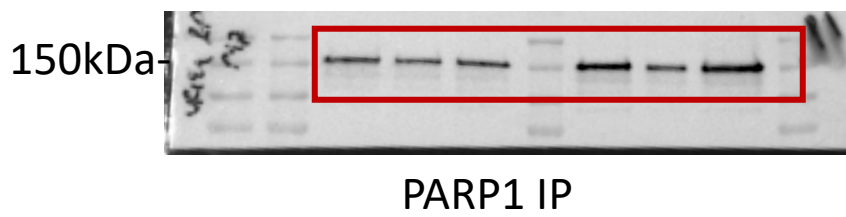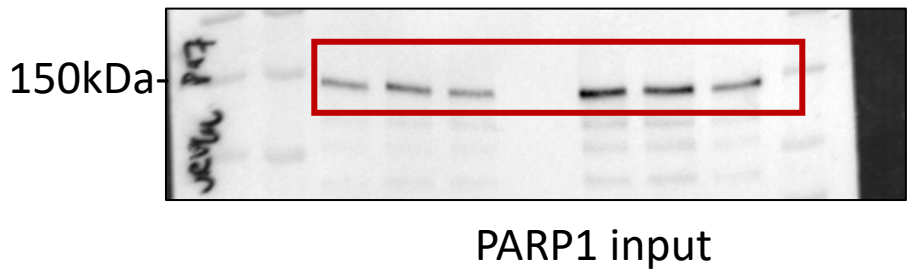

# Supp Fig 4C

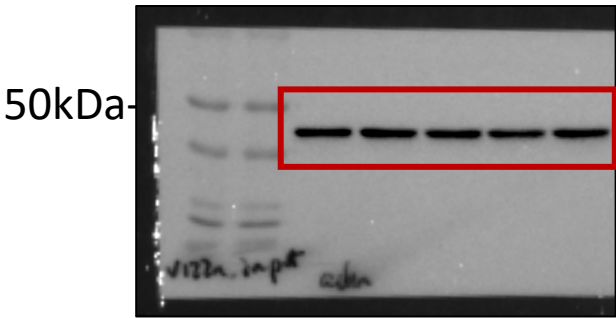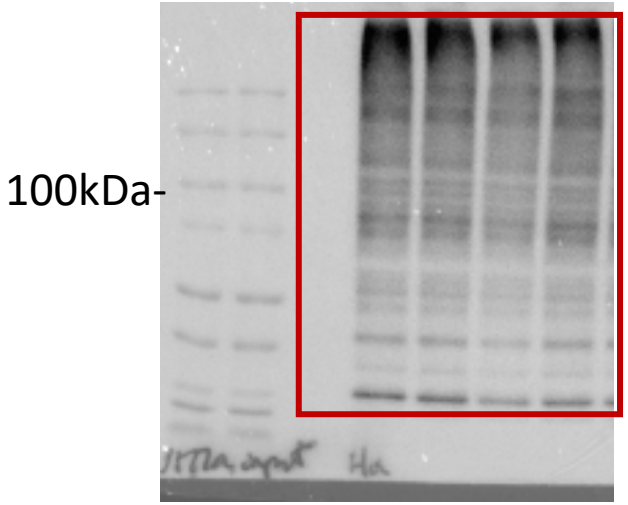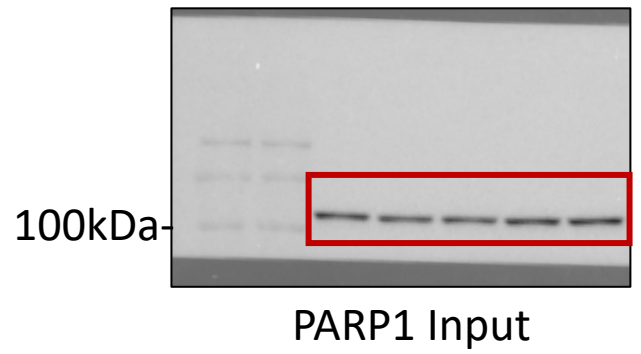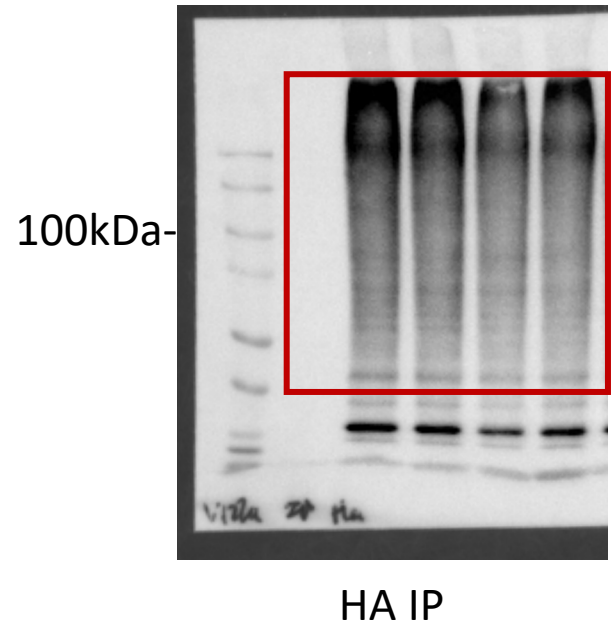

# Supp Fig 4D

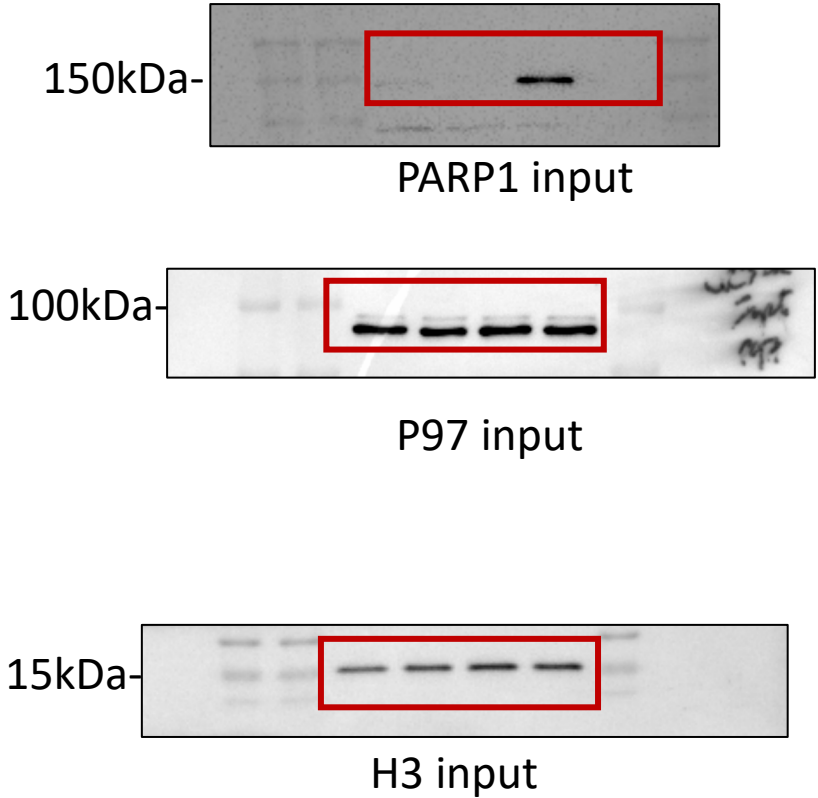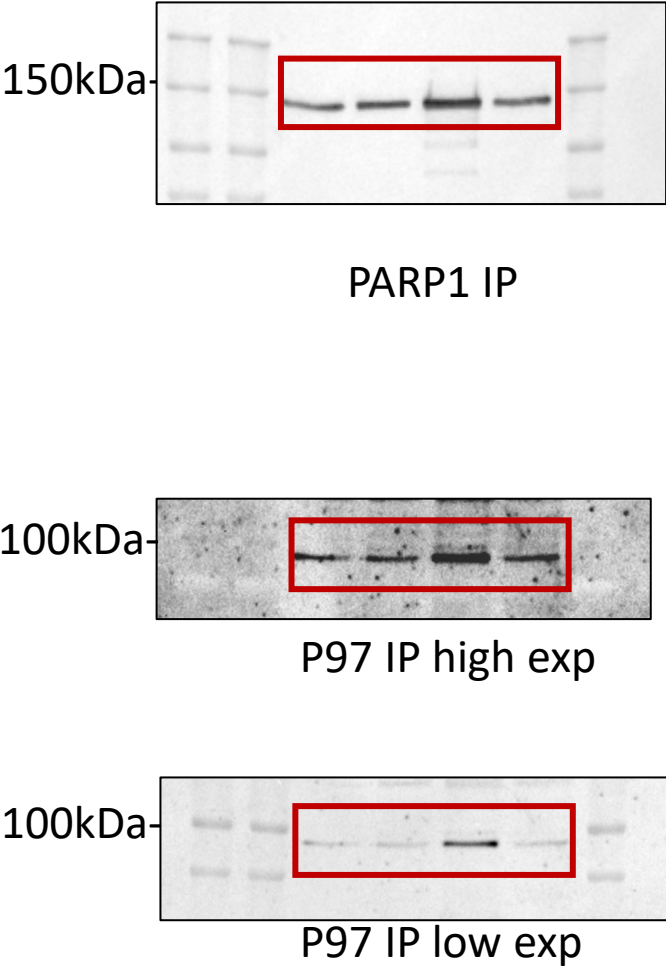

Supplement: Source Data Extended Data Fig. 4 — Unprocessed western blots and/or gels. [file 41556_2021_807_MOESM17_ESM.pdf]
