# Supplementary material for: The ubiquitin-dependent ATPase p97 removes cytotoxic trapped PARP1 from chromatin
Source: Nat Cell Biol. 2022 Jan 10;24(1):62–73. doi: 10.1038/s41556-021-00807-6 (PMC8760077; doi:10.1038/s41556-021-00807-6)
Supplement: Source Data Fig. 4 — Unprocessed western blots and/or gels. [file 41556_2021_807_MOESM9_ESM.pdf]

# Fig 4b

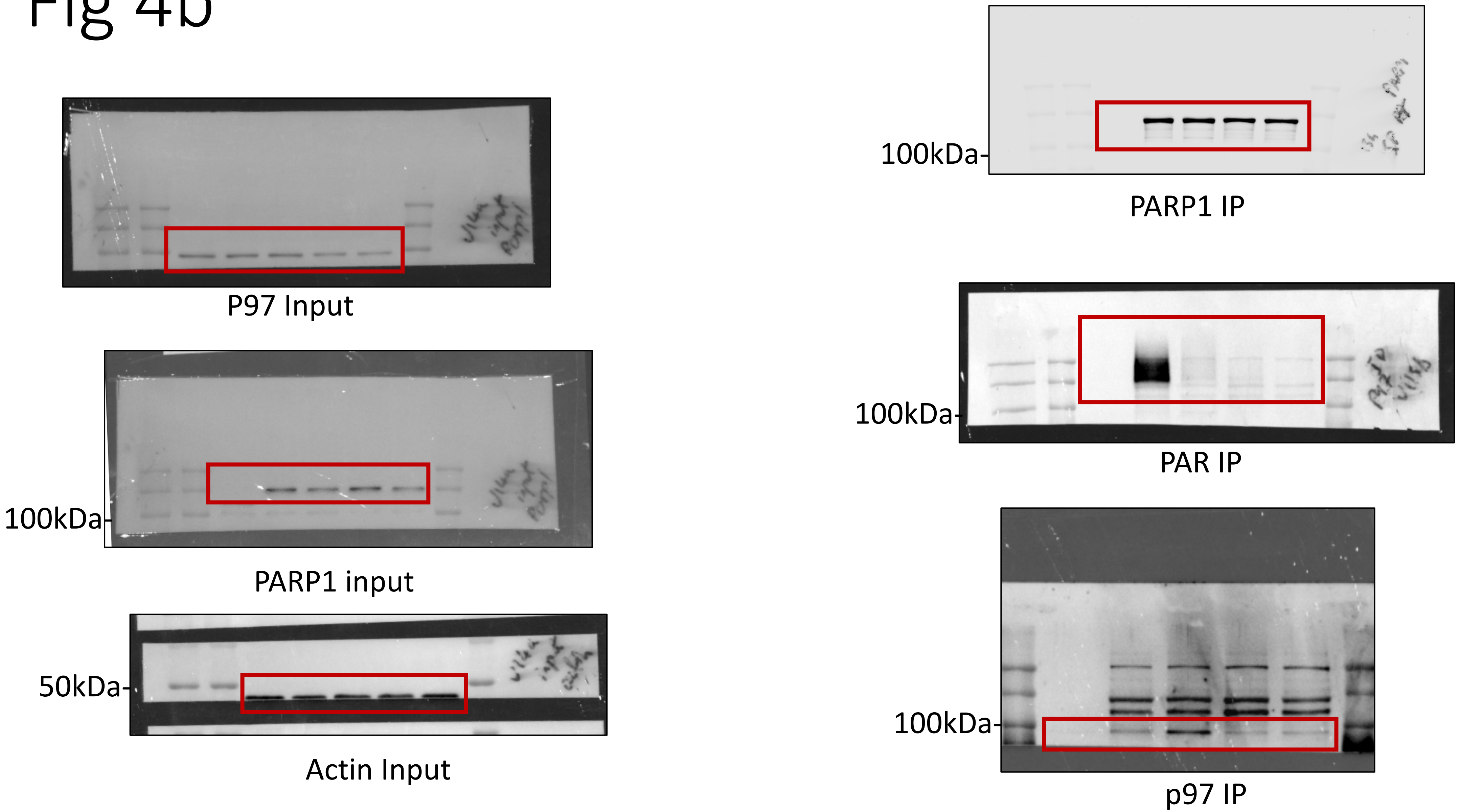
P97 Input
100kDa
PARP1 input
50kDa
Actin Input
100kDa
PARP1 IP
100kDa
PAR IP
100kDa
p97 IP

# Fig 4F

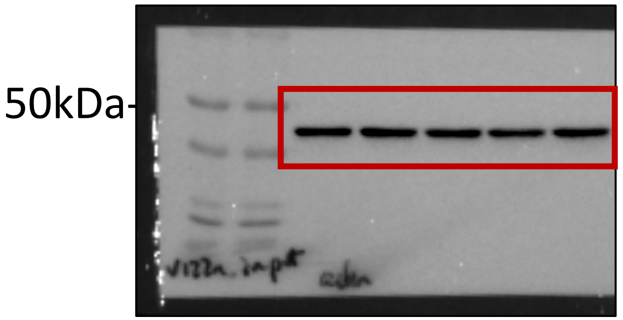


Actin Input

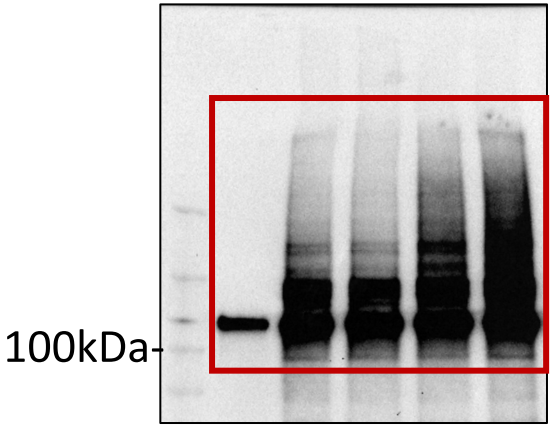


PARP1 IP

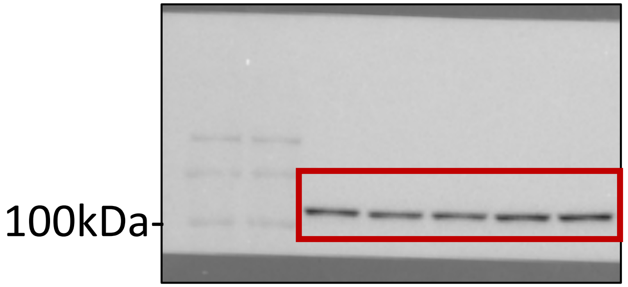


PARP1 Input

# Fig 4G

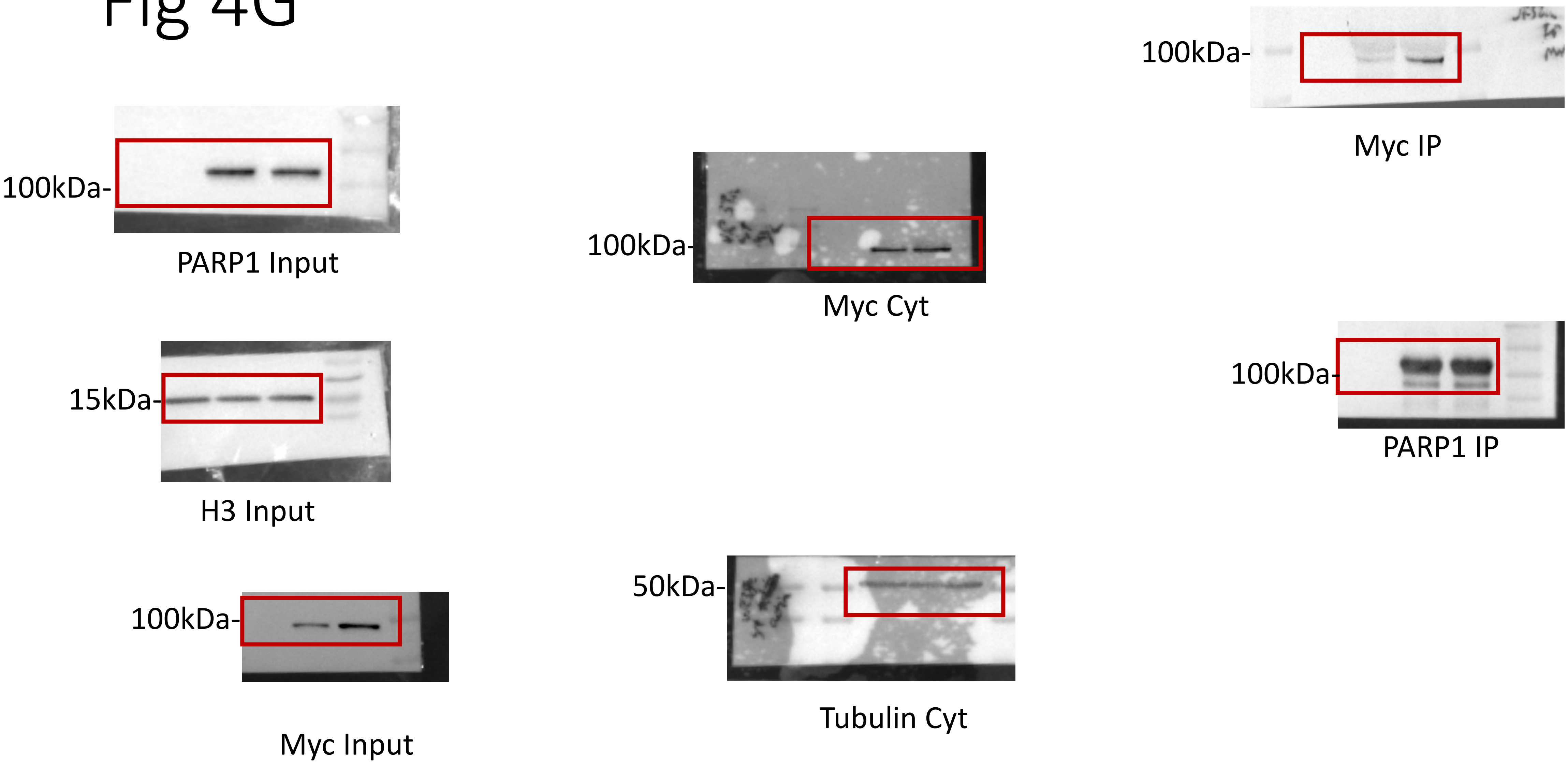
100kDa-
Myc IP
100kDa-
PARP1 Input
100kDa-
Myc Cyt
100kDa-
PARP1 IP
15kDa-
H3 Input
50kDa-
100kDa-
Tubulin Cyt
Myc Input

# Fig 4H

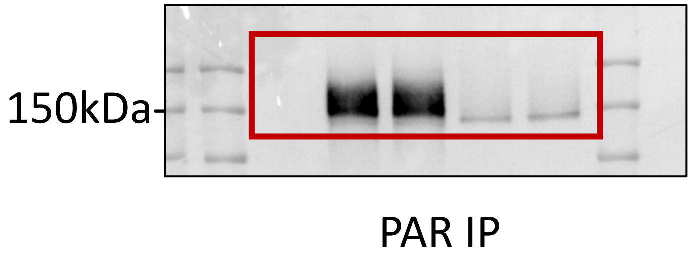

150kDa-
PAR IP


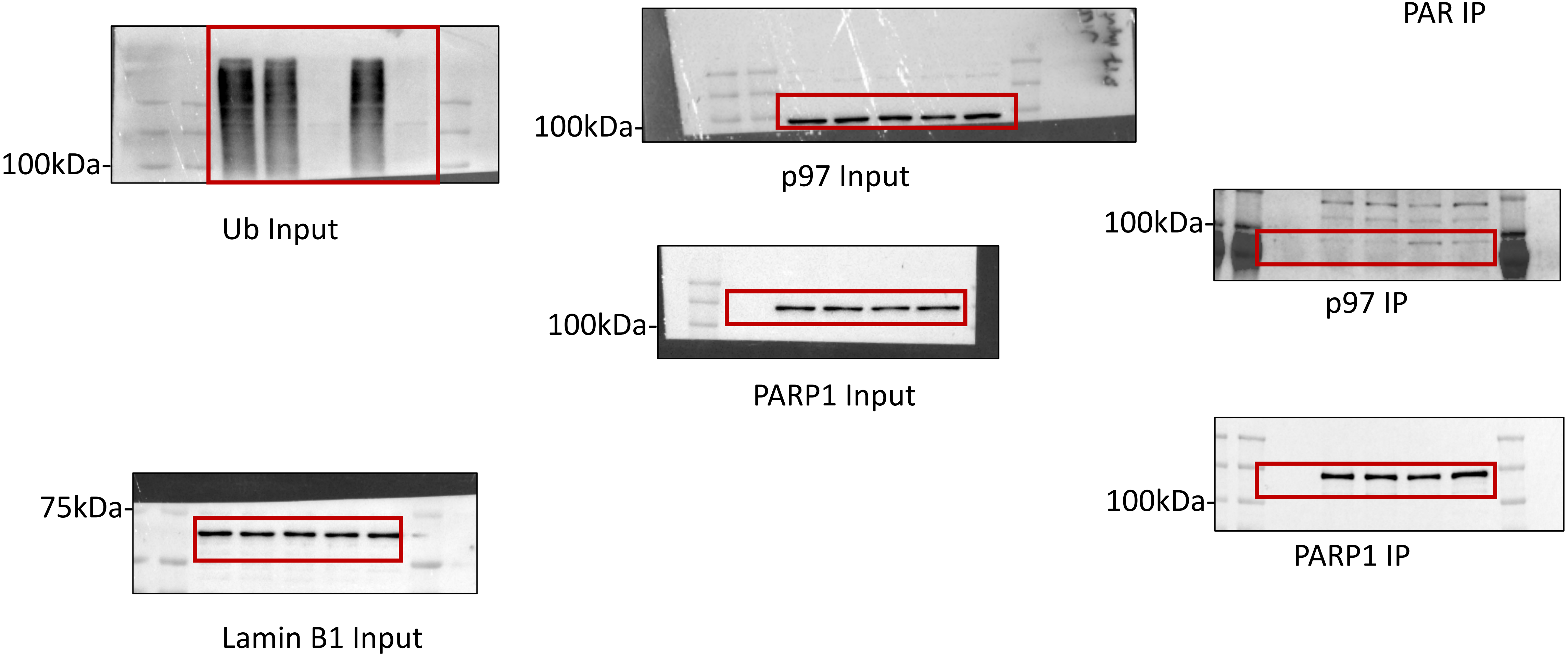

100kDa-
Ub Input
100kDa-
p97 Input
100kDa-
p97 IP
100kDa-
PARP1 Input
100kDa-
PARP1 IP
75kDa-
Lamin B1 Input

# Fig 4J

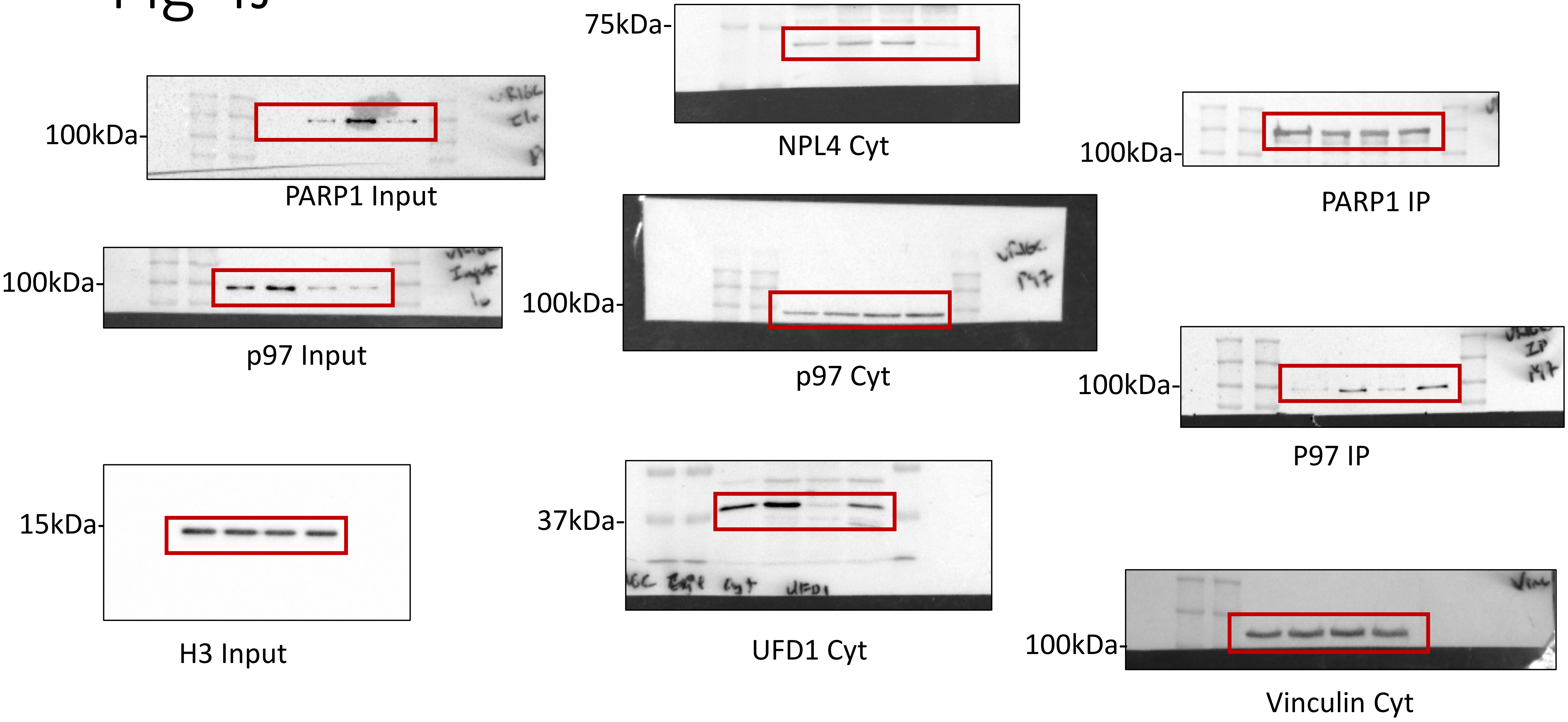

75kDa-
NPL4 Cyt
100kDa-
PARP1 Input
100kDa-
PARP1 IP
100kDa-
p97 Input
100kDa-
p97 Cyt
100kDa-
P97 IP
15kDa-
H3 Input
37kDa-
UFD1 Cyt
100kDa-
Vinculin Cyt

# Fig 4K

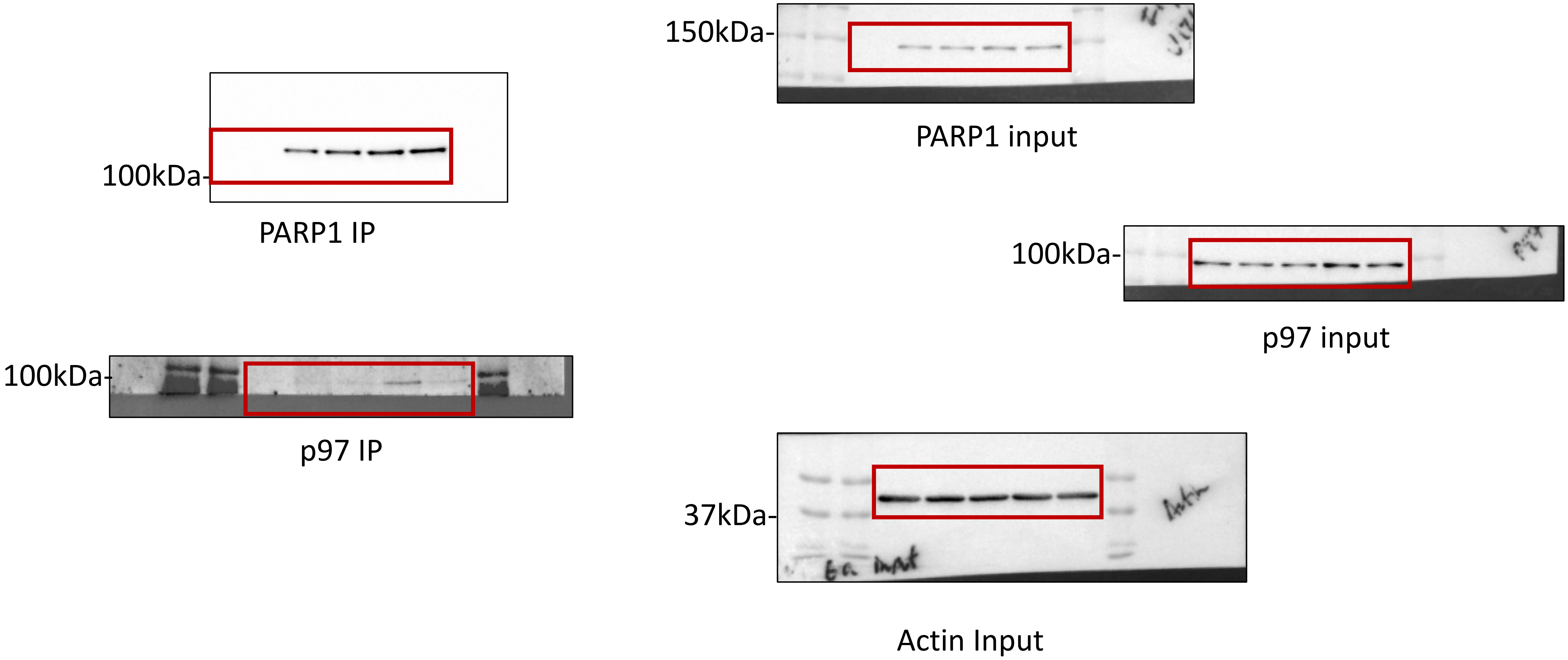
100kDa-
PARP1 IP
100kDa-
p97 IP
150kDa-
PARP1 input
100kDa-
p97 input
37kDa-
Actin Input
