# Supplementary material for: The ubiquitin-dependent ATPase p97 removes cytotoxic trapped PARP1 from chromatin
Source: Nat Cell Biol. 2022 Jan 10;24(1):62–73. doi: 10.1038/s41556-021-00807-6 (PMC8760077; doi:10.1038/s41556-021-00807-6)

Supp Fig 1A

100kDa-

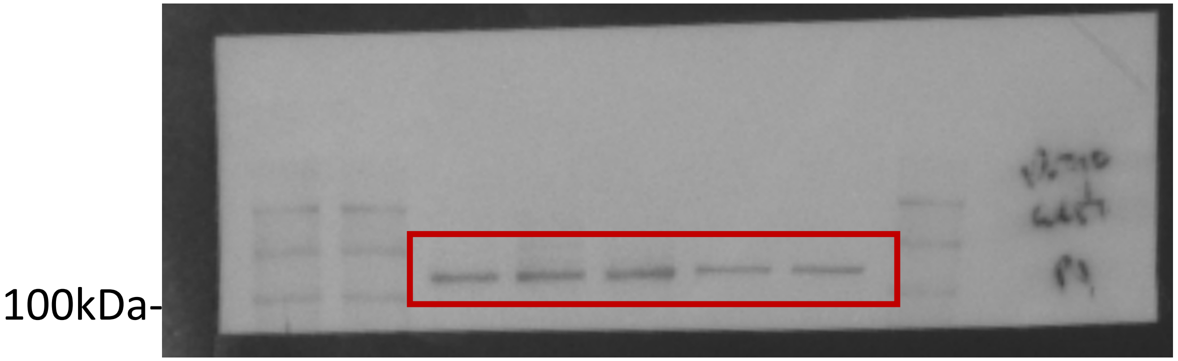

Vinculin

100kDa-

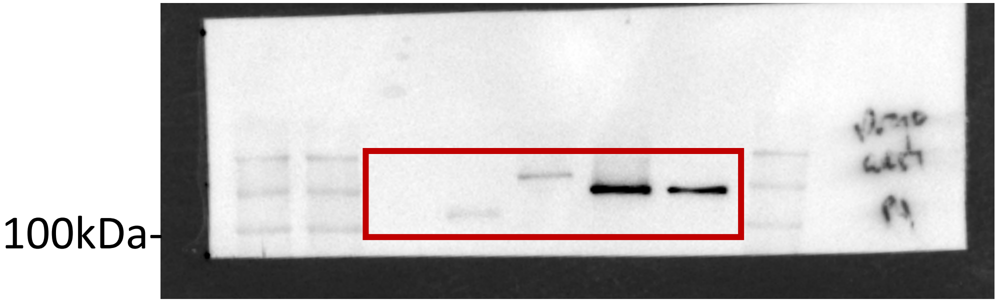

PARP1

Supp Fig 1B

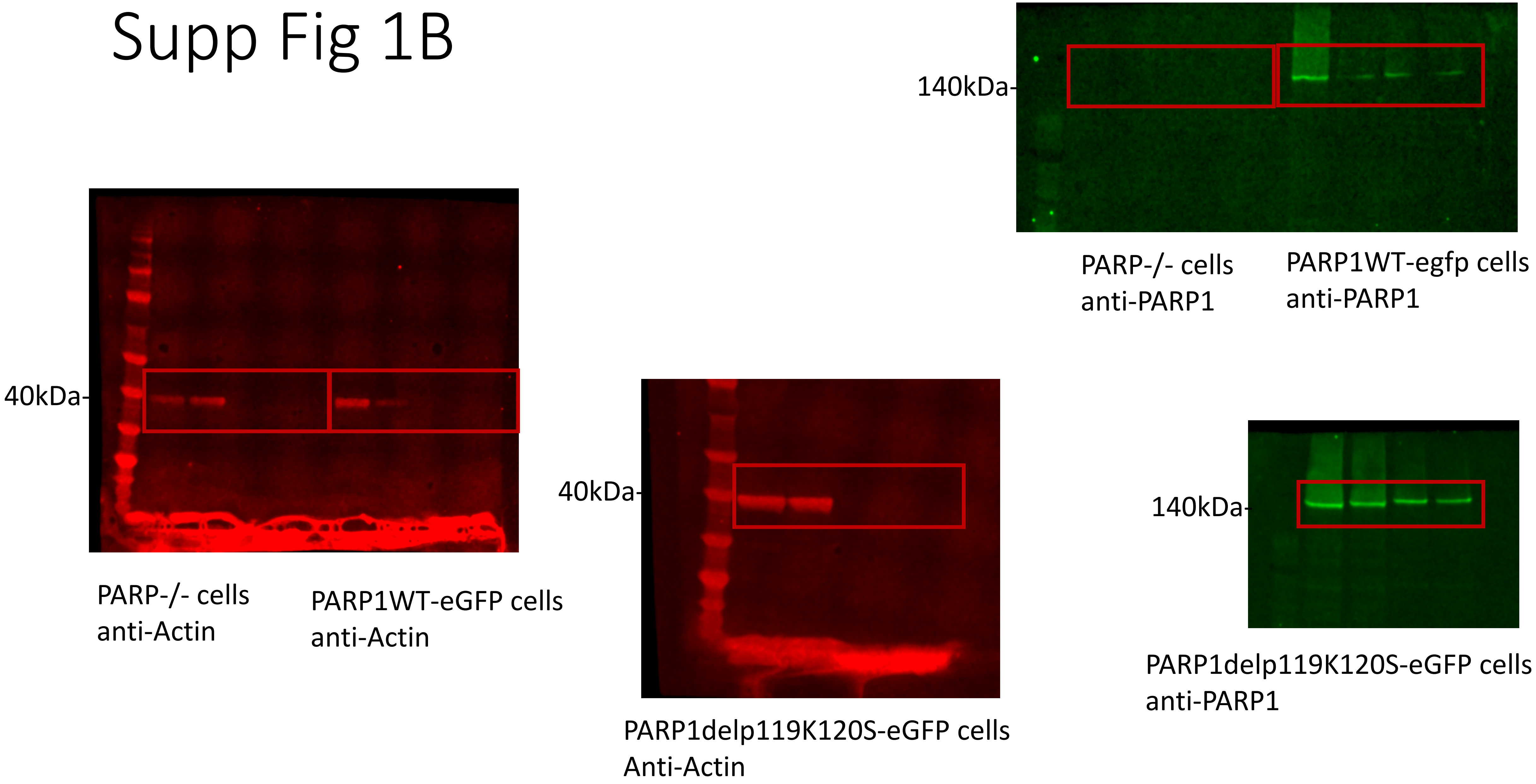
40kDa
PARP-/- cells
anti-Actin
PARP1WT-eGFP cells
anti-Actin
40kDa
PARP1delp119K120S-eGFP cells
Anti-Actin
140kDa
PARP-/- cells
anti-PARP1
PARP1WT-egfp cells
anti-PARP1
140kDa
PARP1delp119K120S-eGFP cells
anti-PARP1

Supp Fig 1C

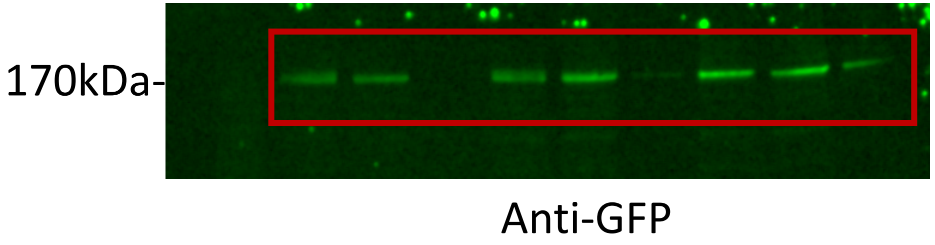

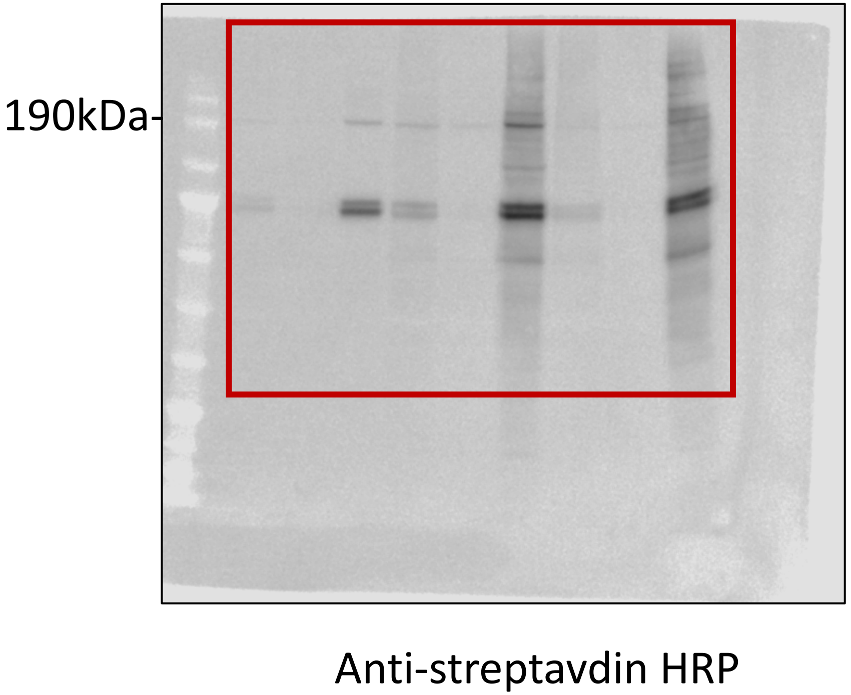

Supplement: Source Data Extended Data Fig. 1 — Unprocessed western blots and/or gels. [file 41556_2021_807_MOESM13_ESM.pdf]
