# Supplementary material for: The ubiquitin-dependent ATPase p97 removes cytotoxic trapped PARP1 from chromatin
Source: Nat Cell Biol. 2022 Jan 10;24(1):62–73. doi: 10.1038/s41556-021-00807-6 (PMC8760077; doi:10.1038/s41556-021-00807-6)
Supplement: Source Data Extended Data Fig. 5 — Unprocessed western blots and/or gels. [file 41556_2021_807_MOESM19_ESM.pdf]

Supp Fig 5C

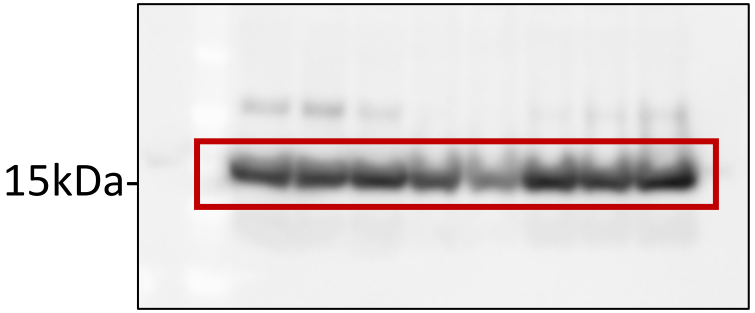

H2AX

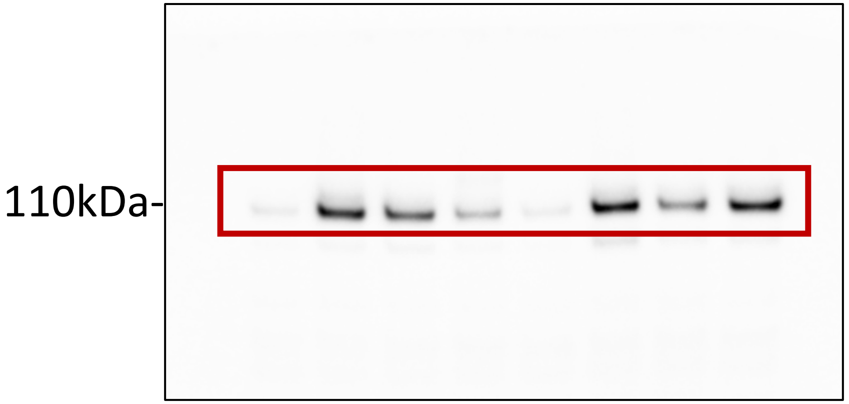

PARP1

Supp Fig 5D

25kDa-

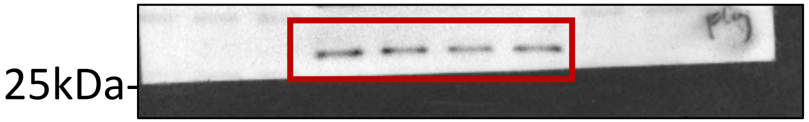

FLAG-RNF4 Cytosol

15kDa-

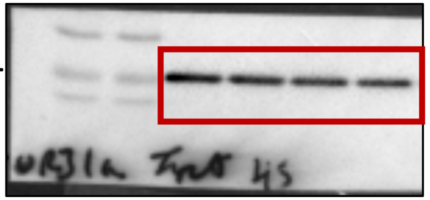

H3 Chr

100kDa-

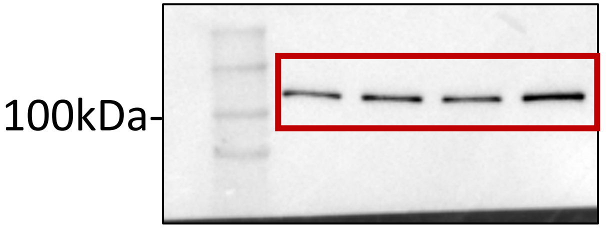

Vinculin Cytosol

150kDa-

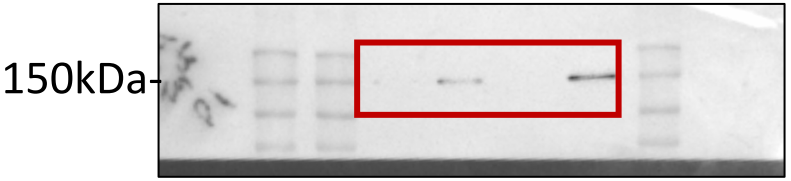

PARP1 Chr

Supp Fig 5E

37kDa-

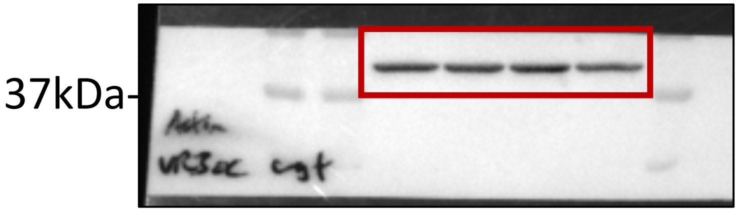

Actin Cyt

100kDa-

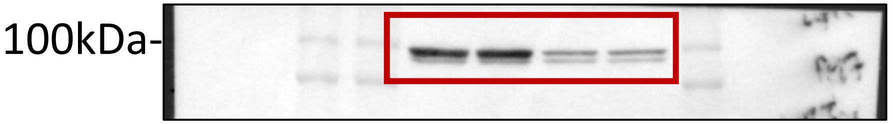

P97 Cyt

100kDa-

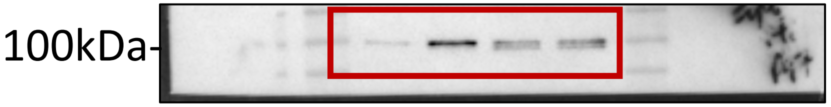

p97 Chr

15kDa-

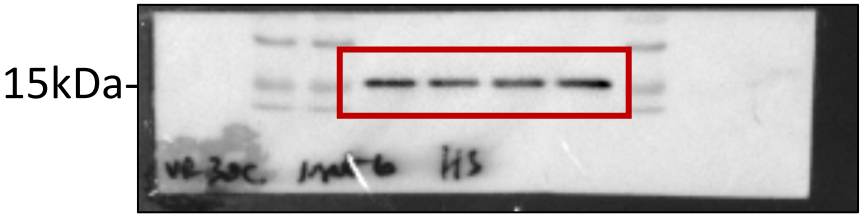

H3 Chr

100kDa-

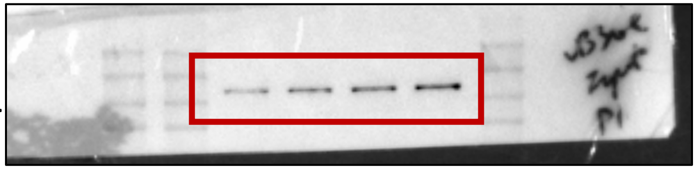

PARP1 Chr

Supp Fig 5H

150kDa-

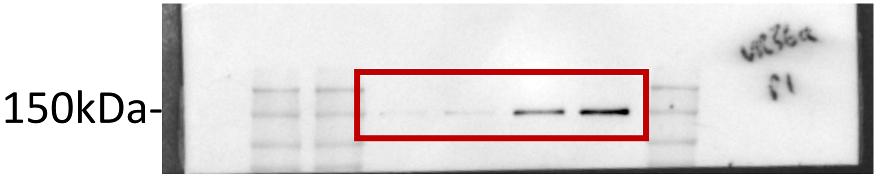

PARP1 input

15kDa-

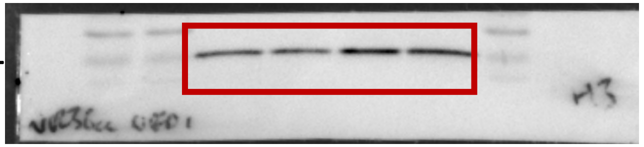

H3 input

37kDa-

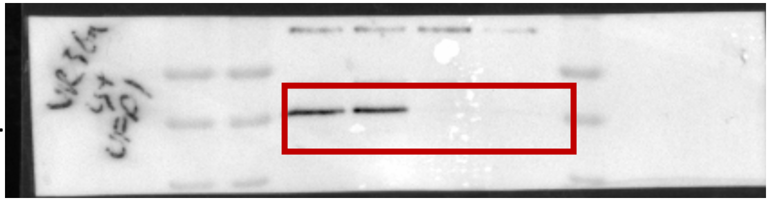

UFD1 Cyt

100kDa-

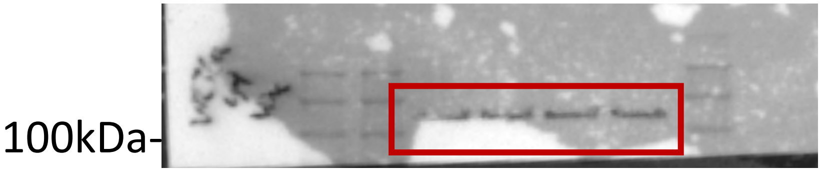

PARP1 Cyt
