# Supplementary material for: The ubiquitin-dependent ATPase p97 removes cytotoxic trapped PARP1 from chromatin
Source: Nat Cell Biol. 2022 Jan 10;24(1):62–73. doi: 10.1038/s41556-021-00807-6 (PMC8760077; doi:10.1038/s41556-021-00807-6)
Supplement: Supplementary file 2 — Peer Review Information [file 41556_2021_807_MOESM2_ESM.pdf]

natureresearch

Peer Review Information

Journal: Nature Cell Biology
Manuscript Title: The ubiquitin-dependent ATPase p97 removes cytotoxic trapped PARP1 from chromatin
Corresponding author name(s): Stephen J. Pettitt, Andrew N.J. Tutt, Kristijan Ramadan, Christopher J. Lord

Reviewer Comments & Decisions:

Decision Letter, initial version:

Date: 30th March 21 19:51:02
From: zhe.wang@nature.com
To: Kristijan.Ramadan@oncology.ox.ac.uk
CC: ncb@springernature.com; jie.wangzhe@nature.com
Subject: Decision on Nature Cell Biology submission NCB-R44936
Message:

*Please delete the link to your author homepage if you wish to forward this email to co-authors.

Dear Professor Ramadan,

I am writing on behalf of my colleague Jie Wang, who is currently out of the office.

Your manuscript, "The ubiquitin-dependent ATPase p97 removes cytotoxic trapped PARP1 from chromatin", has now been seen by 3 referees, who are experts in ATPase, ubiquitin (referee 1); DNA repair, PARP, ubiquitin (referee 2); and repair, cancer (referee 3). As you will see from their comments (attached below) they find this work of potential interest, but have raised substantial concerns, which in our view would need to be addressed with considerable revisions before we can consider publication in Nature Cell Biology.

Nature Cell Biology editors discuss the referee reports in detail within the editorial team, including the chief editor, to identify key referee points that should be addressed with priority, and requests that are overruled as being beyond the scope of the current study. To guide the scope of the revisions, I have

listed these points below. We are committed to providing a fair and constructive peer-review process, so please feel free to contact me if you would like to discuss any of the referee comments further.

In particular, it would be essential to:

A) Strengthen the data to support that p97 directly removes PARP from chromatin as questioned by Reviewer 2:

"p97/VCP plays multiple roles in DNA repair and DNA replication regulation. The authors need to exclude the possibility that defective DNA repair is not the reason why PARP seems to be retained on chromatin after DNA damage. There are multiple means to address this but one is to assess whether the MMS sensitivity of PARP1-/- cells exacerbated by p97 inhibition?"

"Shan Zha reported last year that PARP1 can be rapidly exchanged at sites of laser microirradiation even in the presence of PARP inhibition (PMID: 32890402). These results suggest either that there are activities involved in removing PARP1 from DNA damage sites (such as p97), or that trapping is largely an in vitro phenomenon. Is p97 involved in promoting PARP1 exchange at DNA damage sites using FRAP?"

"In their trap/chase experiments (Fig 4), why are the authors removing PARPi in their chase? Removing talazoparib allows PARP1 to be reactivated, DNA repair to resume, etc? Would it not make more sense to maintain the trapping agent and examine the release of PARP1 from DNA under those conditions?"

"Finally, the authors rely nearly exclusively on p97 inhibitors. As a means to fully exclude off-target effect of the compounds, key experiments could be confirmed with a p97 dominant-negative protein."

B) Add proper controls as requested by Reviewer 1:

"The negative controls for the proteomics experiments appear to be flawed. For the proximity labeling, a cell line unable to undergo Apex labeling at all is a poor choice. Better alternatives would be cells expressing PARP1del.p119K120S-Apex2-eGFP or just Apex2-eGFP. Similarly, the correct background control for the RIME MS-IP experiments would be eGFP expressing cells, not PARP1 knockout cells. These flaws make the analysis of the results and the candidate selection somewhat questionable/arbitrary and may explain the largely unexpected collection of enriched GO terms (Fig. 1G). Also, the decision to focus on candidates with high MS scores but low PSM ratio +/- talazoparib appears counterintuitive, since it might simply favor highly abundant proteins such as SUMO or p97/VCP over proteins that were specifically enriched at trapped PARP1. Of note, the RIME results show p97/VCP to be actually depleted from trapped PARP1 (PSM ratio +/- talazoparib of 0.4 according to Suppl. Table 3), in contrast to the statement in line 226/227."

"The PLA assays are in need of additional controls and quantifications. Fig. 3A shows that a background of PLA foci is observed in the presence of either the PARP1 or (more so) the p97/VCP antibody, even under non-stressed conditions. This background needs to be quantified, and the sum of the background foci must be compared to the "true" PLA foci in all quantifications for each condition. Since p97/VCP is likely to be recruited to/trapped at sites of DNA damage in the presence of MMS and/or CB-5083 independent of PARP1 trapping, a corresponding increase in the p97-antibody-only control is likely and has to be accounted for."

C) All other referee concerns pertaining to strengthening existing data, providing controls, methodological details, clarifications and textual changes as appropriate should also be addressed.

D) Finally please pay close attention to our guidelines on statistical and methodological reporting (listed below) as failure to do so may delay the reconsideration of the revised manuscript. In particular please provide:

- a Supplementary Figure including unprocessed images of all gels/blots in the form of a multi-page pdf file. Please ensure that blots/gels are labeled and the sections presented in the figures are clearly indicated.

- a Supplementary Table including all numerical source data in Excel format, with data for different figures provided as different sheets within a single Excel file. The file should include source data giving rise to graphical representations and statistical descriptions in the paper and for all instances where the figures present representative experiments of multiple independent repeats, the source data of all repeats should be provided.

We would be happy to consider a revised manuscript that would satisfactorily address these points, unless a similar paper is published elsewhere, or is accepted for publication in Nature Cell Biology in the meantime.

When revising the manuscript please:

- ensure that it conforms to our format instructions and publication policies (see below and https://www.nature.com/nature/for-authors).

- provide a point-by-point rebuttal to the full referee reports verbatim, as provided at the end of this letter.

- provide the completed Reporting Summary (found here https://www.nature.com/documents/nr-reporting-summary.pdf). This is essential for reconsideration of the manuscript will be available to

editors and referees in the event of peer review. For more information see http://www.nature.com/authors/policies/availability.html or contact me.

When submitting the revised version of your manuscript, please pay close attention to our href="https://www.nature.com/nature-research/editorial-policies/image-integrity">Digital Image Integrity Guidelines. and to the following points below:

-- that unprocessed scans are clearly labelled and match the gels and western blots presented in figures.
-- that control panels for gels and western blots are appropriately described as loading on sample processing controls
-- all images in the paper are checked for duplication of panels and for splicing of gel lanes.

Finally, please ensure that you retain unprocessed data and metadata files after publication, ideally archiving data in perpetuity, as these may be requested during the peer review and production process or after publication if any issues arise.

Nature Cell Biology is committed to improving transparency in authorship. As part of our efforts in this direction, we are now requesting that all authors identified as ‘corresponding author’ on published papers create and link their Open Researcher and Contributor Identifier (ORCID) with their account on the Manuscript Tracking System (MTS), prior to acceptance. ORCID helps the scientific community achieve unambiguous attribution of all scholarly contributions. You can create and link your ORCID from the home page of the MTS by clicking on ‘Modify my Springer Nature account’. For more information please visit please visit www.springernature.com/orcid.

This journal strongly supports public availability of data. Please place the data used in your paper into a public data repository, or alternatively, present the data as Supplementary Information. If data can only be shared on request, please explain why in your Data Availability Statement, and also in the correspondence with your editor. Please note that for some data types, deposition in a public repository is mandatory - more information on our data deposition policies and available repositories appears below.

Please submit the revised manuscript files and the point-by-point rebuttal to the referee comments using this link:

[REDACTED]

*This url links to your confidential home page and associated information about manuscripts you may have submitted or be reviewing for us. If you wish to forward this email to co-authors, please delete the link to your homepage.

We would like to receive a revised submission within six months.

We hope that you will find our referees' comments, and editorial guidance helpful. Please do not hesitate to contact me if there is anything you would like to discuss.

Best wishes,

Zhe Wang

Zhe Wang, PhD
Senior Editor
Nature Cell Biology

Tel: +44 (0) 207 843 4924
email: zhe.wang@nature.com

Reviewers' Comments:

Reviewer #1:
Remarks to the Author:
This manuscript addresses the role of the ATPase p97 (also known as VCP) in the removal of trapped PARP1 from chromatin. Starting from a combination of proximity labeling and IP-MS proteomics, the authors identified p97/VCP and SUMO1/2 as potential interactors of talazoparib-trapped PARP1. They went on to show that trapped PARP1 is both SUMOylated and ubiquitylated and that PIAS4 and RNF4 are the respective E3 ligases catalyzing these modifications, with RNF4 acting downstream of PIAS4 in its capacity as SUMO-targeted ubiquitin ligase (StUbl). The authors then confirmed that p97/VCP interacts with trapped, ubiquitylated PARP1 and showed that p97/VCP is required for its efficient removal from chromatin. Finally, they showed that talazoparib and the p97/VCP inhibitor CB-5083 exhibit strongly synergistic effects on the viability of BRCA1/2-negative cancer cells in 2D and 3D cellular models.

Krastev et al. present an interesting study containing a wealth of experiments on a mostly high technical standard. Unfortunately, however, their data are largely confirmatory in many points, including the SUMOylation of proteins at sites of DNA damage; the role of the StUbl RNF4 in DNA damage repair; and

the role of p97/VCP in removing ubiquitylated proteins from chromatin, including sites of DNA damage. Basically, they establish trapped PARP1 as a novel "model substrate" to exemplify and connect the aforementioned steps in one pathway, but they do not provide new functional or mechanistic insights into any of the steps. The data on the synergistic action of the PARP and p97/VCP inhibitors are potentially of high clinical relevance, making this study perhaps more suitable for a stronger translation-oriented journal.

Specific comments:

1. Fig. 1: The negative controls for the proteomics experiments appear to be flawed. For the proximity labeling, a cell line unable to undergo Apex labeling at all is a poor choice. Better alternatives would be cells expressing PARP1del.p119K120S-Apex2-eGFP or just Apex2-eGFP. Similarly, the correct background control for the RIME MS-IP experiments would be eGFP expressing cells, not PARP1 knockout cells. These flaws make the analysis of the results and the candidate selection somewhat questionable/arbitrary and may explain the largely unexpected collection of enriched GO terms (Fig. 1G). Also, the decision to focus on candidates with high MS scores but low PSM ratio +/- talazoparib appears counterintuitive, since it might simply favor highly abundant proteins such as SUMO or p97/VCP over proteins that were specifically enriched at trapped PARP1. Of note, the RIME results show p97/VCP to be actually depleted from trapped PARP1 (PSM ratio +/- talazoparib of 0.4 according to Suppl. Table 3), in contrast to the statement in line 226/227.

2. Figs. 3/4: The PLA assays are in need of additional controls and quantifications. Fig. 3A shows that a background of PLA foci is observed in the presence of either the PARP1 or (more so) the p97/VCP antibody, even under non-stressed conditions. This background needs to be quantified, and the sum of the background foci must be compared to the "true" PLA foci in all quantifications for each condition. Since p97/VCP is likely to be recruited to/trapped at sites of DNA damage in the presence of MMS and/or CB-5083 independent of PARP1 trapping, a corresponding increase in the p97-antibody-only control is likely and has to be accounted for.

3. Fig. 4BC: PARP1 appears to be efficiently removed from chromatin even in the absence of PIAS4 or RNF4. How is this possible in light of the model shown in Fig. 4M? On a technical note, the H3 loading controls are heavily overexposed, precluding any quantitative analysis of the results. These experiments should be repeated and quantified in triplicates, with the samples from wildtype and knockout cells loaded on the same gel and with all loading controls in the linear detection range.

Minor points:

4. Fig. 1: The expression levels of the engineered PARP1 fusions used should be compared to the level of endogenous PARP1 in the parental cell line.

5. Figs. 2/S2: The assignment of ubiquitylated and/or SUMOylated PARP1 species is not always clear. Sometimes these bands run at ≤150 kDa, sometimes at >>150 kDa. The authors should more clearly label/explain their identity.

6. It is somewhat surprising that the authors identified UFD1 but not NPL4 to be involved in PARP1 turnover, even though NPL4 appears to be critical for initializing the unfolding of ubiquitylated substrates by p97/VCP for subsequent proteasomal degradation. Did they identify UFD1 (but not NPL4) in their proteomics datasets? Does depletion of UFD1 (but not NPL4) result in the accumulation of trapped PARP1 on chromatin and/or in the reduction of p97 association with chromatin?

7. The recent publication by the Dantuma lab on the role of PARylation of SUMOylated ATX3 during DNA DSB repair should be discussed (Pfeiffer et al, J Cell Sci 2021; doi:10.1242/jcs.247809).

8. line 264/265: The E1 is the ubiquitin activating enzyme, not a ligase.

Reviewer #2:
Remarks to the Author:
PARPi cytotoxicity mainly relies on the enzymatic trapping of PARP1 on DNA. However, exactly how trapped PARP is recognized and processed is poorly understood and an important question in PARP biology and for PARP inhibitor therapies. In this manuscript Krastev and colleagues carried two orthogonal proteomic searches to uncover proteins that interact with trapped PARP. These analyses identified p97/VCP as a candidate trapped PARP-interacting protein.

The authors put forward a model in which trapped PARP1 undergoes sequential sumoylation and poly-ubiquitylation by PIAS4 and RNF4, respectively. These events generate an interacting platform for the p97/VCP unfoldase which removes PARP1-trapped complexes from the chromatin. They finally show that p97 inhibitors can potentiate the effect of talazoparib in killing HR-deficient cells and tumor organoids.

Overall, the identification of a p97/VCP as a factor that modulates PARP is interesting, especially in light of the data that hints at synergy between PARP and p97 inhibitors. However, the main thrust of the paper, i.e. that p97/VCP removes cytotoxic trapped PARP1 from the chromatin is supported primarily by indirect data that also have alternative interpretations.

I have also the following additional comments:

1) p97/VCP plays multiple roles in DNA repair and DNA replication regulation. The authors need to exclude the possibility that defective DNA repair is not the reason why PARP seems to be retained on chromatin after DNA damage. There are multiple means to address this but one is to assess whether the MMS sensitivity of PARP1-/- cells exacerbated by p97 inhibition?

2) Does a p97-dominant negative protein accumulate at sites of DNA lesions in a PARP1-trapping dependent manner? This would strengthen the idea that PARP1 trapping recruits p97.

3) Shan Zha reported last year that PARP1 can be rapidly exchanged at sites of laser microirradiation even in the presence of PARP inhibition (PMID: 32890402). These results suggest either that there are activities involved in removing PARP1 from DNA damage sites (such as p97), or that trapping is largely an in vitro phenomenon. Is p97 involved in promoting PARP1 exchange at DNA damage sites using FRAP?

4) In most systems, p97-dependent removal of ubiquitylated proteins from chromatin results in protein degradation. On prediction is therefore that PARP1 is degraded after talazoparib treatment in a p97-dependent manner. From the figures in the paper (e.g. Fig 2) it does not appear that this is the case, why?

5) The Altmeyer group has linked the E3 ubiquitin ligase TRIP12 to the modulation of trapped PARP1. However, this current manuscript proposes a completely different system for modulating PARP1 (PIAS4-dependent sumoylation following RNF4-dependent ubiquitylation). Can the authors address this? Are there multiple systems for PARP1 modulation, cell type specificity or is there a discrepancy?

6) In their trap/chase experiments (Fig 4), why are the authors removing PARPi in their chase? Removing talazoparib allows PARP1 to be reactivated, DNA repair to resume, etc? Would it not make more sense to maintain the trapping agent and examine the release of PARP1 from DNA under those conditions?

7) I do not fully understand why the CB-5083/talazoparib combination is selective for BRCA-deficient cells and tumors? What is the basis of this selectivity? Could it be that this is due to effects of p97 in processes unrelated to PARP1 trapping?

8) Finally, the authors rely nearly exclusively on p97 inhibitors. As a means to fully exclude off-target effect of the compounds, key experiments could be confirmed with a p97 dominant-negative protein.

Reviewer #3:
Remarks to the Author:
Krastev et al., NCB review

In their study, “The ubiquitin-dependent ATPase p97 removes cytotoxic trapped PARP1 from chromatin”, Krastev and colleagues address the issue of PARPi-mediated PARP1 trapping on DNA damaged chromatin, and the mechanism by which trapped PARP1 is normally released. They do this through a series of elegant, integrative techniques including RIME mass spectrometry, Apex2-proximity labelling and more.

Overall, I found this a very clear and well written study with a strong narrative making it very easy for the reader to understand, appreciate and follow. The overall of topic is of broad interest from a basic biological discovery standpoint, from a clinically-relevant drug discovery/mechanism standpoint, and from a “this is good science” general interest standpoint. The data are of high quality, and collectively support the authors conclusions. There are some occasions, detailed below, where I have concerns over statistical power of some key experimental imaging-based endpoints, which could be addressed by higher throughput and automated quantitation which is now standard.

1) The RIME and Apex2-based mass spectrometry screens for trapped-PARP1 interactors appears to me to be robust, and well controlled. I am not, per se, an expert in mass spectrometry however, and so will defer to other reviewers with regards to technical nuances of those experiments and the means by which strong hits were identified, and other protein hits disregarded. The logic, as presented, does seem to be sound, and it is advantageous to take forward a hit (p97) identified by both methods.

2) Is figure 2E mislabelled in terms of a (+) symbol for the MLN-7243 in lane three? As I read the results, this was mean to test the impact of the Ub inhibitor with and without the SUMO inhibitor (ML-792). But as described in figure, lanes 3 and 5 are identical but I am thinking should not be.

3) In terms of the RNF4 depletion experiment in Fig S2E, while I agree that there was a visual reduction in the Ub-PARP-1 signal present here, the depletion of RNF4 certainly does not completely ablate this, and the blots here are far less convincing in terms of Ub-PARP1 signal versus those shown in Fig 2B-D. To what extent (i.e. quantified data) did RNF4 suppress Ub-PARP1? I feel that this needs to be assessed, to give the reader a sense of the relative contribution of RNF4 to the process. The data in Fig 2G also suggests that loss of RNF4 produces partial effects, albeit to a stronger extent versus the siRNA-based method (perhaps to be expected). Complementation with wildtype and ubiquitylation-dead RNF4 would be prudent here, to consolidate the specificity of the effects being observed within cells. The in vitro experiments in Figures 2HI are very nice.

4) Data in 3C, 3E, 3H need to encompass a quantitation of a lot more than 50 cells per condition, as indicated in the legends for 3C. From a statistical power perspective, these experiments do not meet the bar as is, although I agree the trends certainly support the authors conclusions and the accompanying western blots. Ideally this enumeration should be automated (the methods state some are manually scored some automated – but which are which? why mix methods? and either way manual is not ideal), and performed across several hundred cells per experimental replicate (it is not clear from methods how many times this was repeated, and if the 50 nuclei are from 1 experiment, or pooled from much lower cell numbers gathered from several). Either way, many hundreds of cells per replicate are needed here, with more detailed methodology on how cells are selected (or not) for enumeration. It is also not clear what the black/green bars in 3C or 3H represent (median, average?). Ideally geometric mean values with

95% confidence intervals should be displayed for raw datapoints across pooled experimental repeats, with dots set to a degree of transparency to show data density. Individual geometric mean foci number outcomes between experimental replicates can be compared in terms of statistics also. However, as this is a key piece of quantitative data for this study, this needs to be strengthened.
5) Blots in Figure 4 are convincing, but would be more so if quantified data merging several experiments of quantified PARP1 signal set relative to the trap condition were shown. It is hard to get a sense of the fold differences just by eye. The PLA data in 4D-F has the same issues as I described above with respect to Figure 3, and all those comments apply here. An siRNA resistant RNF4 (wt and catalytic dead) addback would be prudent for data in 4F, with expression controls etc. A lot of the effects in 4G-I could be confounded by differences in cell cycle profile and/or DNA replication status in the cultures treated with different inhibitors during the experiment. Controls are needed to address whether this is an issue or not. Survival data are generally convincing, although a statistical evaluation of synergy is missing. The model in Fig 4M presupposes that RNF4 is the only ligase contributing to trapped PARP1 Ub, which is not supported by the data which shows some remaining Ub of trapped PARP1 in it's absence. Model should reflect this and acknowledge the extent of contributions based on quantified data.
Minor points:
• If possible, breaking up the multi-panel figures would be better. As they are, the data is cramped into four very crowded figures with exceptionally small text (e.g. Figure 1F...). Not sure if this was due to editorial constraints... in my experience NCB allows more than four primary figures and, if that is the case here, I would suggest making use of that and splitting these apart, so they are more digestible on the page.
• Input controls for total Ubiquitin are important for Figure 2G, and are missing.
• Westerns for H3 in figure 4BC are pretty poor, being so over exposed to the extent of almost being meaningless as loading controls. Suggest a do-over.
• Statistical evaluations of data in Fig S4BD are missing.
• The word 'extraction' is misspelled in Figure 4M.

Author Rebuttal to Initial comments

Reviewer #1:

Remarks to the Author:

This manuscript addresses the role of the ATPase p97 (also known as VCP) in the removal of trapped PARP1 from chromatin. Starting from a combination of proximity labeling and IP-MS proteomics, the authors identified p97/VCP and SUMO1/2 as potential interactors of talazoparib-trapped PARP1. They went on to show that trapped PARP1 is both SUMOylated and ubiquitylated and that PIAS4 and RNF4 are the respective E3 ligases catalyzing these modifications, with RNF4 acting downstream of PIAS4 in its capacity as SUMO-targeted ubiquitin ligase (StUbl). The authors then confirmed that p97/VCP interacts with trapped, ubiquitylated PARP1 and showed that p97/VCP is required for its efficient removal from chromatin. Finally, they showed that talazoparib and the p97/VCP inhibitor CB-5083 exhibit strongly synergistic effects on the viability of BRCA1/2-negative cancer cells in 2D and 3D cellular models.

Krastev et al. present an interesting study containing a wealth of experiments on a mostly high technical standard. Unfortunately, however, their data are largely confirmatory in many points, including the SUMOylation of proteins at sites of DNA damage; the role of the StUbl RNF4 in DNA damage repair; and the role of p97/VCP in removing ubiquitylated proteins from chromatin, including sites of DNA damage. Basically, they establish trapped PARP1 as a novel "model substrate" to exemplify and connect the aforementioned steps in one pathway, but they do not provide new functional or mechanistic insights into any of the steps. The data on the synergistic action of the PARP and p97/VCP inhibitors are potentially of high clinical relevance, making this study perhaps more suitable for a stronger translation-oriented journal.

Our response: We thank the reviewer for taking the time to review our manuscript. Whilst we appreciate that the SUMOylation of proteins at sites of DNA damage has been reported previously (and this is only one aspect of our manuscript), we do not think this necessarily undermines the novelty nor importance of our work. Until now, an understanding of the processes that cells use to process trapped PARP1, a drug-induced cytotoxic DNA lesion, was

largely limited to the role of homologous recombination (HR) in repairing the DNA damage that is the result of PARP1 trapping. This has meant that what happens between PARP1 trapping and the activation of HR has remained, for some considerable time, a “black box”. Our work demonstrates that p97-mediated processing provides one explanation. Our observations also highlight that the processes that cells use to remove functional proteins from chromatin as part of normal cellular metabolism, are also co-opted to deal with therapy-induced DNA lesions such as trapped PARP1. As far as we are aware, nobody has demonstrated that trapped PARP1 SUMOylation and ubiquitylation recruits p97 or identified the enzymes that mediate this process. Importantly, we have uncovered novel molecular characteristics of the process which could not have been inferred by prior literature knowledge - e.g. we have established that UFD1, but not NPL4 as the literature would suggest, is the critical mediator of the PARP1-p97 interaction. We also show that this process is only stimulated by PARP inhibitors that trap PARP1 (and not by PARPi that only inhibit PARP1 catalytic activity).

Finally, we feel that this work would also stimulate others into investigating additional aspects of how DNA lesions are processed by cells, which is not only an area that could be of translational interest but is also of relevance to those who focus on the basic biology of normal and cancerous cells.

Specific comments:

1. Fig. 1: The negative controls for the proteomics experiments appear to be flawed. For the proximity labeling, a cell line unable to undergo Apex labeling at all is a poor choice. Better alternatives would be cells expressing PARP1del.p119K120S-Apex2-eGFP or just Apex2-eGFP. Similarly, the correct background control for the RIME MS-IP experiments would be eGFP expressing cells, not PARP1 knockout cells. These flaws make the analysis of the results and the candidate selection somewhat questionable/arbitrary and may explain the largely unexpected collection of enriched GO terms (Fig. 1G). Also, the decision to focus on candidates with high MS scores but low PSM ratio +/- talazoparib appears counterintuitive, since it might simply favor highly

abundant proteins such as SUMO or p97/VCP over proteins that were specifically enriched at trapped PARP1.

Our response: For the RIME MS experiments we used CAL51 *PARP1*$^{-/-}$ cells and derivatives of these expressing either PARP1^{WT}-eGFP or PARP1$^{del.p.119K120S}$-eGFP (the later being a PARP1-trapping defective allele) and isolated proteins using GFP-Trap beads, which harbour a nanobody with a high specificity towards GFP. In any immunoprecipitation experiment, a common problem is proteins that bind to the beads in a non-specific fashion. Given this, as an additional control, we also included an analysis of cells lacking eGFP – this could have been cells expressing a wild type PARP1 transgene but no eGFP or the parental cells of the transgenic cells described above, CAL51 *PARP1*$^{-/-}$ cells. Given the size of these experiments, we opted for the most informative control, which was the parental CAL51 *PARP1*$^{-/-}$ cells, which allowed us to remove from the analysis those proteins that non-specifically to the beads. We see no contradiction or insufficiency in using these cells as a control. As we demonstrated later in the manuscript, this overall approach identified proteins whose interactions were enhanced upon PARP1 trapping, suggesting some validity in the method taken.

To explain this better in the revised manuscript, we have now revised the main text, so that the reasoning for this approach is better set out. For example, the following text is now included:

"As PARP1 translocates to chromatin upon DNA damage, we first used RIME-based immunoprecipitation[11, 13], to identify proteins associated with trapped PARP1 (Figure 1A). In these experiments, PARP1^{WT}-eGFP and PARP1$^{del.p.119K120S}$-eGFP expressing cells were exposed to PARP1 trapping conditions (methyl methanesulfonate (MMS) + talazoparib added to the tissue culture media) after which protein interactions were stabilised by formaldehyde crosslinking. MMS was used to create PARP1-binding DNA lesions, whereas the PARPi, talazoparib, was used to inhibit and trap DNA-bound PARP1. After trapping, chromatin-bound proteins were isolated and PARP1-associated complexes immunoprecipitated from this chromatin fraction using GFP-Trap beads, which harbour a nanobody with high specificity towards GFP. Immunoprecipitated proteins were then identified by mass spectrometry. As a control, we also included an analysis of the parental CAL51 *PARP1*$^{-/-}$ cells lacking eGFP, in order to identify proteins that bind non-specifically to the GFP-Trap beads (Supplementary Figure 1B). These non-specific bead-binding proteins were removed from the list of proteins identified in the PARP1^{WT}-eGFP and PARP1$^{del.p.119K120S}$-eGFP expressing cells (detailed description in Methods)."

As for the APEX labelling experiment, we agree that the *PARP1del.p119K120S-Apex2-eGFP* cells would have been an ideal control. However, when we were generating our models, we did not manage to obtain a clone expressing this particular transgene (despite trying). To remove proteins that bind non-specifically to the beads, irrespective of biotinylation, we used cells that were not treated with biotin-phenol. This is, undoubtedly, the reason why we identified a larger number of PARP1-interacting proteins in the APEX2 labelling experiment, which we could not filter effectively based on a mutant control. For this reason, we used the PSM score as a filter, precisely because this indicates abundance of the protein in the identified complexes. Despite the longer PARP1 protein interaction list, GO enrichment analysis identified relevant processes e.g. base excision repair, as opposed to a random list of proteins.

To acknowledge the above, we have now revised the manuscript so that we now state:

"As an orthogonal MS approach, we employed Apex2-mediated proximity labelling. Apex2 peroxidase generates free radicals which in the presence of biotin-phenol (BP), biotinylates proteins within a ~20 nm radius; biotinylated proteins can then be purified via Streptavidin-binding. To identify proteins associated with trapped PARP1, we performed Apex2 labelling in cells expressing $PARP1^{WT}$-Apex2-eGFP. Western blotting confirmed biotinylation of $PARP1^{WT}$-APEX2-eGFP in the presence, but not absence of biotin-phenol, indicating effective labelling (Supplementary Figure 1C). The amount of labelled PARP1 was further increased when PARP1 labelling was conducted under trapping conditions (MMS + talazoparib) (Supplementary Figure 1C). Although we were unable to generate a clone with a trapping-defective PARP1 allele fused to Apex2-eGFP, we used $PARP1^{WT}$-eGFP-expressing cells as a negative control for the labelling and purification (because of the absence of Apex2, these cells were unable to perform the biotinylation reaction). Biotinylated proteins were then purified under stringent conditions and analysed by mass spectrometry. Non-specific, background protein interactions with beads were removed by filtering the list of $PARP1^{WT}$-Apex2-eGFP-interacting proteins against the list of proteins identified in $PARP1^{WT}$-eGFP expressing cells (detailed analysis description in the Methods). As a result, we identified a higher number of proteins, 360, that associated with PARP1 than for RIME (either in the presence or absence of PARPi, Supplementary Table 3). A STRING network analysis, using a high stringency cut off (0.7) representing the trapped PARP1 interactome network (Supplementary Figure 1D), was enriched in proteins associated with one of the main DNA repair processes PARP1 is involved in, Base Excision Repair (BER), (e.g. PARP1 itself, PCNA, HMGB1, LIG3 and POLE, *p*-value<0.01, Supplementary Figure 1D, E), giving us high confidence in the analysis. Gene Ontology enrichment analysis also identified an enrichment in proteins involved in the spliceosome and ribosome biogenesis (Supplementary Table 4). We also identified a number of well-characterised PARylation targets (e.g. PCNA, NCL, FUS, ILF3[14, 15]) strengthening the notion that we identified *bona fide* PARP1-proximal proteins."

"Of note, the RIME results show p97/VCP to be actually depleted from trapped PARP1 (PSM ratio +/- talazoparib of 0.4 according to Suppl. Table 3), in contrast to the statement in line 226/227."

Our response: In the revised manuscript, we have described our MS data analysis in more detail to make clear that p97 was identified in the APEX2 proximity labelling experiment, based on its abundance. Furthermore, p97 was also identified in the $PARP1^{WT}$-eGFP, but not in the $PARP1^{del.p.119K120S}$-eGFP RIME analysis, suggesting that the interaction is trapping dependent. This information was sufficient in order to prioritise p97 for further analysis.

To acknowledge the above, we have now revised the manuscript so that we now state:

"Among the most abundant labelled proteins were the ubiquitin-like modifier-activating enzyme 1 (UBA1), which has been previously implicated in ubiquitylation events at the sites of DNA damage[16] and the transitional endoplasmic reticulum ATPase, p97 (also known as valosin containing protein, VCP), which acts as a central component of a ubiquitin-controlled process. p97's ATP-dependent unfoldase activity extracts proteins from chromatin prior to their proteasomal degradation or recycling[8-10, 17-19]. Furthermore, p97, working with cofactors that often contain ubiquitin binding domains (UBDs), recognises client proteins via ubiquitylation events, mostly those involving lysine-48 (K48) and lysine-6 (K6)[20, 21] ubiquitylation. p97 was also identified in the $PARP1^{WT}$, but not in the $PARP1^{del.p.119K120S}$ RIME analysis, suggesting that this interaction is trapping-dependent."

2. Figs. 3/4: The PLA assays are in need of additional controls and quantifications. Fig. 3A shows that a background of PLA foci is observed in the presence of either the PARP1 or (more so) the p97/VCP antibody, even under non-stressed conditions. This background needs to be quantified, and the sum of the background foci must be compared to the "true" PLA foci in all quantifications for each condition. Since p97/VCP is likely to be recruited to/trapped at sites of DNA damage in the presence of MMS and/or CB-5083 independent of PARP1 trapping, a corresponding increase in the p97-antibody-only control is likely and has to be accounted for.

Our response: Thank you for pointing this out. We excluded these controls in the original submission so that the figures were not too complex but now include them in the revised manuscript as updated **Figures 4D and E**. The anti-PARP

nature research

antibody, when used alone, produced almost no foci over background levels, whilst the p97 antibody produced a weak signal on its own – around 3-6 foci per nucleus. Importantly, when combined they produced an interaction signal with 10-15 foci per nucleus; this number increased to 30-40 foci in cells grown in trapping conditions and p97 inhibitors.

We have now conducted experiments with the anti-p97 antibody used alone as a control for potential p97 accumulation. This showed only a very modest effect – the control conditions showing 3 ± 3 p97 PLA foci/nucleus, whilst CB-5083 exposure elicited 6.5 ± 4 foci per nucleus. These values are an order of magnitude lower than the p97-PARP1 PLA signal in cells grown in trapping conditions and p97 inhibitor (30 foci/nucleus). With this in mind, we have modified **Figures 4D** and **E** to reflect the modest change in the "p97 only control". The PLA data have also been improved by increasing the number of cells counted and also formatted as per the comments of the other reviewers. Of note, there is no change in the interpretation of the data.

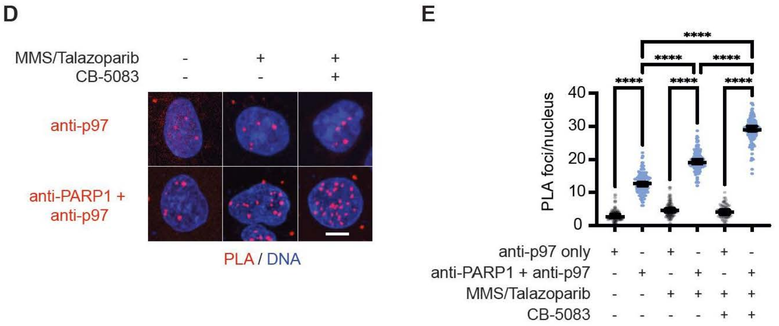

Figure 4D. Confocal microscopy images from a PARP1/p97 PLA experiment where CAL51 cells were exposed to combinations of 0.01 % MMS and 100 nM talazoparib (to induce trapping). PLA was conducted either with p97 antibody only (top row) or p97+PARP1 antibody (bottom row). p97 inhibitor (10 μM CB-5083) was added to stabilise the interaction between p97 and its substrate. Representative images are shown, scale bar = 5 μm. **E.** Quantification of PLA foci/nucleus from experiments in (D)**.** The combination of MMS/talazoparib and CB-5083 enhanced the nuclear PARP1/p97 PLA signal. Quantification of the number of PLA foci/nucleus in n>200 cells from three independent experiments; black bars show the geometric mean ± 95 % CI, p-values were calculated with one-way ANOVA, **** - $p < 0.0001$.

3. Fig. 4BC: PARP1 appears to be efficiently removed from chromatin even in the absence of PIAS4 or RNF4. How is this possible in light of the model shown in Fig. 4M? On a technical note, the H3 loading controls are heavily overexposed, precluding any quantitative analysis of the results. These experiments should be repeated and quantified in triplicates, with the samples from wildtype and knockout cells loaded on the same gel and with all loading controls in the linear detection range.

Our response: We have now repeated these experiments (new **Figure 5B, C**), where the chase was carried out in the presence of talazoparib (also requested by Reviewer #3):

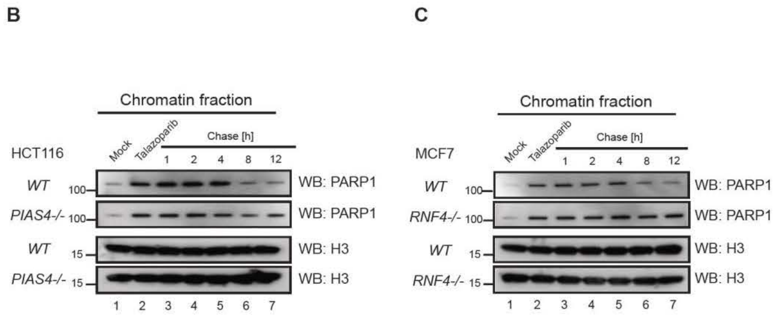

Figure 5 B. Trapped PARP1 is processed in a PIAS4-dependent manner. A trap-chase experiment in HCT116 wild-type or *PIAS4*$^{-/-}$ cells. After PARP1 trapping (100 nM talazoparib + 0.01% MMS), cells were chased in talazoparib-containing media. Samples were collected at indicated time point for chromatin fractionation and Western blotting. In wild type cells, the amount of chromatin-bound PARP1 decreased over the chase period, but in *PIAS4*$^{-/-}$ cells, this process was delayed (see residual PARP1 at 8 and 12 hrs). **C**. Trapped PARP1 is processed in a RNF4-dependent manner. A trap-chase experiment in MCF7 wild-type or RNF4$^{-/-}$ cells similar to (B). The amount of chromatin-bound PARP1 decreases over the chase period. In *RNF4*$^{-/-}$ cells, this process was delayed.

The experiments were repeated multiple times and quantified on the same gels as requested (loading controls shown; full gels included in the revised Supplementary Figure 7). The quantification of this data in now shown in Supplementary Figure 5A and B:

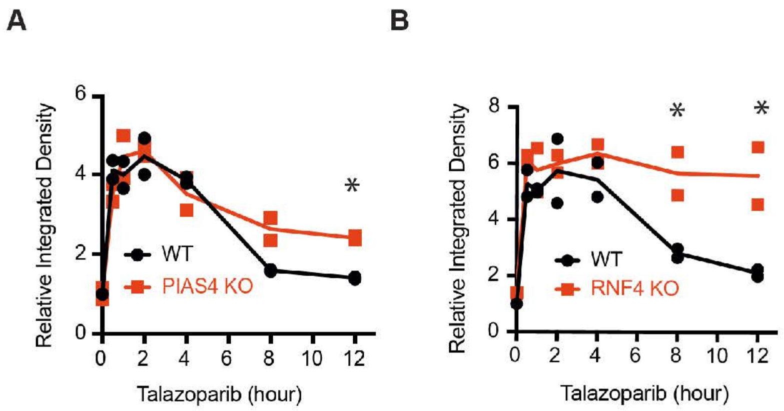

Supplementary Figure 5. A. Quantification of the chromatin bound PARP1 in Figure 5B. * - *p*-value <0.05 on a two-sided *t*-test. **B.** Quantification of the chromatin bound PARP1 in Figure 5C. * - *p*-value <0.05 on a two-sided *t*-test. Two biological replicas are displayed with individual points.

As you can see, the absence of either PIAS4 or RNF4 causes a delay in the removal of PARP1 from the chromatin fraction. These data are in agreement with the biochemical experiments in **Figure 3** and **Supplementary Figure 3**, where even though the absence of PIAS4 or RNF4 reduces the SUMOylation/ubiquitylation (respectively) of trapped PARP1, some residual PARP1 SUMOylation/ubiquitylation exists, suggesting that although PIAS4 and RNF4 are clearly important in this process, other SUMO E3 ligases and ubiquitin E3 ligases might also play a minor role.

With this in mind, we have now modified the model now presented in **Figure 6I** to include “PIAS4 and other SUMO E3 ligase(s)” and “RNF4 and other ubiquitin E3 ligase(s)”.

Minor points:

4. Fig. 1: The expression levels of the engineered PARP1 fusions used should be compared to the level of endogenous PARP1 in the parental cell line.

Our response: We have now assessed this and provided the data in new **Supplementary Figure 1A**. We also show in **Supplementary Figure 1B** the

natureresearch

relative amounts of PARP1^{WT}-eGFP and PARP1$^{del\ p.119K120S}$-eGFP isolated in the RIME experiments.

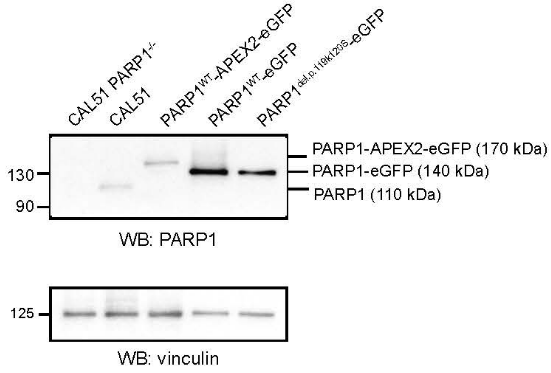

Supplementary Figure 1. **A.** Western blot showing the expression of PARP1 transgenes, detected by an PARP1 antibody.

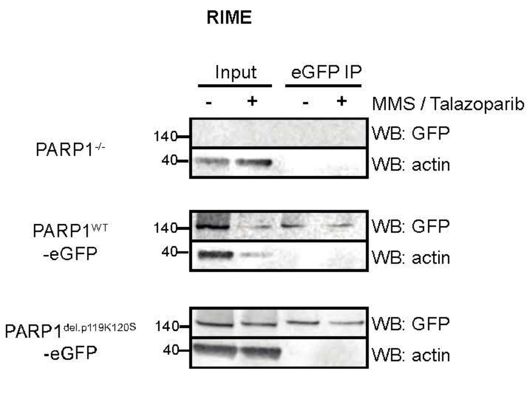

Supplementary Figure 1. **B.** A Western blot analysis of the purified PARP1-associated proteins as described in the RIME experiment in Figure 1A.

5. Figs. 2/S2: The assignment of ubiquitylated and/or SUMOylated PARP1 species is not always clear. Sometimes these bands run at ≤150 kDa, sometimes at >>150 kDa. The authors should more clearly label/explain their identity.

Our response: We have now revised these figures to make clear where

ubiquitylated and/or SUMOylated species are. For example, Figure 2B is now modified to the following:

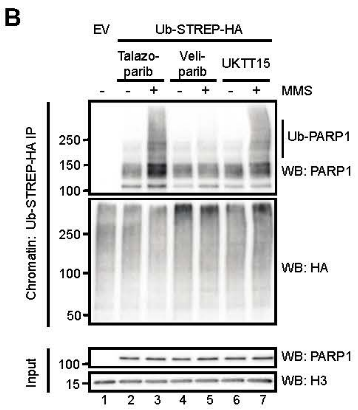

Figure 2. B. PARP1 trapping leads to PARP1 ubiquitylation. HEK293 cells were transfected with Ub-STREP-HA-expressing construct and exposed to combinations of 0.01 % MMS, 100 nM talazoparib, 10 μM Veliparib or 10 μM UKTT15 (a veliparib derivative that induces PARP1 trapping). Chromatin fractions were prepared in denaturing conditions to remove protein/protein interactions and immunoprecipitated with streptactin beads to isolate ubiquitylated proteins. Western blotting of Ub-immunoprecipitates indicated that PARP1 is directly ubiquitylated under trapping conditions. The presence of high MW/Ub forms of PARP1 was increased in the presence of the trapping agent UKTT15 (lane 7), while this was not the case with the non-trapping PARPi veliparib (lane 5).

In addition, we have provided images of all the uncropped blots, which include molecular marker, in new **Supplementary Figure 7**.

6. It is somewhat surprising that the authors identified UFD1 but not NPL4 to be involved in PARP1 turnover, even though NPL4 appears to be critical for initializing the unfolding of ubiquitylated substrates by p97/VCP for subsequent proteasomal degradation. Did they identify UFD1 (but not NPL4) in their proteomics datasets? Does depletion of UFD1 (but not NPL4) result in the accumulation of trapped PARP1 on chromatin and/or in the reduction of p97 association with chromatin?

Our response: To address the first question, we did not detect UFD1 nor NPL4 in the original mass spec profiling, but of course this would not necessarily mean

that these proteins are not involved in processing trapped PARP1 (for example, the interaction could be transient and/or below the level of detection of mass spec.). To address the second question, we have now assessed the interaction between p97 and PARP1 and the total amount of trapped PARP1 in cells where either UFD1 or NPL4 were depleted (new **Figure 4J**). This experiment shows that whilst UFD1 depletion decreased the p97-PARP1 interaction and increased the amount of chromatin-associated trapped PARP1, NPL4 depletion did not.

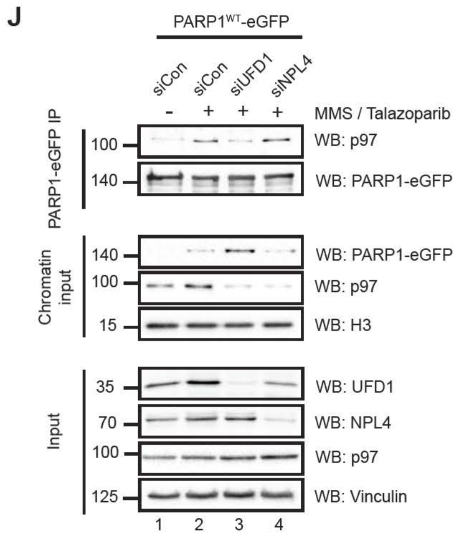

Figure 4J. The p97 adapter UFD1 mediates the interaction between p97 and trapped PARP1. PARP1-p97 interaction was investigated by co-IP from chromatin-bound (trapped) PARP1. RNAi-mediated UFD1 depletion strongly reduced the amount of p97 recruited to the trapped PARP1 complex and caused increased levels of trapped PARP1 in the chromatin fraction. This was not the case in NPL4-depleted cells.

In part, this experiment reproduces our original **Figure 3I**:

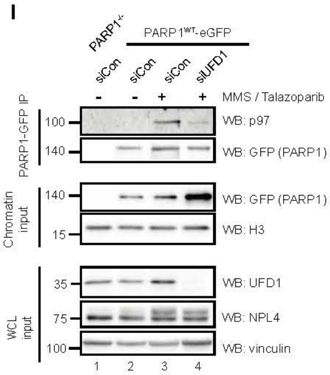

Submitted manuscript Figure 3 I. The p97 adapter UFD1 mediates the interaction with trapped PARP1. PARP1-p97 interaction was investigated by co-IP from the chromatin-bound (trapped) PARP1. RNAi-mediated UFD1 depletion strongly reduced the amount of p97 recruited to the trapped PARP1 complex and caused increased total levels of trapped PARP1 in the chromatin fraction.

…and is also replicated in new **Supplementary Figure 5D**:

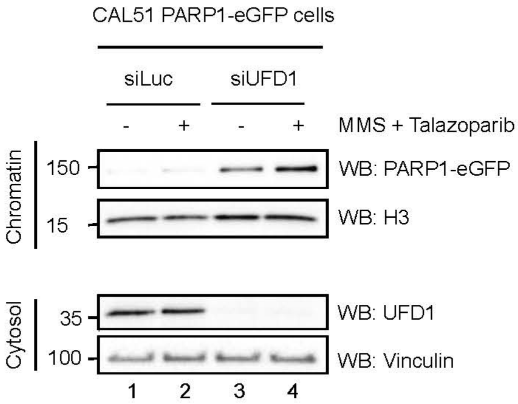

Supplementary Figure 5D. UFD1 is necessary for the efficient extraction of trapped PARP1 from the chromatin. CAL51 PARP1-eGFP expressing cells were transfected with a control siRNA (siLuc) or UFD1-targeting siRNA (siUFD1). 48 hours post transfection, cells were exposed to trapping conditions and then fractionated into cytosolic and chromatin-bound fraction. UFD1 depletion led to higher amount of chromatin bound PARP1, which was further increased by trapping in MMS/Talazoparib conditions.

We note that this might not be the first description of independent roles for UFD1 and NPL4 in p97 substrate processing. For example, CDT1 is removed from

chromatin by p97 in a UFD1-dependent, but NPL4-independent manner (Ramen et al Mol Cell. 2011 Oct 7;44(1):72-84).

Nevertheless, we acknowledge that this is an important point to discuss in the manuscript and have therefore added the following to the revised manuscript:

"Regarding p97 recruitment, our data suggest that UFD1 is required for the recruitment of p97 to trapped PARP1 (Figure 4J). How exactly UFD1 recruits p97 to trapped PARP1 remains to be established. UFD1 is a well-known ubiquitin-chain reader as it possesses Ub-binding domain. In yeast, UFD1 has been shown to bind SUMO (in addition to ubiquitin) and to recruit p97/cdc48 to SUMOylated substrates[39, 40]. However, UFD1 binding to SUMO has never been demonstrated in mammalian cells. Our data presented here suggests that p97 recruitment to trapped PARP1 depends on RNF4-dependent ubiquitylation; it thus seems likely that UFD1 recruits p97 via its canonical role as an ubiquitin-chain reader, directly bridging p97 and the ubiquitin chains on p97 substrates, in this case ubiquitylated PARP1. We also note that although canonically, UFD1 is thought to function as an obligate heterodimer with NPL4, NPL4 silencing did not alter PARP1 trapping nor the PARP1-p97 interaction in the same way that UFD1 depletion did (Figure 4J). Whilst we are unable to entirely rule out a role for NPL4 in the processing of trapped PARP1, it is possible that this is a function, similar to the removal of CDT1 and other substrates from chromatin[7, 32,9], that appears to be UFD1-specific.

7. The recent publication by the Dantuma lab on the role of PARylation of SUMOylated ATX3 during DNA DSB repair should be discussed (Pfeiffer et al, J Cell Sci 2021; doi:10.1242/jcs.247809).

Our response: We thank the reviewer for drawing out attention to this recent paper. We have now introduced a discussion on ATX3 in the main text as follows:

"Recently, the DUB ATXN3 which antagonises RNF4 ubiquitination activity at DNA damage sites, was shown to be recruited to micro-irradiation induced DNA damage in a PAR-dependent manner[39]. In combination with our work here, a tantalising hypothesis can be proposed whereby PARPi mediated PARP1 retention coupled with inhibition of ATXN3 recruitment is as a pre-requisite for RNF4-dependent trapped PARP1 ubiquitination. ."

8. Line 264/265: The E1 is the ubiquitin activating enzyme, not a ligase.

Our response: Thank you. This is now corrected.

End of Referee #1 comments

Reviewer #2:

Remarks to the Author:

PARPi cytotoxicity mainly relies on the enzymatic trapping of PARP1 on DNA. However, exactly how trapped PARP is recognized and processed is poorly understood and an important question in PARP biology and for PARP inhibitor therapies. In this manuscript Krastev and colleagues carried two orthogonal proteomic searches to uncover proteins that interact with trapped PARP. These analyses identified p97/VCP as a candidate trapped PARP-interacting protein.

The authors put forward a model in which trapped PARP1 undergoes sequential sumoylation and poly-ubiquitylation by PIAS4 and RNF4, respectively. These events generate an interacting platform for the p97/VCP unfoldase which removes PARP1-trapped complexes from the chromatin. They finally show that p97 inhibitors can potentiate the effect of talazoparib in killing HR-deficient cells and tumor organoids.

Overall, the identification of a p97/VCP as a factor that modulates PARP is interesting, especially in light of the data that hints at synergy between PARP and p97 inhibitors. However, the main thrust of the paper, i.e. that p97/VCP removes cytotoxic trapped PARP1 from the chromatin is supported primarily by indirect data that also have alternative interpretations.

I have also the following additional comments:

1) p97/VCP plays multiple roles in DNA repair and DNA replication regulation. The authors need to exclude the possibility that defective DNA repair is not the reason why PARP seems to be retained on chromatin after DNA damage. There are multiple means to address this but one is to assess whether the MMS sensitivity of PARP1-/- cells exacerbated by p97 inhibition?

Our response: This is a very important point and by generating new data to address this we have now strengthened the manuscript. We have now carried

out the suggested experiments in *PARP1* wild type and *PARP1*$^{-/-}$ cells using two different alkylating agents, MMS and Temozolomide.

Whilst either MMS or temozolomide enhanced PARP inhibitor sensitivity in a PARP1-dependent manner (the positive control for these experiments), we did not see similar effects when we combined MMS or temozolomide with p97 inhibitor (new **Figure 6D,E**). In comparison, similar concentrations of p97 inhibitor enhanced PARP inhibitor sensitivity (**Figure 6A-C**), suggesting that the effects of p97 on PARP1 trapping and PARPi sensitivity are unlikely to be because of a more generalised effect of p97 inhibitors on DNA repair.

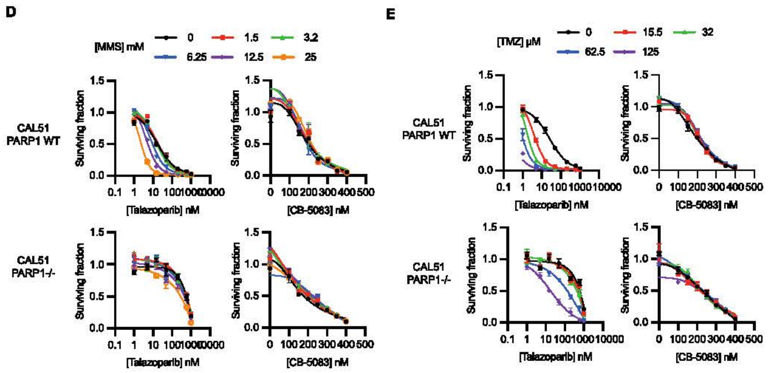

Figure 6 D. and **E**. DNA alkylating agents that are used to induce PARP1 trapping do not enhance the cell inhibitory effects of CB-5083. CAL51 WT or *PARP1*$^{-/-}$ cells were exposed to alkylating agents MMS (D) or temozolomide (TMZ) (E) in combination with either talazoparib (the positive control) or CB-5083 for seven days, after which, cell viability was estimated by CellTiter Glo Reagent.

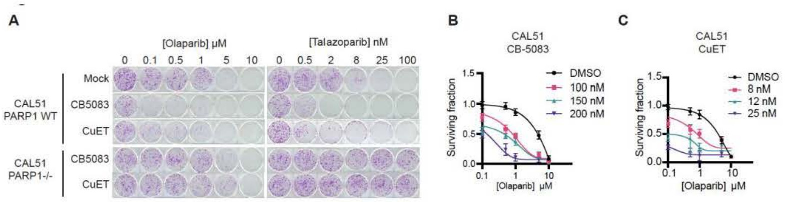

Figure 6A. p97 inhibition potentiates the cytotoxicity of PARP inhibitors. CAL51 cells were exposed to PARP inhibitor (talazoparib or olaparib) in the presence of p97 inhibitor (CB-5083 or CuET) for a period of 14 days after which colonies were fixed and stained by sulphorhodamine B. The presence of either p97 inhibitor led to the potentiation of the PARPi clonogenic effect. Images are shown for the 100 nM CB-5083 and 8 nM CuET exposed samples. This effect was reversed in *PARP1*$^{-/-}$ CAL51 cells, indicating a PARP1-mediated synthetic lethal effect. Drug response curves are shown in (B), (C) and Supplementary Figure 6A, B. **B.** and **C.** Drug-response curves for colony formation experiments shown in (A).

We also show in **Figure 5G-I** that acute p97 inhibition, when used alone (e.g. 3 hours exposure to 10 µM CB-5083), does not appear to induce γH2AX or RAD51 foci, nor alterations in cell cycle progression (new **Supplementary Figure 5I and J**), both of which might be expected if the concentrations of p97 inhibitors used to modulate PARP1 trapping were simply mediating their effects by causing DNA damage or defective DNA repair. Most of our trapping and interaction studies involving p97 were conducted using 1-2 h exposure to p97 inhibitor (at similar concentrations) suggesting that the effects seen are more likely to be reflective of p97 modulating trapped PARP1 than a more general effect of p97 inhibition on DNA repair processes.

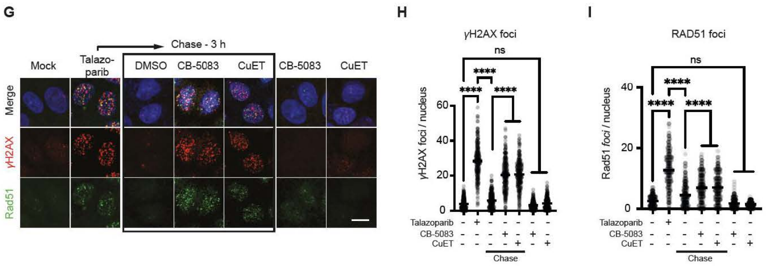

Figure 5 G. PARPi-induced RAD51 and γH2AX foci persist in the presence of p97 inhibitors. Representative confocal microscopy images from a trap-chase experiment (where cells were incubated with talazoparib overnight) are shown, where the effect of PARPi was monitored by immunofluorescent detection of γH2AX and RAD51 foci. In the chase phase the PARPi was washed out and the cells were chased in control or p97 inhibitor-containing media. Representative images for each condition, scale bar = 5 μm. **H** and **I**. Quantification of γH2AX (H) and RAD51 foci (I), from experiment (G). Quantification of the number of foci/nucleus in n>200 cells from three independent experiments; black bars show the geometric mean ± 95 % CI, *p*-values were calculated with one-way ANOVA, **** - $p < 0.0001$.

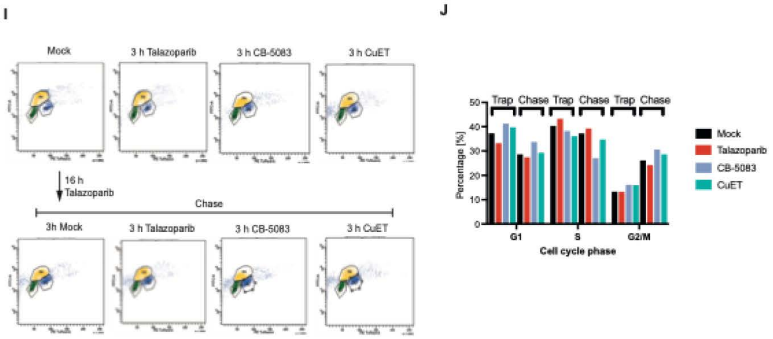

Supplementary Figure 5I. Cell cycle profiling for the experiment shown in Figure 5I. CAL51 cells were exposed to drugs as shown. One hour prior to fixation, 10 μM EdU was added to the media. After cells were removed from culture, EdU was stained by a click reaction with Alexa488-azide and DNA was stained by propidium iodide. **J**. A quantification of the G1, S and G2 populations from (I). The 3 h drug treatment arms do not alter the distribution of the cell cycle phases. In contrast, the 16 h Talazoparib treatment leads to an increase in the G2 population. This accumulation is not changed in the p97 inhibitor exposed arms in the chase period of 3 h.

To reflect this, we have also now added the following passage of text to the discussion:

“During our studies we considered the possibility that the effects on PARP1 trapping and PARP inhibitor cytotoxicity that we observed might not be due to an effect of p97 on trapped PARP1 (the key cytotoxic lesion) but could be due to p97 modulating other DNA repair processes. However, we think this unlikely for a number of reasons: (i) the concentration of p97 inhibitors used in PARP1 trapping and PARP1-interaction experiments (and the duration for which cells were exposed to these) did not, when used as single agents, elicit biomarkers of DNA damage such as γH2AX or RAD51 foci (Figure 5G-I) nor alterations in cell cycle dynamics (Supplementary Figure 5E,F), both of which might be expected if the concentrations of p97 inhibitors used to modulate PARP1 trapping were simply mediating their effects by eliciting DNA damage and/or causing defective DNA repair. Moreover, whilst p97 inhibitors enhanced PARP inhibitor sensitivity in a PARP1-dependent manner (Figure 6A,B), a p97 inhibitor did not alter sensitivity to either of two alkylating agents, MMS or temozolomide (Figure 6C,D). Although it is somewhat difficult to completely negate the possibility that the removal of other p97 substrates from chromatin could influence the repair of trapped PARP1, this data, when taken together with the data showing a SUMO- and ubiquitin dependent interaction between p97 and trapped PARP1, suggests that p97 does play a critical role in the resolution of this DNA lesion. “

2) Does a p97-dominant negative protein accumulate at sites of DNA lesions in a PARP1-trapping dependent manner? This would strengthen the idea that PARP1 trapping recruits p97.

Our response: We have now completed experiments using the dominant negative p97-E578Q mutant that demonstrate that:

1) compared to wild type p97, the p97-E578Q mutant has an enhanced PARP1 interaction under trapping conditions, consistent with the E578Q mutant causing a substrate trapping effect (new **Figure 4G**);

2) the p97-E578Q/PARP1 interaction is trapping-dependent, being much reduced in cells expressing the trapping-defective PARP1$^{del\ p.119K120S}$ mutant (new **Supplementary Figure 4D**).

3) The p97-E578Q mutant colocalises with PARP1 under PARP1 trapping conditions (**Supplementary Figure 4E)**

This is described in detail below:

1) compared to wild type p97, the p97-E578Q mutant has an enhanced PARP1 interaction under trapping conditions, consistent with the E578Q mutant causing a substrate trapping effect (new **Figure 4G**);

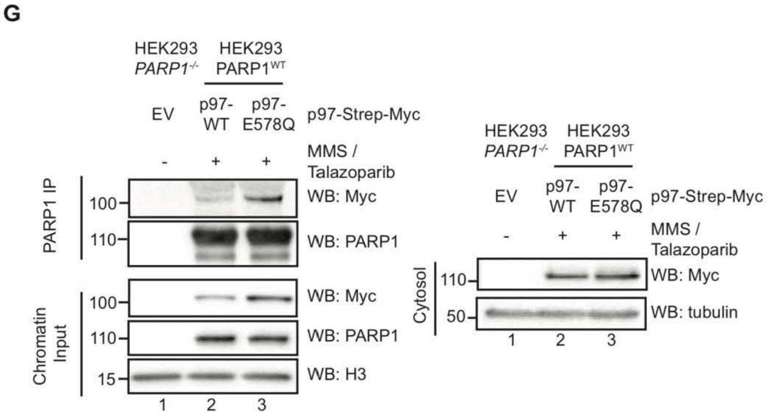

Figure 4G. HEK293 cells expressing either doxycycline-inducible p97-Myc epitope-tagged cDNA or a p97 dominant negative p.E578Q mutant-Myc cDNA were transfected with a FLAG-PARP1-expressing cDNA construct. Cells were exposed to 1 μg/ml doxycycline for 16 h and subsequently exposed to MMS + talazoparib to induce PARP1 trapping. Cells were fractionated and PARP1 immunoprecipitated from the chromatin fraction using PARP-Trap beads. compared to wild type p97, the p97-E578Q mutant has an enhanced PARP1 interaction under trapping conditions, consistent with the E578Q mutant causing a substrate trapping effect.

2) The p97-E578Q/PARP1 interaction is trapping-dependent, being much reduced in cells expressing the trapping-defective PARP1$^{del\ p.119K120S}$ mutant (**Supplementary Figure 4D):**

D

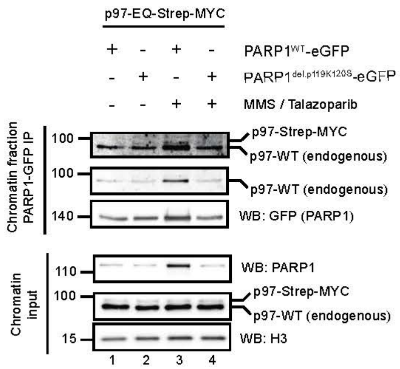

Supplementary Figure 4D. CAL51 PARP1^{WT}-eGFP or PARP1$^{del.p.119K120S}$-eGFP expressing cells were transfected with p97-E578Q -Strep-Myc-expressing cDNA construct. Eighteen hours later, cells were exposed to MMS + talazoparib to induce PARP1 trapping. Cells were fractionated into chromatin and soluble fraction and PARP1 immunoprecipitated from the chromatin fraction using GFP-Trap beads and the association with p97 was investigated by Western blotting.

3) The p97-E578Q mutant colocalises with PARP1 under PARP1 trapping conditions (**Supplementary Figure 4E).**

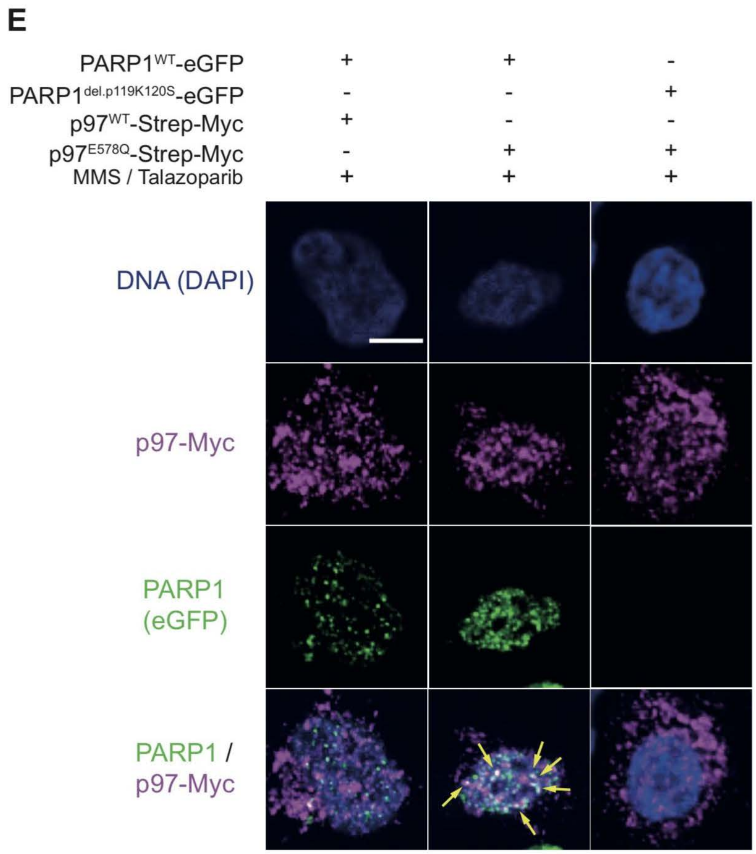

Supplementary Figure 4E. p97-E578Q mutant colocalises with PARP1 under trapping conditions. CAL51 PARP1WT-eGFP and PARP1del.p.119K120S-eGFP cells were transfected with p97-WT-Strep-MYC or p97-E578Q-Strep-MYC constructs and then subsequently exposed to MMS + talazoparib to induce PARP1 trapping. Cell were then pre-extracted and fixed, and stained for trapped PARP1 and MYC (as described in 30). The p97-E578Q-mutant colocalised with the trapped PARP1 signal in CAL51 PARP1WT-eGFP cells (yellow arrows) whereas PARP1del.p.119K120S-eGFP were unable to form trapped PARP1 foci.

3) Shan Zha reported last year that PARP1 can be rapidly exchanged at sites of laser microirradiation even in the presence of PARP inhibition (PMID: 32890402). These results suggest either that there are activities involved in removing PARP1 from DNA damage sites (such as p97), or that trapping is largely an in vitro phenomenon. Is p97 involved in promoting PARP1 exchange at DNA damage sites using FRAP?

Our response: This is an interesting point. We have taken on board the reviewer's suggestion and conducted a FRAP experiment on UV-laser-induced trapped PARP1 (new **Supplementary Figure 5K,L**). The experiments were conducted and quantified similarly to the manuscript described above. Similar to the observations described by Zha et al, we observed a similar recovery of the fluorescent signal at the spot of microirradiation in the presence and absence of talazoparib. The recovery of the fluorescent signal in the presence of talazoparib plus the p97 inhibitor CB-5083 was somewhat slower than for talazoparib alone (talazoparib $t_{1/2}$ = 4.9 ± 1.3 s to talazoparib + CB-5083 $t_{1/2}$ = 7.8 ± 1.4 s, two-sided t-test p-value < 0.05).

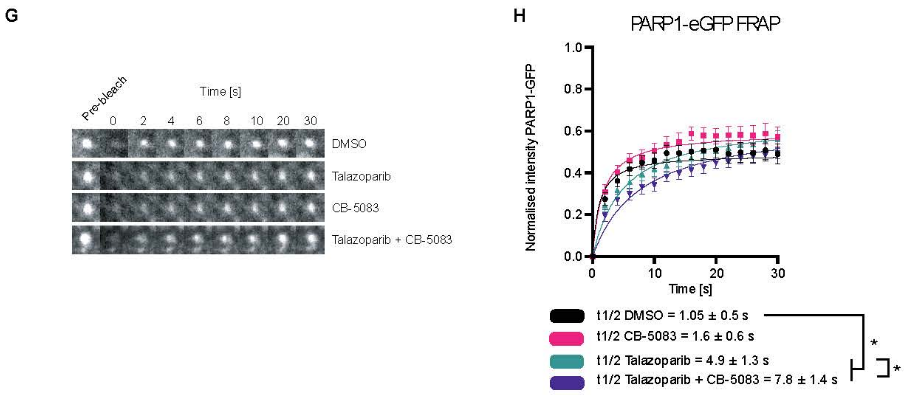

Supplementary Figure 5 K, L. PARP1[WT]-eGFP FRAP in the presence of PARP inhibitor and p97 inhibitor. CAL51 PARP1[WT]-eGFP cells were subjected to UV-microirradiation and the accumulation of PARP1[WT]-eGFP at UV-laser induced DNA damage sites was monitored over time in the presence of DMSO (vehicle), talazoparib, the p97 inhibitor CB-5083 or a combination of talazoparib and CB-5083. At the maximum time of PARP1[WT]-eGFP recruitment (typically 1 min after microirradiation) the focus was bleached with a 488 nm laser and the recovery of the PARP1[WT]-eGFP was

monitored over time as described in[36]. **K.** Image montages of the microirradiation site over time for the corresponding drug treatment arms. **L.** A quantification of the FRAP described in (K). The fluorescent signal was scaled according to the maximum PARP1^{WT}-eGFP immediately prior the photobleach to (equalling 1) and the signal immediately after photobleach (0), as in[36]. The FRAP data was fitted with one site-specific binding model of non-linear regression and the extra sum of squares F test was used to calculate the $t_{1/2}$. The significance was determined with a two-sided t-test from two independent experiments, where 10 to 12 cells were quantified for each condition. * - p-value < 0.05.

ref[36]: Shao, Z. *et al.* Clinical PARP inhibitors do not abrogate PARP1 exchange at DNA damage sites in vivo. *Nucleic Acids Res* **48**, 9694-9709 (2020).

It is very important to stress here that the nature of the DNA damage (and possibly the proteins recruited to the site of damage) caused by trapping conditions (i.e. MMS + talazoparib) might be very different to those caused by UV-laser. Therefore, we feel that even though the above FRAP data could be taken as being consistent with p97 playing a role in the resolution of PARPi-induced damage, it might also be somewhat premature to over-interpret this data until there is a better understanding of what the “exchange at trapped site” actually means. Given this, we have included the FRAP data in the revised manuscript for information but have not gone so far as to state that it reflects the same molecular events, as say, the chromatin PARP trapping data or the PARP1-γH2AX PLA data etc..

4) In most systems, p97-dependent removal of ubiquitylated proteins from chromatin results in protein degradation. On prediction is therefore that PARP1 is degraded after talazoparib treatment in a p97-dependent manner. From the figures in the paper (e.g. Fig 2) it does not appear that this is the case, why?

Our response: We thank the reviewer for raising this point. Whilst p97 certainly promotes the degradation of many of its cognate substrates, this is certainly not the case for all. As summarised elsewhere (van den Boom J., Mol Cell 2017; Ye Y., Front. Mol. Biosci. 2017; Torrecilla I., Philos Trans R Soc Lond, 2017) p97 can also recycle substrates. For example, Aurora B (Ramadan K., Nature, 2007), yeast transcriptional repressor alpha 2 (Wilcox A., Nat Cell Biol, 2009), Ub-LexA-VP16 (Ndoja A., Mol Cell, 2014) are extracted from the chromatin but not degraded.

This could very well be the case for PARP1 as well. Given this we have now updated the main text of the manuscript to state:

“Secondly, in most systems the p97-dependent removal of ubiquitylated proteins is coupled to proteasomal degradation, but this was not the case for PARP1. P97 is also known to participate in substate recycling, as is the case for Aurora B[8], yeast transcriptional repressor alpha[19] and Ub-LexA-VP16[18]. This raises the possibility that PARP1 might also be a p97 substate that is recycled instead of degraded.”

5) The Altmeyer group has linked the E3 ubiquitin ligase TRIP12 to the modulation of trapped PARP1. However, this current manuscript proposes a completely different system for modulating PARP1 (PIAS4-dependent sumoylation following RNF4-dependent ubiquitylation). Can the authors address this? Are there multiple systems for PARP1 modulation, cell type specificity or is there a discrepancy?

Our response: We thank the reviewer for this comment. One interesting aspect of the Altmeyer work on TRIP12, is that TRIP12 is a PAR-dependent E3 ubiquitin ligase i.e. it associates with PARP1 only when PAR is present and not in the presence of PARPi, which as well as trapping PARP1, also inhibit PARylation. This suggests that TRIP12 is not necessarily involved in the processing of trapped PARP1 *per se* but is more involved in the normal removal of PARP1 from the chromatin in the absence of PARPi. As such, we think the mechanism we describe, which involves PIAS4, RNF4 and p97, is distinct from that proposed by Altmeyer and colleagues, with the PIAS4/RNF4/p97 mechanism operating in the presence of PARPi-trapped PARP1, where PAR is largely undetectable.

To acknowledge this, we have now revised the discussion of the manuscript to state:

“In addition to RNF4, recent work identified the E3 ubiquitin ligase TRIP12’s role in ubiquitinating and regulating PARP1 turnover[42]. Importantly, TRIP12 is recruited to PARP1, in a PAR-dependent manner[42]. Under PARP1 trapping conditions used in this work, PAR is largely absent (for example, Figure 4B, H), suggesting that the SUMO/ubiquitin cascade (Figure 2-4) that drives p97 to modulate the PARPi inhibitor-induced trapped complex appears independent of TRIP12.”

We also assessed experimentally the possibility that TRIP12 could modulate the PARP1-p97 interaction. As shown below, we found that the PARP1-p97

interaction caused by PARP1 trapping (assessed by PLA) was not increased by TRIP12 siRNA, suggesting that TRIP12 might not be involved in this particular interaction:

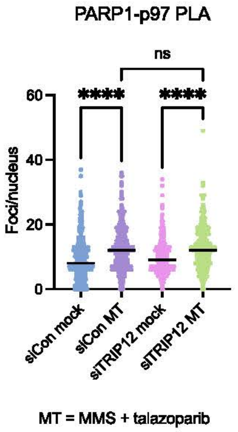

Rebuttal Figure. TRIP12 does not influence the PARP1-p97 interaction. CAL51 cells were transfected with control or TRIP12-targeting SMART pool of siRNAs. 48 hours post transfection the cells were exposed to trapping conditions and after fixation the PARP1-p97 interaction was determined by PLA. TRIP12 depletion did not lead to disruption of the PARP1-p97 interaction, indicating that its ubiquitin E3 activity is dispensable for this process. **** - p-value < 0.0001 ANOVA. The transfection efficiency was monitored by the cytotoxicity caused by PLK1 siRNA.

6) In their trap/chase experiments (Fig 4), why are the authors removing PARPi in their chase? Removing talazoparib allows PARP1 to be reactivated, DNA repair to resume, etc? Would it not make more sense to maintain the trapping agent and examine the release of PARP1 from DNA under those conditions?

Our response: We apologise that we did not make this clear in the first submission. Talazoparib was not present in the chase experiments in initial PIAS4$^{-/-}$ and RNF4$^{-/-}$ cells, but present in the experiments shown in the remaining panels of Figure 4. We have now also conducted the trap-chase experiments in the *PIAS4*$^{-/-}$ and *RNF4*$^{-/-}$ cells in the presence of PARPi and updated the figure. The results are presented in updated **Figure 5 B,C**.

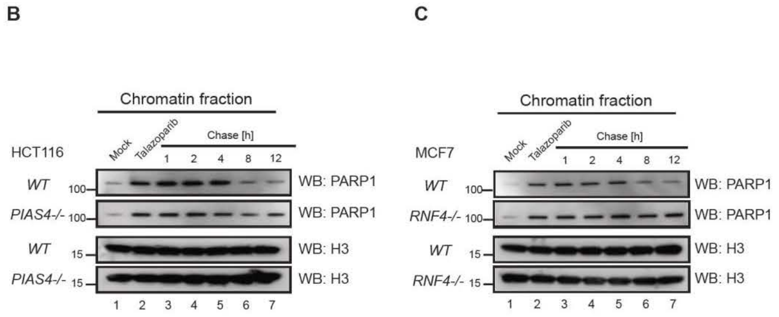

Figure 5 B. Trapped PARP1 is processed in a PIAS4-dependent manner. A trap-chase experiment in HCT116 wild-type or *PIAS4*$^{-/-}$ cells. After PARP1 trapping (100 nM talazoparib + 0.01% MMS), cells were chased in talazoparib-containing media. Samples were collected at indicated time point for chromatin fractionation and Western blotting. In wild type cells, the amount of chromatin-bound PARP1 decreased over the chase period, but in *PIAS4*$^{-/-}$ cells, this process was delayed (see residual PARP1 at 8 and 12 hrs). **C**. Trapped PARP1 is processed in a RNF4-dependent manner. A trap-chase experiment in MCF7 wild-type or RNF4$^{-/-}$ cells similar to (B). The amount of chromatin-bound PARP1 decreases over the chase period. In *RNF4*$^{-/-}$ cells, this process was delayed.

7) I do not fully understand why the CB-5083/talazoparib combination is selective for BRCA-deficient cells and tumors? What is the basis of this selectivity? Could it be that this is due to effects of p97 in processes unrelated to PARP1 trapping?

Our response: Our work and that of others has previously shown that he extent of *BRCA*-gene synthetic lethality of a PARPi is determined by the degree of PARP1 trapping generated. For example, when assessed head-to-head in BRCA1 or BRCA2 isogenic systems, PARP inhibitors that have similar effects on inhibiting PARylation (i.e. EC_{50} all in the 2-6 nM range) elicit different scales of synthetic lethality, which correlates better with the relative trapping abilities of PARP inhibitors than with their ability to inhibit PARylation (Shen et al Clin Cancer Res. 2013 Sep 15;19(18):5003-15). Taking this into account, we presume that agents such as p97 inhibitors that enhance the amount of trapped PARP1, would likely enhance the scale of synthetic lethality seen in a BRCA isogenic system.

8) Finally, the authors rely nearly exclusively on p97 inhibitors. As a means to

fully exclude off-target effect of the compounds, key experiments could be confirmed with a p97 dominant-negative protein.

Our response: As partially detailed in our response to point (2) above, we have now carried out the key experiments using the p97 dominant negative mutant. These show that:

1) compared to wild type p97, the p97-E578Q mutant has an enhanced PARP1 interaction under trapping conditions, consistent with the E578Q mutant causing a substrate trapping effect (new **Figure 4G**);

2) the p97-E578Q/PARP1 interaction is trapping-dependent, being much reduced in cells expressing the trapping-defective PARP1$^{del\ p.119K120S}$ mutant (new **Supplementary Figure 4D**);

3) the p97-E578Q mutant colocalises with PARP1 under PARP1 trapping conditions (**Supplementary Figure 4E).**

In addition to the above described in (2), we also show that:

4) the p97-E578Q mutant leads to accumulation of trapped PARP1 suggesting that the p97-E578Q mutant blocks trapped PARP1 removal. This was observed via cellular fractionation (new **Supplementary Figure 5E**) and pre-extraction immunofluorescence of chromatin bound PARP1 (as described in[30]) (new **Supplementary Figure 5F, G**).

E

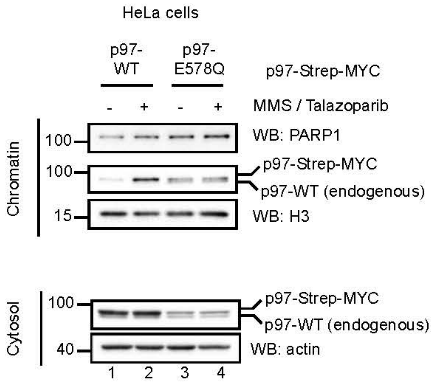

Supplementary Figure 5E. HeLa cells were transfected with either p97-Strep-MYC epitope-tagged cDNA or a p97 dominant negative E578Q mutant-Strep-MYC cDNA. Sixteen hours later, cells were exposed to MMS + talazoparib to induce PARP1 trapping. Cells were fractionated into chromatin and soluble fraction and PARP1 in the chromatin fraction detected by western blotting.

F

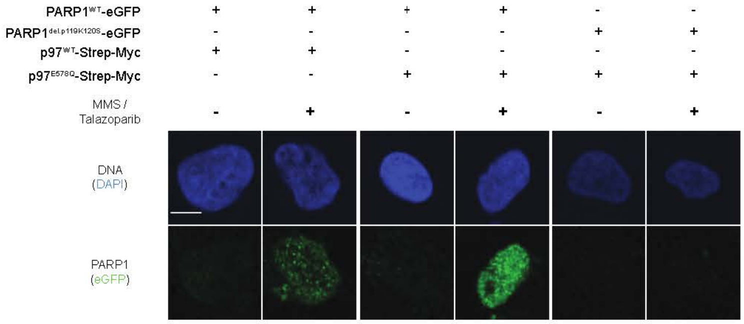

Supplementary Figure 5F. CAL51 cells expressing PARP1^{WT}-eGFP or PARP1$^{del.p119K120S}$-eGFP were transfected with p97^{WT}-Strep-MYC or p97^{E578Q}-Strep-MYC-expressing construct. After trapping and pre-extraction the cells were fixed and imaged to quantify the amount of trapped PARP1 (green). The expression of the p97 E578Q mutant led to stronger chromatin retention of the PARP1^{WT}-eGFP, but not the trapping deficient PARP1$^{del.p119K120S}$-eGFP. The E578Q mutant also caused significantly stronger accumulation of trapped PARP1 than p97-WT when expressed.

G

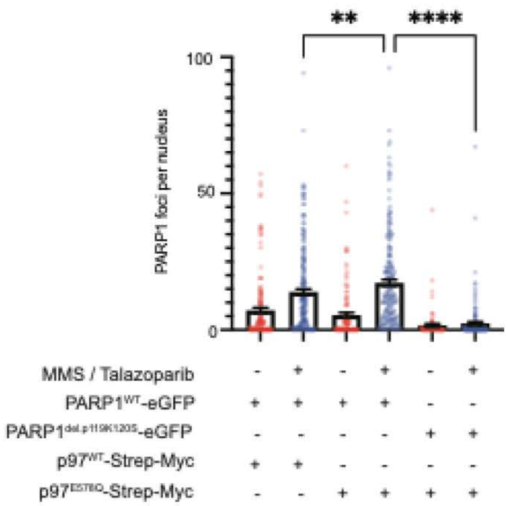

Supplementary Figure 5G. Quantification of the nuclear PARP1-eGFP foci of the experiment presented in (F). * - *p*-value <0.05, ** - *p*-value <0.01 on ANOVA.

End of Referee #2 comments

Reviewer #3:

Remarks to the Author:

Krastev et al., NCB review

In their study, "The ubiquitin-dependent ATPase p97 removes cytotoxic trapped PARP1 from chromatin", Krastev and colleagues address the issue of PARPi-mediated PARP1 trapping on DNA damaged chromatin, and the mechanism by which trapped PARP1 is normally released. They do this through a series of elegant, integrative techniques including RIME mass spectrometry, Apex2-proximity labelling and more.

Overall, I found this a very clear and well written study with a strong narrative making it very easy for the reader to understand, appreciate and follow. The overall of topic is of broad interest from a basic biological discovery standpoint, from a clinically-relevant drug discovery/mechanism standpoint, and from a "this is good science" general interest standpoint. The data are of high quality, and collectively support the authors conclusions. There are some occasions, detailed below, where I have concerns over statistical power of some key experimental imaging-based endpoints, which could be addressed by higher throughput and automated quantitation which is now standard.

1) The RIME and Apex2-based mass spectrometry screens for trapped-PARP1 interactors appears to me to be robust, and well controlled. I am not, per se, an expert in mass spectrometry however, and so will defer to other reviewers with regards to technical nuances of those experiments and the means by which strong hits were identified, and other protein hits disregarded. The logic, as presented, does seem to be sound, and it is advantageous to take forward a hit (p97) identified by both methods.

Our response: We thank the reviewer for taking the time to review our work. We have re-arranged the presentation of the MS results in order to incorporate the comments from the other reviewers and increase the clarity of presentation.

2) Is figure 2E mislabelled in terms of a (+) symbol for the MLN-7243 in lane three? As I read the results, this was mean to test the impact of the Ub inhibitor with and without the SUMO inhibitor (ML-792). But as described in figure, lanes 3 and 5 are identical but I am thinking should not be.

Our response: We thank the reviewer for spotting this mistake. Indeed lane 3 should have (-) as this is the sample cultured in trapping conditions, but without any SUMOylation or ubiquitylation inhibitor (neither MLN-7243 or ML-792). This mistake has now been corrected in the revised manuscript (**Figure 2F)**.

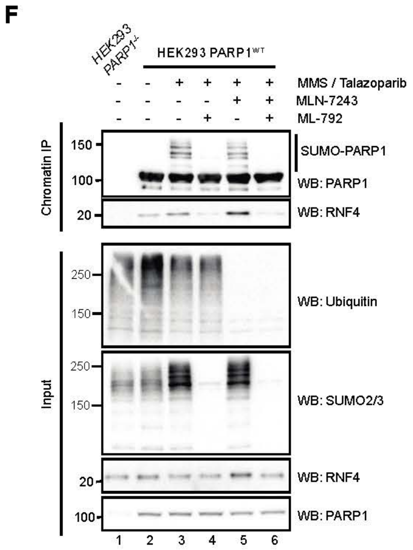

Figure 2F. PARP1 is modified and interacts with RNF4 in a SUMO-dependent manner. HEK293 WT or *PARP1$^{-/-}$* cells were exposed to trapping conditions either in the presence of ubiquitylation (5 µM MLN-7243) or SUMOylation (1 µM ML-792) inhibitors and endogenous PARP1 was immunoprecipitated from the chromatin fraction via PARP1-Trap nanobody. Western blotting for PARP1 revealed that the presence of the modified PARP1 isoforms was abrogated by ML-792 exposure (compare lanes 3 and 4), but not by MLN-7243 exposure (lane 3 vs. 5). This suggests these specific isoforms likely represent SUMOylated PARP1. Abrogating SUMOylation prevented the association between PARP1 and RNF4 (compare lanes 3 and 4), whereas inhibiting ubiquitinating stabilised the interaction.

3) In terms of the RNF4 depletion experiment in Fig S2E, while I agree that there was a visual reduction in the Ub-PARP-1 signal present here, the

natureresearch

depletion of RNF4 certainly does not completely ablate this, and the blots here are far less convincing in terms of Ub-PARP1 signal versus those shown in Fig 2B-D. To what extent (i.e. quantified data) did RNF4 suppress Ub-PARP1? I feel that this needs to be assessed, to give the reader a sense of the relative contribution of RNF4 to the process. The data in Fig 2G also suggests that loss of RNF4 produces partial effects, albeit to a stronger extent versus the siRNA-based method (perhaps to be expected). Complementation with wildtype and ubiquitylation-dead RNF4 would be prudent here, to consolidate the specificity of the effects being observed within cells. The in vitro experiments in Figures 2HI are very nice.

Our response: We thank the reviewer for these comments. We have spent a considerable effort clarifying the role of RNF4 in the ubiquitylation of PARP1 under trapping conditions, and in addition to using isogenic RNF4 wild type and knockout cells and RNF4-siRNA silencing to assess this, we have now added experiments where we complemented RNF4 knockout cells with either wild type or inactive RNF4 mutants (SIM deleted or catalytically inactive H156A mutant), shown as new **Figure 3E and F** and **Supplementary Figure 3I**.

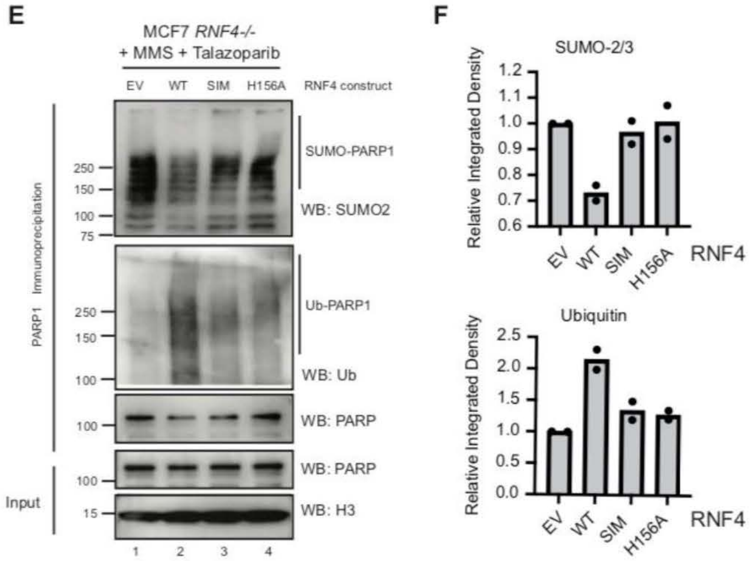

Figure 3. E. MCF7 *RNF4*$^{-/-}$ cells were transfected with indicated RNF4-expressing plasmids (EV: empty vector, WT: wild type, *SIM*: SUMO-interacting motif deleted, H156A catalytic dead) for 48 hours, followed by 30 min talazoparib (10 μM) treatment in the presence of 0.01 % MMS and whole cellular immunoprecipitation using PARP1 antibody. Immunoprecipitate and input were subjected to Western blotting and detected using indicated antibodies **F**. A quantification of the abundance of SUMO2/3 (top) and ubiquitin (bottom) modified PARP1 isoforms in the cells transfected as in (E). Two biological replicates are shown.

I

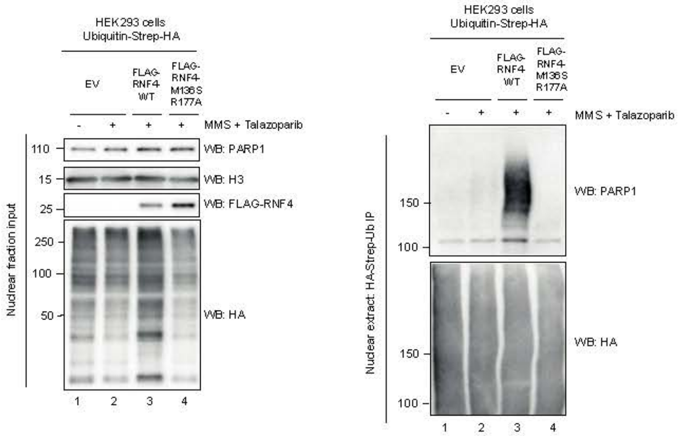

Supplementary Figure 3. I. Overexpression of RNF4-WT increased PARP1 ubiquitination under trapping conditions. HEK293 cells were transfected with Ubiquitin-Strep-HA in combination with either FLAG-RNF4-WT or M136S/R177A mutant (E2 binding mutant, dominant negative) expressing constructs. After treatment with MMS + Talazoparib, the cells were fractionated and ubiquitylated proteins were purified from the chromatin-bound fraction via Streptactin beads. Overexpression of RNF4-WT increased PARP1 ubiquitination whereas M136S/R177A was unable to facilitate PARP1 ubiquitination.

These experiments showed that RNF4 is responsible for up to 85–90 % (depending on the model system used) of the ubiquitylation of trapped PARP1.

In light of this data, we now state in the revised manuscript that:

"Interestingly, in *RNF4*$^{-/-}$ cells, while PARP1 ubiquitylation was decreased (confirming that RNF4 activity is responsible for this modification), PARP1 SUMOylation was increased (Figure 3D, Supplementary Figure 3E, F and G). Re-expressing wild-type RNF4 in *RNF4*$^{-/-}$ cells also reversed these effects, but this was not the case in cells expressing a SIM (SUMO-interacting motifs)-deleted[24] or catalytically inactive p.H156A mutant forms of RNF4 (Figure 3E and F, Supplementary Figure 3H). We also observed strong RNF4-dependent PARP1 ubiquitination by overexpressing wild-type RNF4 in

cells cultured in MMS + PARPi (Supplementary Figure 3I), an effect not seen when we expressed a dominant negative, E2 binding mutant form of RNF4 (p.M136S/R177A). Using the RNF4$^{-/-}$ cell lines and dominant negative mutants of RNF4, we found that RNF4 was responsible for up to 80-95% of the ubiquitylation on trapped PARP1. We also found that *RNF4* gene silencing (siRNA) reduced ubiquitylation of trapped PARP1 (Supplementary Figure 3J). Taken together, these data establish RNF4 as a STUbL E3 ligase for trapped PARP1."

4) Data in 3C, 3E, 3H need to encompass a quantitation of a lot more than 50 cells per condition, as indicated in the legends for 3C. From a statistical power perspective, these experiments do not meet the bar as is, although I agree the trends certainly support the authors conclusions and the accompanying western blots. Ideally this enumeration should be automated (the methods state some are manually scored some automated – but which are which? why mix methods? and either way manual is not ideal), and performed across several hundred cells per experimental replicate (it is not clear from methods how many times this was repeated, and if the 50 nuclei are from 1 experiment, or pooled from much lower cell numbers gathered from several). Either way, many hundreds of cells per replicate are needed here, with more detailed methodology on how cells are selected (or not) for enumeration. It is also not clear what the black/green bars in 3C or 3H represent (median, average?). Ideally geometric mean values with 95% confidence intervals should be displayed for raw datapoints across pooled experimental repeats, with dots set to a degree of transparency to show data density. Individual geometric mean foci number outcomes between experimental replicates can be compared in terms of statistics also. However, as this is a key piece of quantitative data for this study, this needs to be strengthened.

Our response: We thank the reviewer for these comments. In all our microscopy experiments we used manual inspection of the images alongside parallel automated analysis via CellProfiler pipeline and in the revised manuscript, provide the automated quantification alone, for consistency. For each experiment a power calculation was performed with G*power software. The number of nuclei to be scored in order to reach alpha<0.05 for a two-sided *t*-test was typically below 50.

In order to address reviewer #1 concerns, we have also included further controls in the PLA experiments. We have also presented the data as requested (geometric mean with 95% confidence intervals) in a standard colour scheme (For example, **Figure 3I**). Of note, the reanalysis of these data is still consistent with the previously stated conclusions.

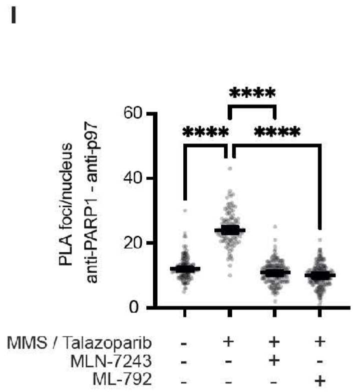

Figure 3I. PARP1/p97 co-localisation is reduced by ubiquitylation (5 μM MLN-7243) or SUMOylation (1 μM ML-792) inhibitors. Quantification of PARP1/p97 PLA foci/nucleus from CAL51 cell cultured in MMS/talazoparib plus the corresponding inhibitors. Either MLN-7243 or ML-792 reduces the number of PLA foci/nucleus. Quantification of the number of PLA foci/nucleus in n>200 cells from three independent experiments; black bars show the geometric mean ± 95 % CI, p-values were calculated with one-way ANOVA, **** - p < 0.0001.

5) Blots in Figure 4 are convincing but would be more so if quantified data merging several experiments of quantified PARP1 signal set relative to the trap condition were shown. It is hard to get a sense of the fold differences just by eye. The PLA data in 4D-F has the same issues as I described above with respect to Figure 3, and all those comments apply here.

Our response: We have now repeated the PARP1 trap-chase experiment in *PIAS4*$^{-/-}$ and *RNF4*$^{-/-}$ cells. The new data with improved loading controls is presented in new figure panels, **Figure 5 B and C**.

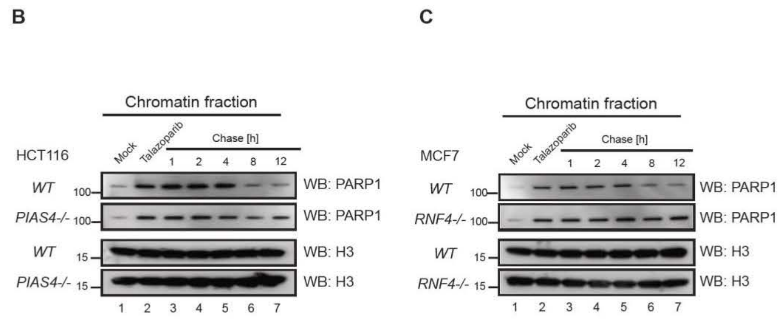

Figure 5 B. Trapped PARP1 is processed in a PIAS4-dependent manner. A trap-chase experiment in HCT116 wild-type or *PIAS4*$^{-/-}$ cells. After PARP1 trapping (100 nM talazoparib + 0.01% MMS), cells were chased in talazoparib-containing media. Samples were collected at indicated time point for chromatin fractionation and Western blotting. In wild type cells, the amount of chromatin-bound PARP1 decreased over the chase period, but in *PIAS4*$^{-/-}$ cells, this process was delayed (see residual PARP1 at 8 and 12 hrs). **C**. Trapped PARP1 is processed in a RNF4-dependent manner. A trap-chase experiment in MCF7 wild-type or RNF4$^{-/-}$ cells similar to (B). The amount of chromatin-bound PARP1 decreases over the chase period. In *RNF4*$^{-/-}$ cells, this process was delayed.

The experiments were repeated multiple times and quantified on the same gels as requested (loading controls shown; full gels included in the revised **Supplementary Figure 7**). The quantification of this data in now shown in **Supplementary Figure 5A and B**:

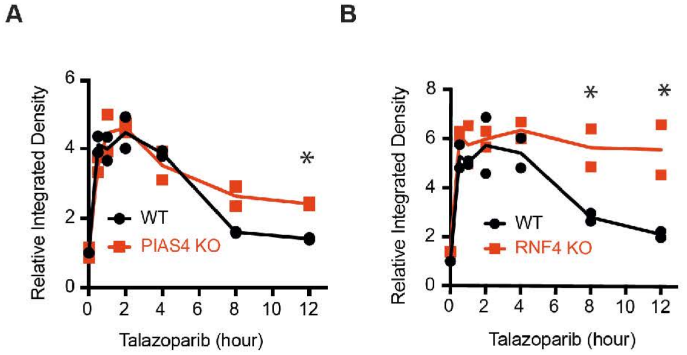

Supplementary Figure 5. A. Quantification of the chromatin bound PARP1 in Figure 5B. * - *p*-value <0.05 on a two-sided *t*-test. **B.** Quantification of the chromatin bound PARP1 in Figure 5C. * - *p*-value <0.05 on a two-sided *t*-test. Two biological replicas are displayed with individual points.

The PLA data was improved and re-plotted as per the previous comment.

An siRNA resistant RNF4 (wt and catalytic dead) addback would be prudent for data in 4F, with expression controls etc.

Our response. We have now employed the use of a dominant negative construct for RNF4 activity (M136S/R177A E2-binding mutant) as further validation of our model. As RNF4 functions as an obligate dimer, overexpression of this construct is able to inhibit RNF4-WT activity. Using this, we now show in new **Supplementary Figure 5B** that this mutant increases the amount of trapped PARP1 compared to cells expressing wild type RNF4. We also noted that this mutant reduced trapped PARP1-ubiquitination in new **Supplementary Figure 3I.**

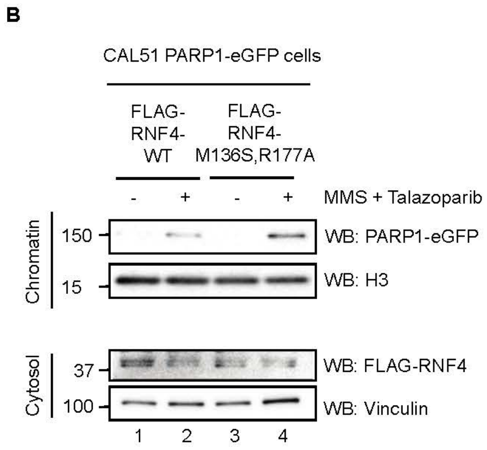

Supplementary Figure 5B. CAL51 PARP1^{WT}-eGFP cells were transfected with FLAG-RNF4-WT or FLAG-RNF-M136S,R177A (E2 binding mutant, dominant negative) constructs. 24 h after expression the cells were treated with trapping conditions and subsequently fractionated into soluble and chromatin-bound fractions. The expression of the FLAG-RNF-M136S,R177A construct led to higher amount of trapped PARP1 in the chromatin fraction.

I

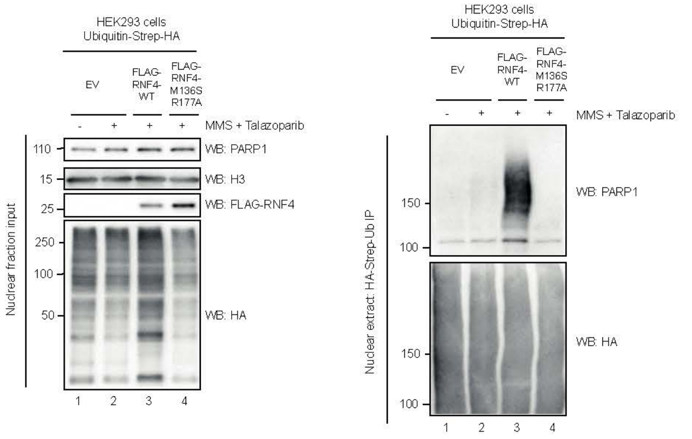

Supplementary Figure 3. I. Overexpression of RNF4-WT increased PARP1 ubiquitination under trapping conditions. HEK293 cells were transfected with Ubiquitin-Strep-HA in combination with either FLAG-RNF4-WT or M136S/R177A mutant (E2 binding mutant, dominant negative) expressing constructs. After treatment with MMS + Talazoparib, the cells were fractionated and ubiquitylated proteins were purified from the chromatin-bound fraction via Streptactin beads. Overexpression of RNF4-WT increased PARP1 ubiquitination, whereas M136S/R177A was unable to facilitate noticeable PARP1 ubiquitination.

A lot of the effects in 4G-I could be confounded by differences in cell cycle profile and/or DNA replication status in the cultures treated with different inhibitors during the experiment. Controls are needed to address whether this is an issue or not.

Our response. We have also now evaluated the cell cycle distribution in cells exposed to p97 inhibitor, as part of the trap-chase experiments - this new data is presented in new **Supplementary Figure 5I and J** and shows that whilst overnight exposure to talazoparib causes an accumulation in G_2/M as expected, neither CB-5083, nor CuET, as used in the trap-chase experiments, did not, suggesting alterations in the cell cycle do not provide a trivial explanation as to the changes in resolution of trapped PARP.

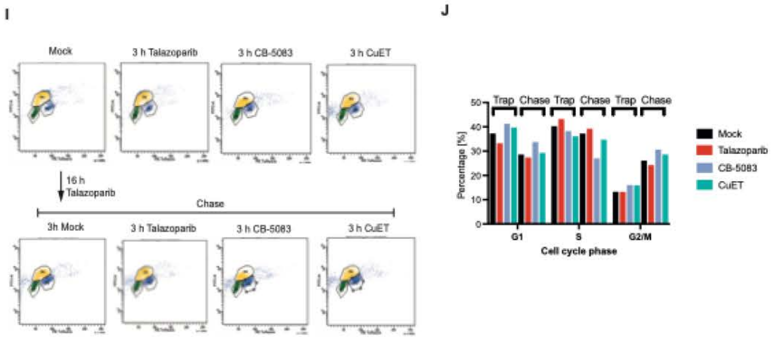

Supplementary Figure 5I. Cell cycle profiling for the experiment shown in Figure 5I. CAL51 cells were exposed to drugs as shown. One hour prior to fixation, 10 μM EdU was added to the media. After cells were removed from culture, EdU was stained by a click reaction with Alexa488-azide and DNA was stained by propidium iodide. **J**. A quantification of the G1, S and G2 populations from (I). The 3 h drug treatment arms do not alter the distribution of the cell cycle phases. In contrast, the 16 h Talazoparib treatment leads to an increase in the G2 population. This accumulation is not changed in the p97 inhibitor exposed arms in the chase period of 3 h.

To reflect this, we have also now added the following passage of text to the discussion:

"During our studies we considered the possibility that the effects on PARP1 trapping and PARP inhibitor cytotoxicity that we observed might not be due to an effect of p97 on trapped PARP1 (the key cytotoxic lesion) but could be due to p97 modulating other DNA repair processes. However, we think this unlikely for a number of reasons: (i) the concentration of p97 inhibitors used in PARP1 trapping and PARP1-interaction experiments (and the duration for which cells were exposed to these) did not, when used as single agents, elicit biomarkers of DNA damage such as γH2AX or RAD51 foci (Figure 5G-I) nor alterations in cell cycle dynamics (Supplementary Figure 5E,F), both of which might be expected if the concentrations of p97 inhibitors used to modulate PARP1 trapping were simply mediating their effects by eliciting DNA damage and/or causing defective DNA repair. Moreover, whilst p97 inhibitors enhanced PARP inhibitor sensitivity in a PARP1-dependent manner (Figure 6A,B), a p97 inhibitor did not alter sensitivity to either of two alkylating agents, MMS or temozolomide (Figure 6C,D). Although it is somewhat difficult to completely negate the possibility that the removal of other p97 substrates from chromatin could influence the repair of trapped PARP1, this data, when taken together with the data showing a SUMO- and ubiquitin dependent interaction between p97 and trapped PARP1, suggests that p97 does play a critical role in the resolution of this DNA lesion. "

Survival data are generally convincing, although a statistical evaluation of synergy is missing.

Our response: A statistical analysis of additivity/supra-additivity using Bliss Independence has now been added to the revised manuscript as **Supplementary Figure 6A,B**. Given we used agents that both inhibit the catalytic activity of their targets but also "trap" enzymes either in chromatin or with substrates, we used an analysis, Bliss, that makes few assumptions about the mode of inhibition of a target.

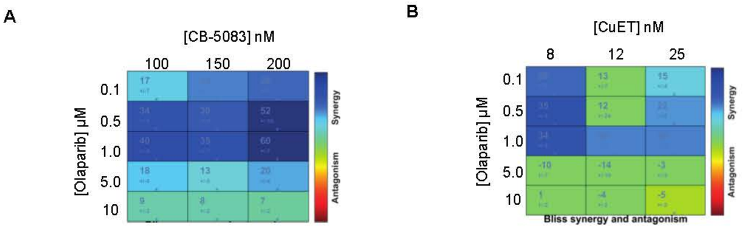

Supplementary Figure 6. A and **B.** Bliss synergy calculation, performed with the Combenefit software (CRUK Cambridge Institute), of the drug-response curves shown in Figure 6 B and C.

The model in Fig 4M presupposes that RNF4 is the only ligase contributing to trapped PARP1 Ub, which is not supported by the data, which shows some remaining Ub of trapped PARP1 in its absence. Model should reflect this and acknowledge the extent of contributions based on quantified data.

Our response. The model, now in **Figure 6I**, has been updated to include the text "RNF4 and other E3 ligase".

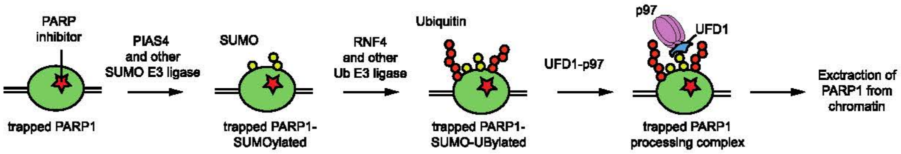

Figure 6I. A model of the processing of trapped PARP1. PARP1 trapped by the presence of PARPi on DNA is processed in a stepwise manner. It is initially SUMOylated in a PIAS4-dependant manner and subsequently ubiquitylated in an RNF4-dependent manner. p97 is recruited to the ubiquitin chains and binds via UFD1 and the ATPase activity of p97 extracts the modified PARP1 from the chromatin.

naturereearch

Minor points:

If possible, breaking up the multi-panel figures would be better. As they are, the data is cramped into four very crowded figures with exceptionally small text (e.g. Figure 1F...). Not sure if this was due to editorial constraints... in my experience NCB allows more than four primary figures and, if that is the case here, I would suggest making use of that and splitting these apart, so they are more digestible on the page.

Our response: We have taken this recommendation on board and expanded the data provided into six main display figures, accompanied by six supplementary figures.

Input controls for total Ubiquitin are important for Figure 2G, and are missing.

Our response: We have added these as requested in new **Supplementary Figures 3B and 3F**.

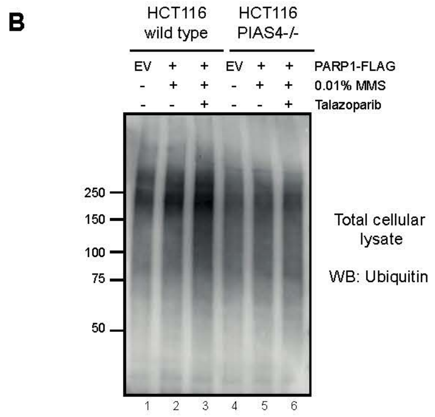

Supplementary Figure 3B. Western blotting for total ubiquitin input for Figure 3A.

F

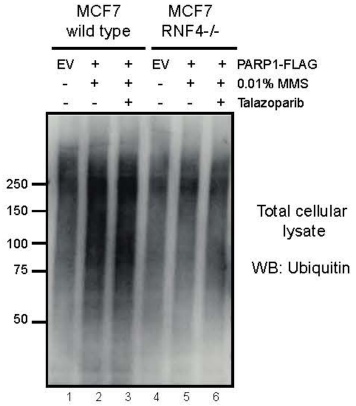

Supplementary Figure 3F. Western blotting for total ubiquitin input for Figure 3D.

Westerns for H3 in figure 4BC are pretty poor, being so over exposed to the extent of almost being meaningless as loading controls. Suggest a do-over.

Our response: As suggested, these experiments have been repeated and are now presented in the new **Figure 5B and C**.

B **C**

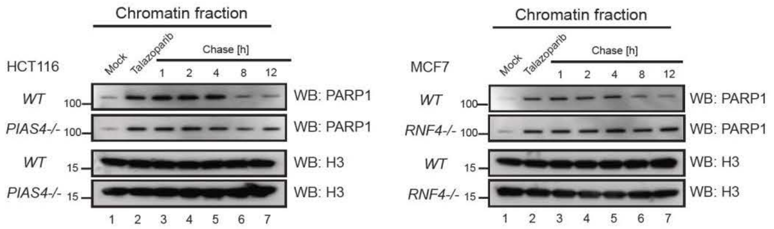

Figure 5 B. Trapped PARP1 is processed in a PIAS4-dependent manner. A trap-chase experiment in HCT116 wild-type or *PIAS4*$^{-/-}$ cells. After PARP1 trapping (100 nM talazoparib + 0.01% MMS), cells were chased in talazoparib-containing media. Samples were collected at indicated time point for chromatin fractionation and Western blotting. In wild type cells, the amount of chromatin-bound PARP1 decreased over the chase period, but in *PIAS4*$^{-/-}$ cells, this process was delayed (see residual PARP1 at 8 and 12 hrs). **C**. Trapped PARP1 is processed in a RNF4-dependent manner. A trap-chase experiment in MCF7 wild-type or RNF4$^{-/-}$ cells similar to (B). The amount of chromatin-bound PARP1 decreases over the chase period. In *RNF4*$^{-/-}$ cells, this process was delayed.

Statistical evaluations of data in Fig S4BD are missing.

Our response: Stats have been added.

The word 'extraction' is misspelled in Figure 4M.

Our response: Thank you for the note. Corrected.

End of Referee #3 comments

Decision Letter, first revision:

Date: 27th September 21 21:01:24
From: jie.wang@nature.com
To: Kristijan.Ramadan@oncology.ox.ac.uk
CC: ncb@springernature.com
Subject: Your manuscript, NCB-R44936A
Message:

Our ref: NCB-R44936A

27th September 2021

Dear Dr. Ramadan,

Thank you for submitting your revised manuscript "The ubiquitin-dependent ATPase p97 removes cytotoxic trapped PARP1 from chromatin" (NCB-R44936A). It has now been seen by the original referees and their comments are below. The reviewers find that the paper has improved in revision, and therefore we'll be happy in principle to publish it in Nature Cell Biology, pending minor revisions to satisfy the referees' final requests and to comply with our editorial and formatting guidelines.

As you will see, reviewer #1 continues to question the interpretation of the RIME and APEX analyses and does not believe trapping-dependent p97-PARP1 interaction can be firmly established based on these experiments. As this conclusion has additionally validated by an alternative approach in extended data fig 4D, we consider this concern can be addressed if you can 1) tone down the claim as suggested by this reviewer ("It is therefore recommended that the authors remove or adjust their statements regarding the trapping-dependent interaction between p97 and PARP1 on pages 10 (top paragraph) and 15

(middle paragraph) of the manuscript.”); 2) acknowledge the caveats associated the experiments; 3) and move extended data fig 4D (and other evidence supporting trapping-dependent interaction if available) to main figures. Another minor concern by this reviewer can be addressed textually.

The current version of your manuscript is in a PDF format. Please email us a copy of the main text including the method in an editable format (Microsoft Word or LaTex)-- we can not proceed with PDFs at this stage.

We are now performing detailed checks on your paper and will send you a checklist detailing our editorial and formatting requirements in about a week. Please do not upload the final materials and make any revisions until you receive this additional information from us.

Thank you again for your interest in Nature Cell Biology Please do not hesitate to contact me if you have any questions.

Sincerely,

Jie Wang, PhD
Senior Editor
Nature Cell Biology

Tel: +44 (0) 207 843 4924
email: jie.wang@nature.com

Reviewer #1 (Remarks to the Author):

In their revised manuscript, Krastev et al. included a number of additional data that satisfactorily address most of this reviewer´s comments on experimental issues. However, a major concern with the interpretation of the proteomics data remains, as well as a minor comment on the potential role of NPL4 - see reviewer responses to points #1 and #6 below. While this work is without doubt important from a translational point of view, it does not provide novel functional concepts. Even though trapped PARP1 has not been described as a p97 target before, it merely represents another example of a ubiquitin- and SUMO-modified, chromatin-bound p97 target. The involvement of UFD1, but not NPL4 in a p97-dependent process is not without precedent (e.g. Raman et al., doi 10.1016/j.molcel.2011.06.036). Also, it is not clear to this reviewer that PARP1 trapping actually causes DNA lesions (as claimed by the authors), as opposed to interfering with the repair of lesions, since most experiments relied on simultaneous MMS treatment to induce DNA damage. So it is not clear if the DNA damage repair pathway studied here differs from previously described repair pathways involving p97.

Specific reviewer responses can be found directly following the authors´ responses below.

****
Specific comments:

1. Fig. 1: The negative controls for the proteomics experiments appear to be flawed. For the proximity labeling, a cell line unable to undergo Apex labeling at all is a poor choice. Better alternatives would be cells expressing PARP1del.p119K120S-Apex2-eGFP or just Apex2-eGFP. Similarly, the correct background control for the RIME MS-IP experiments would be eGFP expressing cells, not PARP1 knockout cells. These flaws make the analysis of the results and the candidate selection somewhat questionable/arbitrary and may explain the largely unexpected collection of enriched GO terms (Fig. 1G). Also, the decision to focus on candidates with high MS scores but low PSM ratio +/- talazoparib appears counterintuitive, since it might simply favor highly abundant proteins such as SUMO or p97/VCP over proteins that were specifically enriched at trapped PARP1.

Our response: For the RIME MS experiments we used CAL51 PARP1–/– cells and derivatives of these expressing either PARP1WT-eGFP or PARP1del.p.119K120S-eGFP (the later being a PARP1-trapping defective allele) and isolated proteins using GFP-Trap beads, which harbour a nanobody with a high specificity towards GFP. In any immunoprecipitation experiment, a common problem is proteins that bind to the beads in a non-specific fashion. Given this, as an additional control, we also included an analysis of cells lacking eGFP – this could have been cells expressing a wild type PARP1 transgene but no eGFP or the parental cells of the transgenic cells described above, CAL51 PARP1–/– cells. Given the size of these experiments, we opted for the most informative control, which was the parental CAL51 PARP1–/– cells, which allowed us to remove from the analysis those proteins that non-specifically to the beads. We see no contradiction or insufficiency in using these cells as a control. As we demonstrated later in the manuscript, this overall approach identified proteins whose interactions were enhanced upon PARP1 trapping, suggesting some validity in the method taken.
To explain this better in the revised manuscript, we have now revised the main text, so that the reasoning for this approach is better set out. For example, the following text is now included:
“As PARP1 translocates to chromatin upon DNA damage, we first used RIME-based immunoprecipitation11, 13, to identify proteins associated with trapped PARP1 (Figure 1A). In these experiments, PARP1WT-eGFP and PARP1del.p.119K120S-eGFP expressing cells were exposed to PARP1 trapping conditions (methyl methanesulfonate (MMS) + talazoparib added to the tissue culture media) after which protein interactions were stabilised by formaldehyde crosslinking. MMS was used to create PARP1-binding DNA lesions, whereas the PARPi, talazoparib, was used to inhibit and trap DNA-bound PARP1. After trapping, chromatin-bound proteins were isolated and PARP1-associated complexes immunoprecipitated from this chromatin fraction using GFP-Trap beads, which harbour a nanobody with high specificity towards GFP. Immunoprecipitated proteins were then identified by mass spectrometry.

As a control, we also included an analysis of the parental CAL51 PARP1–/– cells lacking eGFP, in order to identify proteins that bind non-specifically to the GFP-Trap beads (Supplementary Figure 1B). These non-specific bead-binding proteins were removed from the list of proteins identified in the PARP1WT-eGFP and PARP1del.p.119K120S-eGFP expressing cells (detailed description in Methods).“
As for the APEX labelling experiment, we agree that the PARP1del.p119K120S-Apex2-eGFP cells would have been an ideal control. However, when we were generating our models, we did not manage to obtain a clone expressing this particular transgene (despite trying). To remove proteins that bind non-specifically to the beads, irrespective of biotinylation, we used cells that were not treated with biotin-phenol. This is, undoubtedly, the reason why we identified a larger number of PARP1-interacting proteins in the APEX2 labelling experiment, which we could not filter effectively based on a mutant control. For this reason, we used the PSM score as a filter, precisely because this indicates abundance of the protein in the identified complexes. Despite the longer PARP1 protein interaction list, GO enrichment analysis identified relevant processes e.g. base excision repair, as opposed to a random list of proteins.
To acknowledge the above, we have now revised the manuscript so that we now state:
“As an orthogonal MS approach, we employed Apex2-mediated proximity labelling. Apex2 peroxidase generates free radicals which in the presence of biotin-phenol (BP), biotinylates proteins within a ~20 nm radius; biotinylated proteins can then be purified via Streptavidin-binding. To identify proteins associated with trapped PARP1, we performed Apex2 labelling in cells expressing PARP1WT-Apex2-eGFP. Western blotting confirmed biotinylation of PARP1WT-APEX2-eGFP in the presence, but not absence of biotin-phenol, indicating effective labelling (Supplementary Figure 1C). The amount of labelled PARP1 was further increased when PARP1 labelling was conducted under trapping conditions (MMS + talazoparib) (Supplementary Figure 1C). Although we were unable to generate a clone with a trapping-defective PARP1 allele fused to Apex2- eGFP, we used PARP1WT-eGFP-expressing cells as a negative control for the labelling and purification (because of the absence of Apex2, these cells were unable to perform the biotinylation reaction). Biotinylated proteins were then purified under stringent conditions and analysed by mass spectrometry. Non-specific, background protein interactions with beads were removed by filtering the list of PARP1WT-Apex2-eGFP- interacting proteins against the list of proteins identified in PARP1WT-eGFP expressing cells (detailed analysis description in the Methods). As a result, we identified a higher number of proteins, 360, that associated with PARP1 than for RIME (either in the presence or absence of PARPi, Supplementary Table 3). A STRING network analysis, using a high stringency cut off (0.7) representing the trapped PARP1 interactome network (Supplementary Figure 1D), was enriched in proteins associated with one of the main DNA repair processes PARP1 is involved in, Base Excision Repair (BER), (e.g. PARP1 itself, PCNA, HMGB1, LIG3 and POLE, p-value<0.01, Supplementary Figure 1D, E), giving us high confidence in the analysis. Gene Ontology enrichment analysis also identified an enrichment in proteins involved in the spliceosome and ribosome biogenesis (Supplementary Table 4). We also identified a number of well- characterised PARylation targets (e.g. PCNA, NCL, FUS, ILF314, 15) strengthening the notion that we identified bona fide PARP1-proximal proteins.”

>>>Reviewer response: In the RIME MS experiments, the CAL51 PARP1–/– cells control for unspecific binding to the GFP trap beads, but not for unspecific binding to eGFP, whereas eGFP-expressing CAL51 PARP1+/+ cells would control for both and thus would be a better choice. This also applies to the GFP-IP experiments shown in Fig. 4. However, since the authors provide additional, independent evidence for the PARP1 - p97 interaction, this is acceptable.
By contrast, the negative control for the APEX approach is clearly insufficient. While cells not expressing Apex2 can control for unspecific binding to the streptavidin beads, they cannot control for unspecific biotin labeling of highly abundant proteins by Apex2. Since the whole point of the APEX approach is to infer the specific proximity of biotin-labeled proteins to the bait protein, an Apex2-expressing cell line (e.g. just expressing Apex2-eGFP) is an essential negative control. Otherwise, the unspecific labeling of highly abundant proteins cannot be excluded and could easily explain the correlation between abundance and labeling seen in Fig. 1H.

“Of note, the RIME results show p97/VCP to be actually depleted from trapped PARP1 (PSM ratio +/- talazoparib of 0.4 according to Suppl. Table 3), in contrast to the statement in line 226/227.”

Our response: In the revised manuscript, we have described our MS data analysis in more detail to make clear that p97 was identified in the APEX2 proximity labelling experiment, based on its abundance. Furthermore, p97 was also identified in the PARP1WT-eGFP, but not in the PARP1del.p.119K120S-eGFP RIME analysis, suggesting that the interaction is trapping dependent. This information was sufficient in order to prioritise p97 for further analysis.
To acknowledge the above, we have now revised the manuscript so that we now state:
“Among the most abundant labelled proteins were the ubiquitin-like modifier-activating enzyme 1 (UBA1), which has been previously implicated in ubiquitylation events at the sites of DNA damage16 and the transitional endoplasmic reticulum ATPase, p97 (also known as valosin containing protein, VCP), which acts as a central component of a ubiquitin-controlled process. p97’s ATP-dependent unfoldase activity extracts proteins from chromatin prior to their proteasomal degradation or recycling8-10, 17-19. Furthermore, p97, working with cofactors that often contain ubiquitin binding domains (UBDs), recognises client proteins via ubiquitylation events, mostly those involving lysine-48 (K48) and lysine-6 (K6)20, 21 ubiquitylation. p97 was also identified in the PARP1WT, but not in the PARP1del.p.119K120S RIME analysis, suggesting that this interaction is trapping- dependent.”

>>>Reviewer response: The identification of p97 in the APEX experiment is not convincing due to the lack of an appropriate negative control (see above). Regarding the RIME analysis, the author´s suggestion that the p97 interaction is trapping dependent is incorrect. New Suppl. Table 1 shows that the PSM ratio for p97 is 0.4, indicating that less p97 binds to wild-type PARP1 in the presence of Talazoparib, i.e. under trapping conditions. (Please note that the corresponding data point and label for p97 in Fig. 1E appears to be off - clearly above 0.5.)

In summary, the RIME results clearly show that SUMO1/2 are strong candidates (presumably because of their covalent attachment to PARP1), but this is not true for p97. It is therefore recommended that the authors remove or adjust their statements regarding the trapping-dependent interaction between p97 and PARP1 on pages 10 (top paragraph) and 15 (middle paragraph) of the manuscript.

2. Figs. 3/4: The PLA assays are in need of additional controls and quantifications. Fig. 3A shows that a background of PLA foci is observed in the presence of either the PARP1 or (more so) the p97/VCP antibody, even under non-stressed conditions. This background needs to be quantified, and the sum of the background foci must be compared to the "true" PLA foci in all quantifications for each condition. Since p97/VCP is likely to be recruited to/trapped at sites of DNA damage in the presence of MMS and/or CB-5083 independent of PARP1 trapping, a corresponding increase in the p97-antibody- only control is likely and has to be accounted for.

Our response: Thank you for pointing this out. We excluded these controls in the original submission so that the figures were not too complex but now include them in the revised manuscript as updated Figures 4D and E. The anti-PARP antibody, when used alone, produced almost no foci over background levels, whilst the p97 antibody produced a weak signal on its own – around 3-6 foci per nucleus. Importantly, when combined they produced an interaction signal with 10-15 foci per nucleus; this number increased to 30-40 foci in cells grown in trapping conditions and p97 inhibitors.
We have now conducted experiments with the anti-p97 antibody used alone as a control for potential p97 accumulation. This showed only a very modest effect
– the control conditions showing 3 ± 3 p97 PLA foci/nucleus, whilst CB-5083 exposure elicited 6.5 ± 4 foci per nucleus. These values are an order of magnitude lower than the p97-PARP1 PLA signal in cells grown in trapping conditions and p97 inhibitor (30 foci/nucleus). With this in mind, we have modified Figures 4D and E to reflect the modest change in the “p97 only control”. The PLA data have also been improved by increasing the number of cells counted and also formatted as per the comments of the other reviewers. Of note, there is no change in the interpretation of the data.

>>>Reviewer response: OK.

3. Fig. 4BC: PARP1 appears to be efficiently removed from chromatin even in the absence of PIAS4 or RNF4. How is this possible in light of the model shown in Fig. 4M? On a technical note, the H3 loading controls are heavily overexposed, precluding any quantitative analysis of the results. These experiments should be repeated and quantified in triplicates, with the samples from wildtype and knockout cells loaded on the same gel and with all loading controls in the linear detection range.

Our response: We have now repeated these experiments (new Figure 5B, C), where the chase was carried out in the presence of talazoparib (also requested by Reviewer #3): [...] The experiments were repeated multiple times and quantified on the same gels as requested (loading controls shown; full gels included in the revised Supplementary Figure 7). The quantification of this data in now shown in Supplementary Figure 5A and B: [...] As you can see, the absence of either PIAS4 or RNF4 causes a delay in the removal of PARP1 from the chromatin fraction. These data are in agreement with the biochemical experiments in Figure 3 and Supplementary Figure 3, where even though the absence of PIAS4 or RNF4 reduces the SUMOylation/ubiquitylation (respectively) of trapped PARP1, some residual PARP1 SUMOylation/ubiquitylation exists, suggesting that although PIAS4 and RNF4 are clearly important in this process, other SUMO E3 ligases and ubiquitin E3 ligases might also play a minor role.
With this in mind, we have now modified the model now presented in Figure 6I to include “PIAS4 and other SUMO E3 ligase(s)” and “RNF4 and other ubiquitin E3 ligase(s)”.

>>>Reviewer response: OK.

Minor points:

4. Fig. 1: The expression levels of the engineered PARP1 fusions used should be compared to the level of endogenous PARP1 in the parental cell line.

Our response: We have now assessed this and provided the data in new Supplementary Figure 1A. We also show in Supplementary Figure 1B the relative amounts of PARP1WT-eGFP and PARP1del p.119K120S-eGFP isolated in the RIME experiments.

>>>Reviewer response: OK.

5. Figs. 2/S2: The assignment of ubiquitylated and/or SUMOylated PARP1 species is not always clear. Sometimes these bands run at ≤150 kDa, sometimes at >>150 kDa. The authors should more clearly label/explain their identity.

Our response: We have now revised these figures to make clear where

ubiquitylated and/or SUMOylated species are. For example, Figure 2B is now modified to the following:
In addition, we have provided images of all the uncropped blots, which include molecular marker, in new Supplementary Figure 7.

>>>Reviewer response: OK.

6. It is somewhat surprising that the authors identified UFD1 but not NPL4 to be involved in PARP1 turnover, even though NPL4 appears to be critical for initializing the unfolding of ubiquitylated substrates by p97/VCP for subsequent proteasomal degradation. Did they identify UFD1 (but not NPL4) in their proteomics datasets? Does depletion of UFD1 (but not NPL4) result in the accumulation of trapped PARP1 on chromatin and/or in the reduction of p97 association with chromatin?

Our response: To address the first question, we did not detect UFD1 nor NPL4 in the original mass spec profiling, but of course this would not necessarily mean
that these proteins are not involved in processing trapped PARP1 (for example, the interaction could be transient and/or below the level of detection of mass spec.). To address the second question, we have now assessed the interaction between p97 and PARP1 and the total amount of trapped PARP1 in cells where either UFD1 or NPL4 were depleted (new Figure 4J). This experiment shows that whilst UFD1 depletion decreased the p97-PARP1 interaction and increased the amount of chromatin-associated trapped PARP1, NPL4 depletion did not.
In part, this experiment reproduces our original Figure 3I […] and is also replicated in new Supplementary Figure 5D [...]
We note that this might not be the first description of independent roles for UFD1 and NPL4 in p97 substrate processing. For example, CDT1 is removed from chromatin by p97 in a UFD1-dependent, but NPL4-independent manner (Ramen et al Mol Cell. 2011 Oct 7;44(1):72-84).
Nevertheless, we acknowledge that this is an important point to discuss in the manuscript and have therefore added the following to the revised manuscript:
“Regarding p97 recruitment, our data suggest that UFD1 is required for the recruitment of p97 to trapped PARP1 (Figure 4J). How exactly UFD1 recruits p97 to trapped PARP1 remains to be established. UFD1 is a well-known ubiquitin-chain reader as it possesses Ub-binding domain. In yeast, UFD1 has been shown to bind SUMO (in addition to ubiquitin) and to recruit p97/cdc48 to SUMOylated substrates39, 40. However, UFD1 binding to SUMO has never been demonstrated in mammalian cells. Our data presented here suggests that p97 recruitment to trapped PARP1 depends on RNF4- dependent ubiquitylation; it thus seems likely that UFD1 recruits p97 via its canonical role as an ubiquitin-chain reader, directly bridging p97 and the ubiquitin chains on p97 substrates, in this case ubiquitylated PARP1. We also note that although canonically, UFD1 is thought to function as an obligate heterodimer with NPL4, NPL4 silencing did not alter PARP1 trapping nor the PARP1-p97 interaction in the same way that UFD1 depletion did (Figure 4J). Whilst we are unable to entirely rule out a role for NPL4 in the processing of trapped PARP1, it is possible that this is a function, similar to the removal of CDT1 and other substrates from chromatin7, 32,9, that appears to be UFD1-specific.

>>>Reviewer response: While most of the presented evidence supports the view that PARP1 is an NPL4-independent target of p97, it should be noted that CuET is a specific inhibitor of NPL4 inducing nuclear clustering of NPL4, but not UFD1 or p97 (Skrott et al., doi 10.1038/nature25016). Consequently, the authors should discuss why CuET treatment phenocopies the treatment with the p97 inhibitor CB-5083.

7. The recent publication by the Dantuma lab on the role of PARylation of SUMOylated ATX3 during DNA DSB repair should be discussed (Pfeiffer et al, J Cell Sci 2021; doi:10.1242/jcs.247809).

Our response: We thank the reviewer for drawing out attention to this recent paper. We have now introduced a discussion on ATX3 in the main text as follows:
"Recently, the DUB ATXN3 which antagonises RNF4 ubiquitination activity at DNA damage sites, was shown to be recruited to micro-irradiation induced DNA damage in a PAR-dependent manner39. In combination with our work here, a tantalising hypothesis can be proposed whereby PARPi mediated PARP1 retention coupled with inhibition of ATXN3 recruitment is as a pre-requisite for RNF4-dependent trapped PARP1 ubiquitination. ."

>>>Reviewer response: OK.

8. Line 264/265: The E1 is the ubiquitin activating enzyme, not a ligase.

Our response: Thank you. This is now corrected.

>>>Reviewer response: OK.

Reviewer #2 (Remarks to the Author):

The authors did an exemplary job in revising this manuscript.

Reviewer #3 (Remarks to the Author):

In their revised study, "The ubiquitin-dependent ATPase p97 removes cytotoxic trapped PARP1 from chromatin", Krastev and colleagues present refined experiments and additional controls that substantially improve what was already, in my view, a strong study.

I commend the authors on their additional work, particularly the inclusion of quantified immunoblots, new add-back controls for RNF4 and dominant negative RNF4 (and p97), edits to improve clarity as to what was done, as well as the new lines of investigation that clarify how RNF4 is contributing to PARP1-Ub in this process.

Based on my original concerns and comments, I now consider this a sufficiently consolidated study and not inclined to ask for any further experiments or revisions. While there are naturally some questions that continue to arise from the revised experiments, I feel that this work is ready for the larger scientific community to view it.

Decision letter, final requests:

Date: 1st October 21 15:30:23
From: ncb@springernature.com
To: Kristijan.Ramadan@oncology.ox.ac.uk
CC: ncb@springernature.com
Subject: NCB: Your manuscript, NCB-R44936A
Message:

Our ref: NCB-R44936A

1st October 2021

Dear Dr. Ramadan,

Thank you for your patience as we've prepared the guidelines for final submission of your Nature Cell Biology manuscript, "The ubiquitin-dependent ATPase p97 removes cytotoxic trapped PARP1 from chromatin" (NCB-R44936A). Please carefully follow the step-by-step instructions provided in the attached file, and add a response in each row of the table to indicate the changes that you have made. Please also check and comment on any additional marked-up edits we have proposed within the text. Ensuring that each point is addressed will help to ensure that your revised manuscript can be swiftly handed over to our production team.

We would like to start working on your revised paper, with all of the requested files and forms, as soon as possible (preferably within one week). Please get in contact with us if you anticipate delays.

When you upload your final materials, please include a point-by-point response to any remaining reviewer comments.

If you have not done so already, please alert us to any related manuscripts from your group that are under consideration or in press at other journals, or are being written up for submission to other journals (see: https://www.nature.com/nature-research/editorial-policies/plagiarism#policy-on-duplicate-publication for details).

In recognition of the time and expertise our reviewers provide to Nature Cell Biology's editorial process, we would like to formally acknowledge their contribution to the external peer review of your manuscript entitled "The ubiquitin-dependent ATPase p97 removes cytotoxic trapped PARP1 from chromatin". For those reviewers who give their assent, we will be publishing their names alongside the published article.

Nature Cell Biology offers a Transparent Peer Review option for new original research manuscripts submitted after December 1st, 2019. As part of this initiative, we encourage our authors to support increased transparency into the peer review process by agreeing to have the reviewer comments, author rebuttal letters, and editorial decision letters published as a Supplementary item. When you submit your final files please clearly state in your cover letter whether or not you would like to participate in this initiative. Please note that failure to state your preference will result in delays in accepting your manuscript for publication.

Cover suggestions

As you prepare your final files we encourage you to consider whether you have any images or illustrations that may be appropriate for use on the cover of Nature Cell Biology.

Covers should be both aesthetically appealing and scientifically relevant, and should be supplied at the best quality available. Due to the prominence of these images, we do not generally select images featuring faces, children, text, graphs, schematic drawings, or collages on our covers.

We accept TIFF, JPEG, PNG or PSD file formats (a layered PSD file would be ideal), and the image should be at least 300ppi resolution (preferably 600-1200 ppi), in CMYK colour mode.

If your image is selected, we may also use it on the journal website as a banner image, and may need to make artistic alterations to fit our journal style.

Please submit your suggestions, clearly labeled, along with your final files. We'll be in touch if more information is needed.

natureresearch

Nature Cell Biology has now transitioned to a unified Rights Collection system which will allow our Author Services team to quickly and easily collect the rights and permissions required to publish your work. Approximately 10 days after your paper is formally accepted, you will receive an email in providing you with a link to complete the grant of rights. If your paper is eligible for Open Access, our Author Services team will also be in touch regarding any additional information that may be required to arrange payment for your article.

Please note that Nature Cell Biology is a Transformative Journal (TJ). Authors may publish their research with us through the traditional subscription access route or make their paper immediately open access through payment of an article-processing charge (APC). Authors will not be required to make a final decision about access to their article until it has been accepted. Find out more about Transformative Journals

Authors may need to take specific actions to achieve compliance with funder and institutional open access mandates. For submissions from January 2021, if your research is supported by a funder that requires immediate open access (e.g. according to Plan S principles) then you should select the gold OA route, and we will direct you to the compliant route where possible. For authors selecting the subscription publication route our standard licensing terms will need to be accepted, including our self-archiving policies. Those standard licensing terms will supersede any other terms that the author or any third party may assert apply to any version of the manuscript.

Please note that you will not receive your proofs until the publishing agreement has been received through our system.

For information regarding our different publishing models please see our Transformative Journals page. If you have any questions about costs, Open Access requirements, or our legal forms, please contact ASJournals@springernature.com.

Please use the following link for uploading these materials:
[REDACTED]

If you have any further questions, please feel free to contact me.

Best regards,

Nyx Hills

Staff
Nature Cell Biology

On behalf of

Jie Wang, PhD
Senior Editor
Nature Cell Biology

Tel: +44 (0) 207 843 4924
email: jie.wang@nature.com

Reviewer #1:
Remarks to the Author:
In their revised manuscript, Krastev et al. included a number of additional data that satisfactorily address most of this reviewer´s comments on experimental issues. However, a major concern with the interpretation of the proteomics data remains, as well as a minor comment on the potential role of NPL4 - see reviewer responses to points #1 and #6 below. While this work is without doubt important from a translational point of view, it does not provide novel functional concepts. Even though trapped PARP1 has not been described as a p97 target before, it merely represents another example of a ubiquitin- and SUMO-modified, chromatin-bound p97 target. The involvement of UFD1, but not NPL4 in a p97-dependent process is not without precedent (e.g. Raman et al., doi 10.1016/j.molcel.2011.06.036). Also, it is not clear to this reviewer that PARP1 trapping actually causes DNA lesions (as claimed by the authors), as opposed to interfering with the repair of lesions, since most experiments relied on simultaneous MMS treatment to induce DNA damage. So it is not clear if the DNA damage repair pathway studied here differs from previously described repair pathways involving p97.

Specific reviewer responses can be found directly following the authors´ responses below.

****
Specific comments:

1. Fig. 1: The negative controls for the proteomics experiments appear to be flawed. For the proximity labeling, a cell line unable to undergo Apex labeling at all is a poor choice. Better alternatives would be cells expressing PARP1del.p119K120S-Apex2-eGFP or just Apex2-eGFP. Similarly, the correct background control for the RIME MS-IP experiments would be eGFP expressing cells, not PARP1 knockout cells. These flaws make the analysis of the results and the candidate selection somewhat

questionable/arbitrary and may explain the largely unexpected collection of enriched GO terms (Fig. 1G). Also, the decision to focus on candidates with high MS scores but low PSM ratio +/- talazoparib appears counterintuitive, since it might simply favor highly abundant proteins such as SUMO or p97/VCP over proteins that were specifically enriched at trapped PARP1.

Our response: For the RIME MS experiments we used CAL51 PARP1–/– cells and derivatives of these expressing either PARP1WT-eGFP or PARP1del.p.119K120S-eGFP (the later being a PARP1-trapping defective allele) and isolated proteins using GFP-Trap beads, which harbour a nanobody with a high specificity towards GFP. In any immunoprecipitation experiment, a common problem is proteins that bind to the beads in a non-specific fashion. Given this, as an additional control, we also included an analysis of cells lacking eGFP – this could have been cells expressing a wild type PARP1 transgene but no eGFP or the parental cells of the transgenic cells described above, CAL51 PARP1–/– cells. Given the size of these experiments, we opted for the most informative control, which was the parental CAL51 PARP1–/– cells, which allowed us to remove from the analysis those proteins that non-specifically to the beads. We see no contradiction or insufficiency in using these cells as a control. As we demonstrated later in the manuscript, this overall approach identified proteins whose interactions were enhanced upon PARP1 trapping, suggesting some validity in the method taken.
To explain this better in the revised manuscript, we have now revised the main text, so that the reasoning for this approach is better set out. For example, the following text is now included:
"As PARP1 translocates to chromatin upon DNA damage, we first used RIME-based immunoprecipitation11, 13, to identify proteins associated with trapped PARP1 (Figure 1A). In these experiments, PARP1WT-eGFP and PARP1del.p.119K120S-eGFP expressing cells were exposed to PARP1 trapping conditions (methyl methanesulfonate (MMS) + talazoparib added to the tissue culture media) after which protein interactions were stabilised by formaldehyde crosslinking. MMS was used to create PARP1-binding DNA lesions, whereas the PARPi, talazoparib, was used to inhibit and trap DNA-bound PARP1. After trapping, chromatin-bound proteins were isolated and PARP1-associated complexes immunoprecipitated from this chromatin fraction using GFP-Trap beads, which harbour a nanobody with high specificity towards GFP. Immunoprecipitated proteins were then identified by mass spectrometry. As a control, we also included an analysis of the parental CAL51 PARP1–/– cells lacking eGFP, in order to identify proteins that bind non-specifically to the GFP-Trap beads (Supplementary Figure 1B). These non-specific bead-binding proteins were removed from the list of proteins identified in the PARP1WT-eGFP and PARP1del.p.119K120S-eGFP expressing cells (detailed description in Methods)."
As for the APEX labelling experiment, we agree that the PARP1del.p119K120S-Apex2-eGFP cells would have been an ideal control. However, when we were generating our models, we did not manage to obtain a clone expressing this particular transgene (despite trying). To remove proteins that bind non-specifically to the beads, irrespective of biotinylation, we used cells that were not treated with biotin-phenol. This is, undoubtedly, the reason why we identified a larger number of PARP1-interacting proteins in the APEX2 labelling experiment, which we could not filter effectively based on a mutant control. For this reason, we used the PSM score as a filter, precisely because this indicates abundance of

the protein in the identified complexes. Despite the longer PARP1 protein interaction list, GO enrichment analysis identified relevant processes e.g. base excision repair, as opposed to a random list of proteins.
To acknowledge the above, we have now revised the manuscript so that we now state:
“As an orthogonal MS approach, we employed Apex2-mediated proximity labelling. Apex2 peroxidase generates free radicals which in the presence of biotin-phenol (BP), biotinylates proteins within a ~20 nm radius; biotinylated proteins can then be purified via Streptavidin-binding. To identify proteins associated with trapped PARP1, we performed Apex2 labelling in cells expressing PARP1WT-Apex2-eGFP. Western blotting confirmed biotinylation of PARP1WT-APEX2-eGFP in the presence, but not absence of biotin-phenol, indicating effective labelling (Supplementary Figure 1C). The amount of labelled PARP1 was further increased when PARP1 labelling was conducted under trapping conditions (MMS + talazoparib) (Supplementary Figure 1C). Although we were unable to generate a clone with a trapping-defective PARP1 allele fused to Apex2- eGFP, we used PARP1WT-eGFP-expressing cells as a negative control for the labelling and purification (because of the absence of Apex2, these cells were unable to perform the biotinylation reaction). Biotinylated proteins were then purified under stringent conditions and analysed by mass spectrometry. Non-specific, background protein interactions with beads were removed by filtering the list of PARP1WT-Apex2-eGFP- interacting proteins against the list of proteins identified in PARP1WT-eGFP expressing cells (detailed analysis description in the Methods). As a result, we identified a higher number of proteins, 360, that associated with PARP1 than for RIME (either in the presence or absence of PARPi, Supplementary Table 3). A STRING network analysis, using a high stringency cut off (0.7) representing the trapped PARP1 interactome network (Supplementary Figure 1D), was enriched in proteins associated with one of the main DNA repair processes PARP1 is involved in, Base Excision Repair (BER), (e.g. PARP1 itself, PCNA, HMGB1, LIG3 and POLE, p-value<0.01, Supplementary Figure 1D, E), giving us high confidence in the analysis. Gene Ontology enrichment analysis also identified an enrichment in proteins involved in the spliceosome and ribosome biogenesis (Supplementary Table 4). We also identified a number of well- characterised PARylation targets (e.g. PCNA, NCL, FUS, ILF314, 15) strengthening the notion that we identified bona fide PARP1-proximal proteins.”

>>>Reviewer response: In the RIME MS experiments, the CAL51 PARP1–/– cells control for unspecific binding to the GFP trap beads, but not for unspecific binding to eGFP, whereas eGFP-expressing CAL51 PARP1+/+ cells would control for both and thus would be a better choice. This also applies to the GFP-IP experiments shown in Fig. 4. However, since the authors provide additional, independent evidence for the PARP1 - p97 interaction, this is acceptable.
By contrast, the negative control for the APEX approach is clearly insufficient. While cells not expressing Apex2 can control for unspecific binding to the streptavidin beads, they cannot control for unspecific biotin labeling of highly abundant proteins by Apex2. Since the whole point of the APEX approach is to infer the specific proximity of biotin-labeled proteins to the bait protein, an Apex2-expressing cell line (e.g. just expressing Apex2-eGFP) is an essential negative control. Otherwise, the unspecific labeling of

highly abundant proteins cannot be excluded and could easily explain the correlation between abundance and labeling seen in Fig. 1H.

“Of note, the RIME results show p97/VCP to be actually depleted from trapped PARP1 (PSM ratio +/- talazoparib of 0.4 according to Suppl. Table 3), in contrast to the statement in line 226/227.”

Our response: In the revised manuscript, we have described our MS data analysis in more detail to make clear that p97 was identified in the APEX2 proximity labelling experiment, based on its abundance. Furthermore, p97 was also identified in the PARP1WT-eGFP, but not in the PARP1del.p.119K120S-eGFP RIME analysis, suggesting that the interaction is trapping dependent. This information was sufficient in order to prioritise p97 for further analysis.
To acknowledge the above, we have now revised the manuscript so that we now state:
“Among the most abundant labelled proteins were the ubiquitin-like modifier-activating enzyme 1 (UBA1), which has been previously implicated in ubiquitylation events at the sites of DNA damage16 and the transitional endoplasmic reticulum ATPase, p97 (also known as valosin containing protein, VCP), which acts as a central component of a ubiquitin-controlled process. p97’s ATP-dependent unfoldase activity extracts proteins from chromatin prior to their proteasomal degradation or recycling8-10, 17-19. Furthermore, p97, working with cofactors that often contain ubiquitin binding domains (UBDs), recognises client proteins via ubiquitylation events, mostly those involving lysine-48 (K48) and lysine-6 (K6)20, 21 ubiquitylation. p97 was also identified in the PARP1WT, but not in the PARP1del.p.119K120S RIME analysis, suggesting that this interaction is trapping- dependent.”

>>>Reviewer response: The identification of p97 in the APEX experiment is not convincing due to the lack of an appropriate negative control (see above). Regarding the RIME analysis, the author´s suggestion that the p97 interaction is trapping dependent is incorrect. New Suppl. Table 1 shows that the PSM ratio for p97 is 0.4, indicating that less p97 binds to wild-type PARP1 in the presence of Talazoparib, i.e. under trapping conditions. (Please note that the corresponding data point and label for p97 in Fig. 1E appears to be off - clearly above 0.5.)
In summary, the RIME results clearly show that SUMO1/2 are strong candidates (presumably because of their covalent attachment to PARP1), but this is not true for p97. It is therefore recommended that the authors remove or adjust their statements regarding the trapping-dependent interaction between p97 and PARP1 on pages 10 (top paragraph) and 15 (middle paragraph) of the manuscript.

2. Figs. 3/4: The PLA assays are in need of additional controls and quantifications. Fig. 3A shows that a background of PLA foci is observed in the presence of either the PARP1 or (more so) the p97/VCP antibody, even under non-stressed conditions. This background needs to be quantified, and the sum of the background foci must be compared to the "true" PLA foci in all quantifications for each condition. Since p97/VCP is likely to be recruited to/trapped at sites of DNA damage in the presence of MMS

and/or CB-5083 independent of PARP1 trapping, a corresponding increase in the p97-antibody- only control is likely and has to be accounted for.

Our response: Thank you for pointing this out. We excluded these controls in the original submission so that the figures were not too complex but now include them in the revised manuscript as updated Figures 4D and E. The anti-PARP antibody, when used alone, produced almost no foci over background levels, whilst the p97 antibody produced a weak signal on its own – around 3-6 foci per nucleus. Importantly, when combined they produced an interaction signal with 10-15 foci per nucleus; this number increased to 30-40 foci in cells grown in trapping conditions and p97 inhibitors.
We have now conducted experiments with the anti-p97 antibody used alone as a control for potential p97 accumulation. This showed only a very modest effect
– the control conditions showing 3 ± 3 p97 PLA foci/nucleus, whilst CB-5083 exposure elicited 6.5 ± 4 foci per nucleus. These values are an order of magnitude lower than the p97-PARP1 PLA signal in cells grown in trapping conditions and p97 inhibitor (30 foci/nucleus). With this in mind, we have modified Figures 4D and E to reflect the modest change in the “p97 only control”. The PLA data have also been improved by increasing the number of cells counted and also formatted as per the comments of the other reviewers. Of note, there is no change in the interpretation of the data.

>>>Reviewer response: OK.

3. Fig. 4BC: PARP1 appears to be efficiently removed from chromatin even in the absence of PIAS4 or RNF4. How is this possible in light of the model shown in Fig. 4M? On a technical note, the H3 loading controls are heavily overexposed, precluding any quantitative analysis of the results. These experiments should be repeated and quantified in triplicates, with the samples from wildtype and knockout cells loaded on the same gel and with all loading controls in the linear detection range.

Our response: We have now repeated these experiments (new Figure 5B, C), where the chase was carried out in the presence of talazoparib (also requested by Reviewer #3): [...] The experiments were repeated multiple times and quantified on the same gels as requested (loading controls shown; full gels included in the revised Supplementary Figure 7). The quantification of this data in now shown in Supplementary Figure 5A and B: [...] As you can see, the absence of either PIAS4 or RNF4 causes a delay in the removal of PARP1 from the chromatin fraction. These data are in agreement with the biochemical experiments in Figure 3 and Supplementary Figure 3, where even though the absence of PIAS4 or RNF4 reduces the SUMOylation/ubiquitylation (respectively) of trapped PARP1, some residual PARP1 SUMOylation/ubiquitylation exists, suggesting that although PIAS4 and RNF4 are clearly important in this process, other SUMO E3 ligases and ubiquitin E3 ligases might also play a minor role.
With this in mind, we have now modified the model now presented in Figure 6I to include “PIAS4 and other SUMO E3 ligase(s)” and “RNF4 and other ubiquitin E3 ligase(s)”.

>>>Reviewer response: OK.

Minor points:

4. Fig. 1: The expression levels of the engineered PARP1 fusions used should be compared to the level of endogenous PARP1 in the parental cell line.

Our response: We have now assessed this and provided the data in new Supplementary Figure 1A. We also show in Supplementary Figure 1B the relative amounts of PARP1WT-eGFP and PARP1del p.119K120S-eGFP isolated in the RIME experiments.

>>>Reviewer response: OK.

5. Figs. 2/S2: The assignment of ubiquitylated and/or SUMOylated PARP1 species is not always clear. Sometimes these bands run at ≤150 kDa, sometimes at >>150 kDa. The authors should more clearly label/explain their identity.

Our response: We have now revised these figures to make clear where

ubiquitylated and/or SUMOylated species are. For example, Figure 2B is now modified to the following: In addition, we have provided images of all the uncropped blots, which include molecular marker, in new Supplementary Figure 7.

>>>Reviewer response: OK.

6. It is somewhat surprising that the authors identified UFD1 but not NPL4 to be involved in PARP1 turnover, even though NPL4 appears to be critical for initializing the unfolding of ubiquitylated substrates by p97/VCP for subsequent proteasomal degradation. Did they identify UFD1 (but not NPL4) in their proteomics datasets? Does depletion of UFD1 (but not NPL4) result in the accumulation of trapped PARP1 on chromatin and/or in the reduction of p97 association with chromatin?

Our response: To address the first question, we did not detect UFD1 nor NPL4 in the original mass spec profiling, but of course this would not necessarily mean

that these proteins are not involved in processing trapped PARP1 (for example, the interaction could be transient and/or below the level of detection of mass spec.). To address the second question, we have now assessed the interaction between p97 and PARP1 and the total amount of trapped PARP1 in cells where either UFD1 or NPL4 were depleted (new Figure 4J). This experiment shows that whilst UFD1 depletion decreased the p97-PARP1 interaction and increased the amount of chromatin-associated trapped PARP1, NPL4 depletion did not.
In part, this experiment reproduces our original Figure 3I […] and is also replicated in new Supplementary Figure 5D [...]
We note that this might not be the first description of independent roles for UFD1 and NPL4 in p97 substrate processing. For example, CDT1 is removed from chromatin by p97 in a UFD1-dependent, but NPL4-independent manner (Ramen et al Mol Cell. 2011 Oct 7;44(1):72-84).
Nevertheless, we acknowledge that this is an important point to discuss in the manuscript and have therefore added the following to the revised manuscript:
“Regarding p97 recruitment, our data suggest that UFD1 is required for the recruitment of p97 to trapped PARP1 (Figure 4J). How exactly UFD1 recruits p97 to trapped PARP1 remains to be established. UFD1 is a well-known ubiquitin-chain reader as it possesses Ub-binding domain. In yeast, UFD1 has been shown to bind SUMO (in addition to ubiquitin) and to recruit p97/cdc48 to SUMOylated substrates39, 40. However, UFD1 binding to SUMO has never been demonstrated in mammalian cells. Our data presented here suggests that p97 recruitment to trapped PARP1 depends on RNF4- dependent ubiquitylation; it thus seems likely that UFD1 recruits p97 via its canonical role as an ubiquitin-chain reader, directly bridging p97 and the ubiquitin chains on p97 substrates, in this case ubiquitylated PARP1. We also note that although canonically, UFD1 is thought to function as an obligate heterodimer with NPL4, NPL4 silencing did not alter PARP1 trapping nor the PARP1-p97 interaction in the same way that UFD1 depletion did (Figure 4J). Whilst we are unable to entirely rule out a role for NPL4 in the processing of trapped PARP1, it is possible that this is a function, similar to the removal of CDT1 and other substrates from chromatin7, 32,9, that appears to be UFD1-specific.

>>>Reviewer response: While most of the presented evidence supports the view that PARP1 is an NPL4-independent target of p97, it should be noted that CuET is a specific inhibitor of NPL4 inducing nuclear clustering of NPL4, but not UFD1 or p97 (Skrott et al., doi 10.1038/nature25016). Consequently, the authors should discuss why CuET treatment phenocopies the treatment with the p97 inhibitor CB-5083.

7. The recent publication by the Dantuma lab on the role of PARylation of SUMOylated ATX3 during DNA DSB repair should be discussed (Pfeiffer et al, J Cell Sci 2021; doi:10.1242/jcs.247809).

Our response: We thank the reviewer for drawing out attention to this recent paper. We have now introduced a discussion on ATX3 in the main text as follows:

“Recently, the DUB ATXN3 which antagonises RNF4 ubiquitination activity at DNA damage sites, was shown to be recruited to micro-irradiation induced DNA damage in a PAR-dependent manner39. In combination with our work here, a tantalising hypothesis can be proposed whereby PARPi mediated PARP1 retention coupled with inhibition of ATXN3 recruitment is as a pre-requisite for RNF4-dependent trapped PARP1 ubiquitination. .”

>>>Reviewer response: OK.

8. Line 264/265: The E1 is the ubiquitin activating enzyme, not a ligase.

Our response: Thank you. This is now corrected.

>>>Reviewer response: OK.

Reviewer #2:
Remarks to the Author:
The authors did an exemplary job in revising this manuscript.

Reviewer #3:
Remarks to the Author:
In their revised study, “The ubiquitin-dependent ATPase p97 removes cytotoxic trapped PARP1 from chromatin”, Krastev and colleagues present refined experiments and additional controls that substantially improve what was already, in my view, a strong study.

I commend the authors on their additional work, particularly the inclusion of quantified immunoblots, new add-back controls for RNF4 and dominant negative RNF4 (and p97), edits to improve clarity as to what was done, as well as the new lines of investigation that clarify how RNF4 is contributing to PARP1-Ub in this process.

Based on my original concerns and comments, I now consider this a sufficiently consolidated study and not inclined to ask for any further experiments or revisions. While there are naturally some questions that continue to arise from the revised experiments, I feel that this work is ready for the larger scientific community to view it.

Author Rebuttal, first revision:

Reviewer #1:

Remarks to the Author:

In their revised manuscript, Krastev et al. included a number of additional data that satisfactorily address most of this reviewer´s comments on experimental issues. However, a major concern with the interpretation of the proteomics data remains, as well as a minor comment on the potential role of NPL4 - see reviewer responses to points #1 and #6 below. While this work is without doubt important from a translational point of view, it does not provide novel functional concepts. Even though trapped PARP1 has not been described as a p97 target before, it merely represents another example of a ubiquitin- and SUMO-modified, chromatin-bound p97 target. The involvement of UFD1, but not NPL4 in a p97-dependent process is not without precedent (e.g. Raman et al., doi 10.1016/j.molcel.2011.06.036). Also, it is not clear to this reviewer that PARP1 trapping actually causes DNA lesions (as claimed by the authors), as opposed to interfering with the repair of lesions, since most experiments relied on simultaneous MMS treatment to induce DNA damage. So it is not clear if the DNA damage repair pathway studied here differs from previously described repair pathways involving p97.

Our response: We understand the referee's comments here but think what might not be acknowledged is that the vast majority of the literature concerning ubiquitin- and SUMO-modified, chromatin-bound p97 targets, centres on the removal of proteins from chromatin that are conducting their normal physiological function.

In our manuscript we show that this process is co-opted to remove a therapy-induced lesion (trapped PARP1) from chromatin. Importantly, other than the role of BRCA1/BRCA2 and HR, very little else was understood about how trapped PARP1 is processed by cells - our work now gives some insight into this process.

We also acknowledge that the involvement of UFD1, but not NPL4 in a p97-dependent process is not without precedent and, indeed we assessed this issue in light of a previous comment the referee made (see later). In the last iteration of the manuscript, we cited the relevant literature where UFD1-independent effects have been seen.

In respect to "whether PARP1 trapping actually causes DNA lesions (as claimed by the authors), as opposed to interfering with the repair of lesions" we hope we made clear in the original and revised manuscript that a PARPi that inhibits the catalytic activity of PARP1 (and which impairs PARP1-mediated DNA repair) but which fails to trap PARP1 in chromatin, veliparib, does not elicit PARP1 SUMOylation, ubiquitylation and p97 recruitment – conversely PARPi that do trap PARP1 do elicit PARP1 SUMOylation, ubiquitylation and p97 recruitment. Furthermore, modification of veliparib into the daughter compound UKT115, which does trap PARP1, also elicits PARP1 SUMOylation, ubiquitylation and p97 recruitment, making it beyond reasonable doubt that the effects we see are not explained by PARP1 trapping but better rationalised by PARP1's role in DNA repair.

Specific reviewer responses can be found directly following the authors´ responses below.

****
Specific comments:

1. Fig. 1: The negative controls for the proteomics experiments appear to be flawed. For the proximity labeling, a cell line unable to undergo Apex labeling at all is a poor choice. Better alternatives would be cells expressing PARP1del.p119K120S-Apex2-eGFP or just Apex2-eGFP. Similarly, the correct background control for the RIME MS-IP experiments would be eGFP expressing cells, not PARP1 knockout cells. These flaws make the analysis of the results and the candidate selection somewhat questionable/arbitrary and may explain the largely unexpected collection of enriched GO terms (Fig. 1G). Also, the decision to focus on candidates with high MS scores but low PSM ratio +/- talazoparib appears counterintuitive, since it might simply favor highly abundant proteins such as SUMO or p97/VCP over proteins that were specifically enriched at trapped PARP1.

Our response: For the RIME MS experiments we used CAL51 PARP1–/– cells and derivatives of these expressing either PARP1WT-eGFP or PARP1del.p.119K120S-eGFP (the later being a PARP1-trapping defective allele) and isolated proteins using GFP-Trap beads, which harbour a nanobody with a high specificity towards GFP. In any immunoprecipitation experiment, a common problem is proteins that bind to the beads in a non-specific fashion. Given this, as an additional control, we also included an analysis of cells lacking eGFP – this could have been cells expressing a wild type PARP1 transgene but no eGFP or the parental cells of the transgenic cells described above, CAL51 PARP1–/– cells. Given the size of these experiments, we opted for the most informative control, which was the parental CAL51 PARP1–/– cells, which allowed us to remove from the analysis those proteins that non-specifically to the beads. We see no contradiction or insufficiency
in using these cells as a control. As we demonstrated later in the manuscript, this overall approach identified proteins whose interactions were enhanced upon PARP1 trapping, suggesting some validity in the method taken.

To explain this better in the revised manuscript, we have now revised the main text, so that the reasoning for this approach is better set out. For example, the following text is now included:

"As PARP1 translocates to chromatin upon DNA damage, we first used RIME-based immunoprecipitation11, 13, to identify proteins associated with trapped PARP1 (Figure 1A). In these experiments, PARP1WT-eGFP and PARP1del.p.119K120S-eGFP expressing cells were exposed to PARP1 trapping conditions (methyl methanesulfonate (MMS) + talazoparib added to the tissue culture media) after which protein interactions were stabilised by formaldehyde crosslinking. MMS was used to create PARP1-binding DNA lesions, whereas the PARPi, talazoparib, was used to inhibit and trap DNA-bound PARP1. After trapping, chromatin-bound proteins were isolated and PARP1-associated complexes immunoprecipitated from this chromatin fraction using GFP-Trap beads, which harbour a nanobody with high specificity towards GFP. Immunoprecipitated proteins were then identified by mass spectrometry. As a control, we also included an analysis of the parental CAL51 PARP1–/– cells lacking eGFP, in order to identify proteins that bind non-specifically to the GFP-Trap beads (Supplementary Figure 1B). These non-

specific bead-binding proteins were removed from the list of proteins identified in the PARP1WT-eGFP and PARP1del.p.119K120S-eGFP expressing cells (detailed description in Methods).“

As for the APEX labelling experiment, we agree that the PARP1del.p119K120S-Apex2-eGFP cells would have been an ideal control. However, when we were generating our models, we did not manage to obtain a clone expressing this particular transgene (despite trying). To remove proteins that bind non-specifically to the beads, irrespective of biotinylation, we used cells that were not treated with biotin-phenol. This is, undoubtedly, the reason why we identified a larger number of PARP1-interacting proteins in the APEX2 labelling experiment, which we could not filter effectively based on a mutant control. For this reason, we used the PSM score as a filter, precisely because this indicates abundance of the protein in the identified complexes. Despite the longer PARP1 protein interaction list, GO enrichment analysis identified relevant processes e.g. base excision repair, as opposed to a random list of proteins.
To acknowledge the above, we have now revised the manuscript so that we now state:
“As an orthogonal MS approach, we employed Apex2-mediated proximity labelling. Apex2 peroxidase generates free radicals which in the presence of biotin-phenol (BP), biotinylates proteins within a ~20 nm radius; biotinylated proteins can then be purified via Streptavidin-binding. To identify proteins associated with trapped PARP1, we performed Apex2 labelling in cells expressing PARP1WT-Apex2-eGFP. Western blotting confirmed biotinylation of PARP1WT-APEX2-eGFP in the presence, but not absence of biotin-phenol, indicating effective labelling (Supplementary Figure 1C). The amount of labelled PARP1 was further increased when PARP1 labelling was conducted under trapping conditions (MMS + talazoparib) (Supplementary Figure 1C). Although we were unable to generate a clone with a trapping-defective PARP1 allele fused to Apex2- eGFP, we used PARP1WT-eGFP-expressing cells as a negative control for the labelling and purification (because of the absence of Apex2, these cells were unable to perform the biotinylation reaction). Biotinylated proteins were then purified under stringent conditions and analysed by mass spectrometry. Non-specific, background protein interactions with beads were removed by filtering the list of PARP1WT-Apex2-eGFP- interacting proteins against the list of proteins identified in PARP1WT-eGFP expressing cells (detailed analysis description in the Methods). As a result, we identified a higher number of proteins, 360, that associated with PARP1 than for RIME (either in the presence or absence of PARPi, Supplementary Table 3). A STRING network analysis, using a high stringency cut off (0.7) representing the trapped PARP1 interactome network (Supplementary Figure 1D), was enriched in proteins associated with one of the main DNA repair processes PARP1 is involved in, Base Excision Repair (BER), (e.g. PARP1 itself, PCNA, HMGB1, LIG3 and POLE, p-value<0.01, Supplementary Figure 1D, E), giving us high confidence in the analysis. Gene
Ontology enrichment analysis also identified an enrichment in proteins involved in the spliceosome and ribosome biogenesis (Supplementary Table 4). We also identified a number of well- characterised PARylation targets (e.g. PCNA, NCL, FUS, ILF314, 15) strengthening the notion that we identified bona fide PARP1-proximal proteins.”

>>>Reviewer response: In the RIME MS experiments, the CAL51 PARP1–/– cells control for unspecific binding to the GFP trap beads, but not for unspecific binding to eGFP, whereas eGFP-expressing CAL51 PARP1+/+ cells would control for both and thus would be a better choice. This also applies to the GFP-IP experiments shown in Fig. 4. However, since the authors provide additional, independent evidence for the PARP1 - p97 interaction, this is acceptable.

Our response: We thank the reviewer for taking the time to reassess our manuscript and are happy that they find the response to this query acceptable.

By contrast, the negative control for the APEX approach is clearly insufficient. While cells not expressing Apex2 can control for unspecific binding to the streptavidin beads, they cannot control for unspecific biotin labeling of highly abundant proteins by Apex2. Since the whole point of the APEX approach is to infer the specific proximity of biotin-labeled proteins to the bait protein, an Apex2-expressing cell line (e.g. just expressing Apex2-eGFP) is an essential negative control. Otherwise, the unspecific labeling of highly abundant proteins cannot be excluded and could easily explain the correlation between abundance and labeling seen in Fig. 1H.

Our response: In the revised main text of the manuscript (below), we have now addressed the caveat that we were unable to generate and use a mutant PARP1-APEX2 construct:

"A caveat to our work was our inability to generate a trapping-defective PARP1 mutant fused to Apex2-eGFP: this prevented us from using this as a control in the proximity labelling. Instead, we used the analysis of $PARP1^{WT}$-eGFP-expressing cells to filter out non-specific interactions with beads. As a result of this filtering, we identified a higher number of proteins, 360, that associated with PARP1 in our proximity labelling analysis than for RIME (either in the presence or absence of PARPi, Supplementary Table 3)."

Of note, the RIME results show p97/VCP to be actually depleted from trapped PARP1 (PSM ratio +/- talazoparib of 0.4 according to Suppl. Table 3), in contrast to the statement in line 226/227.

Our response: In the revised manuscript, we have described our MS data analysis in more detail to make clear that p97 was identified in the APEX2 proximity labelling experiment, based on its abundance. Furthermore, p97 was also identified in the PARP1WT-eGFP, but not in the PARP1del.p.119K120S-eGFP RIME analysis, suggesting that the interaction is trapping dependent. This information was sufficient in order to prioritise p97 for further analysis.

To acknowledge the above, we have now revised the manuscript so that we now state:
"Among the most abundant labelled proteins were the ubiquitin-like modifier-activating enzyme 1 (UBA1), which has been previously implicated in ubiquitylation events at the sites of DNA damage16 and the transitional endoplasmic reticulum ATPase, p97 (also known as valosin containing protein, VCP), which acts as a central component of a ubiquitin-controlled process. p97's ATP-dependent unfoldase activity extracts proteins from chromatin prior to their proteasomal degradation or recycling8-10, 17-19. Furthermore, p97, working with cofactors that often contain ubiquitin binding domains (UBDs), recognises client proteins via ubiquitylation events, mostly those involving lysine-48 (K48) and lysine-6 (K6)20, 21 ubiquitylation. p97 was also identified in the PARP1WT, but not in the PARP1del.p.119K120S RIME analysis, suggesting that this interaction is trapping- dependent."

>>>Reviewer response: The identification of p97 in the APEX experiment is not convincing due to the lack of an appropriate negative control (see above). Regarding the RIME analysis, the author´s suggestion that the p97 interaction is trapping dependent is incorrect. New Suppl. Table 1 shows that the

PSM ratio for p97 is 0.4, indicating that less p97 binds to wild-type PARP1 in the presence of Talazoparib, i.e. under trapping conditions. (Please note that the corresponding data point and label for p97 in Fig. 1E appears to be off - clearly above 0.5.)
In summary, the RIME results clearly show that SUMO1/2 are strong candidates (presumably because of their covalent attachment to PARP1), but this is not true for p97. It is therefore recommended that the authors remove or adjust their statements regarding the trapping-dependent interaction between p97 and PARP1 on pages 10 (top paragraph) and 15 (middle paragraph) of the manuscript.

Our response: In light of the reviewer's concerns, we have modified the text in order to tone down the statements about trapping-dependent interaction as follows:

Page 10: "p97 was also identified in the PARP1^{WT}, but not in the PARP1$^{del.p.119K120S}$ RIME analysis, strengthening the notion that it may interact with trapped PARP1."

Page 15: "Our mass spectrometry analysis suggested that PARP1 interacts with p97, an ATPase involved in the removal of ubiquitylated substrate proteins from chromatin."

2. Figs. 3/4: The PLA assays are in need of additional controls and quantifications. Fig. 3A shows that a background of PLA foci is observed in the presence of either the PARP1 or (more so) the p97/VCP antibody, even under non-stressed conditions. This background needs to be quantified, and the sum of the background foci must be compared to the "true" PLA foci in all quantifications for each condition. Since p97/VCP is likely to be recruited to/trapped at sites of DNA damage in the presence of MMS and/or CB-5083 independent of PARP1 trapping, a corresponding increase in the p97-antibody- only control is likely and has to be accounted for.

Our response: Thank you for pointing this out. We excluded these controls in the original submission so that the figures were not too complex but now include them in the revised manuscript as updated Figures 4D and E. The anti-PARP antibody, when used alone, produced almost no foci over background levels, whilst the p97 antibody produced a weak signal on its own – around 3-6 foci per nucleus. Importantly, when combined they produced an interaction signal with 10-15 foci per nucleus; this number increased to 30-40 foci in cells grown in trapping conditions and p97 inhibitors.
We have now conducted experiments with the anti-p97 antibody used alone as a control for potential p97 accumulation. This showed only a very modest effect – the control conditions showing 3 ± 3 p97 PLA foci/nucleus, whilst CB-5083 exposure elicited 6.5 ± 4 foci per nucleus. These values are an order of magnitude lower than the p97-PARP1 PLA signal in cells grown in trapping conditions and p97 inhibitor (30 foci/nucleus). With this in mind, we have modified Figures 4D and E to reflect the modest change in the "p97 only control". The PLA data have also been improved by increasing the number of cells counted and also formatted as per the comments of the other reviewers. Of note, there is no change in the interpretation of the data.

>>>Reviewer response: OK.

Our response: Thank you.

3. Fig. 4BC: PARP1 appears to be efficiently removed from chromatin even in the absence of PIAS4 or RNF4. How is this possible in light of the model shown in Fig. 4M? On a technical note, the H3 loading controls are heavily overexposed, precluding any quantitative analysis of the results. These experiments should be repeated and quantified in triplicates, with the samples from wildtype and knockout cells loaded on the same gel and with all loading controls in the linear detection range.

Our response: We have now repeated these experiments (new Figure 5B, C), where the chase was carried out in the presence of talazoparib (also requested by Reviewer #3): [...] The experiments were repeated multiple times and quantified on the same gels as requested (loading controls shown; full gels included in the revised Supplementary Figure 7). The quantification of this data in now shown in Supplementary Figure 5A and B: [...] As you can see, the absence of either PIAS4 or RNF4 causes a delay in the removal of PARP1 from the chromatin fraction. These data are in agreement with the biochemical experiments in Figure 3 and Supplementary Figure 3, where even though the absence of PIAS4 or RNF4 reduces the SUMOylation/ubiquitylation (respectively) of trapped PARP1, some residual PARP1 SUMOylation/ubiquitylation exists, suggesting that although PIAS4 and RNF4 are clearly important in this process, other SUMO E3 ligases and ubiquitin E3 ligases might also play a minor role. With this in mind, we have now modified the model now presented in Figure 6I to include "PIAS4 and other SUMO E3 ligase(s)" and "RNF4 and other ubiquitin E3 ligase(s)".

>>>Reviewer response: OK.

Our response: Thank you.

Minor points:

4. Fig. 1: The expression levels of the engineered PARP1 fusions used should be compared to the level of endogenous PARP1 in the parental cell line.

Our response: We have now assessed this and provided the data in new Supplementary Figure 1A. We also show in Supplementary Figure 1B the relative amounts of PARP1WT-eGFP and PARP1del p.119K120S-eGFP isolated in the RIME experiments.

>>>Reviewer response: OK.

Our response: Thank you.

5. Figs. 2/S2: The assignment of ubiquitylated and/or SUMOylated PARP1 species is not always clear. Sometimes these bands run at ≤150 kDa, sometimes at >>150 kDa. The authors should more clearly label/explain their identity.

Our response: We have now revised these figures to make clear where ubiquitylated and/or SUMOylated species are. For example, Figure 2B is now modified to the following:

In addition, we have provided images of all the uncropped blots, which include molecular marker, in new Supplementary Figure 7.

>>>Reviewer response: OK.

Our response: Thank you.

6. It is somewhat surprising that the authors identified UFD1 but not NPL4 to be involved in PARP1 turnover, even though NPL4 appears to be critical for initializing the unfolding of ubiquitylated substrates by p97/VCP for subsequent proteasomal degradation. Did they identify UFD1 (but not NPL4) in their proteomics datasets? Does depletion of UFD1 (but not NPL4) result in the accumulation of trapped PARP1 on chromatin and/or in the reduction of p97 association with chromatin?

Our response: To address the first question, we did not detect UFD1 nor NPL4 in the original mass spec profiling, but of course this would not necessarily mean that these proteins are not involved in processing trapped PARP1 (for example, the interaction could be transient and/or below the level of detection of mass spec.). To address the second question, we have now assessed the interaction between p97 and PARP1 and the total amount of trapped PARP1 in cells where either UFD1 or NPL4 were depleted (new Figure 4J). This experiment shows that whilst UFD1 depletion decreased the p97-PARP1 interaction and increased the amount of chromatin-associated trapped PARP1, NPL4 depletion did not.
In part, this experiment reproduces our original Figure 3I […] and is also replicated in new Supplementary Figure 5D [...]
We note that this might not be the first description of independent roles for UFD1 and NPL4 in p97 substrate processing. For example, CDT1 is removed from chromatin by p97 in a UFD1-dependent, but NPL4-independent manner (Ramen et al Mol Cell. 2011 Oct 7;44(1):72-84).

Nevertheless, we acknowledge that this is an important point to discuss in the manuscript and have therefore added the following to the revised manuscript:
"Regarding p97 recruitment, our data suggest that UFD1 is required for the recruitment of p97 to trapped PARP1 (Figure 4J). How exactly UFD1 recruits p97 to trapped PARP1 remains to be established. UFD1 is a well-known ubiquitin-chain reader as it possesses Ub-binding domain. In yeast, UFD1 has been shown to bind SUMO (in addition to ubiquitin) and to recruit p97/cdc48 to SUMOylated substrates39, 40. However, UFD1 binding to SUMO has never been demonstrated in mammalian cells. Our data presented here suggests that p97 recruitment to trapped PARP1 depends on RNF4- dependent ubiquitylation; it thus seems likely that UFD1 recruits p97 via its canonical role as an ubiquitin-chain reader, directly bridging p97 and the ubiquitin chains on p97 substrates, in this case ubiquitylated PARP1. We also note that although canonically, UFD1 is thought to function as an obligate heterodimer with NPL4, NPL4 silencing did not alter PARP1 trapping nor the PARP1-p97 interaction in the same way that UFD1 depletion did (Figure 4J). Whilst we are unable to entirely rule out a role for NPL4 in the processing of trapped PARP1, it is possible that this is a function, similar to the removal of CDT1 and other substrates from chromatin7, 32,9, that appears to be UFD1-specific.

>>>Reviewer response: While most of the presented evidence supports the view that PARP1 is an NPL4-

independent target of p97, it should be noted that CuET is a specific inhibitor of NPL4 inducing nuclear clustering of NPL4, but not UFD1 or p97 (Skrott et al., doi 10.1038/nature25016). Consequently, the authors should discuss why CuET treatment phenocopies the treatment with the p97 inhibitor CB-5083.

Our response: Mechanistically, CuET does not achieve the same molecular outcome as NPL4 gene silencing. Gene silencing will reduce the amount of NPL4 protein, which has the potential to alter the substrate binding profile of p97. Conversely, CuET, by disrupting the ZnF motifs of NPL4, causes the entire p97 pool to form into inactive aggregates. As such, CuET use does not assess whether a related phenotype (such as removal of trapped PARP1 from chromatin) is or is not NPL4 dependent, but merely serves as a tool to easily inactivate the entire p97 pool.
This is as stated in *Skrott et al.*: "…the amount of p97 immunoreactivity within the NPL4–GFP clusters correlated with the GFP signal intensity, suggesting that p97 is immobilized via its interaction with NPL4." To acknowledge this, we have revised the main text as follows:

"We also evaluated the effect of CuET, a metabolite of the approved alcohol-abuse drug disulfiram, which segregates p97 from chromatin into inactive agglomerates by disrupting NPL4 ZnF motifs[37]
PMID: 33402676 and thus serves as a tool that inactivates the entire p97 pool. Because of its ability to inactivate the p97 pool by forming agglomerates, CuET has a distinct mechanism of action compared to CB-5083 and also NPL4 or UFD1 gene silencing."

7. The recent publication by the Dantuma lab on the role of PARylation of SUMOylated ATX3 during DNA DSB repair should be discussed (Pfeiffer et al, J Cell Sci 2021; doi:10.1242/jcs.247809).

Our response: We thank the reviewer for drawing out attention to this recent paper. We have now introduced a discussion on ATX3 in the main text as follows:
"Recently, the DUB ATXN3 which antagonises RNF4 ubiquitination activity at DNA damage sites, was shown to be recruited to micro-irradiation induced DNA damage in a PAR-dependent manner39. In combination with our work here, a tantalising hypothesis can be proposed whereby PARPi mediated PARP1 retention coupled with inhibition of ATXN3 recruitment is as a pre-requisite for RNF4-dependent trapped PARP1 ubiquitination. ."

>>>Reviewer response: OK.

Our response: Thank you.

8. Line 264/265: The E1 is the ubiquitin activating enzyme, not a ligase.

Our response: Thank you. This is now corrected.

>>>Reviewer response: OK.

Our response: Thank you.

---end of reviewer 1 comments---

Reviewer #2:

Remarks to the Author:

The authors did an exemplary job in revising this manuscript.

Our response: Thank you.

---end of reviewer 2 comments---

Reviewer #3:

Remarks to the Author:

In their revised study, "The ubiquitin-dependent ATPase p97 removes cytotoxic trapped PARP1 from chromatin", Krastev and colleagues present refined experiments and additional controls that substantially improve what was already, in my view, a strong study.

I commend the authors on their additional work, particularly the inclusion of quantified immunoblots, new add-back controls for RNF4 and dominant negative RNF4 (and p97), edits to improve clarity as to what was done, as well as the new lines of investigation that clarify how RNF4 is contributing to PARP1-Ub in this process.

Based on my original concerns and comments, I now consider this a sufficiently consolidated study and not inclined to ask for any further experiments or revisions. While there are naturally some questions that continue to arise from the revised experiments, I feel that this work is ready for the larger scientific community to view it.

Our response: This is a very important point and we feel that by generating new data to address the RNF4 issue we have now strengthened the manuscript.

---end of reviewer 3 comments---

Final Decision Letter:

Date:	3rd November 21 21:28:52
From:	jie.wang@nature.com
To:	Kristijan.Ramadan@oncology.ox.ac.uk
CC:	ncb@springernature.com
BCC:	rjsproduction@springernature.com;rjsart@springernature.com
Subject:	Decision on Nature Cell Biology submission NCB-R44936B
Message:	

Dear Dr Ramadan,

I am pleased to inform you that your manuscript, "The ubiquitin-dependent ATPase p97 removes cytotoxic trapped PARP1 from chromatin", has now been accepted for publication in Nature Cell Biology.

Thank you for sending us the final manuscript files to be processed for print and online production, and for returning the manuscript checklists and other forms. Your manuscript will now be passed to our production team who will be in contact with you if there are any questions with the production quality of supplied figures and text.

In approximately 10 business days you will receive an email with a link to choose the appropriate publishing options for your paper and our Author Services team will be in touch regarding any additional information that may be required.

You will not receive your proofs until the publishing agreement has been received through our system.

If you have any questions about our publishing options, costs, Open Access requirements, or our legal forms, please contact ASJournals@springernature.com

Before the manuscript is sent to our printers, we will make changes in the text that may be necessary either to make it conform with house style or to make it intelligible to our wide readership. We look particularly carefully at the titles of all papers to ensure that indexing will be accurate and that they are not unreasonably long. We will ask your approval before the copy is finalized, and you will soon receive the edited proofs. Please check the text and figures carefully. Once your manuscript is typeset and you have completed the appropriate grant of rights, you will receive a link to your electronic proof via email with a request to make any corrections within 48 hours. If, when you receive your proof, you cannot meet this deadline, please inform us at rjsproduction@springernature.com immediately.

Once your paper has been scheduled for online publication, the Nature press office will be in touch to confirm the details. An online order form for reprints of your paper is available at https://www.nature.com/reprints/author-reprints.html. All co-authors, authors' institutions and authors' funding agencies can order reprints using the form appropriate to their geographical region.

Publication is conditional on the manuscript not being published elsewhere and on there being no announcement of this work to any media outlet until the online publication date in Nature Cell Biology.

Please note that Nature Cell Biology is a Transformative Journal (TJ). Authors may publish their research with us through the traditional subscription access route or make their paper immediately open access through payment of an article-processing charge (APC). Authors will not be required to make a final decision about access to their article until it has been accepted. Find out more about Transformative Journals

Authors may need to take specific actions to achieve compliance with funder and institutional open access mandates. For submissions from January 2021, if your research is supported by a funder that requires immediate open access (e.g. according to Plan S principles) then you should select the gold OA route, and we will direct you to the compliant route where possible. For authors selecting the subscription publication route our standard licensing terms will need to be accepted, including our self-archiving policies. Those standard licensing terms will supersede any other terms that the author or any third party may assert apply to any version of the manuscript.

To assist our authors in disseminating their research to the broader community, our SharedIt initiative provides you with a unique shareable link that will allow anyone (with or without a subscription) to read the published article. Recipients of the link with a subscription will also be able to download and print the PDF.

If your paper includes color figures, please be aware that in order to help cover some of the additional cost of four-color reproduction, Nature Research charges our authors a fee for the printing of their color figures. Please contact our offices for exact pricing and details.

As soon as your article is published, you will receive an automated email with your shareable link.

If you have not already done so, we strongly recommend that you upload the step-by-step protocols used in this manuscript to the Protocol Exchange (www.nature.com/protocolexchange), an open online resource established by Nature Protocols that allows researchers to share their detailed experimental know-how. All uploaded protocols are made freely available, assigned DOIs for ease of citation and are fully searchable through nature.com. Protocols and the Nature and Nature research journal papers in which they are used can be linked to one another, and this link is clearly and prominently visible in the online versions of both papers. Authors who performed the specific experiments can act as primary authors for the Protocol as they will be best placed to share the methodology details, but the Corresponding Author of the present research paper should be included as one of the authors. By uploading your Protocols to Protocol Exchange, you are enabling researchers to more readily reproduce or adapt the methodology you use, as well as increasing the visibility of your protocols and papers. You can also establish a dedicated page to collect your lab Protocols. Further information can be found at www.nature.com/protocolexchange/about

You can use a single sign-on for all your accounts, view the status of all your manuscript submissions and reviews, access usage statistics for your published articles and download a record of your refereeing activity for the Nature journals.

Please feel free to contact us if you have any questions.

With kind regards,

Jie Wang, PhD
Senior Editor
Nature Cell Biology

Tel: +44 (0) 207 843 4924
email: jie.wang@nature.com

Click here if you would like to recommend Nature Cell Biology to your librarian
http://www.nature.com/subscriptions/recommend.html#forms

** Visit the Springer Nature Editorial and Publishing website at www.springernature.com/editorial-and-publishing-jobs for more information about our career opportunities. If you have any questions please click here.**
